# Photocatalytic Hydrogenation of Quinolines to Form 1,2,3,4‐Tetrahdyroquinolines Using Water as the Hydrogen Atom Donor

**DOI:** 10.1002/anie.202502864

**Published:** 2025-05-08

**Authors:** Jingjing Zhang, Nico Spreckelmeyer, Jessika Lammert, Maxim‐Aleksa Wiethoff, Matthew James Milner, Christian Mück‐Lichtenfeld, Armido Studer

**Affiliations:** ^1^ Organisch‐Chemisches Institut Universität Münster 48149 Münster Germany; ^2^ Center for Multiscale Theory and Computation Universität Münster 48149 Münster Germany

**Keywords:** Hydrogen atom transfer, Hydrogenation, Isomerization, Photocatalysis, Water activation

## Abstract

The design of a sequential process combining hydrogenation and a subsequent stereomutation is an attractive strategy for the stereoselective reduction of cyclic disubstituted π–systems to access the thermodynamically more stable *trans* isomer, which would be the minor compound considering a kinetically controlled *cis* hydrogenation process. Herein, we demonstrate stereoselective photocatalytic phosphine‐mediated quinoline reductions with water as the hydrogen atom source under mild conditions to afford the corresponding 1,2,3,4‐tetrahydroquinolines with complete selectivity towards reduction of the heteroaromatic part. The method shows broad functional group tolerance and provides access to *trans*‐2,3‐disubstituted tetrahydroquinolines with moderate to excellent diastereoselectivity. These *trans* isomers are not readily obtained using established methods, as transition‐metal‐catalyzed regioselective quinoline hydrogenations provide the corresponding *cis*‐2,3‐disubstituted isomers with high selectivity. Mechanistic studies reveal that the hydrogenation of the 2,3‐disubstituted quinolines proceeds through a cascade process comprising an initial *cis* selective photocatalytic hydrogenation of the heteroarene core of the quinoline, followed by a *trans* selective photoisomerization.

## Graphical abstract

A sequential process combining hydrogenation and a subsequent stereomutation is presented for *trans* selective hydrogenation of quinolines using water as the hydrogen atom source. Mechanistic studies reveal that the hydrogenation proceeds through a cascade process comprising an initial *cis* selective photocatalytic hydrogenation of the heteroarene core of the quinoline followed by a *trans* selective photoisomerization.

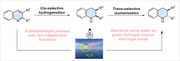

## Introduction

**Figure 1:**
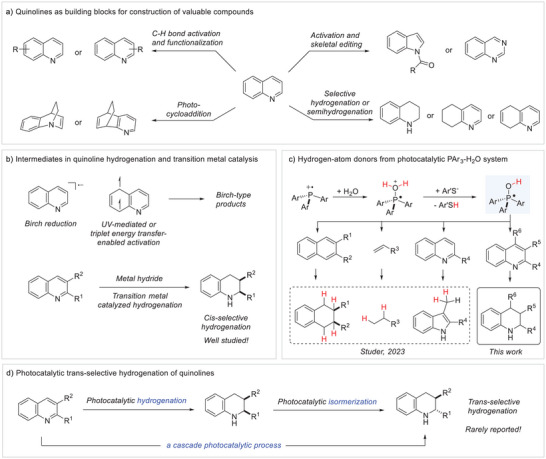
a) Chemical modification of quinolines through peripheral and skeletal editing as well as through photocycloaddition reactions. b) State of the art in the quinoline hydrogenation and (c, d) suggested *cis* selective hydrogenation of quinolines through radical hydrogenation by phosphine‐mediated water activation, followed by epimerization.

Chemical modification of heteroarene skeletons allows the construction of diverse valuable heterocycles, which are typical building blocks in bioactive compounds, natural products, and industrial materials.^[^ ^1^, ^2^, ^3^, ^4^, ^5^ ^]^ Such heteroarene manipulation is a valuable strategy in synthesis, in particular if abundant, readily available heteroarenes are used as the substrates for diversification. Quinolines that are rather reactive heteroarenes by virtue of their reduced aromaticity have often been studied along those lines. The functionalization of quinolines through regioselective C─H bond activation either at the arene‐ or heteroarene moiety has been heavily investigated (Figure 1a).^[^ ^6^, ^7^, ^8^, ^9^ ^]^ Moreover, skeletal editing of quinolines has been successfully applied to reorganize the core structure of the N‐heteroarene through formal single‐atom insertion and deletion.^[^ ^10^, ^11^ ^]^ Recently, there has been a growing interest in the dearomatization of heteroarenes for the construction of three‐dimensional heterocyclic compounds. This trend aligns with the “escape from flatland” concept, which emphasizes the enhancement of molecular complexity, beginning with straightforward and easily accessible planar arenes.^[^ ^12^, ^13^, ^14^ ^]^ Towards this end, photocycloaddition of quinolines has been realized through dearomatization of the arene‐ as well as the heteroarene entity of the quinoline structure.^[^ ^15^, ^16^, ^17^, ^18^ ^]^ Despite these significant achievements, quinoline hydrogenation remains the predominant strategy for “escaping from flatland”, owing to consistent contributions in Birch^[^ ^19^, ^20^, ^21^, ^22^, ^23^, ^24^, ^25^, ^26^ ^]^ and transition‐metal‐catalyzed reductions.^[^ ^27^, ^28^, ^29^ ^]^ Classical Birch reduction by solvated electrons converts the heteroarene into a radical anion intermediate, which is then protonated to afford the corresponding dihydrogenated product after renewed reduction and protonation (Figure 1b). UV‐mediated or triplet energy transfer‐enabled heteroarene reductions offer a photochemical protocol to produce Birch‐type products proceeding through diradical intermediates.^[^ ^30^, ^31^, ^32^ ^]^ Most practically, various hydrogenation methods have been developed using transition‐metal catalysis, thereby also advancing related investigations on transition‐metal hydride species.^[^ ^33^, ^34^ ^]^ Considering the hydrogenation of 2,3‐disubstituted quinolines, the *cis* selective reduction is highly preferred (Figure 1b),^[^ ^35^, ^36^, ^37^, ^38^, ^39^, ^40^ ^]^ while the corresponding *trans*selective hydrogenation is still underexplored, even it reflects the thermodynamically more favorable process.^[^ ^41^, ^42^ ^]^

Recently, it has been demonstrated that photocatalytic deracemization is a promising strategy to construct chiral amine derivatives with high enantioselectivity.^[^ ^43^, ^44^, ^45^, ^46^, ^47^, ^48^, ^49^, ^50^ ^]^ These stereoconvergent processes proceed through a photocatalyzed oxidative generation of achiral α‐aminoalkyl radicals with subsequent reduction by a chiral thiol. Guided by such a strategy, we assumed that a *cis* selective photocatalytic hydrogenation of 2,3‐disubstituted quinolines may eventually result in the corresponding *trans* isomer, if the initial *cis* product is readily epimerized to its thermodynamically more stable *trans* isomer through a photocatalytic cascade process. As for the enantioconvergent processes, stereomutation should proceed through the corresponding α‐aminoalkyl radicals.^[^ ^43^, ^50^ ^]^

Recently, our group reported that alkenes and naphthalenes can be efficiently reduced via direct HAT from Ar_3_POH‐type radicals enabled by a photocatalytic phosphine‐mediated water activation system applying thiol cocatalysis (Figure 1c).^[^ ^51^ ^]^ The Ar_3_POH‐radicals containing a very weak O─H bond (<10 kcal mol^−1^) are readily formed by trapping of oxidatively generated triaryl phosphine radical cations with water and subsequent deprotonation. Considering the hydrogenation of 2,3‐disubstituted naphthalenes, *cis*‐2,3‐disubstituted tetrahydronaphthalenes were obtained with excellent diastereoselectivity in our initial study. We therefore assumed that the partial hydrogenation of quinolines to give 1,2,3,4‐tetrahydroquinolines with our photocatalytic water activation system should be feasible. A first challenge to be overcome lies in the selective hydrogenation of the heteroarene part of the quinoline moiety and skeletal editing to indoles, as noted for a few cases before,^[^ ^51^, ^52^ ^]^ should be suppressed. Further, for 2,3‐disubstituted quinolines, we expected in analogy to the naphthalenes the *cis* stereoisomer to be formed as the kinetic product, which potentially might directly be epimerized under the photocatalytic hydrogenation conditions to the thermodynamically more stable *trans* isomer (Figure 1d).

## Results and Discussion

**Figure 2:**
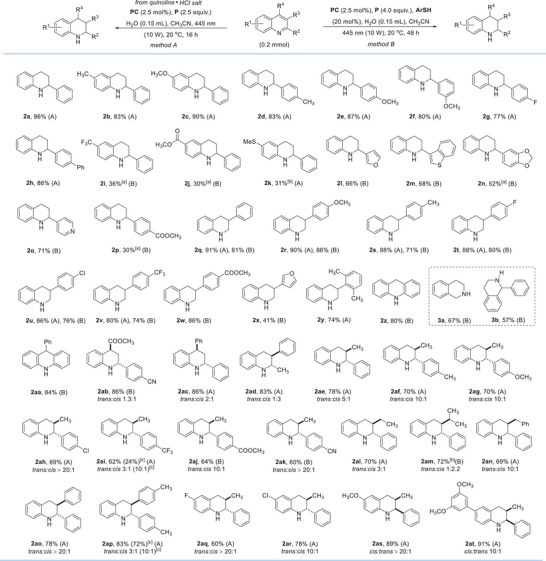
Radical hydrogenation of differently substituted quinolines. All reactions were conducted using a quinoline or its HCl‐salt (1.0 equiv., 0.2 mmol). Applying method A, quinoline HCl‐salts were used as the starting materials, see the Supporting Information for further details. Isolated yields are provided. The *trans*/*cis* ratio was determined by ^1^H‐NMR spectroscopy. ^[a]^ The hydrogenation was accompanied by the formation of the corresponding indole as the byproduct, see Supporting Information for details. ^[b]^ Conducted with (*p*‐CF_3_C_6_H_4_)_3_P in place of (*p*‐MeOC_6_H_4_)_3_P. ^[c]^ Yields and *trans*/*cis* ratios obtained through extending reaction time to 36 h.

To experimentally validate the feasibility of quinoline hydrogenation, the reduction of 2‐phenyl quinoline (**1a**) was tested using tris(4‐methoxyphenyl)phosphine (**P**) in combination with [Ir(dF(CF_3_)ppy)_2_(dtbbpy)](PF_6_)] (dF(CF_3_) ppy, 2‐(2,4‐difluorophenyl)‐5‐trifluoromethylpyridine; dtbbpy, 4,4′‐di‐*tert*‐butyl‐2,2′‐bipyridine, **PC**) as the photocatalyst and H_2_O as the hydrogen source in acetonitrile under irradiation with a 10 W blue light‐emitting diode (LED) (Figure 2). After 48 h, only a small amount of the hydrogenated tetrahydroquinoline **2a** (10%) was formed (Table 1, entry 1). Importantly, hydrogenation occurred with complete regiocontrol at the heteroarene moiety to give the 1,2,3,4‐tetrahydroquinoline. There was no significant increase in the yield of **2a** in the presence of a catalytic or a stoichiometric amount of a thiol co‐catalyst **ArSH** (entries 2 and 3). In these cases, significant amounts of a byproduct were formed (see the Supporting Information). However, by switching to the quinoline hydrochloride salt **1a**‐HCl as the substrate, radical hydrogenation afforded **2a** as a single product in 88% yield without the aid of any **ArSH** (entries 4 and 5). Yield decreased to 62% when using 1.0 equivalent of HCl as a pre‐activator (entry 6). Reaction time could be shortened to 16 h without affecting efficiency, and **2a** was obtained in 86% isolated yield (entry 7). As expected, the reaction did not proceed in the absence of the photocatalyst **PC** and only a very low yield was noted in the absence of the phosphine **P**, as both are required for the chemical transformation of water into the active H–atom donor, the Ar_3_POH‐radical (entries 8 and 9). Other thiols and other photocatalyst tested provided worse results, see Supporting Information.

**Table 1:** Optimization of the conditions for the hydrogenation of quinoline **1a**.

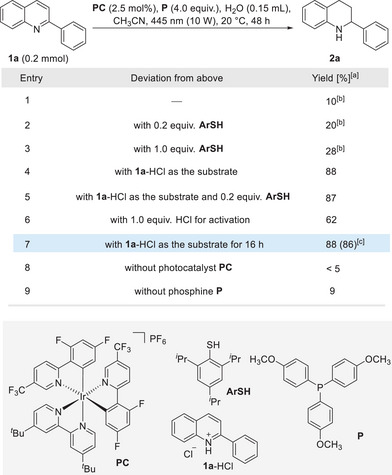

^[a]^ Yield was determined by ^1^H‐NMR analysis of the crude product using dibromomethane as an internal standard.

^[b]^ The reduction was accompanied by the formation of a byproduct, see Supporting Information for details.

^[c]^ 2.5 equivalents of **P** was applied. Isolated yield in parenthesis.

With the optimized conditions in hand (method A, see Table 1 entry 7), we next investigated the scope of the photocatalytic quinoline hydrogenation (Figure 2). In several cases, we found that the substrate hydrochloride was not stable under the reaction conditions, and the formation of unidentified side products was noted. For these substrates, the reaction of the non‐protonated quinoline using an arylthiol as a cocatalyst (method B) delivered the better results. Taken together, method A was efficient for the hydrogenation of electron‐rich quinolines, and method B was utilized for the reduction of electron‐poor or acid‐sensitive quinolines.

2‐Phenylquinolines with electron‐donating groups such as methyl or methoxy at the 6‐position delivered the corresponding tetrahydroquinolines **2b** and **2c** in high yields using method A. We found that the electronic effects of the 2‐aryl substituent are moderate, and the *para*‐methyl, *para*‐methoxy, *para*‐fluoro and *para*‐phenyl derivatives all delivered the targeted hydrogenation products **2d**–**2 h** in good yield (77%–87%, method A). Method A was not efficient for quinolines bearing electron‐withdrawing groups (CF_3_ or CO_2_Me) at the 6‐position. However, hydrogenation for these systems was achieved using method B. Yields were moderate (**2i**, **2j**, and **2p**) due to the formation of indole rearrangement side product (see Supporting Information). Thioethers that are often problematic in transition‐metal‐catalyzed hydrogenations are tolerated, albeit product **2k** was isolated in moderate yield. Pleasingly, heteroaryl substituents are tolerated at the 2‐position, and the 2‐furyl (**2l**), 2‐benzothienyl (**2m**), benzodioxole (**2n**), and 4‐pyridinyl (**2o**) tetrahydroquinolines were isolated in 52%–71% yields (method B). Importantly, competing hydrogenation of the 2‐heteroaryl moiety in all these systems was not observed documenting the high chemoselectivity toward hydrogenation of the quinoline heteroarene entity, despite not being activated through protonation. Notably, both methods could be utilized in most cases for the reduction of 3‐substituted quinolines (**2q**–**2y**) with high functional group compatibility, leading to the corresponding tetrahydroquinolines in good yields. If substrates bear an acid‐sensitive group, such as an ester or a furyl substituent, method B had to be applied (**2w**, **2x**). The quinoline bearing a bulky ortho, ortho′‐dimethylphenyl group at the 3‐position, could not be reduced without HCl activation, presumably due to steric effects (**2y**). Extended fused heteroarenes, acridine (**2z**) and a derivative (**2aa**) were regioselectively hydrogenated. Moreover, the parent isoquinoline and its phenyl‐substituted congener were eligible substrates applying method B to provide the corresponding tetrahydroisoquinolines **3a** and **3b** in good yields.

Stereoselective hydrogenations were investigated next, first focusing on 2,4‐disubstituted quinolines. A mixture of diastereoisomers (**2ab** and **2ac**) were obtained with the *trans* product being formed as the major isomer in very good yield. Notably, a subtle *trans* selectivity was observed in these reductions, contrasting with the typical *cis* selectivity achieved through transition‐metal‐catalyzed hydrogenation protocols. Encouraged by this discovery, the stereoselective hydrogenation of 2,3‐disubstituted quinolines were examined. For the 2‐methyl‐3‐phenyl‐quinoline, the *cis* product was formed as the major isomer (3:1) in good chemical yield, while the 3‐methyl‐2‐phenyl isomer provided the *trans* product with 5:1 diastereoselectivity (see **2ad** and **2ae**). Interestingly, the 2‐aryl substituent showed a strong electronic effect on the stereoselectivity. Thus, the *para*‐methyl, *para*‐methoxy, and *para*‐chloro‐phenyl 3‐methyl‐quinolines delivered the *trans* products with very good (10:1, **2af** and **2ag**) to excellent (>20:1, **2ah**) selectivity, while a modest 3:1 *trans* selectivity was achieved for the CF_3_‐congener (**2ai**). All these stereoselective reductions were conducted using method A. For the *para*‐cyanophenyl and the *para*‐methoxycarbonylphenyl quinolines we switched to method B, and in both cases, a high *trans* selectivity was achieved (**2aj**, **2ak**). The effect of the 3‐substituent on the selectivity was also evaluated, keeping the phenyl substituent at the 2‐position. As compared to the methyl derivative, a slightly lower *trans *selectivity was measured for the ethyl system (3:1, **2al**) and the sterically more hindered isopropyl‐derivative was hydrogenated with moderate *cis* selectivity (**2am**). High *trans* selectivity was obtained for the 3‐benzyl and 3‐phenyl‐substituted quinolines (10:1 and >20:1 for **2an** and **2ao**).

Surprisingly, a significantly reduced selectivity was noted for the more electron‐rich 2‐(*para*‐tolyl)‐3‐(*para*‐tolyl)‐quinoline (see **2ap**). We further found that the diastereoselectivity is strongly influenced by the electronic nature of the 6‐substituent of the quinoline skeleton, with high *trans* stereocontrol observed for the fluoro and chloro systems (see **2aq** and **2ar**) and high *cis* selectivity for the methoxy‐ (**2as**) and 3,5‐dimethoxyphenyl‐substituted quinolines (**2at**). Of note, higher *trans* diastereoselectivities were obtained through extending reaction time to 36 h, albeit lower yields resulted under these conditions (**2ai** and **2ap**).

**Figure 3:**
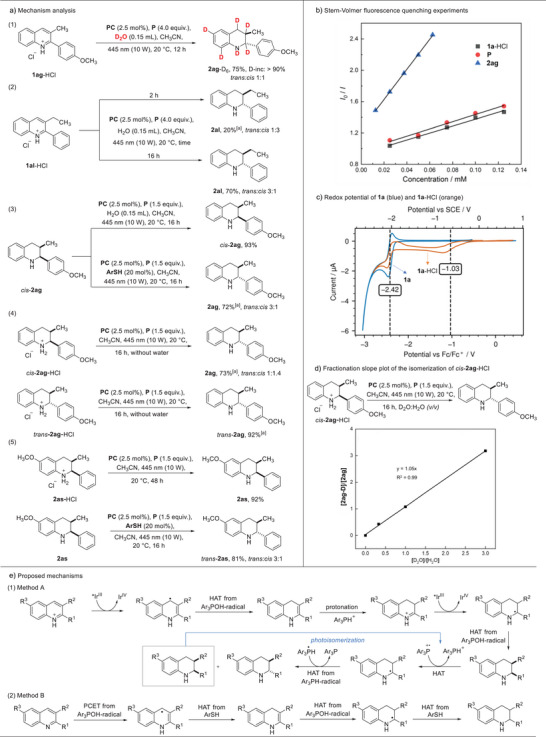
Mechanism investigation. ^[a]^ Yields were determined by ^1^H‐NMR spectroscopy.

In order to figure out the reaction mechanism, a series of deuterium labeling and control experiments were conducted. Running the hydrogenation of **1ag** in the presence of D_2_O revealed that water is the hydrogen source in this reduction, and the deuterated **2ag**‐D_6_ was obtained with >90% deuterium incorporation (Figure 3a, Equation 1). As compared to the reaction with H_2_O a significantly lower *trans*/*cis* diastereoselectivity was noted (10:1 versus 1:1), probably due to the higher energy requirement to break the *α*‐amino C‐D bond of **2ag**‐D_6_ in the photocatalyzed isomerization process (see below). The deuterium labeling at positions 6 and 8 in the benzene ring of **2ag**‐D_6_ was likely caused by a Brønsted acid‐mediated H/D exchange.^[^ ^53^, ^54^ ^]^ To confirm that the *trans* selectivity in these hydrogenations is a result of a stereoinversion at the 2‐position after initial *cis* selective hydrogenation, the diastereoselectivity for the reduction of **1al**·HCl was measured after 2 h and the end of the reaction (Equation 2). Indeed, a 3:1 ratio favoring the *cis* isomer was observed after 2 h (20% yield), while the final product was isolated with a 3:1 *trans*/*cis* selectivity in 70% yield (16 h). To further explore the photocatalyzed isomerization, isomerically pure *cis*‐**2ag** was prepared through a reported method and subjected to our standard conditions (method A). Isomerization did not occur and *cis*‐**2ag** was recovered in high yield (Equation 3). Replacing water with a thiol cocatalyst led to epimerization under otherwise identical conditions (*trans*/*cis* = 3:1), showing that epimerization through the α‐aminoalkyl radical is feasible.^[^ ^43^, ^50^ ^]^ Switching to the HCl‐protonated tetrahydroquinoline *cis*‐**2ag·**HCl as the substrate in the absence of water, partial isomerization could be achieved (*trans*/*cis* = 1:1.4, Equation 4), which indicates that the Ar_3_POH‐radical is likely not involved in the *cis* to *trans* epimerization process. Importantly, *trans*‐**2ag·**HCl was inert under these isomerization condition, indicating that only the *cis* isomer (*cis*‐**2ag**) engages in the isomerization process. Of note, for electron rich quinolines where the *cis* product is formed exclusively in our hydrogenation (see **2as**), isomerization could not be realized starting from *cis*‐**2as·**HCl under the same conditions (Equation 5). This experiment further indicated that for all other compounds, the *cis* diastereoisomer is the likely formed initial major product, as steric effects for **1as** and other congeners are very similar. Notably, we found that for these “inert” compounds, partial epimerization was achieved via a synergistic photocatalytic strategy with a thiol co‐catalyst **ArSH** (Equation 5), further generalizing our approach towards *trans*‐2,3‐disubstituted tetrahydroquinolines also for electron‐rich systems through a two‐step sequence.

We also conducted Stern‐Volmer fluorescence quenching experiments. As shown in Figure 3b, both (*p*‐MeOC_6_H_4_)_3_P (**P**) and the quinoline HCl‐salt of **1a** can quench the excited photocatalyst **PC** with comparable rates, and the reductive quenching by the product tetrahydroquinoline **2ag** was measured to be around 5 times faster. Further, the selected Ir‐photocatalyst **PC** with an oxidation potential of *E* ^0^_ox_(*Ir^III^/Ir^IV^) = −0.89 V vs. SCE in its excited state is not able to reduce **1a** (*E* ^0^_red_ = −2.04 V vs. SCE, as measured by cyclic voltammetry, see Figure 3c). For the HCl‐salt of **1a** reduction occurs at a higher potential (*E* ^0^_red_ = −0.65 V vs. SCE), and reduction with the excited photocatalyst becomes feasible, in agreement with the Stern‐Volmer studies. Moreover, inspired by a recent report of Knowles, a fractionation slope plot was measured for the isomerization of *cis*‐**2ag·**HCl (Figure 3d).^[^ ^55^ ^]^ A slope of 1.05 was determined, which indicated that the isomerization should proceed through a sequential HAT/HAT process, as initial quinoline SET oxidation with subsequent deprotonation, followed by HAT, should lead to a smaller slope (<0.5). Moreover, for a mechanism proceeding through initial HAT followed by SET reduction of the α‐aminoalkyl radical and protonation, a larger slope (>2) is expected. This is in line with the fact that the α‐methyl quinoline also engaged in the epimerization (see **2ad**), where SET‐reduction of the nucleophilic α‐aminyl‐α‐methyl radical is not likely.

Based on these results and our previous findings,^[^ ^51^ ^]^ the following mechanism is suggested for the hydrogenation of the quinoline HCl‐salts (method A). The protonated quinoline first gets reduced by the excited Ir(III)‐photocatalyst to give the corresponding delocalized radical, which can then be reduced through HAT by the Ar_3_POH‐radical that is generated from the oxidized phosphine upon trapping with water and deprotonation (see Figure 1c). The reversed order to access the same intermediate through initial reduction of the quinolinium salt by HAT with the Ar_3_POH‐radical followed by SET‐reduction with the photocatalyst is also possible, based on the Stern‐Volmer studies. The formed enamine can then be protonated to the iminium ion that is reduced by the photocatalyst to the stabilized (likely longer lived) α‐aminoalkyl radical,^[^ ^56^ ^]^ which can then react with an Ar_3_POH‐radical with high diastereoselectivity to the *cis*‐tetrahydroquinoline as the kinetic product. We have prepared the 1,4‐dihydroquinoline (enamine intermediate, for 2‐phenylquinoline) in situ through an alternative method and subjected it to the standard hydrogenation conditions. The corresponding tetrahydroquinoline was obtained in 74% ^1^H‐NMR yield, showing that the enamine is a feasible intermediate in this cascade (see Supporting Information). *Cis* to *trans* isomerization will then likely proceed through oxidation of (*p*‐MeOC_6_H_4_)_3_P by the Ir‐photocatalyst, to give the corresponding (*p*‐MeOC_6_H_4_)_3_P radical cation, which can then abstract a hydrogen atom from the quinoline to regenerate the α‐aminoalkyl radical. The protonated phosphine (*p*‐MeOC_6_H_4_)_3_PH^+^, *E* ^0^_red_(Ph_3_PH^+^) ∼−1.32 V^[^ ^57^ ^]^ vs. SCE should be reduced by the Ir^II^‐complex (*E* ^0^_ox_(Ir^II^/Ir^III^) = −1.37 V vs. SCE), closing the epimerization redox catalytic cycle. We suggest that the (*p*‐MeOC_6_H_4_)_3_PH‐radical thus generated is then reducing the α‐aminoalkyl radical to the tetrahydroquinoline, as supported by the fractionation slope studies. In contrast to the initial reduction with the Ar_3_POH‐radical, reduction with the (*p*‐MeOC_6_H_4_)_3_PH‐radical likely occurs with lower *cis* diastereoselectivity, which eventually leads to partial epimerization with formation of the thermodynamic *trans* product. To evaluate the feasibility of the suggested HAT from an α‐arylated tetrahydroquinoline (THQ) to a triaryl phosphine radical cation, DFT calculations were conducted (see Supporting Information). THQ **2a** was used as the model compound, and it was found that the HAT from **2a** by (Ph_3_P)^+^ to be exergonic in acetonitrile as the solvent (*ΔG_s_* (298 K) = −11.5 kcal/mol). This HAT proceeds with a very low barrier of 1.8 kcal/mol. Moreover, the reduction of the corresponding α‐aminoalkyl radical by the (Ph)_3_PH‐radical to give **2a** is strongly exothermic (*ΔG_s_* (298 K) = −56.0 kcal/mol), and we assume that this second HAT proceeds without any significant barrier.

We believe that under neutral conditions, starting with the neutral quinoline, the concentration of the protonated phosphine (*p*‐MeOC_6_H_4_)_3_PH^+^ is too low for an efficient generation of the (*p*‐MeOC_6_H_4_)_3_PH‐radical and therefore, epimerization cannot proceed in such cases. For the more basic tetrahydroquinolines such as **2as**, the phosphine is not able to efficiently deprotonate the quinolinium salt (see Supporting Information), which suppresses the overall epimerization process. Based on this observation, we also tested the hydrogenation of **1ag** (method A) with phosphines that show lower basicity, which should according to our mechanistic hypothesis reveal lower photoisomerization efficiency. Indeed, with (*p*‐CF_3_C_6_H_4_)_3_P (*trans*/*cis* = 2.5:1, 93%) and (*p*‐FC_6_H_4_)_3_P (*trans*/*cis* = 2.7:1, 92%, see Supporting Information), significantly lower *trans* selectivities were obtained as compared to the (*p*‐MeOC_6_H_4_)_3_P‐mediated reaction (*trans*/*cis* = 10:1, 70%).

Regarding method B, the following mechanism is proposed.^[^ ^43^ ^]^ The oxidatively generated Ar_3_POH‐radical first engages in a proton coupled electron transfer (PCET) to the neutral quinoline to give the H‐adduct radical, which is then reduced by the thiol cocatalyst **ArSH** to give a 2,3‐disubstituted 1,4‐dihydroquinoline. We calculated all other possibilities for potential HAT from the Ar_3_POH‐radical to the neutral quinoline and found all processes to be higher in energy than the PCET process (see Supporting Information for details). Subsequently, we assume that a HAT from a second Ar_3_POH‐radical to the 1,4‐dihydroquinoline is occurring that will lead to the corresponding carbon radical, which is eventually reduced by the thiol. We believe that the thiol reduction occurs with high but not complete *cis* selectivity. Under the applied conditions, *cis* to *trans* epimerization is possible through oxidative regeneration of the α‐aminoalkyl radical (see Figure 3e).^[^ ^58^ ^]^

## Conclusion

In summary, a stereoselective hydrogenation of substituted quinolines using water as the hydrogen atom source is presented. The reaction uses photoredox catalysis in combination with a triarylphosphine to mediate the water activation process. Reduction works for differently substituted quinolines selectively at the heteroarene ring of the quinoline moiety. Special high‐pressure equipment and H_2_ are not required to run these hydrogenations. For 2,3‐disubstituted quinolines, radical hydrogenation provides the *trans*‐2,3‐disubstituted 1,2,3,4‐tetrahydroquinolines in high yield and good to excellent diastereoselectivity. Importantly, transition‐metal‐catalyzed hydrogenation of such substrates with H_2_ leads to the *cis*‐2,3‐disubstituted products, rendering the synthesis of the corresponding *trans* isomers challenging using state‐of‐the‐art methodology. Mechanistic studies revealed that these cascades proceed through a sequence comprising initial *cis* selective hydrogenation followed by photoredox catalyzed epimerization to provide the thermodynamically more stable *trans*‐products. The redox catalyst in charge of the water activation process is also in operation for the *cis* to *trans* isomerization, avoiding catalyst switching for the realization of multiple independent processes. This transformation represents another example to document the synthetic value of our photocatalytic phosphine‐mediated water activation strategy.

## Supporting Information

The authors have cited additional references within the Supporting Information.

## Conflict of Interests

The authors declare no conflict of interest.

## Supporting information



Supporting information

## Data Availability

The data that support the findings of this study are available in the supplementary material of this article.
