# Supplementary material for: Photocatalytic Hydrogenation of Quinolines to Form 1,2,3,4‐Tetrahdyroquinolines Using Water as the Hydrogen Atom Donor
Source: Angew Chem Int Ed Engl. 2025 May 8;64(25):e202502864. doi: 10.1002/anie.202502864 (PMC12171667; doi:10.1002/anie.202502864)
Supplement: Supplementary file 1 — Supporting information [file ANIE-64-e202502864-s001.pdf]

Photocatalytic Hydrogenation of Quinolines to Form 1,2,3,4-Tetrahdyroquinolines using Water as the Hydrogen Atom Donor

Jingjing Zhang, Nico Spreckelmeyer, Jessika Lammert, Maxim-Aleksa Wiethoff, Matthew James Milner, Christian Mück-Lichtenfeld and Armido Studer*

Contents

1. General information 2
2. The synthesis of starting materials 4
3. Reaction optimization 10
4. Synthesis and characterization of products 13
    4.1 General procedures 13
    4.2 Synthesis and characterization of product **2** and **3** 14
    4.3 C-H functionalization of 2-substituted quinolines 42
5. Stern-Volmer quenching experiments 48
6. Cyclic voltammetry 49
7. Fractionation slope measurement 53
8. Deuteration experiment 54
9. Time course of the *trans/cis* selectivity 56
10. The investigation of the photoisomerization intermediate 57
11. Proton transfer equilibrium 62
12. Effect of phosphine on the diastereoselectivities 65
13. The hydrogenation of 1,4-dihydroquinoline intermediate 68
14. Kinetic isotope effect 69
15. DFT calculations 69
16. NMR spectra 134
17. References 208

1. General information

Chemicals: All reactions involving air- or moisture-sensitive reagents or intermediates were carried out in pre-heated glassware using standard Schlenk techniques at ambient room temperature, unless otherwise specified. All commercially available reagents were purchased from Sigma-Aldrich, Alfa Aesar, TCI Chemicals, Acros Organics or ABCR in the highest purity grade and used without further purification. The following solvents (ACROS ExtraDry solvents with ACROSeal® cap) were purchased from ACROS Organics, stored under 3 or 4 Å activated molecular sieves and collected under positive argon pressure: acetonitrile (CH_3CN), dimethylformamide (DMF), dimethylacetamide (DMA), chloroform ($CHCl_3$) and methanol (CH_3OH). Anhydrous tetrahydrofuran (THF) was refluxed over sodium and freshly distilled from potassium prior to use. Thin layer chromatography (TLC) was performed on Merck silica gel 60 F-254 plates and visualized by fluorescence quenching under UV light. Column chromatography was performed on Merck or Fluka silica gel 60 (40-63 μm).

NMR Spectroscopy: ^{1}H-NMR, ^{13}C-NMR and ^{19}F-NMR spectra were recorded using a Bruker DPX 300, Bruker AV 300, Bruker AV 400, Agilent DD2 500 or Agilent DD2 600 spectrometer at 299 K or 300 K. ^{1}H-NMR chemical shifts are given relative to TMS and residual monoprotic solvent peaks were used as an internal reference for ^{1}H-NMR ($CDCl_3$: $\delta = 7.26$ ppm; DMSO: $\delta = 2.50$ ppm) and ^{13}C-NMR spectra ($CDCl_3$: $\delta = 77.16$ ppm; DMSO: $\delta = 39.52$ ppm). ^{31}P-NMR spectra are referenced according to the proton signal as the primary reference for the unified chemical shift scale. Coupling constants (J) are quoted to the nearest 0.1 Hz. The following abbreviations (or combinations thereof) were used to describe ^{1}H-, ^{13}C and ^{31}P-NMR multiplicities: s = singlet, broad singlet = brs, d = doublet, t = triplet, q = quartet, hept = heptet, m = multiplet.

Mass Spectrometry: High-resolution (HRMS) ESI (m/z) spectra were measured on a Bruker MicroTof or ThermoFisher Scientific LTQ XL Orbitrap. High-resolution APCI (m/z) spectra were measured on a Thermo-Fisher Scientific Orbitrap LTQ XL. High-resolution EI (m/z) spectra were measured on a Thermo-Fischer Scientific Exactive GC Orbitrap GC-MS System. MassLinx 4.0 of Water-Micromass was used for data analysis.

GC-FID: GC-FID was conducted on an Agilent GC 6890 equipped with a flame ionization detector (FID) and an Agilent HP-5, Methyl Siloxan (Model No: 19091Z-413) column using H_2 as carrier gas with a flow rate of 1.5 mL min^{-1}. The method used starts with the injection temperature T_0, the column is heated to temperature T_1 (ramp) and this temperature is held for an additional time t ($T_0 = 50$ °C, $T_1 = 300$ °C, ramp = 10 °C min^{-1}, $t = 15$ min).

Light Sources: Photochemical reactions were performed with one LED lamp (10 W, 445 nm) as light source. The reaction temperature was kept at 20 °C using a circulating water system.

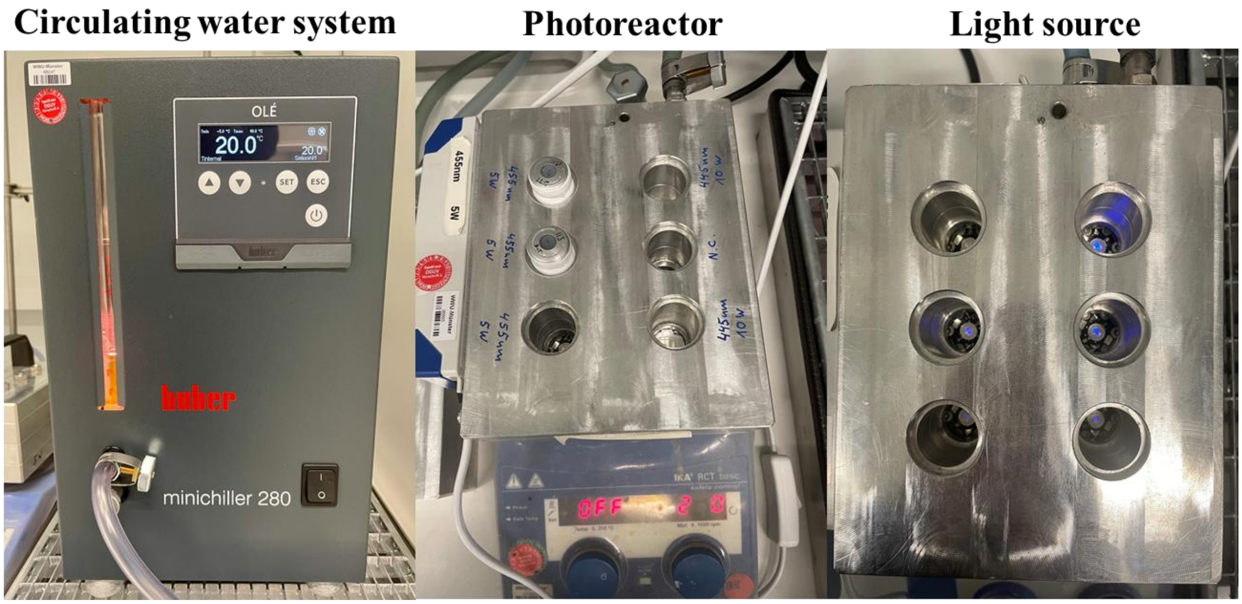

Figure S1. Photoreactors used in this work (445 nm, 10 W).

2. The synthesis of starting materials

Some substrates used in this work are commercially available unless otherwise specified. Substrates **1b**, **1c**, **1d**, **1e**, **1h**, **1f**, **1g**, **1i** and **1j** were synthesized through the reported coupling methods.[1-3] Substrates **1k**, **1l**, **1m**, **1n** and **1o** were synthesized through the reported methods.[4-5] 3-Substituted quinolines **1p**, **1q**, **1r**, **1s**, **1t**, **1u**, **1v** and **1w** were also synthesized through the reported methods.[6-7] Exemplified synthesis procedures for several quinolines are shown as below.

The synthesis of 1i:

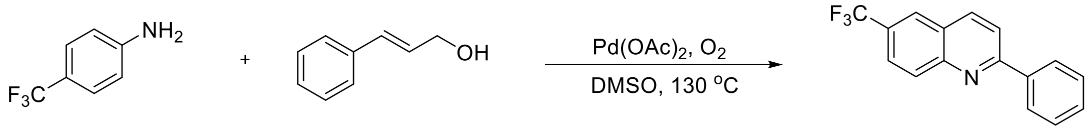

Allyl alcohol (0.50 mmol), aniline (0.50 mmol), $Pd(OAc)_2$ (11.2 mg, 10 mol%) and DMSO (2.0 mL) were added into a test tube attached to an oxygen balloon (1 atm). The reaction was stirred and heated at 130 °C for 12 h. Then the reaction was quenched by the addition of 10 mL water. The aqueous solution was extracted with ethyl acetate (20 mL) and the extract was dried with anhydrous Na_2SO_4. The solvent was removed and the crude product was isolated by flash chromatography on silica gel (pentane/ EtOAc 4/1).

^{1}H NMR (300 MHz, $CDCl_3$) δ 8.27 (d, *J* = 8.7 Hz, 2H), 8.19 (dd, *J* = 8.1, 1.6 Hz, 2H), 8.15 – 8.11 (m, 1H), 7.96 (d, *J* = 8.6 Hz, 1H), 7.89 (dd, *J* = 8.9, 2.0 Hz, 1H), 7.60 – 7.46 (m, 3H).

^{13}C NMR (76 MHz, $CDCl_3$) δ 159.3, 149.3, 139.0, 137.5, 130.9, 130.0, 129.0, 127.7, 126.1, 125.5 (*d*, J = 4.4 Hz), 125.3 (*d*, *J* = 3.0 Hz), 124.1 (*d*, *J* = 272.2 Hz), 120.1.

The NMR data were in agreement with those reported in the literature.[8]

The synthesis of 1k:

The **1k** was synthesized through the reported coupling methods.[9]

^{1}H NMR (400 MHz, $CDCl_3$) δ 8.16 (s, 2H), 8.10 (d, *J* = 9.5 Hz, 1H), 8.06 (d, *J* = 8.9 Hz, 1H), 7.86 (d, *J* = 8.6 Hz, 1H), 7.61 (dd, *J* = 8.8, 2.2 Hz, 1H), 7.55 (s, 3H), 7.46 (d, *J* = 15.9 Hz, 1H), 2.61 (s, 3H).

^{13}C NMR (101 MHz, $CDCl_3$) δ 156.6, 146.6, 139.7, 137.2, 135.6, 130.1, 129.4, 129.3, 129.0, 127.8, 127.6, 122.5, 119.6, 15.9.

The NMR data were in agreement with those reported in the literature.[9]

The synthesis of 1m:

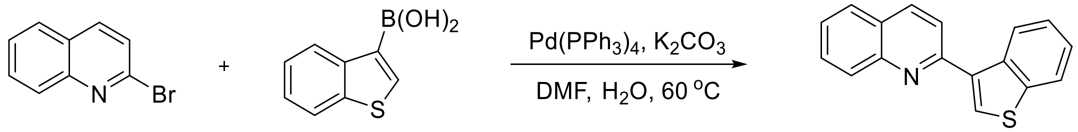

2-Bromoquinoline (0.21 g, 1.03 mmol), benzo[b]thien-3-ylboronic acid (0.37 g, 2.06 mmol), $Pd(PPh_3)_4$ (0.066 g, 0.057 mmol), and K_2CO_3 (0.29 g, 2.06 mmol) were dissolved in degassed DMF (1.4 mL) and degassed water (0.35 mL). The solution was stirred overnight at 60 °C under argon and a blue color was observed. The solution was extracted with dichloromethane and the solvent was removed under vacuum. The desired product was isolated by flash chromatography on silica gel (pentane/EtOAc 3/1).[10]

^{1}H NMR (300 MHz, $CDCl_3$) δ 8.87 (d, *J* = 7.9 Hz, 1H), 8.23 (dd, *J* = 10.7, 8.8 Hz, 2H), 7.94 (d, *J* = 7.0 Hz, 2H), 7.88 – 7.72 (m, 3H), 7.61 – 7.39 (m, 3H).

^{13}C NMR (76 MHz, $CDCl_3$) δ 154.5, 148.2, 141.0, 137.5, 136.7, 136.6, 129.8, 129.7, 127.60, 127.56, 127.0, 126.4, 125.0, 124.9, 124.8, 122.7, 120.8.

The NMR data were in agreement with those reported in the literature.[10]

The synthesis of 1w:

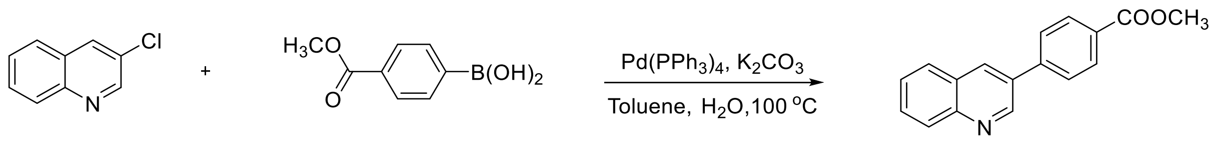

3-Chloroquinoline (0.32 g, 2.0 mmol), (4-(methoxycarbonyl)phenyl)boronic acid (0.54 g, 3.0 mmol), tetrakis-(triphenylphosphine)-palladium ($Pd(PPh_3)_4$) (0.11 g, 0.10 mmol), and K_2CO_3 (0.41 g, 3.0 mmol) were dissolved in degassed toluene (10 mL) and degassed water (3.0 mL). The solution was stirred overnight at 100 °C under argon and a blue color was observed. The solution was extracted with dichloromethane and the solvent was removed under vacuum. The desired product was isolated by flash chromatography on silica gel (pentane/EtOAc 5/1).

^{1}H NMR (300 MHz, $CDCl_3$) δ 9.19 (d, *J* = 2.4 Hz, 1H), 8.33 (d, *J* = 2.2 Hz, 1H), 8.26 – 8.10 (m, 3H), 7.88 (dd, *J* = 8.1, 1.5 Hz, 1H), 7.83 – 7.68 (m, 3H), 7.59 (ddd, *J* = 8.1, 6.9, 1.2 Hz, 1H), 3.97 (s, 3H).

^{13}C NMR (76 MHz, $CDCl_3$) δ 166.7, 149.5, 147.7, 142.2, 133.7, 132.6, 130.4, 129.9, 129.7, 129.3, 128.1, 127.8, 127.3, 127.2, 52.2.

The NMR data were in agreement with those reported in the literature.[7]

The synthesis of 1ab:

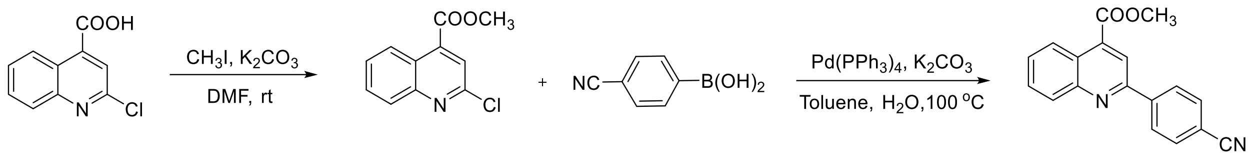

Procedure A: Potassium carbonate (2.77 g, 20.1 mmol) and methyl iodide (0.94 mL, 15.1 mmol) were added to a DMF (15 mL) solution of commercially available 2-chloro-4-quinolinecarboxylic acid (2.09 g, 10.5 mmol), and the mixture was stirred overnight at room temperature under an argon atmosphere. The reaction solution was added to a saturated aqueous solution of NaCl, and the formed crystals were collected by filtration, washed with water, and dried to obtain the methyl 2-chloroquinoline-4-carboxylate as a yellow solid.

Procedure B: Methyl 2-chloroquinoline-4-carboxylate (0.44 g, 2.0 mmol), (4-cyanophenyl)boronic acid (0.44 g, 3.0 mmol), tetrakis-(triphenylphosphine)-palladium ($Pd(PPh_3)_4$) (0.11 g, 0.10 mmol), and K_2CO_3 (0.41 g, 3.0 mmol) were dissolved in degassed toluene (10 mL) and degassed water (3.0 mL). The solution was stirred overnight at 100 °C under argon. The solution was extracted with dichloromethane and the solvent was removed under vacuum. The desired product was isolated by flash chromatography on silica gel (pentane/EtOAc 2/1).

^{1}H NMR (300 MHz, $CDCl_3$) δ 8.79 – 8.70 (m, 1H), 8.37 (s, 1H), 8.29 (d, *J* = 8.5 Hz, 2H), 8.20 (ddd, *J* = 8.5, 1.4, 0.7 Hz, 1H), 7.85 – 7.73 (m, 3H), 7.70 – 7.61 (m, 1H), 4.08 (s, 3H).

^{13}C NMR (76 MHz, $CDCl_3$) δ 166.4, 154.2, 149.2, 142.7, 136.0, 132.6, 130.5, 130.4, 128.7, 127.9, 125.5, 124.4, 119.9, 118.7, 113.1, 52.9.

The NMR data were in agreement with those reported in the literature.[11]

The synthesis of 1ad:

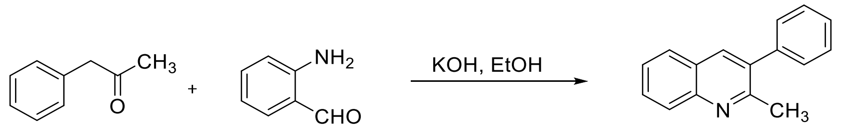

1-Phenylpropan-2-one (2.6 g, 19.3 mmol) and 2-aminobenzaldehyde (2.5 g, 20 mmol) were placed together with 10 mL aqueous KOH solution (33%) and 40 mL ethanol in a round bottom flask equipped with a condenser. The solution was heated to reflux for 3 h. After the solution has cooled down to room temperature concentrated acetic acid was added to consume remaining KOH and ethanol was removed under reduced pressure. The solution was extracted with dichloromethane and the solvent was removed under vacuum. The desired product was isolated by flash chromatography on silica gel (pentane/EtOAc 5/1).

^{1}H NMR (300 MHz, $CDCl_3$) δ 7.98 (dd, *J* = 8.5, 1.0 Hz, 1H), 7.86 (s, 1H), 7.68 (d, *J* = 8.7 Hz, 1H), 7.60 (ddd, *J* = 8.5, 6.9, 1.5 Hz, 1H), 7.44 – 7.26 (m, 6H), 2.58 (s, 3H).

^{13}C NMR (76 MHz, $CDCl_3$) δ 157.4, 147.1, 139.9, 136.1, 135.8, 129.4, 129.3, 128.5, 128.4, 127.6, 127.5, 126.9, 126.1, 24.6.

The NMR data were in agreement with those reported in the literature.[12]

The synthesis of 1ae:

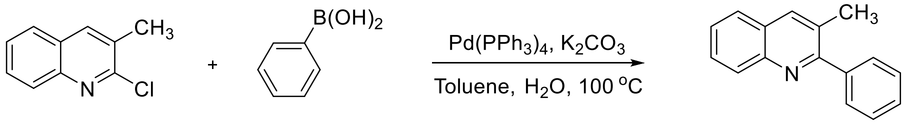

2-Chloro-3-methylquinoline (0.35 g, 2.0 mmol), phenylboronic acid (0.37 g, 3.0 mmol), tetrakis-(triphenylphosphine)-palladium ($Pd(PPh_3)_4$) (0.11 g, 0.10 mmol), and K_2CO_3 (0.41 g, 3.0 mmol) were dissolved in degassed toluene (10 mL) and degassed water (3.0 mL). The solution was stirred overnight at 100 °C under argon. The solution was extracted with dichloromethane and the solvent was removed under vacuum. The desired product was isolated by flash chromatography on silica gel (pentane/EtOAc 5/1).

^{1}H NMR (300 MHz, $CDCl_3$) δ 8.16 (d, J = 8.2 Hz, 1H), 8.01 (s, 1H), 7.78 (d, J = 8.1 Hz, 1H), 7.67 (ddd, J = 8.4, 6.9, 1.5 Hz, 1H), 7.63 – 7.57 (m, 2H), 7.56 – 7.40 (m, 4H), 2.46 (s, 3H).

^{13}C NMR (76 MHz, $CDCl_3$) δ 160.6, 146.7, 140.9, 136.8, 129.3, 129.3, 128.9, 128.8, 128.34, 128.27, 128.2, 127.6, 126.7, 126.5, 20.6.

The NMR data were in agreement with those reported in the literature.[13]

The synthesis of 1am:

The **1am** was synthesized through the reported coupling methods.[14]

^{1}H NMR (300 MHz, $CDCl_3$) δ 8.17 – 8.07 (m, 2H), 7.83 (dd, J = 8.2, 1.5 Hz, 1H), 7.67 (ddd, J = 8.5, 6.9, 1.5 Hz, 1H), 7.58 – 7.39 (m, 6H), 3.26 (p, J = 6.8 Hz, 1H), 1.26 (d, J = 6.8 Hz, 6H).

^{13}C NMR (76 MHz, $CDCl_3$) δ 160.5, 146.3, 141.1, 140.5, 132.9, 129.4, 129.0, 128.9, 128.4, 128.1, 127.9, 127.2, 126.4, 29.4, 24.3.

The NMR data were in agreement with those reported in the literature.[14]

The synthesis of 1an:

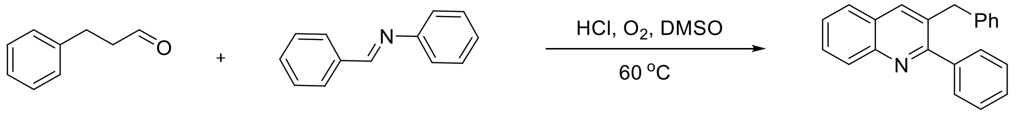

To a mixture of 3-phenylpropanal (80.4 mg, 0.60 mmol) and (*E*)-*N*,1-diphenylmethanimine (130.3 mg, 0.72 mmol) in dry DMSO (2.0 mL) was added HCl (4.0 M in dioxane) (5%), and the reaction mixture was stirred under an air atmosphere at 60 °C for 6 h. The reaction mixture was then poured into aqueous $NaHCO_3$ (2.0 M, 10 mL) and water (20 mL). The resulting solution was extracted with Et_2O (3 × 20 mL). The combined organic phases were dried over anhydrous Na_2SO_4, concentrated in vacuum, and the resulting residue was purified by column chromatography on silica gel (pentane/EtOAc 8/1) to afford the product.

^{1}H NMR (300 MHz, $CDCl_3$) δ 8.04 (dd, *J* = 8.5, 1.0 Hz, 1H), 7.79 (s, 1H), 7.61 (d, *J* = 8.0 Hz, 1H), 7.58 – 7.51 (m, 1H), 7.42 – 7.25 (m, 6H), 7.15 – 7.02 (m, 3H), 6.88 (d, *J* = 6.3 Hz, 2H), 4.01 (s, 2H).

^{13}C NMR (76 MHz, $CDCl_3$) δ 160.8, 146.7, 140.7, 140.0, 137.1, 132.6, 129.4, 129.2, 129.1, 128.9, 128.6, 128.4, 128.3, 127.6, 127.2, 126.5, 126.3, 39.2.

The NMR data were in agreement with those reported in the literature.[15]

The synthesis of 1ao:

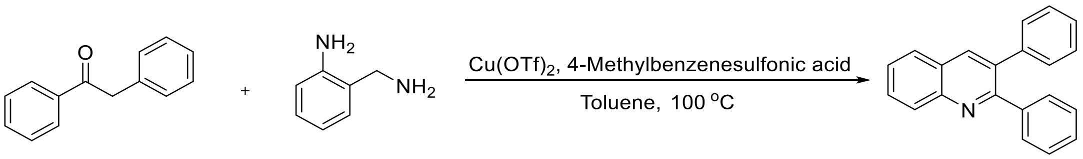

A mixture of 1,2-diphenylethan-1-one (0.59 g, 3.0 mmol), $Cu(OTf)_2$ (0.22 g, 0.60 mmol), 2-(aminomethyl)aniline (1.1 g, 9.0 mmol), and 4-methylbenzenesulfonic acid (0.02 g, 1.0 mmol) in toluene (10 mL) was stirred at 100 °C in a sealed Schlenk tube for 36 h. After the reaction was completed, the reaction mixture was cooled to room temperature. The resulting mixture was concentrated and extracted with dichloromethane (2 × 50 mL). The combined organic phases were dried over anhydrous Na_2SO_4, concentrated in vacuum, and the resulting residue was purified by column chromatography on silica gel (pentane/EtOAc 8/1) to afford the product.

^{1}H NMR (300 MHz, $CDCl_3$) δ 8.25 (d, *J* = 8.5 Hz, 1H), 8.21 (s, 1H), 7.90 (d, *J* = 7.3 Hz, 1H), 7.77 (ddd, *J* = 8.5, 6.9, 1.5 Hz, 1H), 7.60 (ddd, *J* = 8.1, 6.9, 1.3 Hz, 1H), 7.49 (dd, *J* = 6.6, 3.1 Hz, 2H), 7.35 – 7.26 (m, 8H).

^{13}C NMR (76 MHz, $CDCl_3$) δ 158.4, 147.3, 140.4, 140.0, 137.6, 134.6, 130.1, 129.8, 129.7, 129.5, 128.3, 128.1, 128.0, 127.5, 127.2, 126.8.

The NMR data were in agreement with those reported in the literature.[16]

The synthesis of 1ap:

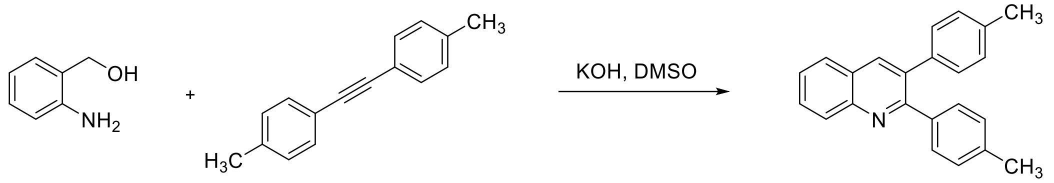

In an oven-dried round bottom flask, a solution of aminophenylmethanol (1.2 g, 10 mmol), 1,2-di-*p*-tolylethyne (1.6 g, 8.0 mmol) and 1.0 equiv of KOH in 20 mL of DMSO were added under inert atmosphere. The resulting reaction mixture was stirred at 90 °C for 24−30 h. The reaction was monitored by TLC analysis. After complete consumption of starting material, the reaction mixture was cooled to room temperature. The reaction mixture was then diluted with ethyl acetate (10 mL) and water (15 mL). The layers were separated, and the organic layer was washed with aqueous saturated brine solution and dried over Na_2SO_4. And the resulting residue was purified by column chromatography on silica gel (pentane/EtOAc 5/1) to afford the product.

^{1}H NMR (300 MHz, $CDCl_3$) δ 8.09 (dd, *J* = 8.5, 1.1 Hz, 1H), 8.00 (s, 1H), 7.71 (dd, *J* = 8.0, 1.5 Hz, 1H), 7.59 (ddd, *J* = 8.4, 6.9, 1.5 Hz, 1H), 7.40 (ddd, *J* = 8.1, 6.9, 1.2 Hz, 1H), 7.26 (d, *J* = 8.2 Hz, 2H), 7.07 – 6.94 (m, 6H), 2.24 (s, 3H), 2.22 (s, 3H).

^{13}C NMR (76 MHz, $CDCl_3$) δ 158.5, 147.3, 137.8, 137.7, 137.5, 137.3, 136.9, 134.5, 130.0, 129.6, 129.4, 129.4, 129.0, 128.7, 127.4, 127.2, 126.6, 21.4, 21.2.

The NMR data were in agreement with those reported in the literature.[12]

The synthesis of 1at:

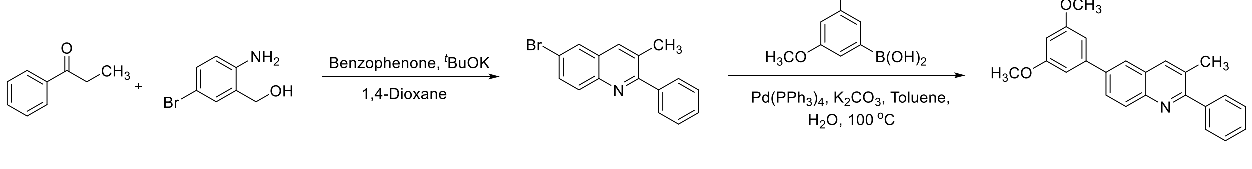

Procedure A: To a solution of (2-amino-5-bromophenyl)methanol (2.0 g, 10 mmol) in 1,4-dioxane (30 mL), the propiophenone (1.3 g, 10 mmol), benzophenone (1.8 g, 10 mmol), and potassium tert-butoxide (1.1 g, 10 mmol) were added under an argon atmosphere. The resulting mixture was stirred at 90 °C overnight.

Then, the reaction was cooled to room temperature and filtered through Celite. The resulting solution was evaporated in vacuo. The product was obtained after purification with column chromatography on silica gel (pentane/EtOAc 10/1).

Procedure B: 6-Bromo-3-methyl-2-phenylquinoline (0.60 g, 2.0 mmol), (3,5-dimethoxyphenyl)boronic acid (0.55 g, 3.0 mmol), tetrakis-(triphenylphosphine)-palladium ($Pd(PPh_3)_4$) (0.11 g, 0.10 mmol), and K_2CO_3 (0.41 g, 3.0 mmol) were dissolved in degassed toluene (10 mL) and degassed water (3.0 mL). The solution was stirred overnight at 100 °C under argon and a blue color was observed. The solution was extracted with dichloromethane and the solvent was removed under vacuum. The desired product was isolated by flash chromatography on silica gel (pentane/EtOAc 1/1).

^{1}H NMR (300 MHz, $CDCl_3$) δ 8.21 (d, *J* = 8.7 Hz, 1H), 8.10 (d, *J* = 1.3 Hz, 1H), 8.02 – 7.90 (m, 2H), 7.69 – 7.60 (m, 2H), 7.57 – 7.45 (m, 3H), 6.89 (d, *J* = 2.3 Hz, 2H), 6.55 (t, *J* = 2.2 Hz, 1H), 3.92 (s, 6H), 2.56 – 2.46 (m, 3H).

^{13}C NMR (76 MHz, $CDCl_3$) δ 161.2, 146.1, 142.8, 140.7, 139.1, 137.1, 129.8, 129.5, 128.9, 128.6, 128.4, 128.3, 127.7, 124.6, 105.7, 99.6, 94.3, 55.5, 20.7.

HRMS (ESI) m/z: $[M + Na]^+$ Calcd for $C_{24}H_{21}NO_2Na$ 378.1464; found 378.1464.

The general procedure for synthesis of quinoline hydrochlorides:

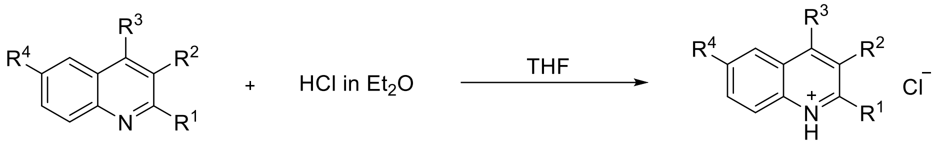

The quinoline (2.0 mmol) was dissolved in 10 mL THF. Then, 5.0 equivalents of (2.0 M HCl in Et_2O) were slowly added (about 10 minutes) to the solution at 0 °C (ice bath). After one hour, the white solid formed was filtered and washed with THF (10 mL) and Et_2O (10 mL), then dried under vacuum to afford the corresponding quinoline hydrochloride.

3. Reaction optimization

To an oven dried Schlenk tube with a magnetic stirring bar, **P** (0.8 mmol, 4.0 equiv.), **PC** (0.005 mmol, 2.5 mol%), deionized water (0.15 mL) and acetonitrile (4.0 mL) were added under argon atmosphere using standard Schlenk techniques at ambient temperature. Then, the substrate (0.2 mmol, 1.0 equiv.) was added under argon atmosphere. The tube was sealed and placed in the photoreactor, and then irradiated with a 10 W 445 nm LED at 20 °C using the standard set-up. After the specified time, the irradiation was stopped.

The volatiles were removed in vacuo, then the crude residue was analyzed via ^{1}H NMR using dibromo-methane as an internal standard.

The suggested mechanism for the formation of side product **2a'**: the radical rearrangement enabled by intramolecular radical addition of **Int1**, generated via HAT of **POH** to quinoline**,** resulted in the skatole radical **Int2** after the ring opening and rearomatization. The second electrophilic quinoline could continue to react with the nucleophilic C-radical of **Int2** giving C-H functionalization product **2a'**.

2-Phenyl-4-((2-phenyl-1H-indol-3-yl)methyl)quinoline **2a'**. **(Table S1, Entry 1)** Using the quinoline **1a** (0.2 mmol, 1.0 equiv.) and stirring at room temperature for 48 h under 10 W blue LED irradiation. The crude residue was purified by chromatography (pentane/EtOAc 6/1) to give **2a'** (25.4 mg, 0.064 mmol, 64%) as a colorless liquid.

^{1}H NMR (300 MHz, $CDCl_3$) δ 8.35 (s, 1H), 8.25 (d, *J* = 7.5 Hz, 1H), 8.20 (d, *J* = 8.0 Hz, 1H), 7.91 – 7.84 (m, 2H), 7.77 (ddd, *J* = 8.4, 6.9, 1.4 Hz, 1H), 7.62 – 7.54 (m, 2H), 7.51 – 7.45 (m, 3H), 7.43 – 7.33 (m, 7H), 7.25 (ddd, *J* = 8.1, 6.8, 1.2 Hz, 1H), 7.08 (ddd, *J* = 8.0, 7.0, 1.0 Hz, 1H), 4.75 (d, *J* = 1.2 Hz, 2H).

^{13}C NMR (76 MHz, $CDCl_3$) δ 157.6, 148.3, 147.4, 140.0, 136.3, 136.2, 132.6, 130.5, 129.4, 129.3, 129.1, 129.0, 128.6, 128.0, 127.6, 126.7, 126.2, 123.1, 122.7, 120.2, 119.3, 118.4, 111.0, 108.4, 27.4.

HRMS (ESI) m/z: $[M + Na]^+$ Calcd for $C_{30}H_{22}N_2Na$ 433.1675; found 433.1675.

Table S1. Reaction optimization and possible mechanism for the formation of side product **2a'**.

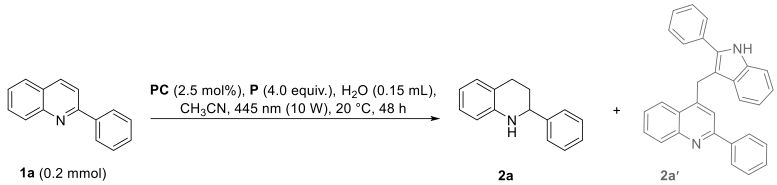

1a (0.2 mmol) **2a** **2a′**

Entry	Deviation from above	Yield (**2a**)[a]	Yield (**2a′**)[c]
1	—	10%[b]	64%
2	with 0.2 equiv. **ArSH**	20%[b]	48%
3	with 1.0 equiv. **ArSH**	28%[b]	41%
4	with **1a**-HCl as the substrate	88%	0
5	with **1a**-HCl as the substrate and 0.2 equiv. **ArSH**	87%	0
6	with **1a**-HCl as the substrate for 16 h	88% (86%)	0
7	without photocatalyst **PC**	< 5%	< 5%
8	without phosphine **P**	9%	< 5%

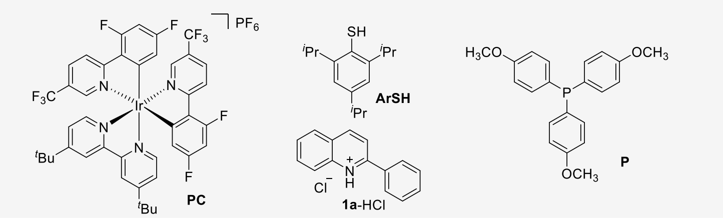

PC **ArSH** **1a**-HCl **P**

Commonly-used HAT catalysts tested:

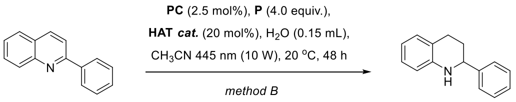

1a (0.2 mmol) **2a**

HAT *cat.*

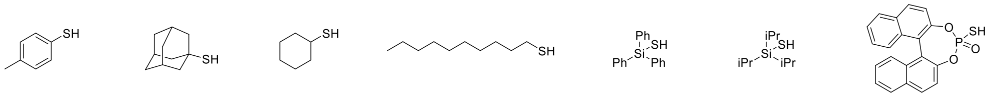

Yield of **2a**	< 5%	20%	8%	9%	34%	14%	0

Commonly-used photocatalysts tested:

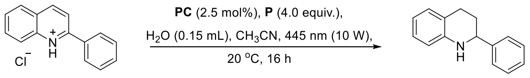

1a-HCl (0.2 mmol) **2a**

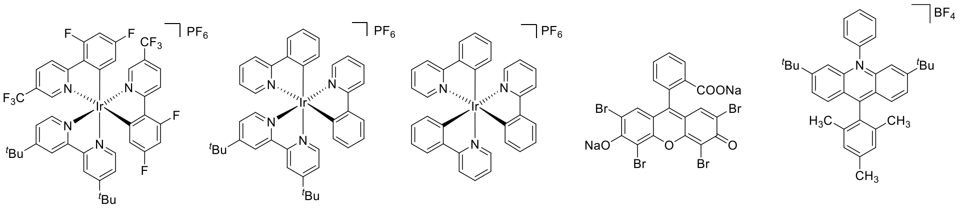

	**PC1**	**PC2**	**PC3**	**PC4**	**PC5**
Yield of **2a**:	86%	52%	45%	10%	12%

4. Synthesis and characterization of products

4.1 General procedures

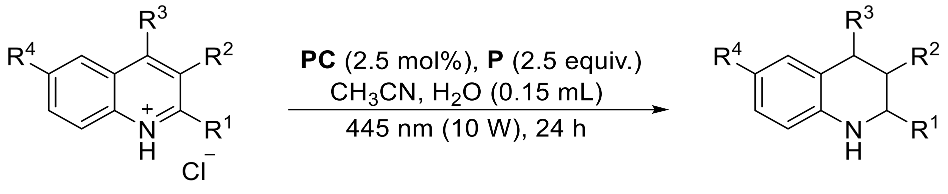

Method A**:** To an oven dried Schlenk tube with a magnetic stirring bar, **P** (0.5 mmol, 2.5 equiv.), **PC** (0.005 mmol, 2.5 mol%), deionized water (0.15 mL) and acetonitrile (4.0 mL) were added under argon atmosphere using standard Schlenk techniques at ambient temperature. Then, the quinoline HCl salt **1-HCl** (0.2 mmol, 1.0 equiv.) was added under argon atmosphere. The tube was sealed and placed in the photoreactor, and then irradiated with a 10 W 445 nm LED at 20 ^{o}C using the standard set-up. After 16 hours, the irradiation was stopped. The volatiles were removed in vacuo, then the crude residue was purified by flash column chromatography on silica (pentane, pentane/EtOAc or pentane/Et_2O mixtures, as detailed in the individual entries), to afford the corresponding product **2**.

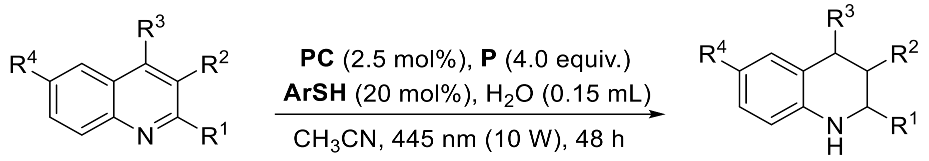

Method B**:** To an oven dried Schlenk tube with a magnetic stirring bar, **P** (0.8 mmol, 4.0 equiv.), **PC** (0.005 mmol, 2.5 mol%), deionized water (0.15 mL) and acetonitrile (4.0 mL) were added under argon atmosphere using standard Schlenk techniques at ambient temperature. Then, the quinoline **1** (0.2 mmol, 1.0 equiv.) and the HAT catalyst **ArSH** (0.04 mmol, 0.2 equiv.) were added under argon atmosphere. The tube was sealed and placed in the photoreactor, and then irradiated with a 10 W 445 nm LED at 20 ^{o}C using the standard set-up. After 48 hours, the irradiation was stopped. The volatiles were removed in vacuo, then the crude residue was purified by flash column chromatography on silica (pentane, pentane/EtOAc or pentane/Et_2O mixtures, as detailed in the individual entries), to afford the corresponding product **2**.

4.2 Synthesis and characterization of product 2 and 3

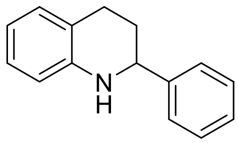

2a, 86%

2-Phenyl-1,2,3,4-tetrahydroquinoline **2a**. According to the **Method A**, using **1a-HCl** (0.2 mmol, 1.0 equiv.) and stirring at room temperature for 16 h under 10 W blue LED irradiation. The crude residue was purified by chromatography (pentane/EtOAc 8/1) to give **2a** (35.9 mg, 0.170 mmol, 86%) as a colorless liquid.

^{1}H NMR (300 MHz, $CDCl_3$) δ 7.43 – 7.27 (m, 5H), 7.01 (d, *J* = 7.3 Hz, 2H), 6.65 (t, *J* = 7.4 Hz, 1H), 6.55 (d, *J* = 8.2 Hz, 1H), 4.45 (dd, *J* = 9.2, 3.4 Hz, 1H), 4.04 (s, 1H), 2.93 (ddd, *J* = 16.2, 10.5, 5.5 Hz, 1H), 2.74 (dt, *J* = 16.3, 4.8 Hz, 1H), 2.13 (dddd, *J* = 13.3, 5.5, 4.5, 3.4 Hz, 1H), 2.00 (dddd, *J* = 13.0, 10.6, 9.3, 5.1 Hz, 1H).

^{13}C NMR (76 MHz, $CDCl_3$) δ 144.8, 144.7, 129.3, 128.6, 127.4, 126.9, 126.5, 120.9, 117.2, 114.0, 56.3, 31.0, 26.4.

The NMR data were in agreement with those reported in the literature.[17]

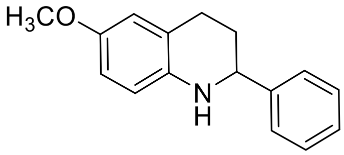

2c, 90%

6-Methyl-2-phenyl-1,2,3,4-tetrahydroquinoline **2c**. According to the **Method A**, using **1c-HCl** (0.2 mmol, 1.0 equiv.) and stirring at room temperature for 16 h under 10 W blue LED irradiation. The crude residue was purified by chromatography (pentane/EtOAc 5/1) to give **2c** (43.0 mg, 0.180 mmol, 90%) as a colorless liquid.

^{1}H NMR (300 MHz, $CDCl_3$) δ 7.43 – 7.32 (m, 4H), 7.32 – 7.27 (m, 1H), 6.69 – 6.59 (m, 2H), 6.51 (d, *J* = 8.3 Hz, 1H), 4.38 (dd, *J* = 9.5, 3.2 Hz, 1H), 3.75 (s, 3H), 2.95 (ddd, *J* = 16.5, 10.7, 5.8 Hz, 1H), 2.73 (dt, *J* = 16.6, 4.7 Hz, 1H), 2.12 (dddd, *J* = 13.1, 5.8, 4.1, 3.2 Hz, 1H), 2.00 (dddd, *J* = 14.8, 13.0, 7.4, 3.6 Hz, 1H).

^{13}C NMR (76 MHz, $CDCl_3$) δ 151.9, 144.9, 139.0, 128.6, 127.4, 126.6, 122.1, 115.1, 114.7, 113.1, 56.6, 55.9, 31.1, 26.8.

The NMR data were in agreement with those reported in the literature.[18]

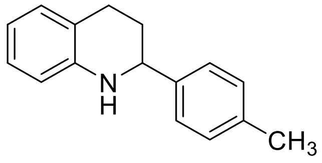

2d, 83%

2-(*p*-Tolyl)-1,2,3,4-tetrahydroquinoline **2d**. According to the **Method A**, using **1d-HCl** (0.2 mmol, 1.0 equiv.) and stirring at room temperature for 24 h under 10 W blue LED irradiation. The crude residue was purified by chromatography (pentane/EtOAc 8/1) to give **2d** (37.0 mg, 0.166 mmol, 83%) as a colorless liquid.

^{1}H NMR (300 MHz, $CDCl_3$) δ 7.35 – 7.28 (m, 2H), 7.20 (d, *J* = 7.8 Hz, 2H), 7.08 – 6.97 (m, 2H), 6.68 (t, *J* = 7.4 Hz, 1H), 6.56 (d, *J* = 8.2 Hz, 1H), 4.44 (dd, *J* = 9.4, 3.4 Hz, 1H), 4.03 (s, 1H), 2.96 (ddd, *J* = 16.3, 10.7, 5.6 Hz, 1H), 2.77 (dt, *J* = 16.4, 4.7 Hz, 1H), 2.39 (s, 3H), 2.14 (dddd, *J* = 13.2, 5.5, 4.3, 3.3 Hz, 1H), 2.01 (dddd, *J* = 12.9, 10.7, 9.3, 5.1 Hz, 1H).

^{13}C NMR (76 MHz, $CDCl_3$) δ 144.8, 141.8, 137.1, 129.3, 129.2, 126.9, 126.5, 120.9, 117.1, 114.0, 56.0, 31.0, 26.5, 21.1.

The NMR data were in agreement with those reported in the literature.[19]

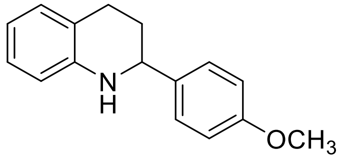

2e, 87%

2-(4-Methoxyphenyl)-1,2,3,4-tetrahydroquinoline **2e**. According to the **Method A**, using **1e-HCl** (0.2 mmol, 1.0 equiv.) and stirring at room temperature for 24 h under 10 W blue LED irradiation. The crude residue was purified by chromatography (pentane/EtOAc 5/1) to give **2e** (41.5 mg, 0.170 mmol, 87%) as a colorless liquid with inseparable 4,4'-dimethoxy-1,1'-biphenyl, which was generated from tris(4-methoxyphenyl)phosphane.

^{1}H NMR (300 MHz, $CDCl_3$) δ 7.23 (d, *J* = 8.4 Hz, 2H), 6.96 – 6.90 (m, 2H), 6.81 (d, *J* = 8.7 Hz, 2H), 6.57 (td, *J* = 7.4, 1.2 Hz, 1H), 6.45 (dd, *J* = 8.3, 1.2 Hz, 1H), 4.30 (dd, *J* = 9.5, 3.3 Hz, 1H), 3.94 (d, *J* = 16.2 Hz,

1H), 3.73 (s, 3H), 2.84 (td, *J* = 10.8, 5.4 Hz, 1H), 2.66 (dt, *J* = 16.4, 4.7 Hz, 1H), 2.00 (dddd, *J* = 9.9, 8.5, 4.5, 2.6 Hz, 1H), 1.95 – 1.79 (m, 1H).

The NMR data were in agreement with those reported in the literature.[20]

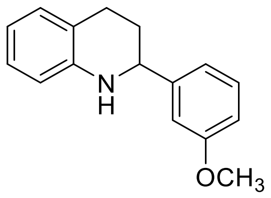

2f, 80%

2-(3-Methoxyphenyl)-1,2,3,4-tetrahydroquinoline **2f**. According to the **Method A**, using **1f-HCl** (0.2 mmol, 1.0 equiv.) and stirring at room temperature for 16 h under 10 W blue LED irradiation. The crude residue was purified by chromatography (pentane/EtOAc 8/1) to give **2f** (38.2 mg, 0.160 mmol, 80%) as a colorless liquid with inseparable 4,4'-dimethoxy-1,1'-biphenyl, which was generated from tris(4-methoxyphenyl)phosphane.

^{1}H NMR (300 MHz, $CDCl_3$) δ 7.2 (t, *J* = 8.0 Hz, 1H), 7.0 – 6.9 (m, 4H), 6.8 (ddd, *J* = 8.2, 2.4, 1.2 Hz, 1H), 6.6 (td, *J* = 7.4, 1.2 Hz, 1H), 6.5 (dd, *J* = 8.5, 1.3 Hz, 1H), 4.3 (dd, *J* = 9.4, 3.3 Hz, 1H), 4.0 (s, 1H), 3.7 (s, 3H), 2.9 (ddd, *J* = 16.3, 10.7, 5.5 Hz, 1H), 2.7 (dt, *J* = 16.4, 4.8 Hz, 1H), 2.1 – 2.0 (m, 1H), 2.0 – 1.8 (m, 1H).

The NMR data were in agreement with those reported in the literature.[21]

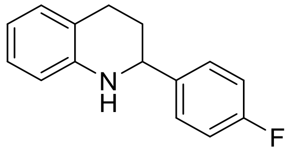

2g, 77%

2-(4-Fluorophenyl)-1,2,3,4-tetrahydroquinoline **2g**. According to the **Method A**, using **1g-HCl** (0.2 mmol, 1.0 equiv.) and stirring at room temperature for 16 h under 10 W blue LED irradiation. The crude residue was purified by chromatography (pentane/EtOAc 15/1) to give **2g** (34.9 mg, 0.154 mmol, 77%) as a colorless liquid.

^{1}H NMR (300 MHz, $CDCl_3$) δ 7.41 – 7.30 (m, 2H), 7.03 (td, *J* = 8.1, 5.1 Hz, 4H), 6.67 (td, *J* = 7.4, 1.2 Hz, 1H), 6.59 – 6.51 (m, 1H), 4.43 (dd, *J* = 9.3, 3.3 Hz, 1H), 3.98 (s, 1H), 2.90 (dt, *J* = 12.3, 6.0 Hz, 1H), 2.73 (dt, *J* = 16.4, 4.8 Hz, 1H), 2.10 (dddd, *J* = 13.3, 5.5, 4.5, 3.4 Hz, 1H), 1.96 (dddd, *J* = 12.9, 10.6, 9.3, 5.1 Hz, 1H).

^{19}F NMR (282 MHz, $CDCl_3$) δ -115.33.

The NMR data were in agreement with those reported in the literature.[22]

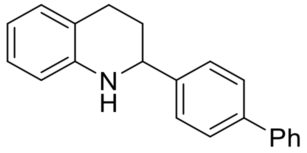

2h, 86%

2-([1,1'-Biphenyl]-4-yl)-1,2,3,4-tetrahydroquinoline **2h**. According to the **Method A**, using **1h-HCl** (0.2 mmol, 1.0 equiv.) and stirring at room temperature for 16 h under 10 W blue LED irradiation. The crude residue was purified by chromatography (pentane/EtOAc 15/1) to give **2h** (49.0 mg, 0.170 mmol, 86%) as a colorless liquid.

^{1}H NMR (300 MHz, $CDCl_3$) δ 7.65 – 7.55 (m, 4H), 7.50 – 7.40 (m, 4H), 7.40 – 7.30 (m, 1H), 7.02 (d, *J* = 7.2 Hz, 2H), 6.67 (t, *J* = 7.3 Hz, 1H), 6.57 (d, *J* = 7.7 Hz, 1H), 4.50 (dd, *J* = 9.2, 3.3 Hz, 1H), 4.08 (s, 1H), 2.95 (ddd, *J* = 16.2, 10.5, 5.5 Hz, 1H), 2.77 (dt, *J* = 16.4, 4.8 Hz, 1H), 2.17 (dq, *J* = 13.3, 4.5 Hz, 1H), 2.11 – 1.95 (m, 1H).

The NMR data were in agreement with those reported in the literature.[23]

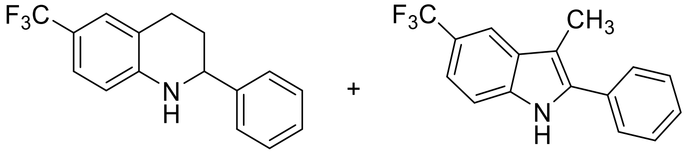

2i, 36% **2i'**, 30%

2-Phenyl-6-(trifluoromethyl)-1,2,3,4-tetrahydroquinoline **2i** and 3-methyl-2-phenyl-5-(trifluoromethyl)-1H-indole **2i'**. According to the **Method B**, using **1i** (0.2 mmol, 1.0 equiv.) and stirring at room temperature for 16 h under 10 W blue LED irradiation. The crude residue was purified by chromatography

(pentane/EtOAc 20/1) to give **2i** (19.9 mg, 0.072 mmol, 36%) and **2i'** (16.5 mg, 0.060 mmol, 30%) as colorless liquids.

^{1}H NMR (300 MHz, CDCl3, **2i**) δ 7.28 (d, *J* = 3.6 Hz, 4H), 7.18 – 7.13 (m, 2H), 6.46 (d, *J* = 8.9 Hz, 1H), 4.43 (dd, *J* = 8.8, 3.6 Hz, 1H), 4.30 (s, 1H), 2.88 – 2.76 (m, 1H), 2.67 (dt, *J* = 16.4, 5.1 Hz, 1H), 2.07 (ddt, *J* = 13.9, 5.3, 2.6 Hz, 1H), 2.00 – 1.82 (m, 1H).

^{19}F NMR (282 MHz, $CDCl_3$, **2i**) δ -60.86.

^{1}H NMR (300 MHz, CDCl3, **2i'**) δ 8.24 (s, 1H), 7.89 (s, 1H), 7.62 – 7.55 (m, 2H), 7.51 (ddd, *J* = 7.8, 6.9, 1.2 Hz, 2H), 7.42 (dd, *J* = 10.0, 1.6 Hz, 3H), 2.49 (s, 3H).

^{19}F NMR (282 MHz, $CDCl_3$, **2i'**) δ -60.19.

The NMR data were in agreement with those reported in the literature.[24]

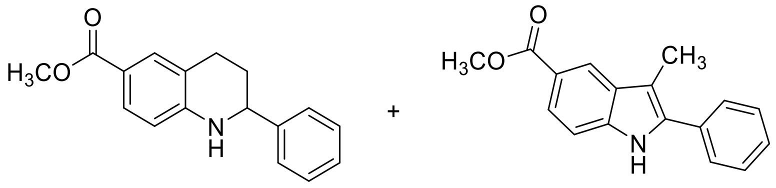

2j, 36% **2j'**, 24%

Methyl 2-phenyl-1,2,3,4-tetrahydroquinoline-6-carboxylate **2j** and methyl 3-methyl-2-phenyl-1H-indole-5-carboxylate **2j'**. According to the **Method B**, using **1j** (0.2 mmol, 1.0 equiv.) and stirring at room temperature for 16 h under 10 W blue LED irradiation. The crude residue was purified by chromatography (pentane/EtOAc 20/1) to give **2j** (16.0 mg, 0.060 mmol, 30%) and **2j'** (12.7 mg, 0.048 mmol, 24%) as colorless liquids.

^{1}H NMR (300 MHz, $CDCl_3$, **2j**) δ 7.75 – 7.66 (m, 2H), 7.35 (d, *J* = 3.1 Hz, 5H), 6.49 (d, *J* = 8.9 Hz, 1H), 4.51 (d, *J* = 3.2 Hz, 1H), 3.85 (s, 3H), 2.89 (ddd, *J* = 15.4, 10.0, 5.1 Hz, 1H), 2.74 (dt, *J* = 16.3, 5.0 Hz, 1H), 2.29 – 2.03 (m, 1H), 2.08 – 1.79 (m, 1H).

^{1}H NMR (500 MHz, $CDCl_3$, **2j'**) δ 8.38 (d, *J* = 0.7 Hz, 1H), 8.23 (s, 1H), 7.92 (dd, *J* = 8.5, 1.7 Hz, 1H), 7.60 – 7.56 (m, 2H), 7.53 – 7.47 (m, 2H), 7.42 – 7.35 (m, 2H), 3.95 (d, *J* = 0.4 Hz, 3H), 2.49 (s, 3H).

^{13}C NMR (126 MHz, $CDCl_3$, **2j'**) δ 168.2, 138.4, 135.2, 132.7, 129.7, 128.9, 127.73, 127.72, 123.72, 122.0, 121.6, 110.2, 110.0, 51.8, 9.6.

HRMS (ESI) m/z: $[M + Na]^+$ Calcd for $C_{17}H_{15}NO_2Na$ 288.0995; found 288.0997.

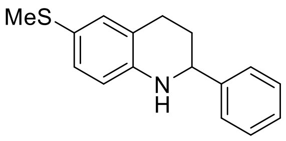

2k, 31%

6-(Methylthio)-2-phenyl-1,2,3,4-tetrahydroquinoline **2k**. According to the **Method A**, using **1k-HCl** (0.2 mmol, 1.0 equiv.) stirring at room temperature for 16 h under 10 W blue LED irradiation. The crude residue was purified by chromatography (pentane/EtOAc 8/1) to give **2k** (15.8 mg, 0.062 mmol, 31%) as a colorless liquid.

^{1}H NMR (599 MHz, $CDCl_3$) δ 7.37 (d, *J* = 30.5 Hz, 4H), 7.30 (d, *J* = 17.3 Hz, 1H), 7.06 (d, *J* = 9.9 Hz, 2H), 6.51 (d, *J* = 8.9 Hz, 1H), 4.44 (s, 1H), 2.89 (d, *J* = 32.3 Hz, 1H), 2.72 (d, *J* = 26.1 Hz, 1H), 2.43 (s, 3H), 2.13 (d, *J* = 26.5 Hz, 1H), 2.00 (d, *J* = 37.9 Hz, 1H).

^{13}C NMR (101 MHz, $CDCl_3$) δ 144.6, 143.8, 131.6, 129.4, 128.7, 127.7, 126.6, 124.0, 121.8, 114.7, 56.4, 30.8, 26.4, 19.4.

HRMS (ESI): m/z Calcd for $[M+Na]^+$ $C_{16}H_{17}NSNa$ 278.0974; found 278.0974.

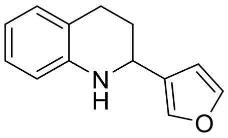

2l, 66%

2-(Furan-3-yl)-1,2,3,4-tetrahydroquinoline **2l**. According to the **Method B**, using **1l** (0.2 mmol, 1.0 equiv.) and stirring at room temperature for 48 h under 10 W blue LED irradiation. The crude residue was purified by chromatography (pentane/EtOAc 8/1) to give **2l** (26.3 mg, 0.130 mmol, 66%) as a colorless liquid.

^{1}H NMR (300 MHz, $CDCl_3$) δ 7.41 (d, *J* = 1.4 Hz, 2H), 7.04 – 6.96 (m, 2H), 6.66 (td, *J* = 7.4, 1.2 Hz, 1H), 6.53 (d, *J* = 8.8 Hz, 1H), 6.43 (s, 1H), 4.42 (dd, *J* = 9.2, 3.3 Hz, 1H), 3.96 (s, 1H), 2.92 (ddd, *J* = 16.2, 10.5, 5.6 Hz, 1H), 2.77 (dt, *J* = 16.4, 4.9 Hz, 1H), 2.12 (dddd, *J* = 13.2, 5.6, 4.5, 3.2 Hz, 1H), 1.98 (dddd, *J* = 13.0, 10.5, 9.2, 5.3 Hz, 1H).

^{13}C NMR (76 MHz, $CDCl_3$) δ 144.3, 143.3, 139.2, 129.4, 129.0, 126.9, 120.9, 117.4, 114.2, 108.9, 48.0, 29.8, 26.2.

HRMS (ESI) m/z: $[M + Na]^+$ Calcd for $C_{13}H_{13}NONa$ 222.0889; found 222.0889.

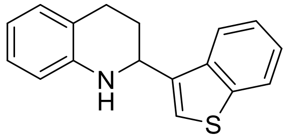

2m, 68%

2-(Benzo[b]thiophen-3-yl)-1,2,3,4-tetrahydroquinoline **2m**. According to the **Method B**, using the alkene **1m** (0.2 mmol, 1.0 equiv.) and stirring at room temperature for 48 h under 10 W blue LED irradiation. The crude residue was purified by chromatography (pentane/EtOAc 8/1) to give **2m** (36.4 mg, 0.140 mmol, 68%) as a colorless liquid.

^{1}H NMR (400 MHz, $CDCl_3$) δ 7.93 – 7.83 (m, 2H), 7.43 – 7.33 (m, 3H), 7.04 (t, J = 7.5 Hz, 2H), 6.69 (td, J = 7.4, 1.2 Hz, 1H), 6.59 (d, J = 7.3 Hz, 1H), 4.99 – 4.83 (m, 1H), 4.18 (s, 1H), 2.96 (ddd, J = 15.8, 10.0, 5.5 Hz, 1H), 2.77 (dt, J = 16.4, 5.1 Hz, 1H), 2.29 (dtd, J = 13.1, 5.4, 3.5 Hz, 1H), 2.18 (tdd, J = 10.0, 6.8, 4.4 Hz, 1H).

^{13}C NMR (101 MHz, $CDCl_3$) δ 144.4, 141.1, 139.4, 137.2, 129.4, 127.0, 124.5, 124.0, 123.1, 122.4, 121.9, 121.0, 117.5, 114.2, 51.2, 28.7, 26.1.

HRMS (ESI) m/z: $[M + Na]^+$ Calcd for $C_{17}H_{15}NSNa$ 288.0817; found 288.0817.

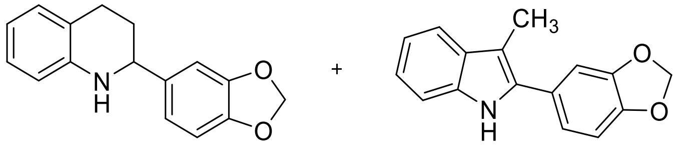

2n, 52% **2n'**, 22%

2-(Benzo[d][1,3]dioxol-5-yl)-1,2,3,4-tetrahydroquinoline **2n** and 2-(benzo[d][1,3]dioxol-5-yl)-3-methyl-1H-indole **2n'**. According to the **Method B**, using **1n** (0.2 mmol, 1.0 equiv.) and stirring at room temperature for 48 h under 10 W blue LED irradiation. The crude residue was purified by chromatography (pentane/EtOAc 8/1) to give **2n** (26.3 mg, 0.104 mmol, 52%) and **2n'** (11.0 mg, 0.044 mmol, 22%) as colorless liquids.

^{1}H NMR (300 MHz, $CDCl_3$, **2n**) δ 7.04 – 6.96 (m, 2H), 6.90 (d, J = 1.7 Hz, 1H), 6.84 (dd, J = 8.0, 1.7 Hz, 1H), 6.78 (d, J = 7.9 Hz, 1H), 6.65 (td, J = 7.4, 1.2 Hz, 1H), 6.53 (d, J = 7.5 Hz, 1H), 5.95 (s, 2H), 4.35 (dd, J = 9.4, 3.3 Hz, 1H), 3.98 (s, 1H), 2.92 (ddd, J = 16.3, 10.8, 5.5 Hz, 1H), 2.73 (dt, J = 16.4, 4.7 Hz, 1H), 2.14 – 2.03 (m, 1H), 1.94 (dddd, J = 13.0, 10.8, 9.4, 5.1 Hz, 1H).

^{13}C NMR (76 MHz, $CDCl_3$, **2n**) δ 147.8, 146.8, 144.7, 138.9, 129.3, 126.9, 120.9, 119.7, 117.2, 114.0, 108.2, 107.0, 101.0, 56.1, 31.2, 26.4.

The NMR data were in agreement with those reported in the literature.[25]

^{1}H NMR (300 MHz, $CDCl_3$, **2n'**) δ 7.95 (s, 1H), 7.63 – 7.58 (m, 1H), 7.41 – 7.34 (m, 1H), 7.26 – 7.13 (m, 2H), 7.10 – 7.03 (m, 2H), 6.95 (dd, J = 7.6, 0.9 Hz, 1H), 6.05 (s, 2H), 2.45 (s, 3H).

^{13}C NMR (151 MHz, $CDCl_3$, **2n'**) δ 148.0, 147.0, 135.6, 133.9, 130.0, 127.4, 122.1, 121.5, 119.5, 118.8, 110.5, 108.7, 108.3, 108.1, 101.2, 9.6.

HRMS (ESI) m/z: $[M + Na]^+$ Calcd for $C_{16}H_{13}NO_2Na$ 274.0838; found 274.0838.

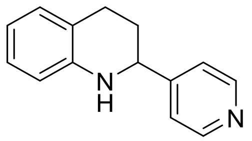

2o, 71%

2-(Pyridin-4-yl)-1,2,3,4-tetrahydroquinoline **2o**. According to the **Method B**, using **1o** (0.2 mmol, 1.0 equiv.) and stirring at room temperature for 48 h under 10 W blue LED irradiation. The crude residue was purified by chromatography (pentane/EtOAc 8/1) to give **2o** (29.8 mg, 0.140 mmol, 71%) as a colorless liquid.

^{1}H NMR (300 MHz, $CDCl_3$) δ 8.61 – 8.50 (m, 2H), 7.34 – 7.28 (m, 2H), 7.08 – 6.95 (m, 2H), 6.68 (td, J = 7.4, 1.2 Hz, 1H), 6.59 (dd, J = 7.9, 1.2 Hz, 1H), 4.47 (dd, J = 8.3, 3.6 Hz, 1H), 4.14 (s, 1H), 2.88 (ddd, J = 15.4, 9.4, 5.3 Hz, 1H), 2.67 (dt, J = 16.4, 5.5 Hz, 1H), 2.14 (dtd, J = 13.0, 5.6, 3.6 Hz, 1H), 1.97 (dddd, J = 13.2, 9.4, 8.3, 5.1 Hz, 1H).

^{13}C NMR (76 MHz, $CDCl_3$) δ 153.9, 150.1, 143.9, 129.4, 127.1, 121.6, 120.7, 117.7, 114.2, 55.0, 30.2, 25.5.

HRMS (ESI) m/z: $[M + Na]^+$ Calcd for $C_{14}H_{14}N_2Na$ 233.1049; found 233.1049.

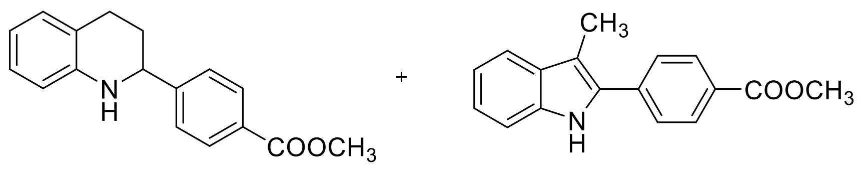

2p, 30% **2p′**, 51%

Methyl 4-(1,2,3,4-tetrahydroquinolin-2-yl)benzoate **2p**. According to the **Method B**, using **1p** (0.2 mmol, 1.0 equiv.) and stirring at room temperature for 48 h under 10 W blue LED irradiation. The crude residue was purified by chromatography (pentane/EtOAc 8/1) to give **2p** (16.0 mg, 0.060 mmol, 30%) and **2p'** (22.8 mg, 0.110 mmol, 56%) as colorless liquids (as reported by our recent work).[26]

^{1}H NMR (300 MHz, $CDCl_3$, **2p**) δ 7.94 (d, *J* = 8.3 Hz, 2H), 7.39 (d, *J* = 8.4 Hz, 2H), 7.03 – 6.86 (m, 2H), 6.60 (td, *J* = 7.4, 1.2 Hz, 1H), 6.50 (dd, *J* = 7.9, 1.2 Hz, 1H), 4.44 (dd, *J* = 8.9, 3.4 Hz, 1H), 4.01 (s, 1H), 3.84 (s, 3H), 2.84 (ddd, *J* = 15.9, 10.1, 5.4 Hz, 1H), 2.63 (dt, *J* = 16.4, 5.0 Hz, 1H), 2.16 – 2.00 (m, 1H), 1.98 – 1.83 (m, 1H).

^{13}C NMR (76 MHz, $CDCl_3$, **2p**) δ 167.0, 150.1, 144.3, 129.9, 129.35, 129.30, 127.0, 126.5, 120.8, 117.5, 114.1, 55.9, 52.1, 30.8, 26.0.

The NMR data were in agreement with those reported in the literature.[27]

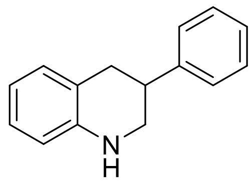

2q, 91% (A), 81% (B)

3-Phenyl-1,2,3,4-tetrahydroquinoline **2q**. According to the **Method A** or **Method B**, using **1q-HCl** (0.2 mmol, 1.0 equiv.) (**1q** for **Method B**) and stirring at room temperature for 16 h (48 h for **Method B**) under 10 W blue LED irradiation. The crude residue was purified by chromatography (pentane/EtOAc 8/1) to give **2q** (**A**: 38.0 mg, 0.180 mmol, 91%; **B**: 33.8 mg, 0.160 mmol, 81%) as a colorless liquid.

^{1}H NMR (400 MHz, $CDCl_3$) δ 7.26 (ddd, *J* = 8.8, 6.0, 1.3 Hz, 2H), 7.20 – 7.12 (m, 3H), 6.97 – 6.90 (m, 2H), 6.57 (td, *J* = 7.4, 1.2 Hz, 1H), 6.48 (d, *J* = 7.2 Hz, 1H), 3.88 (s, 1H), 3.38 (ddd, *J* = 11.1, 3.7, 1.9 Hz, 1H), 3.25 (t, *J* = 10.7 Hz, 1H), 3.07 (tdd, *J* = 10.1, 5.8, 3.7 Hz, 1H), 2.96 – 2.84 (m, 2H).

^{13}C NMR (101 MHz, $CDCl_3$) δ 143.91, 143.86, 129.6, 128.7, 127.3, 127.1, 127.0, 126.7, 121.5, 117.3, 114.2, 48.4, 38.7, 34.7.

The NMR data were in agreement with those reported in the literature.[28]

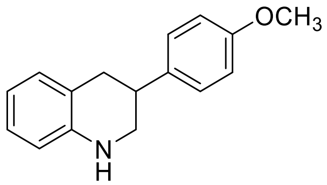

2r, 90% (A), 86% (B)

3-(4-Methoxyphenyl)-1,2,3,4-tetrahydroquinoline **2r**. According to the **Method A** or **Method B**, using **1r-HCl** (0.2 mmol, 1.0 equiv.) (**1r** for **Method B**) and stirring at room temperature for 16 h (48 h for **Method B**) under 10 W blue LED irradiation. The crude residue was purified by chromatography (pentane/EtOAc 8/1) to give **2r** (**A**: 43.0, 0.180 mmol, 90%; **B**: 41.1 mg, 0.170 mmol, 86%) as a colorless liquid.

^{1}H NMR (300 MHz, $CDCl_3$) δ 7.22 – 7.14 (m, 2H), 7.07 – 6.99 (m, 2H), 6.93 – 6.86 (m, 2H), 6.66 (td, *J* = 7.3, 1.2 Hz, 1H), 6.56 (dd, *J* = 8.4, 1.2 Hz, 1H), 3.82 (s, 3H), 3.45 (ddd, *J* = 11.2, 3.4, 1.4 Hz, 1H), 3.30 (t, *J* = 10.6 Hz, 1H), 3.18 – 3.05 (m, 1H), 3.03 – 2.92 (m, 2H).

^{13}C NMR (76 MHz, $CDCl_3$) δ 158.4, 144.1, 136.0, 129.6, 128.1, 127.0, 121.4, 117.1, 114.0, 55.3, 48.6, 37.8, 34.8.

The NMR data were in agreement with those reported in the literature.[22]

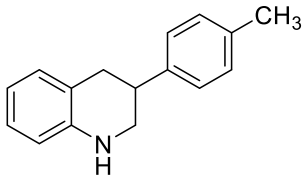

2s, 88% (A), 71% (B)

3-(*p*-Tolyl)-1,2,3,4-tetrahydroquinoline **2s**. According to the **Method A** or **Method B**, using **1s-HCl** (0.2 mmol, 1.0 equiv.) (**1s** for **Method B**) and stirring at room temperature for 16 h (48 h for **Method B**) under 10 W blue LED irradiation. The crude residue was purified by chromatography (pentane/EtOAc 8/1) to give **2s** (**A**: 39.2 mg, 0.176 mmol, 88%; **B**: 31.6 mg, 0.140 mmol, 71%) as a colorless liquid.

^{1}H NMR (300 MHz, $CDCl_3$) δ 7.15 (s, 4H), 7.06 – 6.95 (m, 2H), 6.65 (td, *J* = 7.4, 1.2 Hz, 1H), 6.55 (dd, *J* = 8.4, 1.2 Hz, 1H), 3.50 – 3.41 (m, 1H), 3.39 – 3.26 (m, 1H), 3.19 – 3.05 (m, 1H), 3.03 – 2.94 (m, 2H), 2.35 (s, 3H).

^{13}C NMR (76 MHz, $CDCl_3$) δ 144.0, 140.8, 136.2, 129.5, 129.3, 127.1, 127.0, 121.5, 117.1, 114.1, 48.5, 38.3, 34.7, 21.0.

The NMR data were in agreement with those reported in the literature.[22]

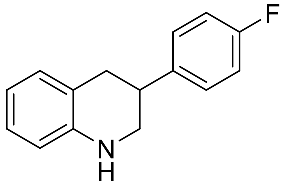

2t, 88% (A), 80% (B)

3-(4-Fluorophenyl)-1,2,3,4-tetrahydroquinoline **2t**. According to the **Method A** or **Method B**, using **1t-HCl** (0.2 mmol, 1.0 equiv.) (**1t** for **Method B**) and stirring at room temperature for 16 h (48 h for **Method B**) under 10 W blue LED irradiation. The crude residue was purified by chromatography (pentane/EtOAc 8/1) to give **2t** (**A**: 40.0 mg, 0.176 mmol, 88%; **B**: 36.3 mg, 0.160 mmol, 80%) as a colorless liquid.

^{1}H NMR (300 MHz, $CDCl_3$) δ 7.16 – 7.06 (m, 2H), 6.99 – 6.87 (m, 4H), 6.57 (td, J = 7.4, 1.2 Hz, 1H), 6.47 (d, J = 8.5 Hz, 1H), 3.41 – 3.30 (m, 1H), 3.21 (t, J = 10.5 Hz, 1H), 3.13 – 2.99 (m, 1H), 2.89 (d, J = 7.9 Hz, 2H).

^{19}F NMR (282 MHz, $CDCl_3$) δ -116.49.

^{13}C NMR (76 MHz, $CDCl_3$) δ 161.7 (d, J = 244.6 Hz), 144.0, 140.0 (d, J = 3.2 Hz), 129.5, 128.6 (d, J = 7.7 Hz), 127.1, 121.1, 117.2, 115.4 (d, J = 21.1 Hz), 114.1, 48.4, 38.0, 34.7.

The NMR data were in agreement with those reported in the literature.[29]

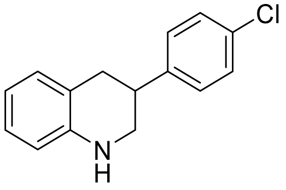

2u, 86% (A), 76% (B)

3-(4-Chlorophenyl)-1,2,3,4-tetrahydroquinoline **2u**. According to the **Method A** or **Method B**, using **1u-HCl** (0.2 mmol, 1.0 equiv.) (**1u** for **Method B**) and stirring at room temperature for 16 h (48 h for **Method**

B) under 10 W blue LED irradiation. The crude residue was purified by chromatography (pentane/EtOAc 10/1) to give **2u** (**A**: 41.8 mg, 0.170 mmol, 86%; **B**: 36.9 mg, 0.150 mmol, 76%) as a colorless liquid.

^{1}H NMR (300 MHz, $CDCl_3$) δ 7.35 – 7.28 (m, 2H), 7.22 – 7.12 (m, 2H), 7.07 – 6.94 (m, 2H), 6.66 (td, *J* = 7.4, 1.2 Hz, 1H), 6.56 (d, *J* = 7.8 Hz, 1H), 4.17 – 3.87 (m, 1H), 3.45 (ddt, *J* = 11.1, 3.7, 1.1 Hz, 1H), 3.37 – 3.26 (m, 1H), 3.20 – 3.07 (m, 1H), 2.98 (d, *J* = 7.8 Hz, 2H).

^{13}C NMR (76 MHz, $CDCl_3$) δ 143.9, 142.3, 132.4, 129.5, 128.7, 128.6, 127.1, 120.9, 117.3, 114.1, 48.2, 38.1, 34.5.

The NMR data were in agreement with those reported in the literature.[22]

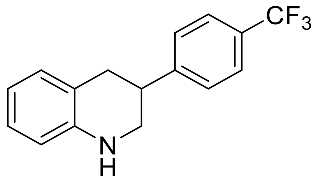

2v, 80% (A), 74% (B)

3-(4-(Trifluoromethyl)phenyl)-1,2,3,4-tetrahydroquinoline **2v**. According to the **Method A** or **Method B**, using **1v-HCl** (0.2 mmol, 1.0 equiv.) (**1v** for **Method B**) and stirring at room temperature for 16 h (48 h for **Method B**) under 10 W blue LED irradiation. The crude residue was purified by chromatography (pentane/EtOAc 8/1) to give **2v** (**A**: 44.3 mg, 0.160 mmol, 80%; **B**: 41.0 mg, 0.150 mmol, 74%) as a colorless liquid.

^{1}H NMR (400 MHz, $CDCl_3$) δ 7.63 – 7.56 (m, 2H), 7.36 (d, *J* = 8.1 Hz, 2H), 7.08 – 7.00 (m, 2H), 6.67 (td, *J* = 7.4, 1.2 Hz, 1H), 6.57 (dd, *J* = 7.9, 1.1 Hz, 1H), 4.05 (s, 1H), 3.52 – 3.45 (m, 1H), 3.42 – 3.32 (m, 1H), 3.31 – 3.17 (m, 1H), 3.02 (d, *J* = 8.3 Hz, 2H).

^{19}F NMR (376 MHz, $CDCl_3$) δ -62.42.

The NMR data were in agreement with those reported in the literature.[29]

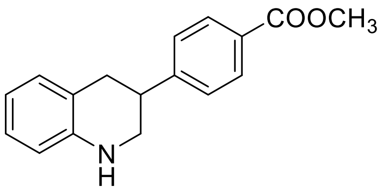

2w, 71%

Methyl 4-(1,2,3,4-tetrahydroquinolin-3-yl)benzoate **2w**. According to the **Method B**, using **1w** (0.2 mmol, 1.0 equiv.) and stirring at room temperature for 48 h under 10 W blue LED irradiation. The crude residue was purified by chromatography (pentane/EtOAc 6/1) to give **2w** (37.9 mg, 0.140 mmol, 71%) as a colorless liquid.

^{1}H NMR (300 MHz, $CDCl_3$) δ 8.01 (d, *J* = 8.4 Hz, 2H), 7.32 (d, *J* = 8.3 Hz, 2H), 7.08 – 6.98 (m, 2H), 6.66 (td, *J* = 7.3, 1.2 Hz, 1H), 6.56 (d, *J* = 7.5 Hz, 1H), 3.92 (s, 3H), 3.52 – 3.43 (m, 1H), 3.36 (t, *J* = 10.5 Hz, 1H), 3.22 (dtd, *J* = 9.8, 6.6, 3.3 Hz, 1H), 3.06 – 2.97 (m, 2H).

^{13}C NMR (76 MHz, $CDCl_3$) δ 167.0, 149.2, 143.9, 129.9, 129.5, 128.6, 127.3, 127.1, 120.8, 117.3, 114.2, 52.1, 48.0, 38.8, 34.4.

HRMS (ESI) m/z: $[M + Na]^+$ Calcd for $C_{17}H_{17}NO_2Na$ 290.1151; found 290.1152.

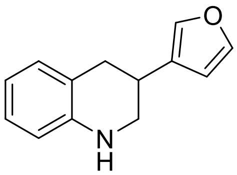

2x, 41%

3-(Furan-3-yl)-1,2,3,4-tetrahydroquinoline **2x**. According to the **Method B**, using **1x** (0.2 mmol, 1.0 equiv.) and stirring at room temperature for 48 h under 10 W blue LED irradiation. The crude residue was purified by chromatography (pentane/EtOAc 6/1) to give **2x** (16.3 mg, 0.080 mmol, 41%) as a colorless liquid with a small amount of inseparable unidentified side product.

^{1}H NMR (300 MHz, $CDCl_3$) δ 7.39 (t, *J* = 1.7 Hz, 1H), 7.30 (d, *J* = 0.8 Hz, 1H), 6.99 (d, *J* = 7.4 Hz, 2H), 6.63 (dd, *J* = 7.5, 1.2 Hz, 1H), 6.57 – 6.48 (m, 1H), 6.35 (dd, *J* = 1.9, 0.9 Hz, 1H), 4.20 – 3.74 (m, 1H), 3.50 (ddd, *J* = 10.9, 3.4, 2.0 Hz, 1H), 3.33 – 3.20 (m, 1H), 3.19 – 2.97 (m, 2H), 2.93 – 2.81 (m, 1H).

^{13}C NMR (76 MHz, $CDCl_3$) δ 144.1, 142.9, 138.5, 129.6, 127.3, 127.0, 120.3, 117.3, 114.1, 109.7, 47.7, 34.0, 29.6.

HRMS (ESI) m/z: [M + Na]$^+$ Calcd for $C_{13}H_{13}NONa$ 222.0889; found 222.0890.

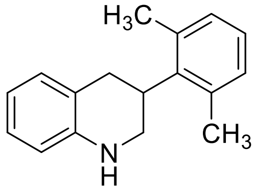

2y, 74%

3-(2,6-Dimethylphenyl)-1,2,3,4-tetrahydroquinoline **2y**. According to the **Method A**, using **1y-HCl** (0.2 mmol, 1.0 equiv.) and stirring at room temperature for 24 h under 10 W blue LED irradiation. The crude residue was purified by chromatography (pentane/EtOAc 8/1) to give **2y** (35.1 mg, 0.150 mmol, 74%) as a colorless liquid.

^{1}H NMR (300 MHz, $CDCl_3$) δ 7.17 – 6.92 (m, 5H), 6.70 (td, *J* = 7.4, 1.2 Hz, 1H), 6.61 (dd, *J* = 7.9, 1.2 Hz, 1H), 3.86 – 3.77 (m, 1H), 3.71 (tdd, *J* = 11.8, 5.0, 3.3 Hz, 1H), 3.45 – 3.27 (m, 2H), 2.85 (ddd, *J* = 16.3, 5.2, 1.9 Hz, 1H), 2.44 (s, 6H).

^{13}C NMR (76 MHz, $CDCl_3$) δ 144.6, 139.2, 136.8, 129.7, 126.9, 126.4, 121.8, 117.4, 114.6, 44.7, 36.6, 30.9, 21.7.

HRMS (ESI) m/z: [M + Na]$^+$ Calcd for $C_{17}H_{19}NNa$ 260.1410; found 260.1410.

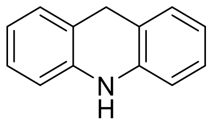

2z, 81%

9,10-Dihydroacridine **2z**. According to the **Method B**, using **1z** (0.2 mmol, 1.0 equiv.) and stirring at room temperature for 48 h under 10 W blue LED irradiation. The crude residue was purified by chromatography (pentane/EtOAc 20/1) to give **2z** (29.3 mg, 0.160 mmol, 81%) as a colorless liquid.

^{1}H NMR (300 MHz, $CDCl_3$) δ 7.19 – 7.06 (m, 4H), 6.88 (td, *J* = 7.4, 1.2 Hz, 2H), 6.68 (dd, *J* = 7.9, 1.2 Hz, 2H), 5.95 (s, 1H), 4.08 (s, 2H).

^{13}C NMR (76 MHz, $CDCl_3$) δ 140.2, 128.6, 127.0, 120.7, 120.1, 113.5, 31.4.

The NMR data were in agreement with those reported in the literature.[30]

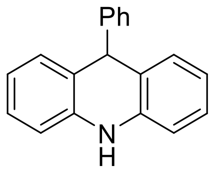

2aa, 84%

9-Phenyl-9,10-dihydroacridine **2aa**. According to the **Method B**, using **1aa** (0.2 mmol, 1.0 equiv.) and stirring at room temperature for 48 h under 10 W blue LED irradiation. The crude residue was purified by chromatography (pentane/EtOAc 20/1) to give **2aa** (43.2 mg, 0.170 mmol, 84%) as a colorless liquid.

^{1}H NMR (300 MHz, $CDCl_3$) δ 7.20 – 7.09 (m, 4H), 7.08 – 6.93 (m, 5H), 6.75 (t, *J* = 7.3 Hz, 2H), 6.66 (d, *J* = 7.9 Hz, 2H), 6.03 (s, 1H), 5.21 (s, 1H).

^{13}C NMR (76 MHz, $CDCl_3$) δ 147.1, 138.9, 129.6, 128.6, 127.9, 127.3, 126.3, 123.6, 120.9, 113.8, 47.5.

The NMR data were in agreement with those reported in the literature.[31]

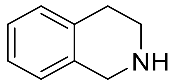

3a, 67%

1,2,3,4-Tetrahydroisoquinoline **3a**. According to the **Method B**, using the isoquinoline (0.2 mmol, 1.0 equiv.) and stirring at room temperature for 48 h under 10 W blue LED irradiation. The crude residue was purified by chromatography (EtOAc) to give **3a** (17.8 mg, 0.170 mmol, 67%) as a colorless liquid.

^{1}H NMR (300 MHz, $CDCl_3$) δ 7.22 – 7.07 (m, 3H), 7.07 – 6.97 (m, 1H), 4.04 (s, 2H), 3.16 (t, *J* = 6.0 Hz, 2H), 2.82 (t, *J* = 6.0 Hz, 2H).

^{13}C NMR (76 MHz, $CDCl_3$) δ 136.1, 134.9, 129.3, 126.2, 126.0, 125.7, 48.4, 44.0, 29.3.

The NMR data were in agreement with those reported in the literature.[32]

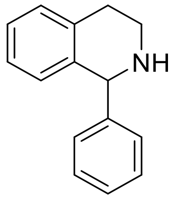

3b, 57%

1-Phenyl-1,2,3,4-tetrahydroisoquinoline **3b**. According to the **Method B**, using the 1-phenylisoquinoline (0.2 mmol, 1.0 equiv.) and stirring at room temperature for 48 h under 10 W blue LED irradiation. The crude residue was purified by chromatography (EtOAc) to give **3b** (23.0 mg, 0.110 mmol, 57%) as a colorless liquid.

^{1}H NMR (300 MHz, $CDCl_3$) δ 7.34 (hd, *J* = 6.7, 4.3 Hz, 5H), 7.20 (d, *J* = 3.7 Hz, 2H), 7.15 – 7.00 (m, 1H), 6.81 (d, *J* = 7.6 Hz, 1H), 5.15 (s, 1H), 3.41 – 3.26 (m, 1H), 3.21 – 3.01 (m, 2H), 2.96 – 2.74 (m, 1H), 1.96 (s, 1H).

^{13}C NMR (76 MHz, $CDCl_3$) δ 145.0, 138.4, 135.5, 129.1, 129.0, 128.5, 128.1, 127.4, 126.3, 125.7, 62.2, 42.3, 29.9.

The NMR data were in agreement with those reported in the literature.[33]

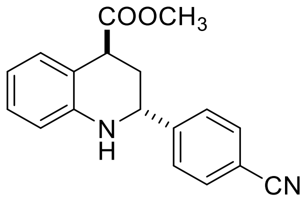

2ab, 86%

Methyl 2-(4-cyanophenyl)-1,2,3,4-tetrahydroquinoline-4-carboxylate **2ab**. According to the **Method B**, using **1ab** (0.2 mmol, 1.0 equiv.) and stirring at room temperature for 48 h under 10 W blue LED irradiation. The crude residue was purified by chromatography (pentane/EtOAc 6/1) to give **2ab** (50.2 mg, 0.170 mmol, 86%) as a colorless liquid (a 1.3:1 trans:cis mixture of diastereoisomers).

^{1}H NMR (300 MHz, $CDCl_3$, *trans*-**2ab**) δ 7.60 – 7.54 (m, 2H), 7.52 – 7.45 (m, 2H), 7.19 – 7.13 (m, 1H), 7.06 – 6.93 (m, 1H), 6.64 (dd, *J* = 7.4, 1.2 Hz, 1H), 6.57 – 6.52 (m, 1H), 4.66 (dd, *J* = 10.9, 3.0 Hz, 1H), 3.71 (dd, *J* = 5.7, 2.7 Hz, 1H), 3.67 (s, 3H), 2.35 – 2.24 (m, 1H), 1.84 (ddd, *J* = 13.4, 10.9, 5.7 Hz, 1H).

^{13}**C NMR** (76 MHz, $CDCl_3$, both diastereoisomers) δ 174.3, 174.1, 149.4, 148.7, 144.1, 132.6, 132.6, 130.6, 128.5, 128.5, 128.3, 127.6, 127.5, 118.8, 118.5, 118.0, 117.7, 116.4, 115.2, 115.1, 111.7, 111.5, 55.3, 52.8, 52.3, 52.2, 43.6, 41.2, 34.8, 33.1.

HRMS (ESI) m/z: $[M + Na]^+$ Calcd for $C_{18}H_{16}N_2O_2Na$ 315.1104; found 315.1104.

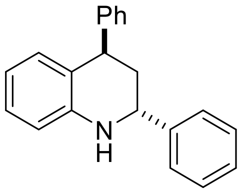

2ac, 86%, *trans:cis* 2:1

2,4-Diphenyl-1,2,3,4-tetrahydroquinoline **2ac**. According to the **Method A**, using the alkene **1ac-HCl** (0.2 mmol, 1.0 equiv.) and stirring at room temperature for 16 h under 10 W blue LED irradiation. The crude residue was purified by chromatography (pentane/EtOAc 15/1) to give **2ac** (49.0 mg, 0.170 mmol, 86%) as a colorless liquid (a 2:1 trans:cis mixture of diastereoisomers).

^{1}**H NMR** (300 MHz, $CDCl_3$, *trans*-**2ac**) δ 7.26 – 7.22 (m, 4H), 7.20 – 7.14 (m, 4H), 7.06 – 7.00 (m, 2H), 6.80 (ddd, J = 7.7, 1.6, 0.7 Hz, 1H), 6.59 – 6.53 (m, 2H), 6.52 – 6.48 (m, 1H), 4.24 (dd, J = 9.8, 3.5 Hz, 1H), 4.07 (t, J = 4.7 Hz, 1H), 2.22 – 2.11 (m, 2H).

^{13}**C NMR** (76 MHz, $CDCl_3$, both diastereoisomers) δ 146.9, 144.8, 144.4, 130.6, 129.7, 128.73, 128.70, 128.68, 128.6, 128.3, 127.8, 127.6, 127.5, 127.3, 126.69, 126.67, 126.5, 126.1, 122.1, 117.7, 117.3, 114.4, 114.1, 57.3, 52.0, 45.0, 42.1, 41.7, 39.1.

The NMR data were in agreement with those reported in the literature.[18]

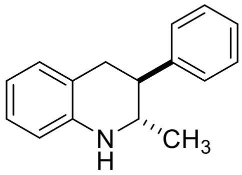

2ad, 83%, *trans:cis* 1:3

2-Methyl-3-phenyl-1,2,3,4-tetrahydroquinoline **2ad**. According to the **Method A**, using **1ad-HCl** (0.2 mmol, 1.0 equiv.) and stirring at room temperature for 16 h under 10 W blue LED irradiation. The crude

residue was purified by chromatography (pentane/EtOAc 15/1) to give **2ad** (37.0 mg, 0.166 mmol, 83%) as a colorless liquid (a 1:3 trans:cis mixture of diastereoisomers).

^{1}H NMR (500 MHz, $CDCl_3$, *cis*-**2ad**) δ 7.34 – 7.29 (m, 2H), 7.25 – 7.20 (m, 3H), 7.10 – 7.05 (m, 2H), 6.73 (td, *J* = 7.4, 1.2 Hz, 1H), 6.60 (dt, *J* = 7.8, 1.6 Hz, 1H), 3.75 (qd, *J* = 6.5, 3.5 Hz, 1H), 3.31 – 3.25 (m, 1H), 3.15 (dd, *J* = 16.1, 6.5 Hz, 2H), 1.01 (d, *J* = 6.5 Hz, 3H).

^{13}C NMR (126 MHz, $CDCl_3$, both diastereoisomers) δ 144.2, 144.0, 143.8, 142.1, 129.6, 129.1, 128.7, 128.5, 128.2, 127.8, 127.0, 126.9, 126.6, 126.4, 121.7, 120.6, 117.5, 116.9, 114.4, 113.6, 52.4, 50.3, 46.3, 42.2, 36.0, 30.3, 20.9, 18.1.

The NMR data were in agreement with those reported in the literature.[14]

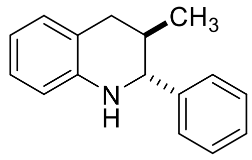

2ae, 88%, *trans:cis* 5:1

3-Methyl-2-phenyl-1,2,3,4-tetrahydroquinoline **2ae**. According to the **Method A**, using the alkene **1ae-HCl** (0.2 mmol, 1.0 equiv.) and stirring at room temperature for 16 h under 10 W blue LED irradiation. The crude residue was purified by chromatography (pentane/EtOAc 15/1) to give **2ae** (39.2 mg, 0.176 mmol, 88%) as a colorless liquid (a 5:1 trans:cis mixture of diastereoisomers).

^{1}H NMR (300 MHz, $CDCl_3$, *trans*-**2ae**) δ 7.31 – 7.20 (m, 5H), 6.92 (ddq, *J* = 7.7, 3.9, 1.4 Hz, 2H), 6.56 (td, *J* = 7.4, 1.2 Hz, 1H), 6.42 (dd, *J* = 8.2, 1.2 Hz, 1H), 3.88 (d, *J* = 8.9 Hz, 1H), 2.73 (dd, *J* = 16.3, 4.9 Hz, 1H), 2.53 (dd, *J* = 16.3, 10.6 Hz, 1H), 1.99 (dddd, *J* = 13.1, 8.4, 6.6, 3.3 Hz, 1H), 0.76 (d, *J* = 6.6 Hz, 3H).

^{13}C NMR (76 MHz, $CDCl_3$, both diastereoisomers) δ 144.5, 144.2, 143.5, 143.0, 129.7, 129.1, 128.5, 128.1, 127.7, 127.6, 127.2, 127.1, 126.90, 126.85, 120.9, 120.1, 117.1, 117.0, 113.7, 113.3, 63.4, 59.4, 35.4, 33.8, 33.4, 31.9, 18.5, 15.2.

The NMR data were in agreement with those reported in the literature.[18]

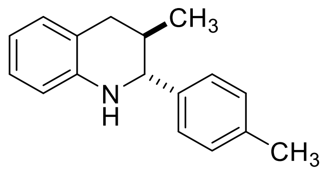

2af, 70%, *trans:cis* 10:1

3-Methyl-2-(*p*-tolyl)-1,2,3,4-tetrahydroquinoline **2af**. According to the **Method A**, using the alkene **1af-HCl** (0.2 mmol, 1.0 equiv.) and stirring at room temperature for 16 h under 10 W blue LED irradiation. The crude residue was purified by chromatography (pentane/EtOAc 15/1) to give **2af** (33.2 mg, 0.140 mmol, 70%) as a colorless liquid (a 10:1 trans:cis mixture of diastereoisomers).

^{1}H NMR (300 MHz, $CDCl_3$, *trans*-**2af**) δ 7.20 – 7.13 (m, 2H), 7.07 (d, *J* = 8.1 Hz, 2H), 6.95 – 6.86 (m, 2H), 6.55 (td, *J* = 7.4, 1.2 Hz, 1H), 6.40 (dd, *J* = 8.3, 1.2 Hz, 1H), 3.83 (d, *J* = 9.0 Hz, 1H), 2.72 (dd, *J* = 16.3, 4.8 Hz, 1H), 2.52 (dd, *J* = 16.2, 10.7 Hz, 1H), 2.27 (s, 3H), 2.03 – 1.88 (m, 1H), 0.74 (d, *J* = 6.6 Hz, 3H).

^{13}C NMR (76 MHz, $CDCl_3$, *trans*-**2af**) δ 144.6, 140.5, 137.3, 129.2, 129.1, 127.5, 126.8, 121.0, 116.9, 113.3, 63.1, 35.5, 33.8, 21.2, 18.6.

The NMR data were in agreement with those reported in the literature.[34]

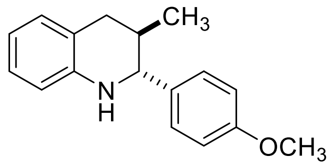

2ag, 70%, *trans:cis* > 20:1

2-(4-Methoxyphenyl)-3-methyl-1,2,3,4-tetrahydroquinoline **2ag**. According to the **Method A**, using the alkene **1ag-HCl** (0.2 mmol, 1.0 equiv.) and stirring at room temperature for 16 h under 10 W blue LED irradiation. The crude residue was purified by chromatography (pentane/EtOAc 15/1) to give **2ag** (35.4 mg, 0.140 mmol, 70%) as a colorless liquid.

^{1}H NMR (300 MHz, $CDCl_3$, *trans*-**2ag**) δ 7.29 (d, *J* = 8.6 Hz, 2H), 7.00 (ddd, *J* = 7.1, 3.8, 2.3 Hz, 2H), 6.94 – 6.84 (m, 2H), 6.64 (td, *J* = 7.3, 1.2 Hz, 1H), 6.49 (dd, *J* = 8.3, 1.2 Hz, 1H), 3.91 (d, *J* = 10.1 Hz, 1H), 3.83 (s, 3H), 2.82 (dd, *J* = 16.3, 4.9 Hz, 1H), 2.62 (dd, *J* = 16.3, 10.8 Hz, 1H), 2.09 – 1.98 (m, 1H), 0.83 (d, *J* = 6.6 Hz, 3H).

^{13}**C NMR** (76 MHz, $CDCl_3$, *trans*-**2ag**) δ 159.1, 144.6, 135.5, 129.1, 128.6, 126.8, 121.0, 116.9, 113.9, 113.3, 62.8, 55.3, 35.6, 33.9, 18.5.

The NMR data were in agreement with those reported in the literature.[34]

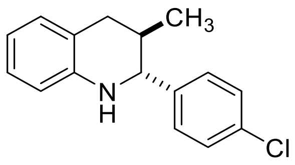

2ah, 69%, *trans:cis* > 20:1

2-(4-Chlorophenyl)-3-methyl-1,2,3,4-tetrahydroquinoline **2ah**. According to the **Method A**, using the alkene **1ah-HCl** (0.2 mmol, 1.0 equiv.) and stirring at room temperature for 16 h under 10 W blue LED irradiation. The crude residue was purified by chromatography (pentane/EtOAc 15/1) to give **2ah** (35.4 mg, 0.140 mmol, 69%) as a colorless liquid with inseparable 4,4'-dimethoxy-1,1'-biphenyl, which was generated from tris(4-methoxyphenyl)phosphane.

^{1}**H NMR** (300 MHz, $CDCl_3$, *trans*-**2ah**) δ 7.40 – 7.30 (m, 4H), 7.07 – 7.00 (m, 2H), 6.68 (td, *J* = 7.4, 1.2 Hz, 1H), 6.57 – 6.49 (m, 1H), 3.97 (d, *J* = 8.9 Hz, 1H), 2.82 (dd, *J* = 16.3, 4.8 Hz, 1H), 2.69 – 2.56 (m, 1H), 2.12 – 1.96 (m, 1H), 0.85 (d, *J* = 6.6 Hz, 3H).

^{13}**C NMR** (101 MHz, $CDCl_3$, *trans*-**2ah**) δ 144.2, 142.0, 129.2, 128.9, 128.6, 126.9, 120.8, 117.3, 113.4, 62.7, 35.2, 33.9, 18.4.

HRMS (ESI) m/z: $[M + Na]^+$ Calcd for $C_{16}H_{16}ClNNa$ 280.0863; found 280.0863.

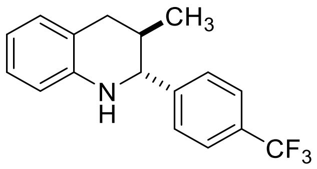

2ai, 62%, *trans:cis* 3.3:1

3-Methyl-2-(4-(trifluoromethyl)phenyl)-1,2,3,4-tetrahydroquinoline **2ai**. According to the **Method A**, using the alkene **1ai-HCl** (0.2 mmol, 1.0 equiv.) and stirring at room temperature for 16 h under 10 W blue LED irradiation. The crude residue was purified by chromatography (pentane/EtOAc 15/1) to give **2ai** (36.1 mg, 0.124 mmol, 62%) as a colorless liquid (a 3.3:1 trans:cis mixture of diastereoisomers).

^{1}H NMR (300 MHz, $CDCl_3$, *trans*-**2ai**) δ 7.52 (t, *J* = 8.8 Hz, 2H), 7.40 (d, *J* = 8.1 Hz, 2H), 6.97 – 6.90 (m, 2H), 6.59 (td, *J* = 7.4, 1.2 Hz, 1H), 6.45 (d, *J* = 7.4 Hz, 1H), 3.96 (d, *J* = 8.6 Hz, 1H), 2.73 (dd, *J* = 16.3, 4.9 Hz, 1H), 2.53 (dd, *J* = 16.3, 10.3 Hz, 1H), 2.06 – 1.95 (m, 1H), 0.77 (d, *J* = 6.6 Hz, 3H).

^{19}F NMR (282 MHz, $CDCl_3$) δ -62.39 (*cis*-**2ai**), -62.44 (*trans*-**2ai**).

The NMR data were in agreement with those reported in the literature.[34]

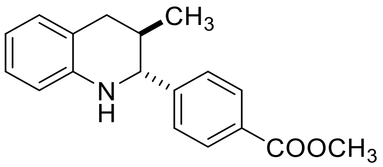

2aj, 64%, *trans:cis* 10:1

Methyl 4-(3-methyl-1,2,3,4-tetrahydroquinolin-2-yl)benzoate **2aj**. According to the **Method B**, using the alkene **1aj** (0.2 mmol, 1.0 equiv.) and stirring at room temperature for 48 h under 10 W blue LED irradiation. The crude residue was purified by chromatography (pentane/EtOAc 15/1) to give **2aj** (36.0 mg, 0.130 mmol, 64%) as a colorless liquid (a 10:1 trans:cis mixture of diastereoisomers).

^{1}H NMR (400 MHz, $CDCl_3$, *trans*-**2aj**) δ 8.02 (d, *J* = 8.4 Hz, 2H), 7.44 (d, *J* = 8.3 Hz, 2H), 7.06 – 6.97 (m, 2H), 6.67 (td, *J* = 7.4, 1.2 Hz, 1H), 6.53 (dd, *J* = 7.9, 1.2 Hz, 1H), 4.04 (d, *J* = 8.7 Hz, 1H), 3.92 (s, 3H), 2.80 (dd, *J* = 16.3, 4.9 Hz, 1H), 2.61 (dd, *J* = 16.3, 10.4 Hz, 1H), 2.08 (dddd, *J* = 10.4, 8.7, 6.7, 4.9 Hz, 1H), 0.84 (d, *J* = 6.6 Hz, 3H).

^{13}C NMR (101 MHz, $CDCl_3$, *trans*-**2aj**) δ 166.9, 148.9, 144.1, 129.8, 129.6, 129.2, 127.6, 127.0, 120.7, 117.3, 113.5, 63.0, 52.1, 35.0, 33.9, 18.5.

HRMS (ESI) m/z: $[M + Na]^+$ Calcd for $C_{18}H_{19}NO_2Na$ 304.1308; found 304.1308.

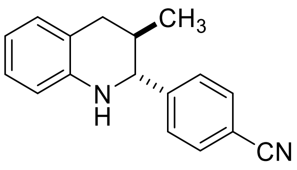

2ak, 60%, *trans:cis* > 20:1

4-(3-Methyl-1,2,3,4-tetrahydroquinolin-2-yl)benzonitrile **2ak**. According to the **Method B**, using the alkene **1ak** (0.2 mmol, 1.0 equiv.) and stirring at room temperature for 48 h under 10 W blue LED irradiation. The crude residue was purified by chromatography (pentane/EtOAc 8/1) to give **2ak** (29.8 mg, 0.120 mmol, 60%) as a colorless liquid.

^{1}H NMR (300 MHz, $CDCl_3$, *trans*-**2ak**) δ 7.64 (d, *J* = 8.3 Hz, 2H), 7.48 (d, *J* = 8.3 Hz, 2H), 7.07 – 6.96 (m, 2H), 6.68 (td, *J* = 7.4, 1.2 Hz, 1H), 6.58 – 6.50 (m, 1H), 4.05 (d, *J* = 8.4 Hz, 1H), 2.78 (dd, *J* = 16.3, 5.0 Hz, 1H), 2.60 (dd, *J* = 16.3, 9.9 Hz, 1H), 2.12 – 2.05 (m, 1H), 0.85 (d, *J* = 6.6 Hz, 3H).

^{13}C NMR (76 MHz, $CDCl_3$, *trans*-**2ak**) δ 147.2, 141.7, 130.3, 127.3, 126.3, 125.0, 118.6, 116.8, 115.7, 111.6, 109.5, 60.8, 32.6, 31.8, 16.4.

HRMS (ESI) m/z: $[M + Na]^+$ Calcd for $C_{17}H_{16}N_2Na$ 271.1206; found 271.1206.

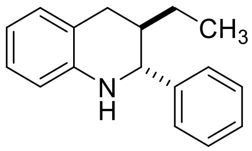

2al, 70%, *trans:cis* 3:1

3-Ethyl-2-phenyl-1,2,3,4-tetrahydroquinoline **2al**. According to the **Method A**, using the alkene **1al-HCl** (0.2 mmol, 1.0 equiv.) and stirring at room temperature for 16 h under 10 W blue LED irradiation. The crude residue was purified by chromatography (pentane/EtOAc 15/1) to give **2al** (33.2 mg, 0.140 mmol, 70%) as a colorless liquid (a 3:1 trans:cis mixture of diastereoisomers).

^{1}H NMR (300 MHz, $CDCl_3$, *trans*-**2al**) δ 7.27 (d, *J* = 4.6 Hz, 3H), 7.21 – 7.17 (m, 2H), 6.97 – 6.91 (m, 2H), 6.61 – 6.56 (m, 1H), 6.43 (dd, *J* = 8.3, 1.2 Hz, 1H), 4.01 (d, *J* = 8.1 Hz, 1H), 2.79 (dd, *J* = 16.2, 4.6 Hz, 1H), 2.47 (dd, *J* = 16.2, 9.6 Hz, 1H), 1.81 (dq, *J* = 12.9, 4.5 Hz, 1H), 1.29 – 1.18 (m, 1H), 1.08 – 0.94 (m, 1H), 0.83 – 0.77 (m, 3H).

^{13}C NMR (76 MHz, $CDCl_3$, both diastereoisomers) δ 144.4, 144.0, 143.1, 129.7, 129.4, 128.5, 128.1, 127.5, 127.5, 127.3, 127.1, 127.0, 126.9, 120.6, 120.2, 116.9, 113.6, 113.2, 61.5, 58.9, 40.1, 38.9, 31.3, 29.5, 25.3, 22.5, 12.0, 11.2.

The NMR data were in agreement with those reported in the literature.[34]

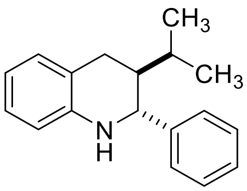

2am, 72%, *trans:cis* 1:2.2

3-Isopropyl-2-phenyl-1,2,3,4-tetrahydroquinoline **2am**. According to the **Method A**, using the alkene **1am-HCl** (0.2 mmol, 1.0 equiv.), the phosphine P(p-CF_3-C_6H_4)$_3$ (0.5 mmol, 2.5 equiv.), a reduced amount of deionized water (0.025 mL), additional **ArSH** (20 mol%) and stirring at room temperature for 48 h under 30 W blue Kessil-lamp (456 nm) irradiation. The crude residue was purified by chromatography (pentane/EtOAc 25/1) to give *trans*-**2am** (11.5 mg, 0.046 mmol, 23%) and *cis*-**2am** (24.5 mg, 0.098 mmol, 49%) as two colorless liquids respectively.

trans-**2am**:

^{1}H NMR (300 MHz, $CDCl_3$) δ 7.40 – 7.27 (m, 5H), 7.04 – 6.96 (m, 2H), 6.64 (td, *J* = 7.4, 1.2 Hz, 1H), 6.50 (dd, *J* = 7.5, 1.5 Hz, 1H), 4.30 (d, *J* = 8.1 Hz, 1H), 2.77 – 2.60 (m, 2H), 1.90 (tt, *J* = 8.6, 5.2 Hz, 1H), 1.54 (td, *J* = 6.9, 4.8 Hz, 1H), 0.96 (d, *J* = 6.9 Hz, 3H), 0.85 (d, *J* = 6.8 Hz, 3H).

^{13}C NMR (76 MHz, $CDCl_3$) δ 144.5, 144.3, 129.5, 128.6, 127.6, 127.5, 126.9, 121.1, 117.0, 113.2, 59.2, 44.1, 27.1, 26.1, 21.6, 16.9.

HRMS (ESI): m/z Calcd for $[M+H]^+$ $C_{18}H_{21}NH^+$ 252.1747; found 252.1747.

cis-**2am**:

^{1}H NMR (300 MHz, $CDCl_3$) δ 7.25 (d, *J* = 27.1 Hz, 0H), 7.03 (d, *J* = 14.2 Hz, 1H), 6.66 (td, *J* = 7.4, 1.2 Hz, 1H), 6.52 (dd, *J* = 8.5, 1.3 Hz, 1H), 4.64 (dd, *J* = 4.2, 1.3 Hz, 1H), 4.39 (s, 1H), 2.83 (tt, *J* = 16.8, 16.2, 4.9, 3.9 Hz, 1H), 2.52 (tt, *J* = 16.4, 12.2 Hz, 1H), 1.94 (d, *J* = 30.1 Hz, 0H), 1.27 (d, *J* = 28.4 Hz, 0H), 1.11 (d, *J* = 6.4 Hz, 3H), 0.85 (d, *J* = 6.6 Hz, 3H).

^{13}C NMR (76 MHz, $CDCl_3$) δ 144.2, 143.9, 129.7, 128.3, 127.6, 127.2, 120.9, 116.8, 113.4, 56.9, 43.6, 28.4, 26.9, 22.3, 20.4.

HRMS (ESI): m/z Calcd for $[M+H]^+$ $C_{18}H_{21}NH^+$ 252.1747; found 252.1748.

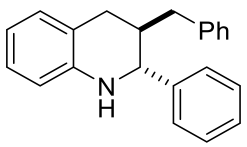

2an, 70%, *trans:cis* 10:1

3-Benzyl-2-phenyl-1,2,3,4-tetrahydroquinoline **2an**. According to the **Method A**, using the alkene **1an-HCl** (0.2 mmol, 1.0 equiv.) and stirring at room temperature for 16 h under 10 W blue LED irradiation. The crude residue was purified by chromatography (pentane/EtOAc 15/1) to give **2an** (41.9 mg, 0.140 mmol, 70%) as a colorless liquid (as a 10:1 trans:cis mixture of diastereoisomers).

^{1}H NMR (400 MHz, $CDCl_3$, *trans*-**2an**) δ 7.35 (d, *J* = 4.5 Hz, 4H), 7.30 – 7.24 (m, 3H), 7.21 – 7.17 (m, 1H), 7.13 – 7.10 (m, 2H), 7.02 (td, *J* = 7.5, 1.5 Hz, 1H), 6.91 (dd, *J* = 7.5, 1.4 Hz, 1H), 6.62 (td, *J* = 7.4, 1.2 Hz, 1H), 6.55 (dd, *J* = 7.9, 1.2 Hz, 1H), 4.19 (d, *J* = 6.6 Hz, 1H), 4.11 (s, 1H), 2.69 – 2.58 (m, 2H), 2.50 (dd, *J* = 16.3, 7.4 Hz, 1H), 2.38 – 2.28 (m, 2H).

^{13}C NMR (101 MHz, $CDCl_3$, *trans*-**2an**) δ 143.2, 143.0, 139.2, 128.5, 128.0, 127.6, 127.2, 126.5, 126.1, 125.9, 124.9, 118.9, 115.9, 112.1, 59.5, 39.2, 38.1, 29.6.

HRMS (ESI) m/z: $[M + Na]^+$ Calcd for $C_{22}H_{21}NNa$ 322.1566; found 322.1566.

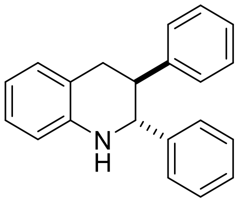

2ao, 78%, *trans:cis* > 20:1

2,3-Diphenyl-1,2,3,4-tetrahydroquinoline **2ao**. According to the **Method A**, using the alkene **1ao-HCl** (0.2 mmol, 1.0 equiv.) and stirring at room temperature for 16 h under 10 W blue LED irradiation. The crude residue was purified by chromatography (pentane/EtOAc 15/1) to give **2ao** (44.5 mg, 0.156 mmol, 78%) as a colorless liquid.

^{1}H NMR (300 MHz, $CDCl_3$, ***trans*-2ao**) δ 7.32 – 7.22 (m, 4H), 7.19 – 7.14 (m, 3H), 7.13 – 7.07 (m, 3H), 7.06 – 7.02 (m, 2H), 6.72 (td, *J* = 7.4, 1.2 Hz, 1H), 6.64 – 6.59 (m, 1H), 4.50 (d, *J* = 9.0 Hz, 1H), 4.23 (s, 1H), 3.34 – 3.22 (m, 1H), 3.21 – 3.12 (m, 1H), 3.04 (dd, *J* = 15.1, 3.9 Hz, 1H).

^{13}C NMR (76 MHz, $CDCl_3$, ***trans*-2ao**) δ 144.3, 142.7, 140.5, 129.1, 128.24, 128.19, 128.0, 127.51, 127.48, 127.1, 126.4, 121.2, 117.0, 113.4, 62.6, 46.4, 35.0.

The NMR data were in agreement with those reported in the literature.[35]

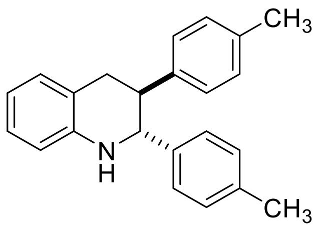

2ap, 83%, *trans:cis* 3:1

2,3-Di-p-tolyl-1,2,3,4-tetrahydroquinoline **2ap**. According to the **Method A**, using the alkene **1ap-HCl** (0.2 mmol, 1.0 equiv.) and stirring at room temperature for 16 h under 10 W blue LED irradiation. The crude residue was purified by chromatography (pentane/EtOAc 15/1) to give **2ap** (52.0 mg, 0.166 mmol, 83%) as a colorless liquid (as a 3:1 trans:cis mixture of diastereoisomers).

When extending reaction time to 36 h, **2ap** was given in 72% yield with a 10:1 trans:cis diastereoselectivity.

^{1}H NMR (300 MHz, $CDCl_3$, ***trans*-2ap**) **^{1}H NMR** (300 MHz, $CDCl_3$) δ 6.98 (td, J = 5.1, 2.0 Hz, 4H), 6.93 – 6.87 (m, 4H), 6.84 – 6.79 (m, 2H), 6.61 – 6.57 (m, 1H), 6.47 (dd, J = 8.4, 1.2 Hz, 1H), 4.34 (d, J = 8.8 Hz, 1H), 4.04 (s, 1H), 3.09 – 2.86 (m, 3H), 2.18 (s, 3H), 2.17 (s, 3H).

^{13}C NMR (76 MHz, $CDCl_3$, both diastereoisomers) δ 144.5, 144.3, 139.9, 139.8, 138.8, 138.3, 137.0, 136.6, 135.9, 135.7, 129.5, 129.03, 128.98, 128.9, 128.7, 128.5, 128.3, 127.8, 127.6, 127.4, 127.13, 127.06, 121.3, 120.8, 117.1, 116.9, 113.7, 113.3, 62.1, 60.1, 45.7, 43.0, 35.3, 29.8, 21.13, 21.10, 21.05.

HRMS (ESI) m/z: $[M + Na]^+$ Calcd for $C_{23}H_{23}NNa$ 336.1723; found 336.1721.

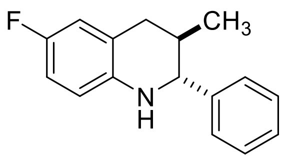

2aq, 60%, *trans:cis* > 20:1

6-Fluoro-3-methyl-2-phenyl-1,2,3,4-tetrahydroquinoline **2aq**. According to the **Method A**, using the alkene **1aq-HCl** (0.2 mmol, 1.0 equiv.) and stirring at room temperature for 16 h under 10 W blue LED irradiation. The crude residue was purified by chromatography (pentane/EtOAc 15/1) to give **2aq** (28.9 mg, 0.120 mmol, 60%) as a colorless liquid.

^{1}H NMR (599 MHz, $CDCl_3$, ***trans*-2aq**) δ 7.35 (d, *J* = 4.3 Hz, 4H), 7.32 – 7.28 (m, 1H), 6.72 (d, *J* = 8.8 Hz, 2H), 6.43 (dd, *J* = 8.2, 4.8 Hz, 1H), 3.92 (d, *J* = 8.9 Hz, 1H), 2.78 (dd, *J* = 16.5, 4.9 Hz, 1H), 2.59 (dd, *J* = 16.5, 10.7 Hz, 1H), 2.11 – 2.01 (m, 1H), 0.83 (d, *J* = 6.6 Hz, 3H).

^{19}F NMR (282 MHz, $CDCl_3$, ***trans*-2aq**) δ -128.32.

^{13}C NMR (151 MHz, $CDCl_3$, ***trans*-2aq**) δ 155.5 (d, *J* = 234.6 Hz), 143.2, 140.8, 128.5, 127.7, 127.5, 122.17 (d, *J* = 6.7 Hz), 115.2 (d, *J* = 21.7 Hz), 113.9 (d, *J* = 7.6 Hz), 113.3 (d, *J* = 22.4 Hz), 63.4, 35.4, 33.6, 18.4.

HRMS (ESI) m/z: $[M + Na]^+$ Calcd for $C_{16}H_{16}FNNa$ 264.1159; found 264.1159.

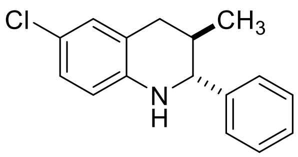

2ar, 78%, *trans:cis* 10:1

6-Chloro-3-methyl-2-phenyl-1,2,3,4-tetrahydroquinoline **2ar**. According to the **Method A**, using the alkene **1ar-HCl** (0.2 mmol, 1.0 equiv.) and stirring at room temperature for 16 h under 10 W blue LED irradiation. The crude residue was purified by chromatography (pentane/EtOAc 15/1) to give **2ar** (40.0 mg, 0.156 mmol, 78%) as a colorless liquid (as a 10:1 trans:cis mixture of diastereoisomers).

^{1}H NMR (599 MHz, $CDCl_3$ ***trans*-2ar**) δ 7.37 – 7.28 (m, 5H), 6.98 – 6.92 (m, 2H), 6.42 (d, *J* = 8.4 Hz, 1H), 4.02 (s, 1H), 3.94 (d, *J* = 8.7 Hz, 1H), 2.76 (dd, *J* = 16.4, 4.8 Hz, 1H), 2.56 (dd, *J* = 16.4, 10.5 Hz, 1H), 2.08 – 2.00 (m, 1H), 0.84 (d, *J* = 6.7 Hz, 3H).

^{13}C NMR (151 MHz, $CDCl_3$ ***trans*-2ar**) δ 143.2, 143.0, 128.7, 128.5, 127.8, 127.4, 126.6, 122.3, 121.3, 114.2, 63.1, 34.9, 33.4, 18.4.

HRMS (ESI) m/z: $[M + Na]^+$ Calcd for $C_{16}H_{16}ClNNa$ 280.0863; found 280.0863.

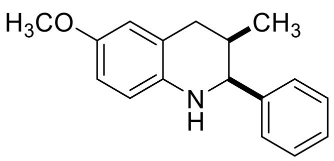

2as, 89%, *cis:trans* > 20:1

6-Methoxy-3-methyl-2-phenyl-1,2,3,4-tetrahydroquinoline **2as**. According to the **Method A**, using the alkene **1as-HCl** (0.2 mmol, 1.0 equiv.) and stirring at room temperature for 16 h under 10 W blue LED irradiation. The crude residue was purified by chromatography (pentane/EtOAc 5/1) to give **2as** (45.0 mg, 0.180 mmol, 89%) as a colorless liquid.

^{1}H NMR (300 MHz, $CDCl_3$, ***cis*-2as**) δ 7.43 – 7.26 (m, 5H), 6.75 – 6.64 (m, 2H), 6.58 (d, *J* = 8.5 Hz, 1H), 4.52 (d, *J* = 3.3 Hz, 1H), 3.81 (s, 3H), 3.07 (dd, *J* = 16.3, 5.1 Hz, 1H), 2.54 (dd, *J* = 16.3, 5.9 Hz, 1H), 2.45 – 2.29 (m, 1H), 0.88 (d, *J* = 6.9 Hz, 3H).

^{13}C NMR (76 MHz, $CDCl_3$ ***cis*-2as**) δ 151.8, 143.2, 138.3, 128.2, 127.2, 127.1, 121.3, 115.1, 114.9, 113.0, 59.6, 55.8, 34.0, 32.2, 14.8.

The NMR data were in agreement with those reported in the literature.[14]

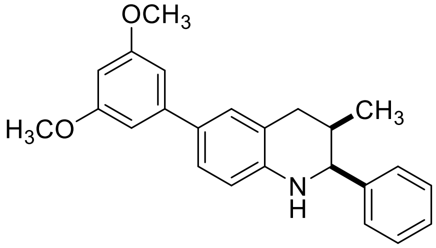

2at, 91%, *cis:trans* 10:1

6-(3,5-Dimethoxyphenyl)-3-methyl-2-phenyl-1,2,3,4-tetrahydroquinoline **2at**. According to the **Method A**, using the alkene **1at-HCl** (0.2 mmol, 1.0 equiv.) and stirring at room temperature for 16 h under 10 W blue LED irradiation. The crude residue was purified by chromatography (pentane/EtOAc 3/1) to give **2at** (65.3 mg, 0.180 mmol, 91%) as a colorless liquid (as a 1:10 trans:cis mixture of diastereoisomers).

^{1}H NMR (300 MHz, $CDCl_3$, ***cis*-2at**) δ 7.27 – 7.14 (m, 7H), 6.62 (d, *J* = 2.3 Hz, 2H), 6.52 (d, *J* = 8.1 Hz, 1H), 6.31 (t, *J* = 2.3 Hz, 1H), 4.45 (d, *J* = 3.6 Hz, 1H), 3.75 (s, 6H), 2.92 (dd, *J* = 16.2, 4.9 Hz, 1H), 2.47 (dd, *J* = 16.1, 6.9 Hz, 1H), 2.25 (dtd, *J* = 8.6, 5.9, 4.0 Hz, 1H), 0.76 (d, *J* = 6.9 Hz, 3H).

^{13}**C NMR** (76 MHz, $CDCl_3$, ***cis*-2at**) δ 161.0, 144.0, 143.7, 142.7, 129.9, 128.4, 128.2, 127.2, 125.8, 120.2, 113.9, 104.5, 98.2, 59.5, 55.4, 33.4, 31.9, 15.3.

HRMS (ESI) m/z: $[M + Na]^+$ Calcd for $C_{24}H_{25}NO_2Na$ 382.1777; found 382.1777.

4.3 C-H functionalization of 2-substituted quinolines

The conditions in Table S1, Entry 2 can be applied to synthesize the C-H functionalization product ***2'*:**

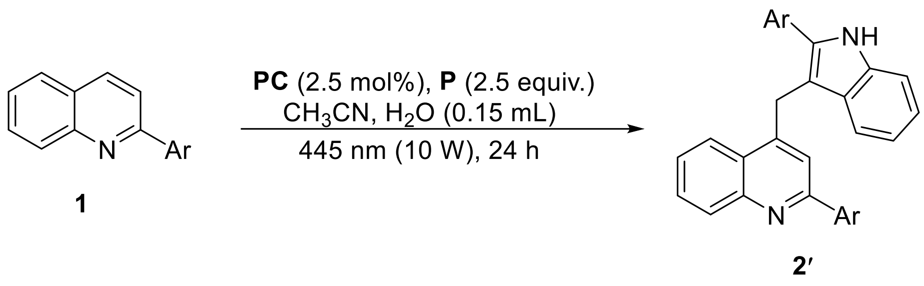

General procedure (GP) for C-H functionalization of 2-substituted quinolines: To an oven dried Schlenk tube with a magnetic stirring bar, **P** (0.5 mmol, 2.5 equiv.), **PC** (0.005 mmol, 2.5 mol%), deionized water (0.15 mL) and acetonitrile (4.0 mL) were added under argon atmosphere using standard Schlenk techniques at ambient temperature. Then, the quinoline **2** (0.2 mmol, 1.0 equiv.) and the HAT catalyst **ArSH** (0.04 mmol, 0.2 equiv.) were added under argon atmosphere. The tube was sealed and placed in the photoreactor, and was then irradiated with a 10 W 445 nm LED at 20 ^{o}C using the standard set-up. After 24 hours, the irradiation was stopped. The volatiles were removed in vacuo, then the crude residue was purified by flash column chromatography on silica (pentane, pentane/EtOAc or pentane/Et_2O mixtures, as detailed in the individual entries), to afford the corresponding product **2'**.

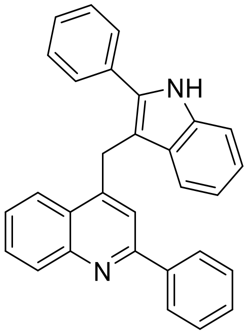

2a′, 64%

2-Phenyl-4-((2-phenyl-1H-indol-3-yl)methyl)quinoline **2a'**. According to the **GP**, using **1a** (0.2 mmol, 1.0 equiv.) and stirring at room temperature for 24 h under 10 W blue LED irradiation. The crude residue was purified by chromatography (pentane/EtOAc 6/1) to give **2a'** (25.4 mg, 0.064 mmol, 64%) as a colorless liquid.

^{1}H NMR (300 MHz, $CDCl_3$) δ 8.35 (s, 1H), 8.25 (d, *J* = 7.5 Hz, 1H), 8.20 (d, *J* = 8.0 Hz, 1H), 7.91 – 7.84 (m, 2H), 7.77 (ddd, *J* = 8.4, 6.9, 1.4 Hz, 1H), 7.62 – 7.54 (m, 2H), 7.51 – 7.45 (m, 3H), 7.43 – 7.33 (m, 7H), 7.25 (ddd, *J* = 8.1, 6.8, 1.2 Hz, 1H), 7.08 (ddd, *J* = 8.0, 7.0, 1.0 Hz, 1H), 4.75 (d, *J* = 1.2 Hz, 2H).

^{13}C NMR (76 MHz, $CDCl_3$) δ 157.6, 148.3, 147.4, 140.0, 136.3, 136.2, 132.6, 130.5, 129.4, 129.3, 129.1, 129.0, 128.6, 128.0, 127.6, 126.7, 126.2, 123.1, 122.7, 120.2, 119.3, 118.4, 111.0, 108.4, 27.4.

HRMS (ESI) m/z: $[M + Na]^+$ Calcd for $C_{30}H_{22}N_2Na$ 433.1675; found 433.1675.

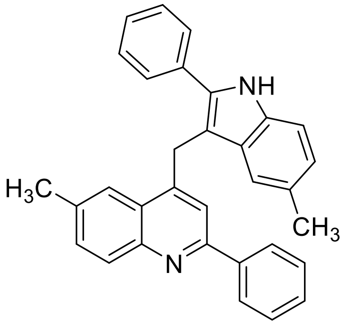

2b', 71%

6-Methyl-4-((5-methyl-2-phenyl-1*H*-indol-3-yl)methyl)-2-phenylquinoline **2b'**. According to the **GP**, using **1b** (0.2 mmol, 1.0 equiv.) and stirring at room temperature for 24 h under 10 W blue LED irradiation. The crude residue was purified by chromatography (pentane/EtOAc 6/1) to give **2b'** (31.1 mg, 0.071 mmol, 71%) as a colorless liquid.

^{1}H NMR (400 MHz, $CDCl_3$) δ 8.24 (s, 1H), 8.14 (d, *J* = 8.6 Hz, 1H), 7.96 (s, 1H), 7.91 – 7.84 (m, 2H), 7.60 (dd, *J* = 8.6, 1.9 Hz, 1H), 7.53 (s, 1H), 7.51 – 7.45 (m, 2H), 7.36 (dtd, *J* = 12.2, 7.2, 3.9 Hz, 6H), 7.18 (s, 1H), 7.07 (dd, *J* = 8.3, 1.6 Hz, 1H), 4.69 (s, 2H), 2.58 (s, 3H), 2.36 (s, 3H).

^{13}C NMR (101 MHz, $CDCl_3$) δ 156.7, 146.8, 146.7, 140.1, 136.3, 136.1, 134.5, 132.8, 131.5, 130.2, 129.8, 129.4, 129.1, 128.9, 128.6, 127.9, 127.6, 127.5, 126.7, 124.3, 122.3, 118.9, 118.4, 110.7, 108.0, 27.4, 22.0, 21.5.

HRMS (ESI) m/z: $[M + Na]^+$ Calcd for $C_{32}H_{26}N_2Na$ 461.1988; found 461.1988.

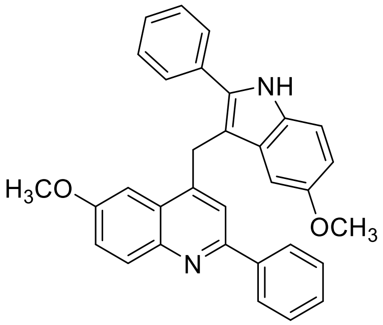

2c', 40%

6-Methoxy-4-((5-methoxy-2-phenyl-1*H*-indol-3-yl)methyl)-2-phenylquinoline **2c'**. According to the **GP**, using **1c** (0.2 mmol, 1.0 equiv.) and stirring at room temperature for 24 h under 10 W blue LED irradiation. The crude residue was purified by chromatography (pentane/EtOAc 6/1) to give **2c'** (18.8 mg, 0.040 mmol, 40%) as a colorless liquid.

^{1}H NMR (599 MHz, $CDCl_3$) δ 8.17 (s, 1H), 8.14 (d, *J* = 9.5 Hz, 1H), 7.88 – 7.85 (m, 2H), 7.56 (s, 1H), 7.48 – 7.43 (m, 2H), 7.41 (dd, *J* = 9.2, 2.7 Hz, 1H), 7.39 – 7.30 (m, 8H), 6.89 (dd, *J* = 8.8, 2.4 Hz, 1H), 6.81 (d, *J* = 2.4 Hz, 1H), 4.63 (s, 2H), 3.89 (s, 3H), 3.69 (s, 3H).

^{13}C NMR (151 MHz, $CDCl_3$) δ 157.7, 155.1, 154.5, 136.9, 132.6, 131.9, 131.2, 129.9, 129.1, 128.7, 128.6, 127.9, 127.6, 127.5, 127.3, 121.5, 118.7, 113.0, 111.8, 101.5, 100.8, 55.8, 55.5, 27.9.

HRMS (ESI) m/z: $[M + Na]^+$ Calcd for $C_{32}H_{26}N_2Na$ 493.1886; found 493.1886.

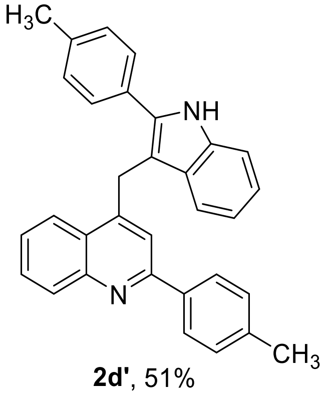

2d', 51%

2-(p-Tolyl)-4-((2-(p-tolyl)-1*H*-indol-3-yl)methyl)quinoline **2d'**. According to the **GP**, using **1d** (0.2 mmol, 1.0 equiv.) and stirring at room temperature for 24 h under 10 W blue LED irradiation. The crude residue was purified by chromatography (pentane/EtOAc 6/1) to give **2d'** (22.3 mg, 0.051 mmol, 51%) as a colorless liquid.

^{1}H NMR (599 MHz, $CDCl_3$) δ 8.26 (s, 1H), 8.22 (d, *J* = 8.4 Hz, 1H), 8.19 – 8.15 (m, 1H), 7.77 (d, *J* = 8.2 Hz, 2H), 7.76 – 7.72 (m, 1H), 7.57 – 7.52 (m, 2H), 7.45 (d, *J* = 8.1 Hz, 1H), 7.38 – 7.33 (m, 3H), 7.22 (ddd, *J* = 8.1, 7.0, 1.1 Hz, 1H), 7.20 – 7.15 (m, 4H), 7.06 (ddd, *J* = 8.0, 7.0, 1.0 Hz, 1H), 4.71 (s, 2H), 2.34 (s, 3H), 2.34 (s, 3H).

^{13}C NMR (151 MHz, $CDCl_3$) δ 157.6, 148.4, 147.4, 139.2, 138.1, 136.5, 136.2, 130.5, 129.9, 129.6, 129.5, 129.3, 128.3, 128.1, 127.7, 127.6, 126.8, 126.1, 123.3, 122.7, 120.2, 119.3, 118.5, 111.0, 108.2, 27.5, 21.4, 21.4.

HRMS (ESI) m/z: $[M + Na]^+$ Calcd for $C_{32}H_{26}N_2Na$ 461.1988; found 461.1988.

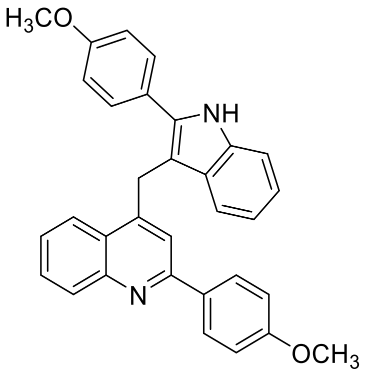

2e', 49%

2-(4-Methoxyphenyl)-4-((2-(4-methoxyphenyl)-1*H*-indol-3-yl)methyl)quinoline **2e'**. According to the **GP**, using **1e** (0.2 mmol, 1.0 equiv.) and stirring at room temperature for 24 h under 10 W blue LED irradiation. The crude residue was purified by chromatography (pentane/EtOAc 6/1) to give **2e'** (23.0 mg, 0.049 mmol, 49%) as a colorless liquid.

^{1}H NMR (599 MHz, $CDCl_3$) δ 8.22 (s, 2H), 8.17 (d, *J* = 8.3 Hz, 1H), 7.85 (d, *J* = 8.8 Hz, 2H), 7.74 (t, *J* = 7.7 Hz, 1H), 7.55 (t, *J* = 7.6 Hz, 1H), 7.52 (s, 1H), 7.46 (d, *J* = 8.1 Hz, 1H), 7.41 – 7.37 (m, 3H), 7.23 (ddd, *J* = 8.1, 7.1, 1.1 Hz, 1H), 7.07 (ddd, *J* = 8.0, 7.1, 0.9 Hz, 1H), 6.93 – 6.88 (m, 4H), 4.69 (s, 2H), 3.81 (s, 3H), 3.79 (s, 3H).

^{13}C NMR (151 MHz, $CDCl_3$) δ 159.5, 136.2, 135.9, 129.5, 128.9, 126.4, 125.8, 125.0, 123.1, 122.4, 120.1, 119.0, 118.0, 114.5, 114.0, 110.8, 107.6, 55.3, 27.4.

HRMS (ESI) m/z: $[M + Na]^+$ Calcd for $C_{32}H_{26}N_2Na$ 493.1886; found 493.1886.

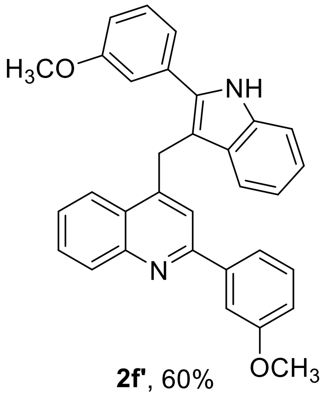

2f', 60%

2-(3-Methoxyphenyl)-4-((2-(3-methoxyphenyl)-1*H*-indol-3-yl)methyl)quinoline **2f'**. According to the **GP**, using **1f** (0.2 mmol, 1.0 equiv.) and stirring at room temperature for 24 h under 10 W blue LED irradiation. The crude residue was purified by chromatography (pentane/EtOAc 6/1) to give **2f'** (28.2 mg, 0.060 mmol, 60%) as a colorless liquid.

^{1}H NMR (300 MHz, $CDCl_3$) δ 8.30 (s, 1H), 8.14 (s, 1H), 8.12 (d, *J* = 7.1 Hz, 1H), 7.68 (t, *J* = 7.7 Hz, 1H), 7.50 (t, *J* = 6.9 Hz, 1H), 7.46 (s, 1H), 7.40 (d, *J* = 2.5 Hz, 1H), 7.36 (d, *J* = 5.6 Hz, 1H), 7.31 (d, *J* = 9.3 Hz, 1H), 7.25 – 7.13 (m, 4H), 7.00 (q, *J* = 7.3 Hz, 2H), 6.88 (d, *J* = 4.2 Hz, 1H), 6.85 – 6.75 (m, 2H), 4.67 (s, 2H), 3.71 (s, 3H), 3.47 (s, 3H).

^{13}C NMR (76 MHz, $CDCl_3$) δ 159.9, 159.9, 157.4, 148.2, 147.5, 141.4, 136.1, 136.1, 133.9, 130.5, 130.2, 129.6, 129.5, 129.4, 126.7, 126.3, 123.2, 122.8, 120.2, 120.1, 120.0, 119.2, 118.7, 115.3, 113.9, 112.9, 112.6, 111.1, 108.5, 55.3, 55.0, 27.3.

HRMS (ESI) m/z: $[M + Na]^+$ Calcd for $C_{32}H_{26}N_2Na$ 493.1886; found 493.1886.

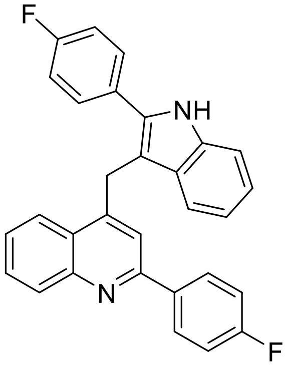

2g', 63%

2-(4-Fluorophenyl)-4-((2-(4-fluorophenyl)-1*H*-indol-3-yl)methyl)quinoline **2g'**. According to the **GP**, using **1g** (0.2 mmol, 1.0 equiv.) and stirring at room temperature for 24 h under 10 W blue LED irradiation. The crude residue was purified by chromatography (pentane/EtOAc 6/1) to give **2g'** (28.1 mg, 0.063 mmol, 63%) as a colorless liquid.

^{1}H NMR (500 MHz, $CDCl_3$) δ 8.38 (s, 1H), 8.22 (d, *J* = 7.9 Hz, 1H), 8.17 (d, *J* = 9.5 Hz, 1H), 7.90 – 7.81 (m, 2H), 7.77 (ddd, *J* = 8.4, 6.9, 1.4 Hz, 1H), 7.58 (ddd, *J* = 8.3, 6.9, 1.3 Hz, 1H), 7.49 (s, 1H), 7.47 (d, *J* = 8.2 Hz, 1H), 7.43 – 7.38 (m, 3H), 7.28 – 7.24 (m, 1H), 7.12 – 7.09 (m, 1H), 7.06 (tdd, *J* = 8.7, 4.0, 2.2 Hz, 4H), 4.69 (d, *J* = 1.2 Hz, 2H).

^{13}C NMR (126 MHz, $CDCl_3$) δ 163.6, 162.5, 156.3, 148.2, 147.4, 136.1, 136.0, 135.3, 130.4, 129.5, 129.4, 129.3, 128.7, 126.5, 126.3, 123.1, 122.8, 120.3, 119.2, 117.9, 116.1, 115.5, 111.1, 108.3, 27.3.

^{19}F NMR (470 MHz, $CDCl_3$) δ -112.7, -113.0.

HRMS (ESI) m/z: $[M + Na]^+$ Calcd for $C_{30}H_{20}F_2N_2Na$ 469.1487; found 469.1487.

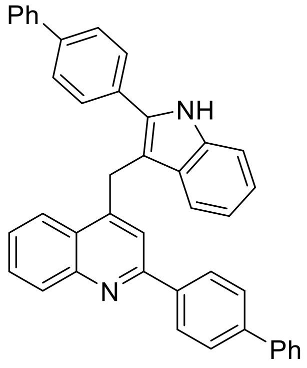

2h', 58%

2-([1,1'-Biphenyl]-4-yl)-4-((2-([1,1'-biphenyl]-4-yl)-1*H*-indol-3-yl)methyl)quinoline **2h'**. According to the **GP**, using **1h** (0.2 mmol, 1.0 equiv.) and stirring at room temperature for 36 h under 10 W blue LED irradiation. The crude residue was purified by chromatography (pentane/EtOAc 6/1) to give **2h'** (32.6 mg, 0.058 mmol, 58%) as a colorless liquid.

^{1}H NMR (400 MHz, $CDCl_3$) δ 8.28 (s, 1H), 8.20 (d, *J* = 9.0 Hz, 1H), 8.15 (d, *J* = 8.5 Hz, 1H), 7.90 (d, *J* = 8.5 Hz, 2H), 7.73 – 7.68 (m, 1H), 7.56 – 7.48 (m, 11H), 7.42 (d, *J* = 8.1 Hz, 1H), 7.35 (t, *J* = 7.8 Hz, 6H), 7.29 – 7.23 (m, 3H), 7.22 – 7.19 (m, 1H), 7.03 (ddd, *J* = 8.0, 7.1, 1.0 Hz, 1H), 4.72 (d, *J* = 1.2 Hz, 2H).

^{13}C NMR (101 MHz, $CDCl_3$) δ 157.1, 141.9, 140.8, 140.6, 140.2, 136.2, 135.9, 130.5, 129.5, 129.4, 128.9, 128.8, 128.03, 127.97, 127.8, 127.6, 127.5, 127.4, 127.1, 127.0, 126.7, 126.3, 123.2, 122.9, 120.3, 119.3, 118.4, 111.1, 108.7, 27.5.

HRMS (ESI) m/z: $[M + Na]^+$ Calcd for $C_{42}H_{30}N_2Na$ 585.2301; found 585.2301.

5. Stern-Volmer quenching experiments

Stern-Volmer luminescence quenching analysis was conducted using a Jasco FP8550 spectrofluorometer at 25 °C. The following parameters were employed: Excitation bandwidth = 5 nm, data interval = 0.5 nm, scan speed = 500 nm/min, response time = 2 sec. The samples were measured in Hellma fluorescence QS quartz cuvettes (chamber volume = 1.4 mL, H × W × D = 46 mm × 12.5 mm, 12.5 mm) fitted with a PTFE stopper. Samples were prepared in 1.4 mL quartz cuvettes equipped with PTFE stoppers inside an argon filled glove bag. The CH_3CN solution of Ir[dF(CF_3)ppy]$_2$(dtbbpy)PF_6 (1.5×10^{-6} M) were excited at λ_{ex} = 380 nm and the emission was recorded at 474 nm. For each quenching experiment, the quenching reagent (2.5×10^{-4} M) was titrated in 100 µl steps to a solution (300 µl) of Ir[dF(CF_3)ppy]$_2$(dtbbpy)PF_6 in a screw-top 10.0 mm quartz cuvette. Then, cuvette was filled with CH_3CN to a total volume of 1 mL. I_0 is the luminescence intensity without the quencher, I is the intensity in the presence of the quencher. The results are listed below:

Entry	**P** / mM	I_0/I	[**1a**-HCl] / mM	I_0/I	[**2ag**] / mM	I_0/I
1	0	1	0	1	0	1
2	0.025	1.11	0.025	1.04	0.025	1.49
3	0.050	1.17	0.050	1.15	0.050	1.72
4	0.075	1.33	0.075	1.27	0.075	1.96
5	0.100	1.45	0.100	1.40	0.100	2.19
6	0.125	1.54	0.125	1.47	0.125	2.45

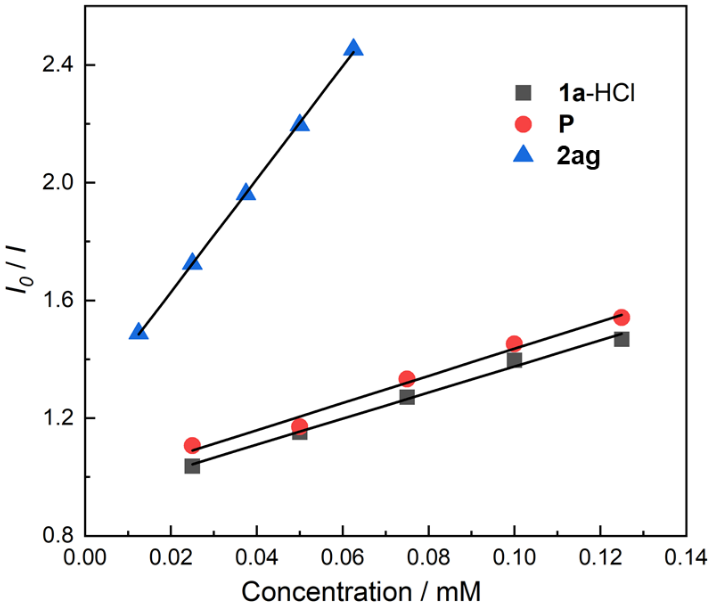

6. Cyclic voltammetry

Equipment

Cyclic voltammetry (CV) experiments were carried out in an air-tight three-electrode undivided measuring cell (rhd instruments, TSC 1600 closed) with an approximate sample volume of 1.0 mL, with a platinum disc (diameter 0.25 mm) as the working electrode (WE) and a platinum crucible as the counter electrode (CE). A silver wire pseudo-reference electrode (rhd instruments, Ag wire MicroPseudo reference) was used. Measurements were performed using a Metrohm Autolab potentiostat (Metrohm, PGSTAT204) and data were collected and analysed using the Autolab Nova 2.1 program. Figures were prepared for publication using the Python libraries pandas and matplotlib.

Materials

Acetonitrile was obtained from Acros Organics in extra-dry grade, then degassed using three freeze-pump-thaw cycles and stored over activated molecular sieves (3 Å) under argon. Tetrabutylammonium hexafluorophosphate (>99.0%, for electrochemical analysis) was purchased from Sigma Aldrich. Ferrocene (high purity, 99+%) was purchased from Alfa Aesar. All chemicals were used without further purification.

Experimental

Experiments were performed on 1 mL of a 2 mM solution of the analyte in acetonitrile, with 0.1 M $[Bu_4N][PF_6]$ as the supporting electrolyte, at a scan rate of 100 mV s^{-1}. The starting potential was chosen so as to avoid redox events and was set to 0.0 V in the case of the free base, and 0.3 V in the case of the

hydrochloride salt. These potentials are vs the Ag pseudo-reference electrode and correspond to potentials vs Fc/Fc^+ of roughly −0.3 and 0.0 V respectively. For both depicted CVs, the initial scanning direction was towards positive potentials; scanning in the reverse direction did not reveal significantly different behaviour. In all cases 2–3 scans were measured.

Samples were prepared as follows: the solids were weighed and dried in a vial on a Schlenk line before filling the headspace with argon, and bringing into an argon-filled glove bag. The working electrode of the cell was polished before use by making figure-of-eight motions in a water-alumina (0.05 µm) slurry on a microfibre polishing pad, after which alumina residues were removed by sonication and rinsing with deionized water. The solids were dissolved in dry, degassed acetonitrile, and transferred to the cell under argon in the glove bag. After successful measurement the cell was returned to the glove bag and reopened, a small quantity of a stock solution of ferrocene was added, and the sample with the internal reference remeasured with a fresh working electrode. All potentials were then referenced to the potential of the Fc/Fc^+ redox couple. Potentials are also provided referenced to SCE for convenience, for which a conversion factor of +0.380 V for Fc/Fc^+ vs SCE has been used.

Standard potentials E_0 of reversible redox events, or irreversible events where both anodic and cathodic peaks were discernible, were estimated as usual using half-wave potentials $E_{½}$. For irreversible peaks, the inflection point of the curve was instead used as the best estimate of the standard potential. Voltages were not compensated for IR drop.

Results and Discussion

The cyclic voltammogram of the quinoline **1a** (Figure S1 or Figure S2) shows a single partially reversible reduction at −2.42 V vs Fc/Fc^+ (−2.04 V vs SCE). With a freshly polished electrode, the wave appears almost reversible, however repeated scans lead to increasing irreversibility. An attempt to measure at different scan rates in sequence showed this clearly (Figure S3), as the CV at 100 mV s^{-1} using a fresh electrode (Figure S1) is significantly more reversible than that measured after three scans each at 500 and 200 mV s^{-1}. This implies passivation of the Pt working electrode, presumably by the product of the single-electron reduction. This is further demonstrated by the shift of the solvent electrochemical window to a more negative potential between first and subsequent measurements (Figure S2). The addition of ferrocene to the cell for use as an internal reference caused the reduction to become completely irreversible.

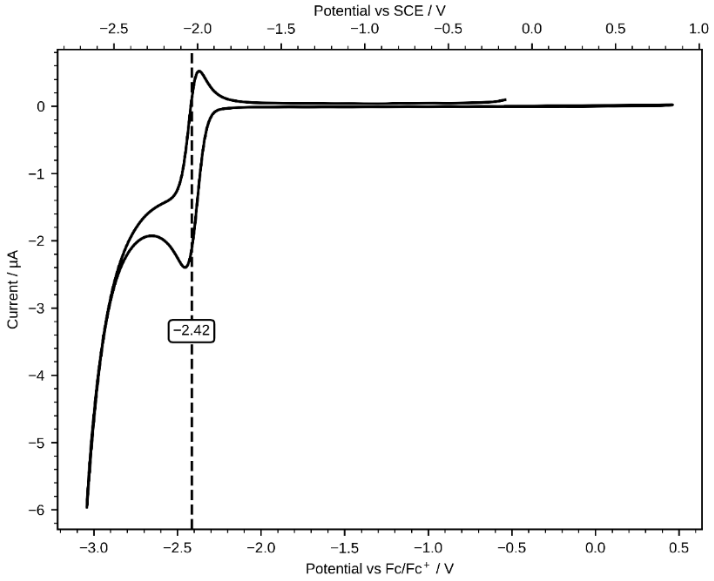

Figure S2 Cyclic voltammogram of the quinoline **1a** (2 mM) in acetonitrile with [Bu_4N^+][PF_6^-] (0.1 M) as the supporting electrolyte, at a scan rate of 100 mV s^{-1}, with a 0.25 mm diameter Pt working electrode. Potentials were referenced separately to the Fc/Fc^+ redox couple using ferrocene as an internal standard. Only the first scan is shown.

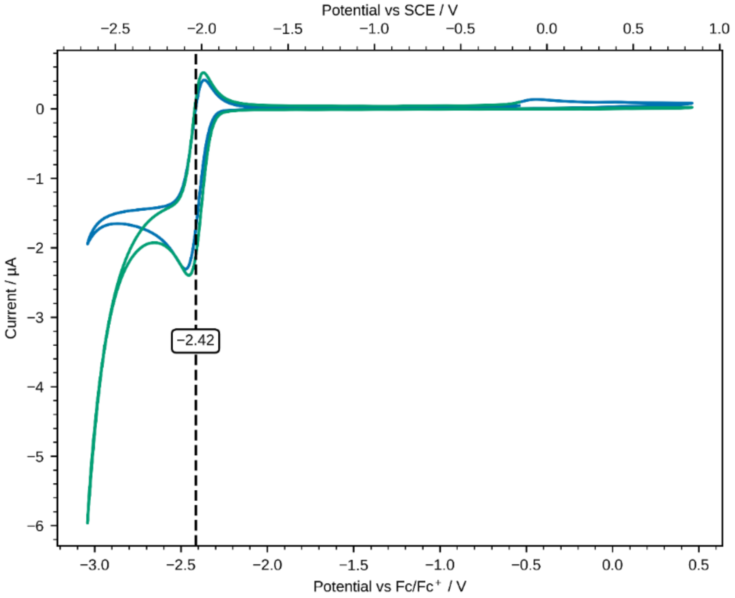

Figure S3 Cyclic voltammogram of the quinoline **1a** (2 mM) in acetonitrile with [Bu_4N^+][PF_6^-] (0.1 M) as the supporting electrolyte, at a scan rate of 100 mV s^{-1}, with a 0.25 mm diameter Pt working electrode. Potentials were referenced separately to the Fc/Fc^+ redox couple using ferrocene as an internal standard. Both the first (green) and second (blue) scans are shown.

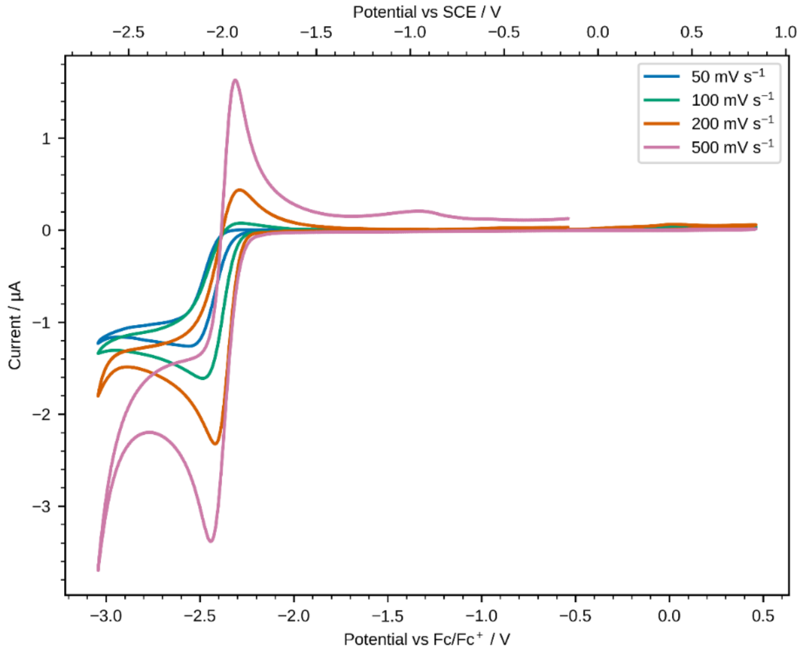

Figure S4 Sequential voltammograms at various scan rates of the quinoline **1a** (2 mM) in acetonitrile with $[Bu_4N^+][PF_6^-]$ (0.1 M) as the supporting electrolyte, with a 0.25 mm diameter Pt working electrode. Three scans were performed at each scan rate, going from fast to slow. The electrode was not changed or cleaned between scans. Only the first scan of each scan rate is shown. Direct comparison of the scan here at 100 mV s^{-1} (green) and that in Figure 1 with a fresh electrode demonstrates the increasing irreversibility with increasing number of completed scans, even accounting for the effect of scan rate.

Meanwhile the cyclic voltammogram of the quinoline HCl salt **1a**-HCl (Figure S4) shows two main reduction events. The first is a broad irreversible reduction peak at −1.03 V vs Fc/Fc^+ (−0.65 V vs SCE). The second comes at an almost identical potential (−2.41 V vs Fc/Fc^+ (−2.03 V vs SCE)) to the reduction of the free base, so it is assumed that it is also here reduction of the free base that is observed. Given its potential and the working electrode material (platinum), it is plausible that the initial reduction at −1.03 V is a hydrogen evolution reaction, resulting in free base in the vicinity of the WE that can then be further reduced. Whether hydrogen gas could evolve in the absence of an electrode, indeed, one that excels in catalyzing such reactions, is not known. The interesting form of the second reduction peak may be due to protonation of the reduced species in the acidic environment. Of note is also that the separation of the two reduction waves decreases significantly (−0.2 V) upon the addition of ferrocene (Figure S4, dashed orange curve). As the potential of the second reduction matches that of the free base, it was used to reference the whole voltammogram to the Fc/Fc^+ redox pair. This choice leads to the conclusion that the reduction of the protonated species is affected significantly by the presence of ferrocene.

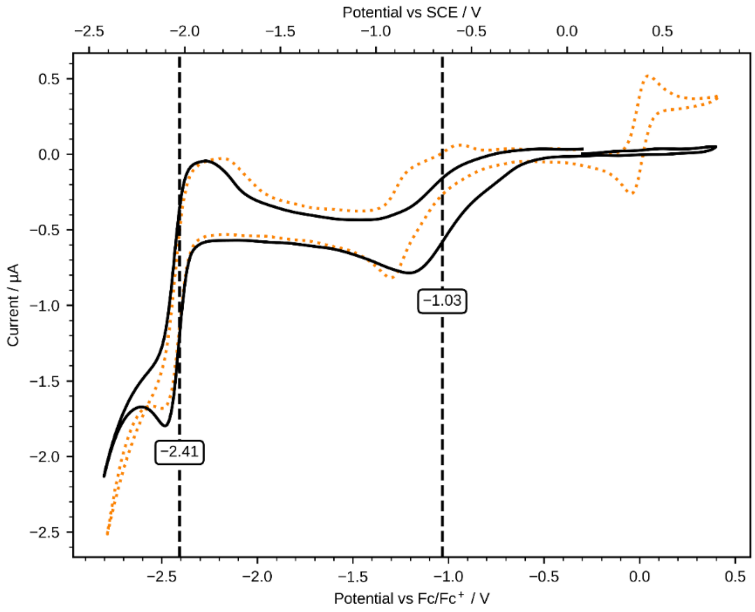

Figure S5 Cyclic voltammogram (black, solid) of the quinoline HCl salt **1a**-HCl (2 mM) in acetonitrile with $[Bu_4N^+][PF_6^-]$ (0.1 M) as the supporting electrolyte, at a scan rate of 100 mV s^{-1}, with a 0.25 mm diameter Pt working electrode. Potentials were referenced to the Fc/Fc^+ redox couple using ferrocene as an internal standard (orange, dotted).

7. Fractionation slope measurement

The *cis*-**2ag** was synthesized via the reported method[14] and was then applied to investigate reaction mechanism. The NMR data are listed below:

^{1}H NMR (300 MHz, $CDCl_3$) δ 7.30 – 7.19 (m, 2H), 7.10 – 6.99 (m, 2H), 6.90 (d, *J* = 8.7 Hz, 2H), 6.73 – 6.64 (m, 1H), 6.59 (d, *J* = 7.8 Hz, 1H), 4.50 (d, *J* = 3.5 Hz, 1H), 4.13 (s, 1H), 3.84 (s, 3H), 2.99 (dd, *J* = 16.1, 5.0 Hz, 1H), 2.53 (dd, *J* = 16.1, 6.9 Hz, 1H), 2.30 (tdd, *J* = 6.8, 4.9, 3.6 Hz, 1H), 0.86 (d, *J* = 6.9 Hz, 3H).

The NMR data were in agreement with those reported in the literature.[14]

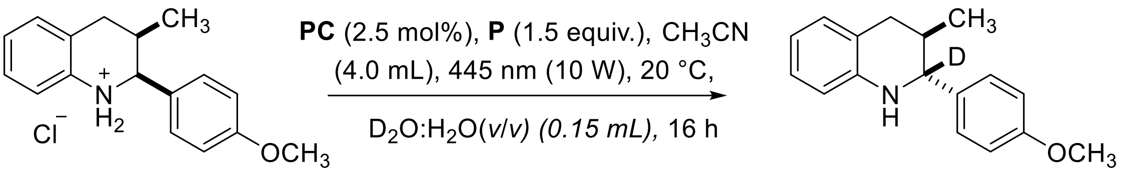

cis-**2ag**-HCl

A. D_2O:H_2O(*v*/*v*) = 1/3 **2ag-D**/**2ag** = 0.43

B. D_2O:H_2O(*v*/*v*) = 1/1 **2ag-D**/**2ag**= 1.08

C. D_2O:H_2O(*v*/*v*) = 3/1 **2ag-D**/**2ag** = 3.18

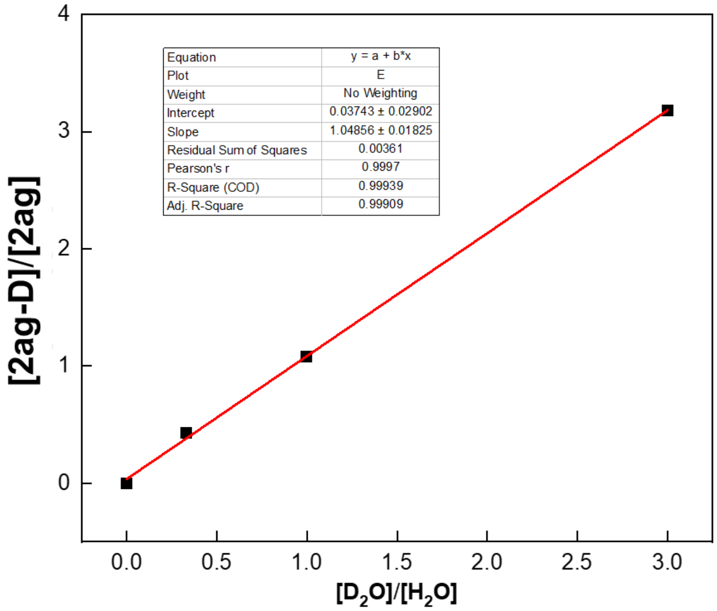

8. Deuteration experiment

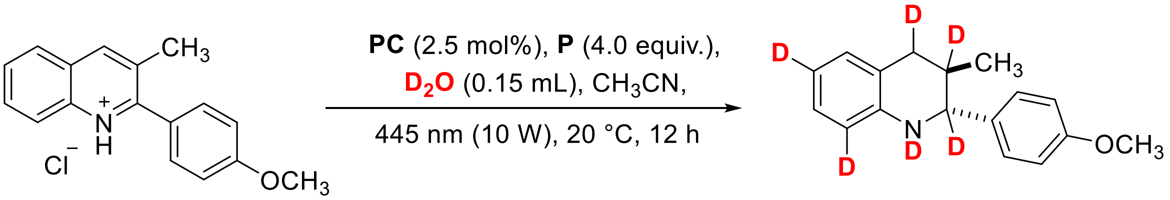

1ag-HCl

2ag-D_6, 75%, D-inc: > 90%
trans:cis 1:1

To an oven dried Schlenk tube with a magnetic stirring bar, **P** (0.8 mmol, 4.0 equiv.), **PC** (0.005 mmol, 2.5 mol%), D_2O (0.15 mL) and acetonitrile (4.0 mL) were added under argon atmosphere using standard Schlenk techniques at ambient temperature. Then, the quinoline HCl salt **1ag-HCl** (0.2 mmol, 1.0 equiv.) was added under argon atmosphere. The tube was sealed and placed in the photoreactor, and then irradiated with a 10 W 445 nm LED at 20 °C using the standard set-up. After 16 hours, the irradiation was stopped. The volatiles were removed in vacuo, then the crude residue was purified by flash column chromatography on silica (pentane/EtOAc 5/1), to afford the corresponding product **2ag-**D_6 as a 1:1 trans:cis mixture of diastereoisomers.

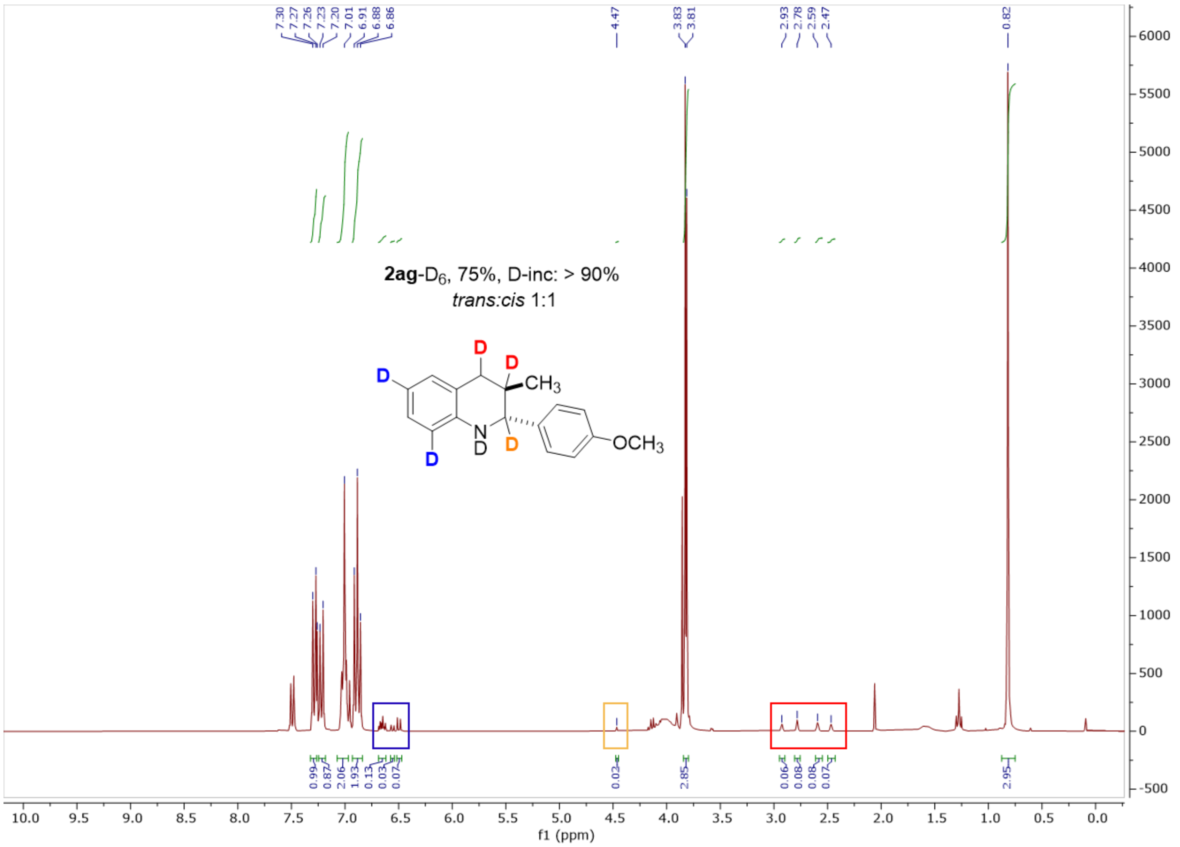

9. Time course of the trans/cis selectivity

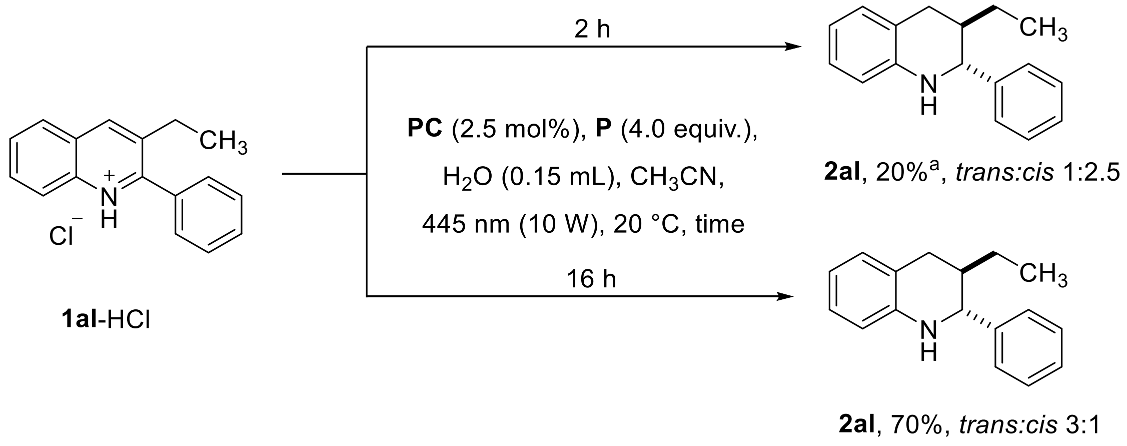

These two reactions were set up in parallel and the general process is shown below:

To an oven dried Schlenk tube with a magnetic stirring bar, **P** (0.8 mmol, 4.0 equiv.), **PC** (0.005 mmol, 2.5 mol%), D_2O (0.15 mL) and acetonitrile (4.0 mL) were added under argon atmosphere using standard Schlenk techniques at ambient temperature. Then, the quinoline HCl salt **1al-HCl** (0.2 mmol, 1.0 equiv.) was added under argon atmosphere. The tube was sealed and placed in the photoreactor, and then irradiated with a 10 W 445 nm LED at 20 ^{o}C using the standard set-up. After 2 hours (the reaction time for the other one is 16 hours), the irradiation was stopped. The volatiles were removed in vacuo, then the crude residue was purified by flash column chromatography on silica (pentane/EtOAc 10/1), to afford the corresponding product **2al** as a 1:2.5 trans:cis mixture of diastereoisomers.

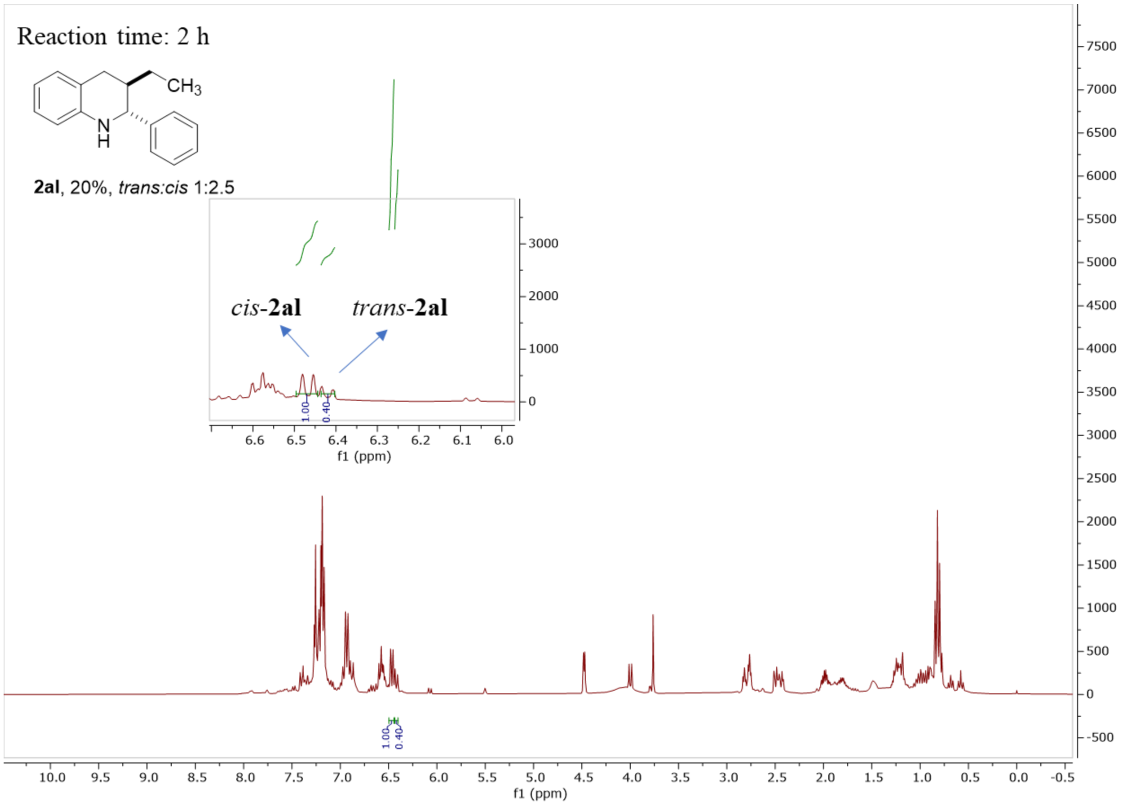

10. The investigation of the photoisomerization intermediate

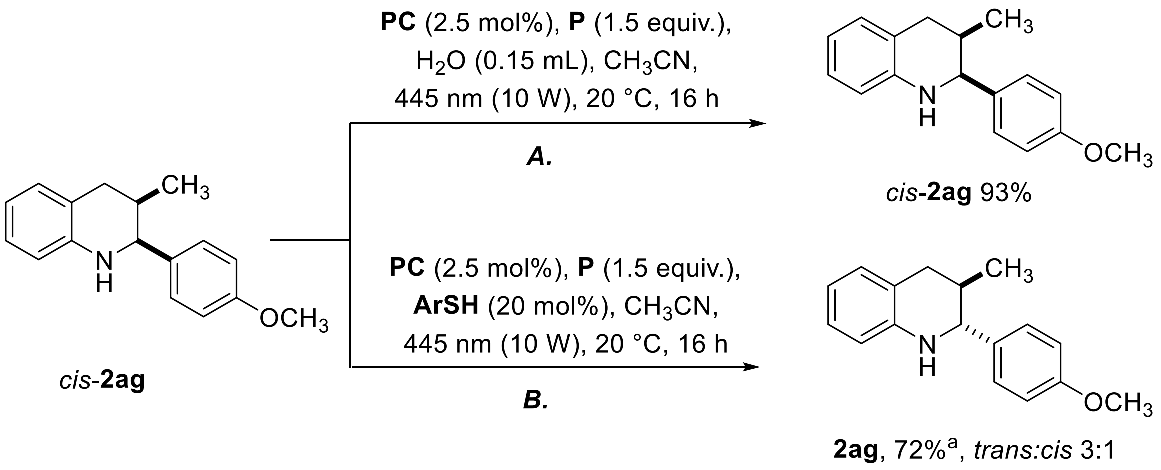

Process A:

To an oven dried Schlenk tube with a magnetic stirring bar, **P** (0.5 mmol, 1.5 equiv.), **PC** (0.005 mmol, 2.5 mol%), H_2O (0.15 mL) and acetonitrile (4.0 mL) were added under argon atmosphere using standard Schlenk techniques at ambient temperature. Then, the *cis*-**2ag** (0.2 mmol, 1.0 equiv.) was added under argon

atmosphere. The tube was sealed and placed in the photoreactor, and was then irradiated with a 10 W 445 nm LED at 20 °C using the standard set-up. After 16 hours, the irradiation was stopped. The volatiles were removed in vacuo, then the crude residue was purified by flash column chromatography on silica (pentane/EtOAc 10/1), to afford the corresponding product *cis*-**2ag**.

Process B:

To an oven dried Schlenk tube with a magnetic stirring bar, **P** (0.5 mmol, 1.5 equiv.), **PC** (0.005 mmol, 2.5 mol%), **ArSH** (0.04 mmol, 0.2 equiv.) and acetonitrile (4.0 mL) were added under argon atmosphere using standard Schlenk techniques at ambient temperature. Then, the *cis*-**2ag** (0.2 mmol, 1.0 equiv.) was added under argon atmosphere. The tube was sealed and placed in the photoreactor, and was then irradiated with a 10 W 445 nm LED at 20 °C using the standard set-up. After 16 hours, the irradiation was stopped. The volatiles were removed in vacuo, then the crude residue was analyzed through ^{1}H NMR.

The crude NMR for **Process B**:

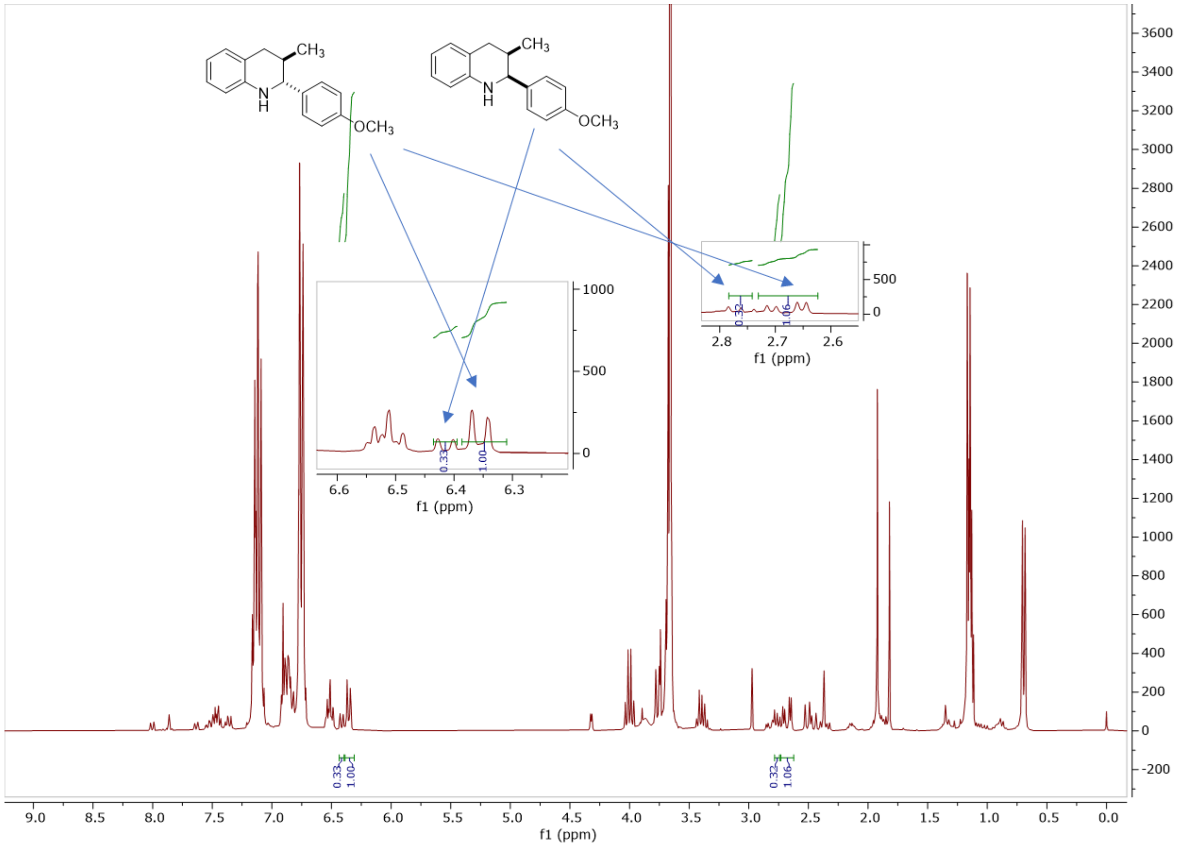

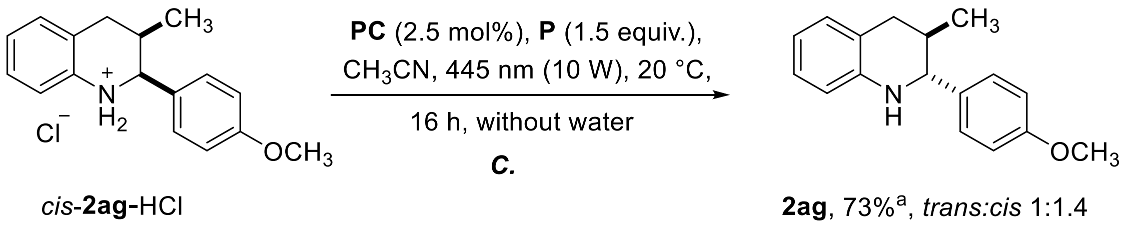

Process C:

To an oven dried Schlenk tube with a magnetic stirring bar, **P** (0.5 mmol, 1.5 equiv.), **PC** (0.005 mmol, 2.5 mol%) and acetonitrile (4.0 mL) were added under argon atmosphere using standard Schlenk techniques at ambient temperature. Then, the *cis*-**2ag-HCl** (0.2 mmol, 1.0 equiv.) was added under argon atmosphere. The tube was sealed and placed in the photoreactor, and was then irradiated with a 10 W 445 nm LED at 20 °C using the standard set-up. After 16 hours, the irradiation was stopped. The volatiles were removed in vacuo, then the crude residue was analyzed through ^{1}H NMR spectroscopy.

The crude NMR for **Process C**:

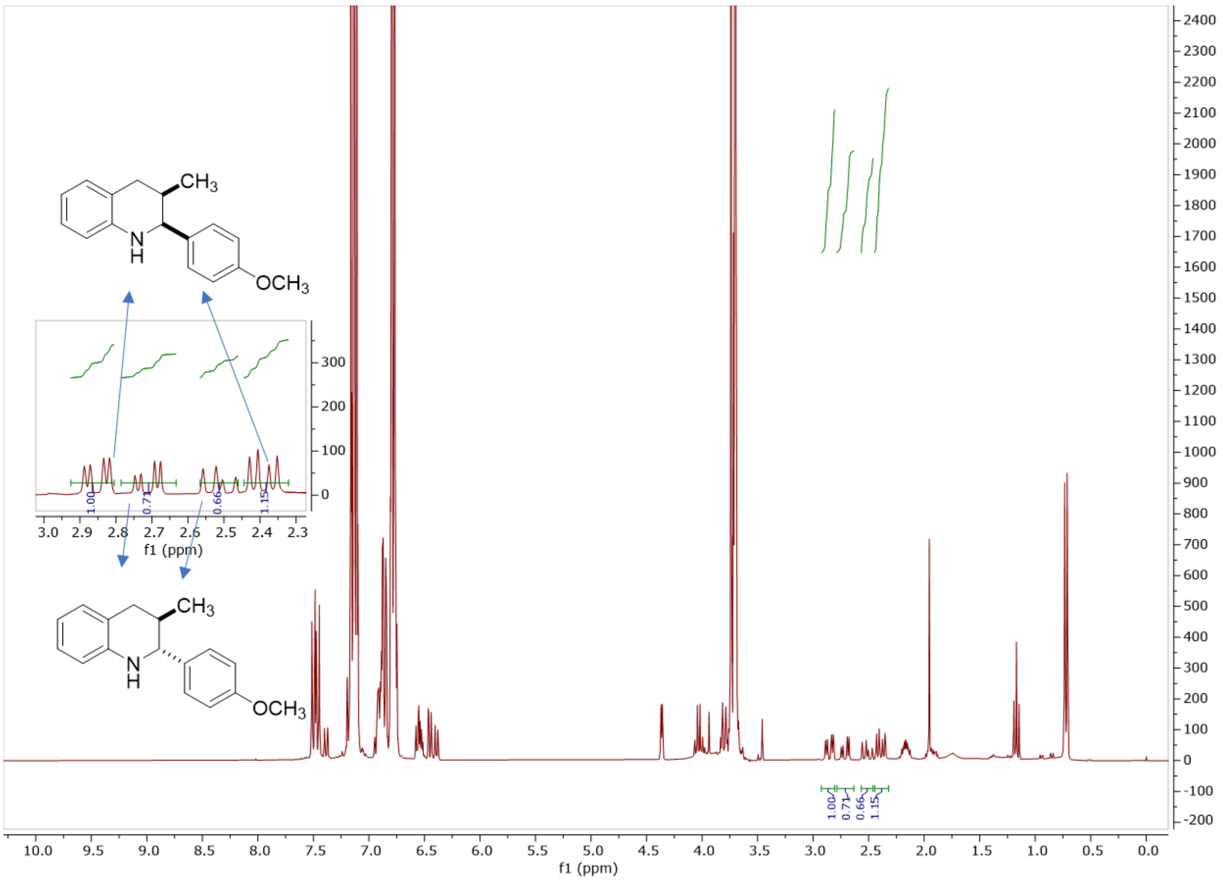

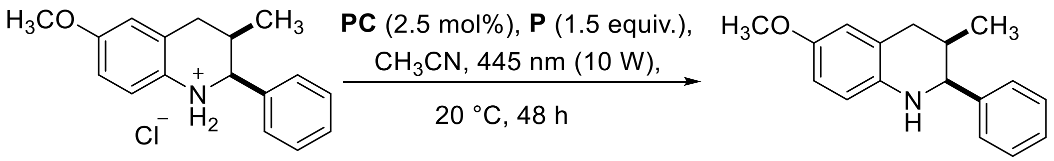

2as-HCl **2as**, 92%

According to the **Process C**, using the **2as-HCl** (0.2 mmol, 1.0 equiv.) and stirring at room temperature for 48 h under 10 W blue LED irradiation. The crude residue was purified by chromatography (pentane/EtOAc 8/1) to give **2as** as the pure isomer.

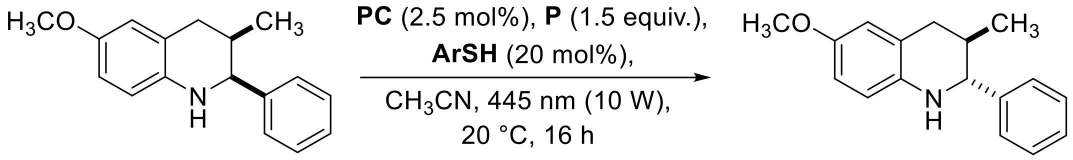

2as *trans*-**2as**, 81%, *trans:cis* 3:1

According to the **Process B**, using the **2as** (0.2 mmol, 1.0 equiv.) and stirring at room temperature for 16 h under 10 W blue LED irradiation. The volatiles were removed in vacuo, then the crude residue was analyzed through ^{1}H NMR spectroscopy.

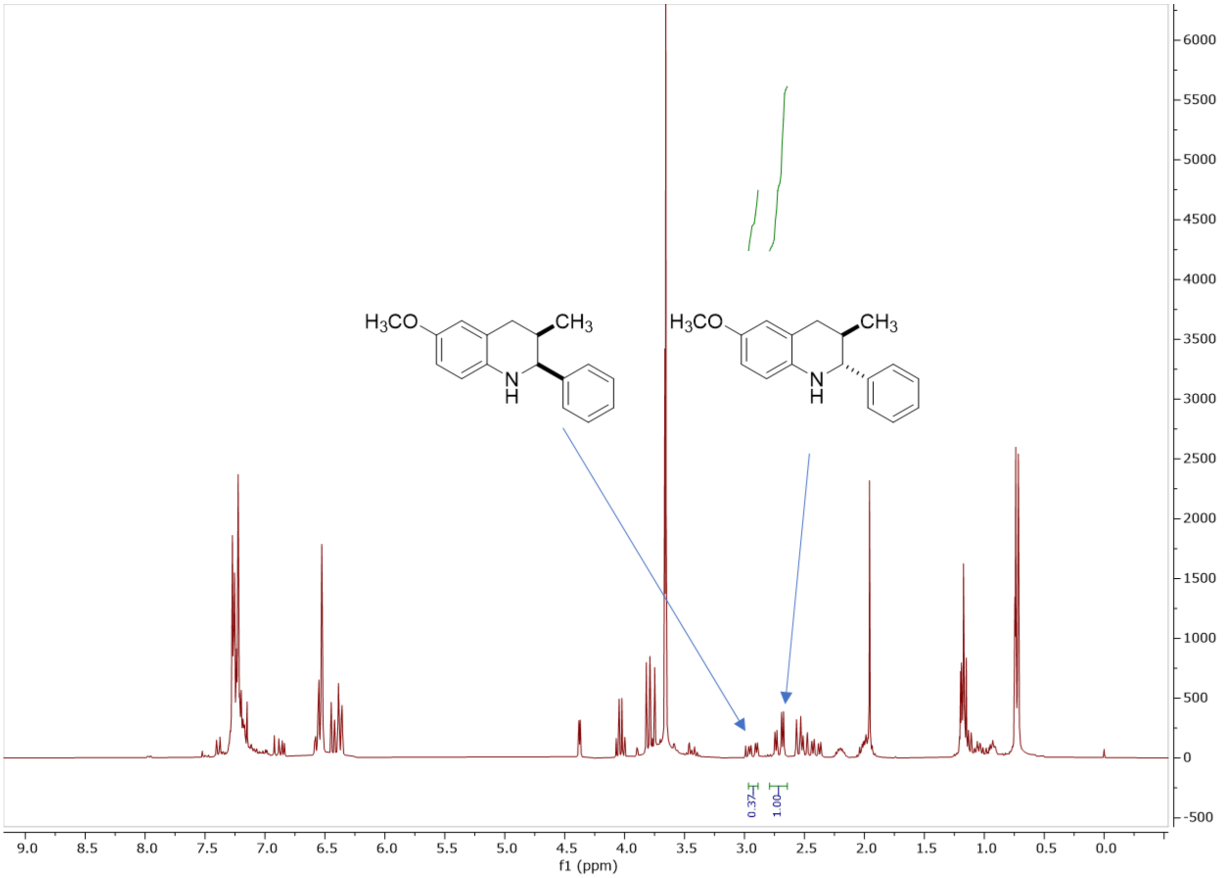
H3CO
CH3
N
H
H3CO
CH3
N
H
0.37
1.00
f1 (ppm)

11. Proton transfer equilibrium

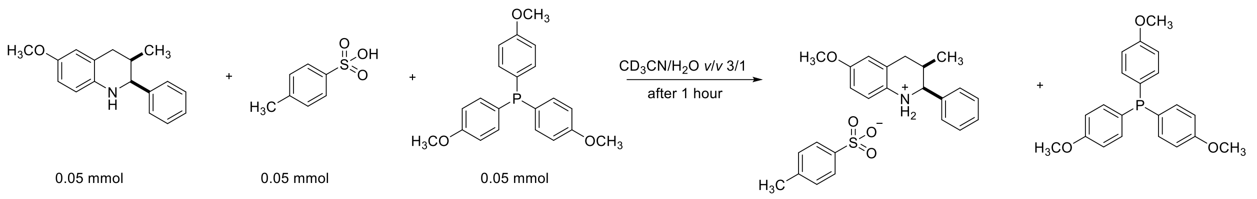

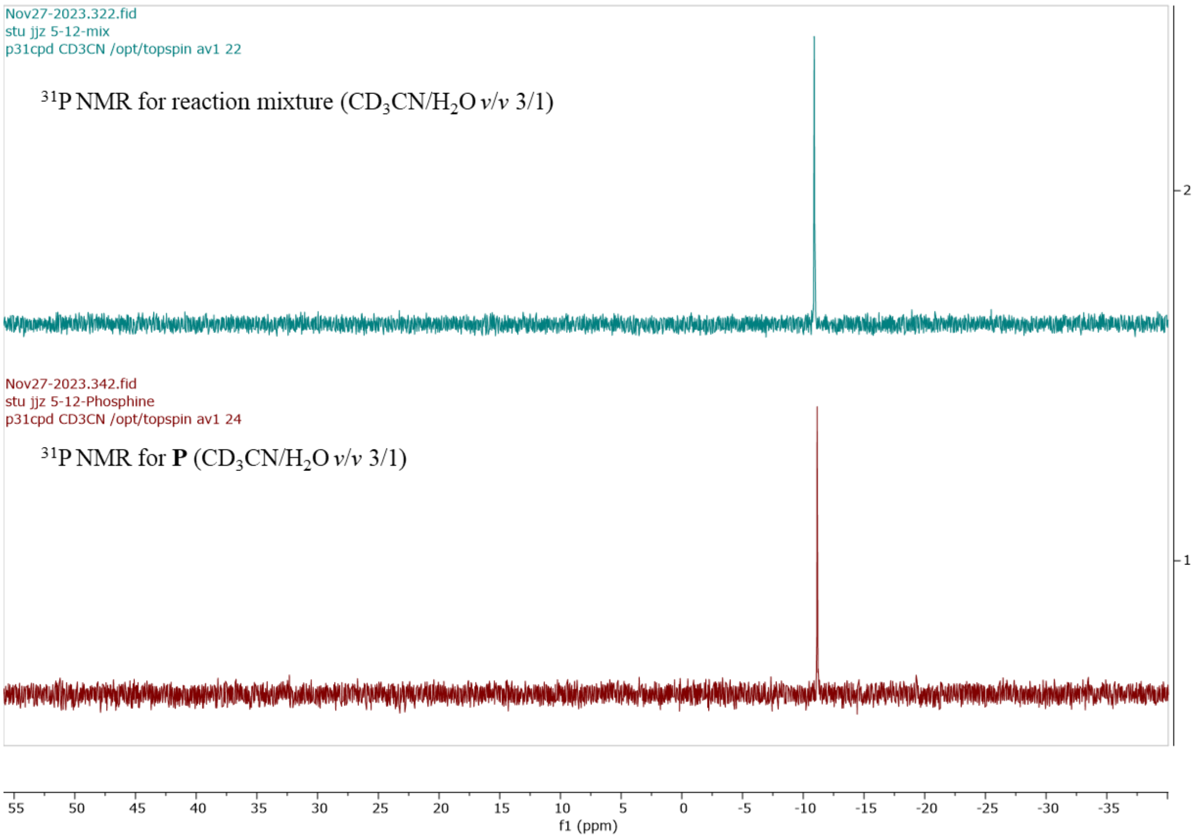

Comments: The mixed solvent was used to increase the solubility of the generated salt and simulate reaction conditions. According to the ^{31}P NMR spectra, the non-protonated phosphine was suggested, indicating that **2as** is more basic than **P**.

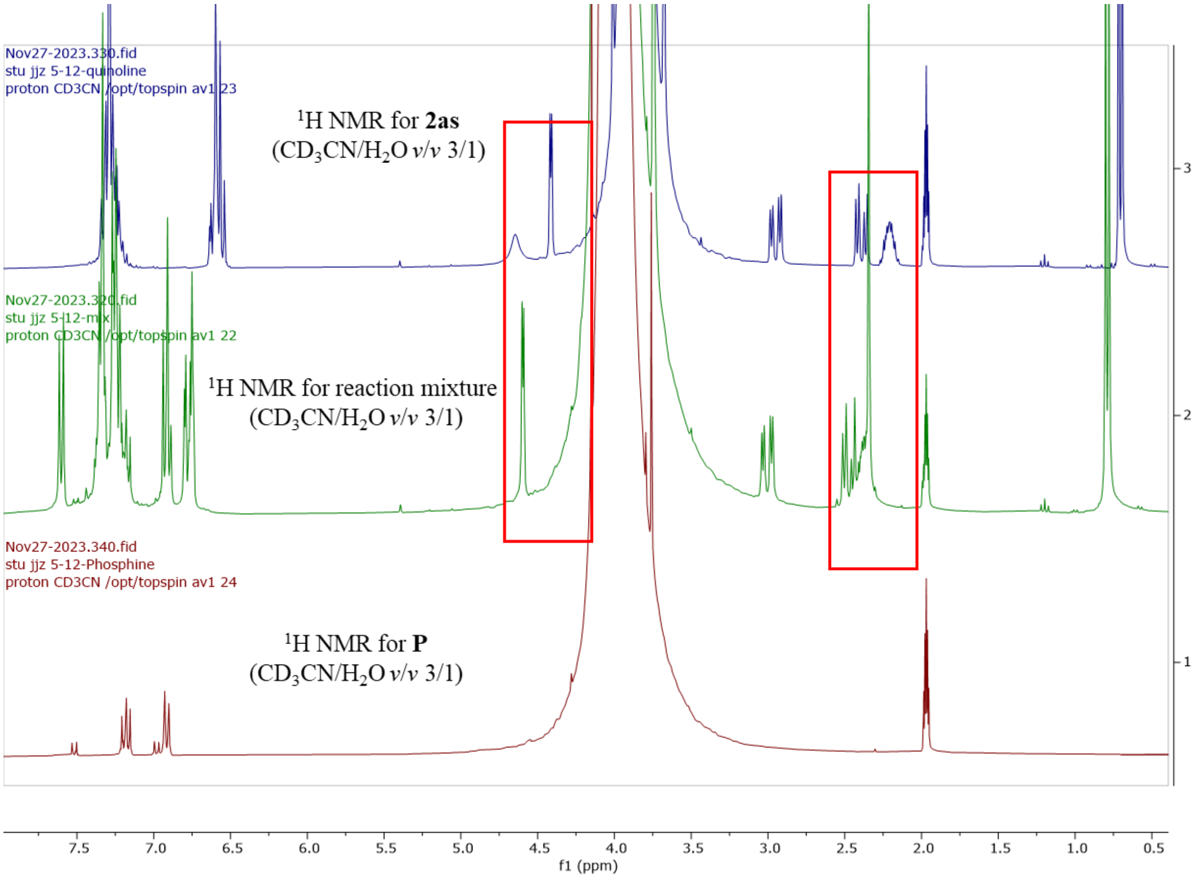

Comments According to the ^{1}H NMR spectrum, the completely protonated **2as** was suggested, also indicating that **2as** is more basic than **P**.

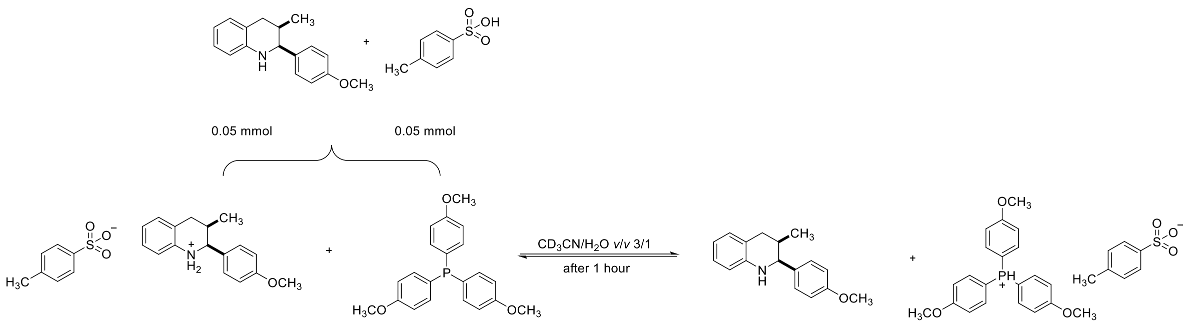

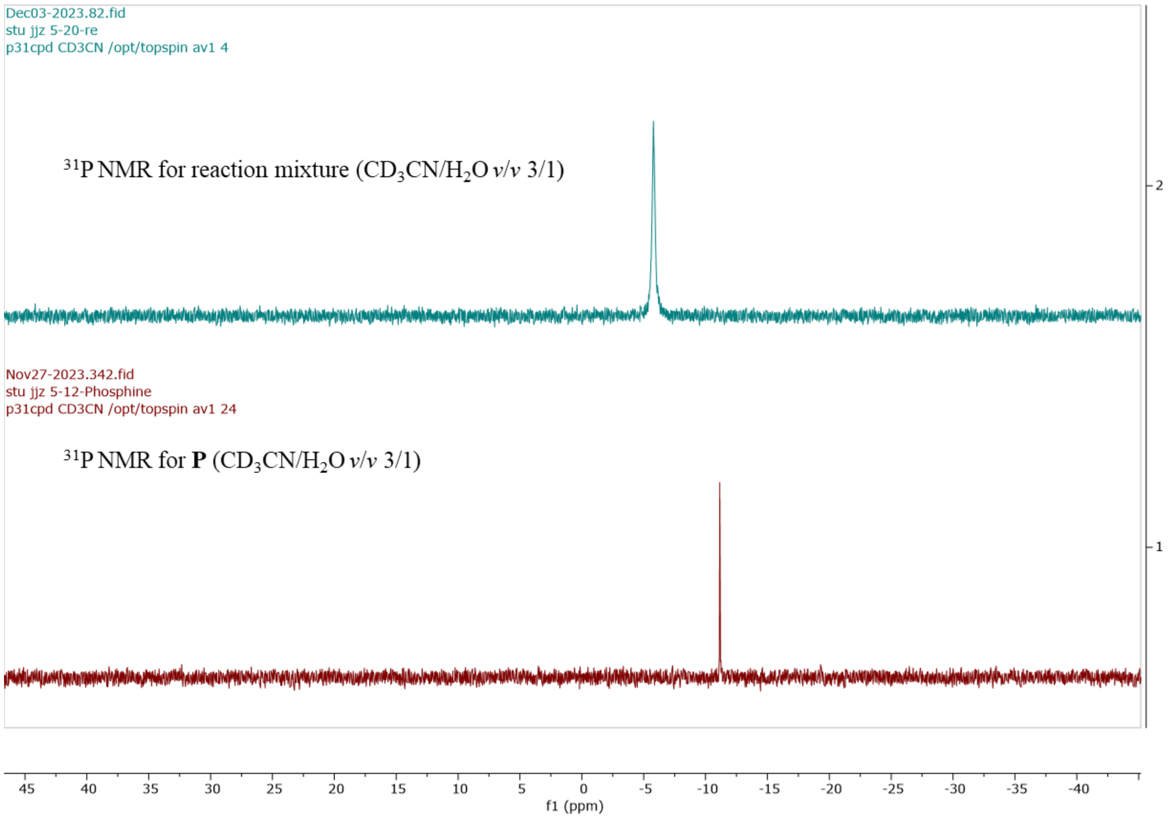

Comments: The mixed solvent was used to increase the solubility of the generated salt and simulate reaction conditions. According to the ^{31}P NMR spectra, the protonated phosphine was suggested.

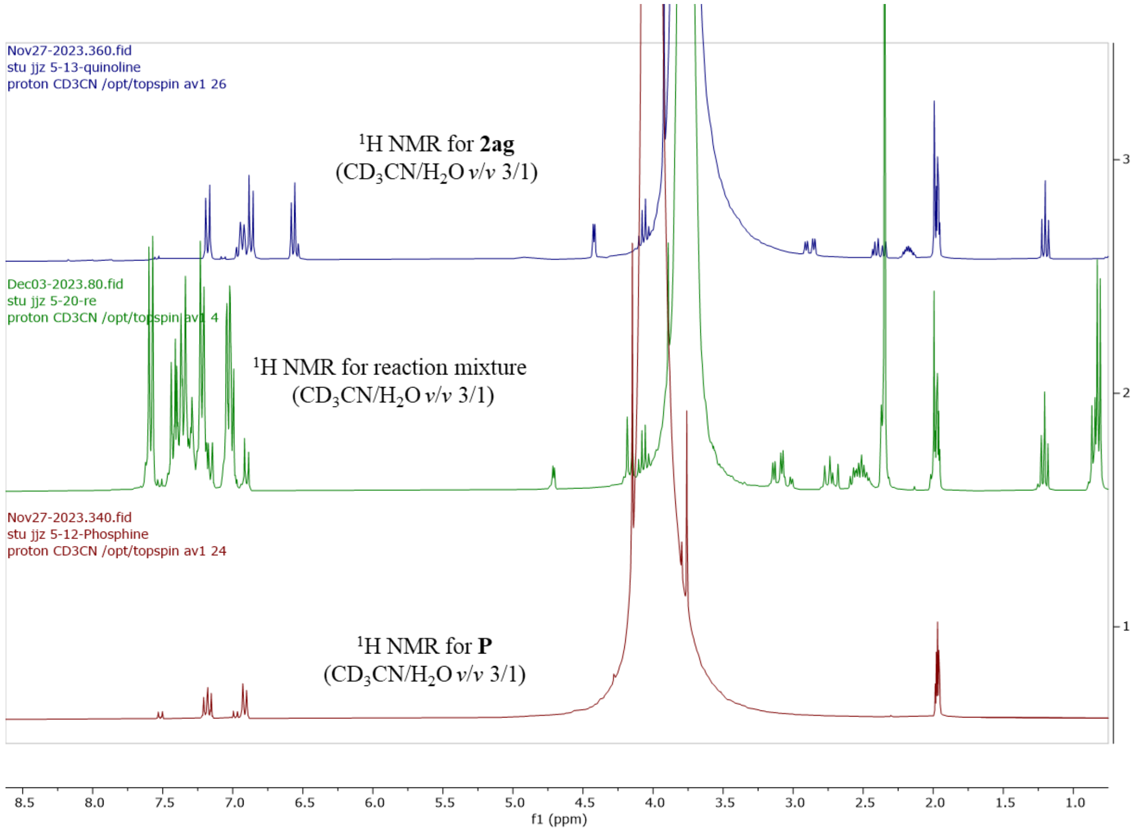

Comments: According to the ^{1}N NMR spectra, both protonated phosphine and protonated **2ag** were formed, indicating that **P** and **2ag** show comparable basicity. The proton transfer equilibrium was built**.**

12. Effect of phosphine on the diastereoselectivity

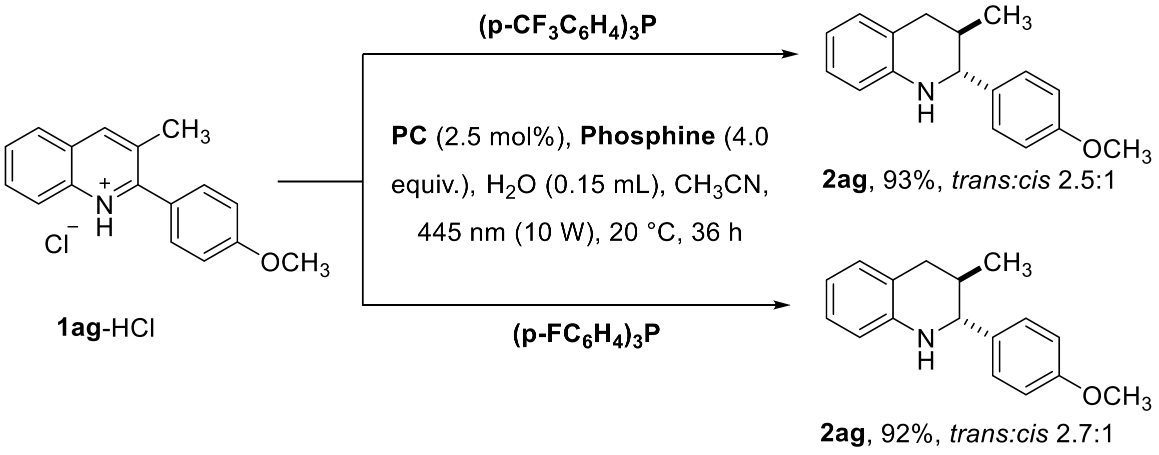

According to the **Method A**, using the **1ag-HCl** (0.2 mmol, 1.0 equiv.) and stirring at room temperature for 36 h under 10 W blue LED irradiation. The volatiles were removed in vacuo, then the crude residue was analyzed through ^{1}H NMR spectroscopy.

The reaction with **(p-CF$_3$C$_6$H$_4$)$_3$P:**

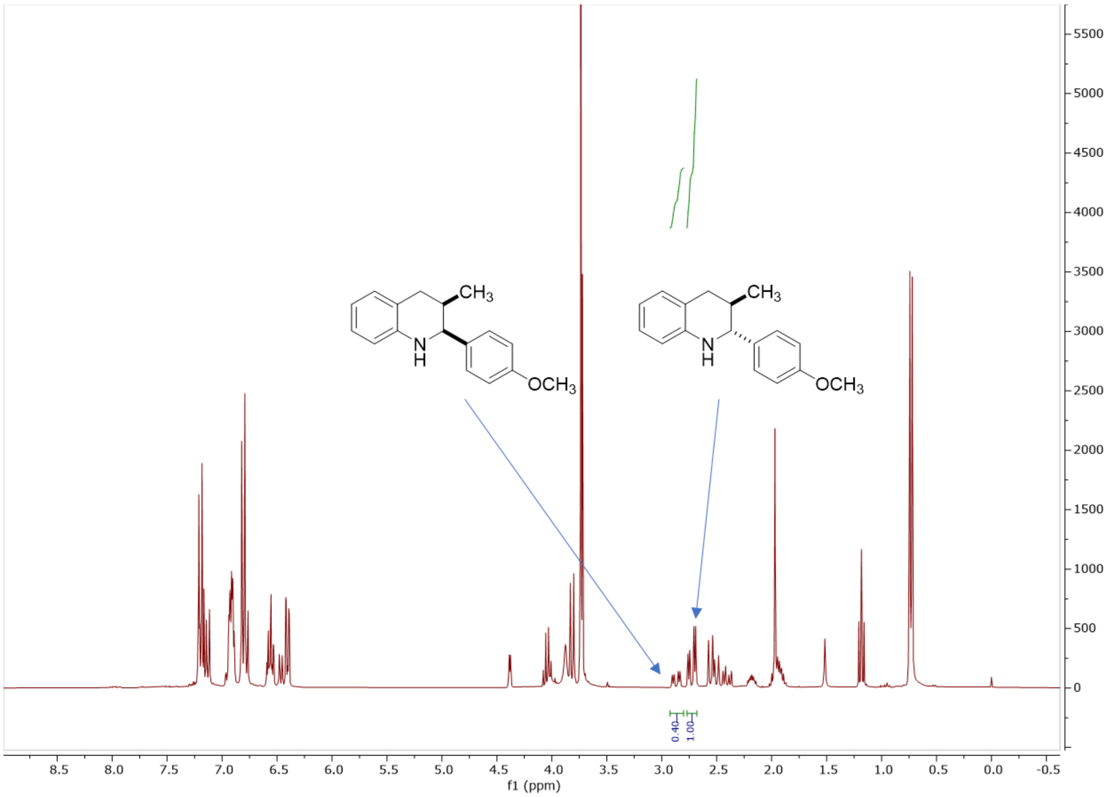

The reaction with **(p-FC$_6$H$_4$)$_3$P:**

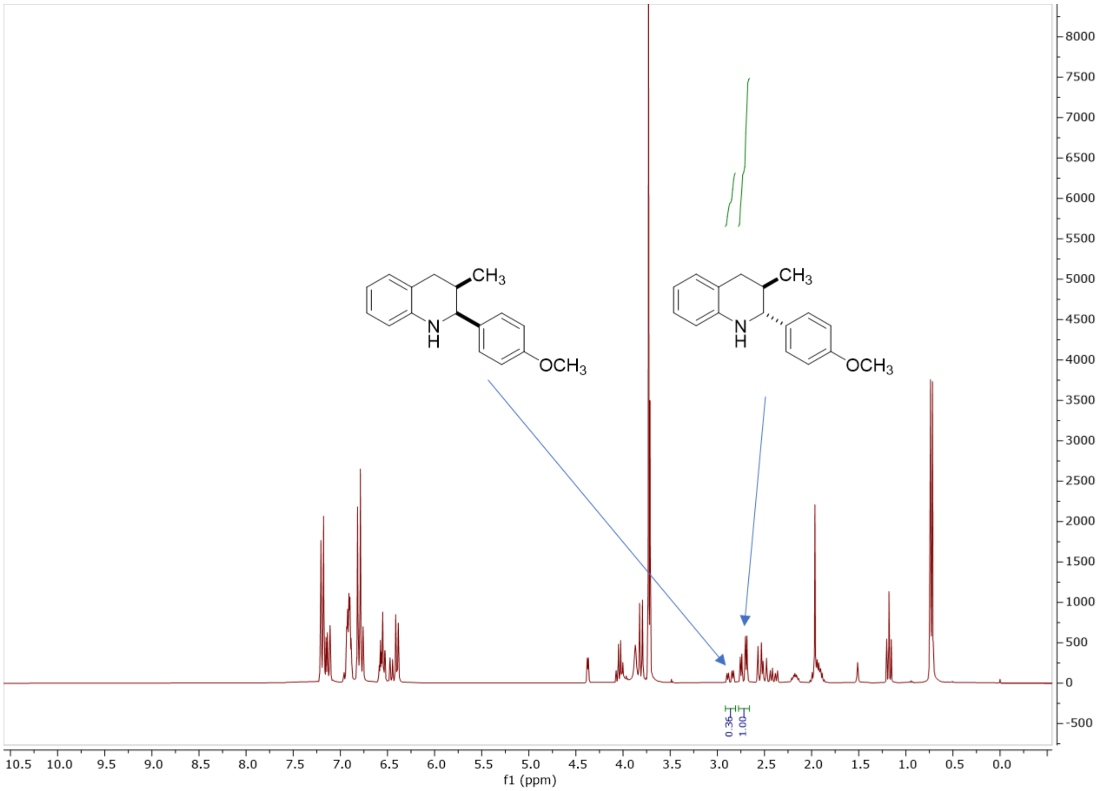

CH3
N
H
OCH3
CH3
N
H
OCH3
0.36
1.00
8000
7500
7000
6500
6000
5500
5000
4500
4000
3500
3000
2500
2000
1500
1000
500
0
-500
10.5 10.0 9.5 9.0 8.5 8.0 7.5 7.0 6.5 6.0 5.5 5.0 4.5 4.0 3.5 3.0 2.5 2.0 1.5 1.0 0.5 0.0
f1 (ppm)

13. The hydrogenation of 1,4-dihydroquinoline intermediate

The 1,4-hydrosilylation of 2-phenyl quinoline was performed based on the literature[36] with some adjustments. The reaction was performed in a glovebox using a Young-NMR tube. $B(C_6F_5)_3$ (13 mg, 5 mol%) was dissolved in $CDCl_3$ (0.25 mL) and the solution was added into the Young-NMR tube. The silane (77 µL, 68 mg, 1 eq.) was added into the Young-NMR tube and the mixture was briefly shaken. The quinoline (103 mg, 0.500 mmol, 1 eq.) was dissolved in $CDCl_3$ (0.75 mL) and the solution was added partly into the Young-NMR tube (0.05 mL every minute followed by shaking the tube). The product was obtained in 84% NMR yield. The hydrogenation of this 1,4-dihydroquinoline intermediate gave the corresponding tetrahydroquinoline product in 74% yield under our radical conditions, supporting the feasibility of our suggested initial 1,4-reduction process. Note that desilylation is extremely fast under aqueous conditions, therefore, directly the dihydroquinoline will be formed under the applied reaction conditions.

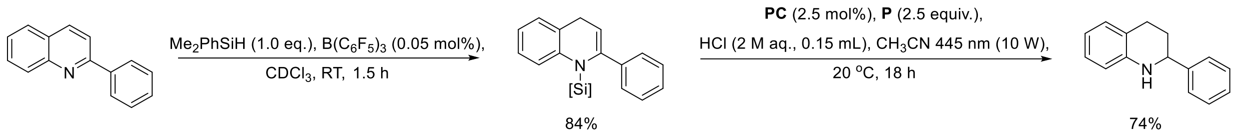

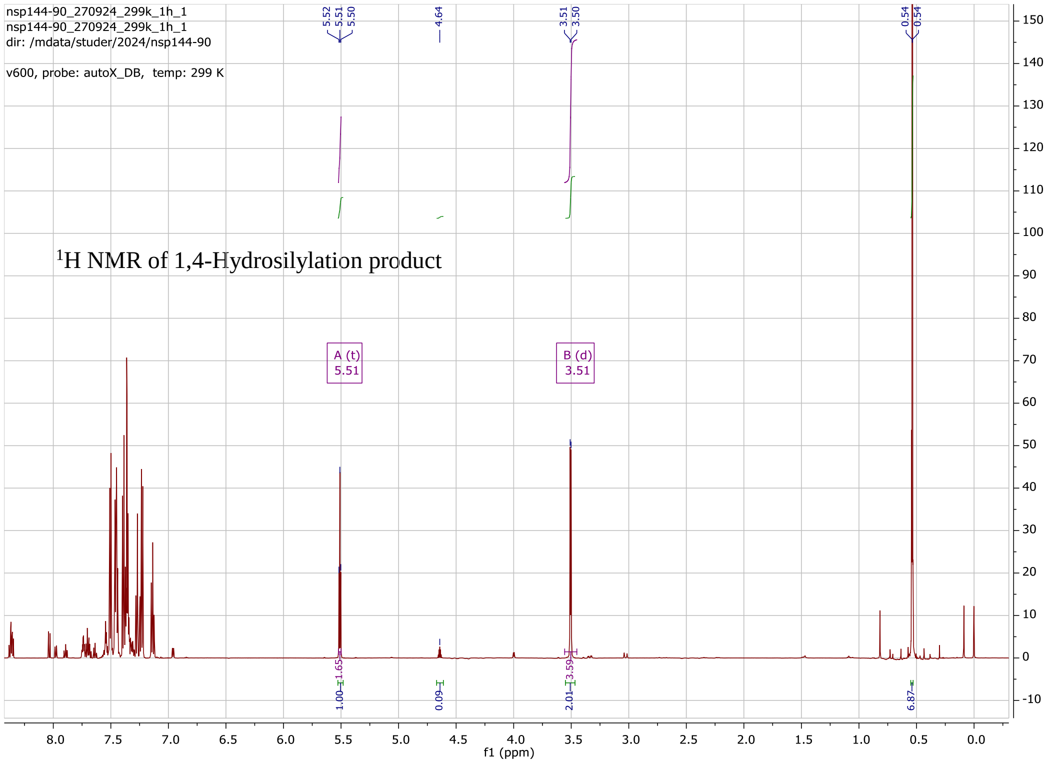

14. Kinetic isotope effect

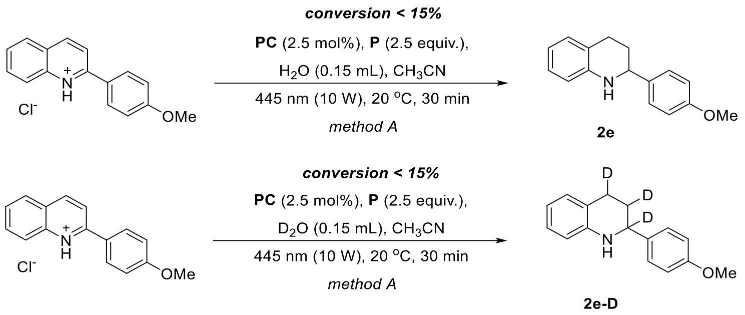

KIE = k_H/k_D ≈ yield(**2e**)/yield(**2e-D**) = 1.14

The KIE value of 1.14 for the hydrogenation of **2e**-HCl via Method A indicated that the hydrogen atom transfer is not in the rate-determining step.

15. DFT calculations

Methods

All structures were optimized without geometry constraints using the PBE0 hybrid functional[37,38] and an atom-pairwise dispersion correction (D3).[39,40] A flexible triple zeta basis set (def2-TZVP)[41] was used in all calculations. The nature of the optimized stationary points was proven by the number of imaginary harmonic vibrational frequencies (0 for minima, 1 for transition structures). For the determination of free enthalpy contributions at 298 K (G^{RRHO}), the rigid-rotor-harmonic-oscillator model and a rotor approximation for vibrational modes with wave numbers below 100 cm^{-1} was applied.[42] Electronic energies of the optimized structures were recalculated with the hybrid functional PW6B95(-D3)[43] using the def2-TZVP basis set. Nudged elastic band (NEB)[44,45] calculations were run using PBEh-3c.[46]

Solvation free energies (G_{solv}) for T = 298.15 K in acetonitrile were obtained with COSMO-RS.[47,48] Relative values of free enthalpy in solution ($\Delta G(298)_{solv}$), i.e. the barriers and reaction free energies of HAT steps were obtained using the sum of the differences in PW6B95-D3 electronic energies, $G^{RRHO}(298K)$, and G_{solv} as

$$\Delta G(298)_{solv} = \Delta E(\text{PW6B95-D3}) + \Delta G^{rrho}(298K) + \Delta G_{solv} \quad (1)$$

All DFT calculations were performed with the TURBOMOLE program,[49] except the NEB calculations for which ORCA (Version 6)[50] was used. Calculated energies are reported in Table S2.

Results

Reduction of quinoline and phenylquinoline. Figures S6 and S7 summarize the results of the systematic computation of the energetics of the hydrogen atom transfer (HAT) from $Ar_3P(OH)$ radical to quinoline and Ph-quinoline, testing the 8 possible positions of the aromatic rings (except the bridging carbon atoms). The product radical free energies are given in blue, activation free energies - given in red - indicate the very low barrier of addition. We could locate transition structures for all hydrogen transfer reactions to carbon atoms (**TS1-[2-8]** and **TS2-[2-8]**). The most stable product is in both cases the radical with the SOMO fully delocalized over the quinoline rings (**PR1-1** and **PR2-1**).

We were not able to locate transition structures for N-H bond formation. Subsequently, we conducted reaction path optimizations (NEB) starting from a partly optimized reactant complex (r(N-H) = 3.5 Å) and ending with the product complex. The plots of PW6B95-D3 energies along the points of the path (Figure S8) indicate a constant decrease of energy, with a steep drop at a region of the path when electron and proton are transferred simultaneously. The visualization of the spin density in Figure S8 demonstrates the spatial independence of electron transfer (π-to-π) and proton transfer (from O-to-N).

The electronic energies of the transition states **TS1-[2-8]** and **TS2-[2-8]** are slightly higher than the energy of the path for H transfer to N in the ET/PT region. We therefore conclude that there is a kinetic preference for hydrogen transfer leading to the N-H intermediates **PR1-1** and **PR1-2**, which are also the thermodynamically preferred intermediates.

Figure S9 shows the spin density of all transition structures of hydrogen transfer to carbon atoms of the quinolines. Apparently, the electron has been transferred almost entirely to the quinoline fragment before the O-H bond starts to disappear.

Racemization of phenyl tetrahydroquinoline (PhTHQ). We calculated the free energy barrier for hydrogen atom transfer from Ph-THQ (**2a**) by triphenyl phosphine radical cation (Figure S10, Table S3). The atom transfer occurs via a low barrier (1.8 kcal/mol) with the spin density predominantly located on the tetrahydroquinoline moiety. The reaction is moderately exothermic (-11.5 kcal/mol).

Subsequent hydrogen atom transfer from the $Ph_3PH^{\cdot}$ radical is considerably more exergonic (ΔG298 = -56.0 kcal/mol). We were not able to locate a transition structure on the singlet or triplet potential energy surface and assume that this reaction (formally a radical recombination) occurs without an intrinsic enthalpic barrier.

Figure S6 DFT calculated activation free energies and reaction free energies of the hydrogen atom transfer from $Ar_3P(OH)$ radical to quinoline

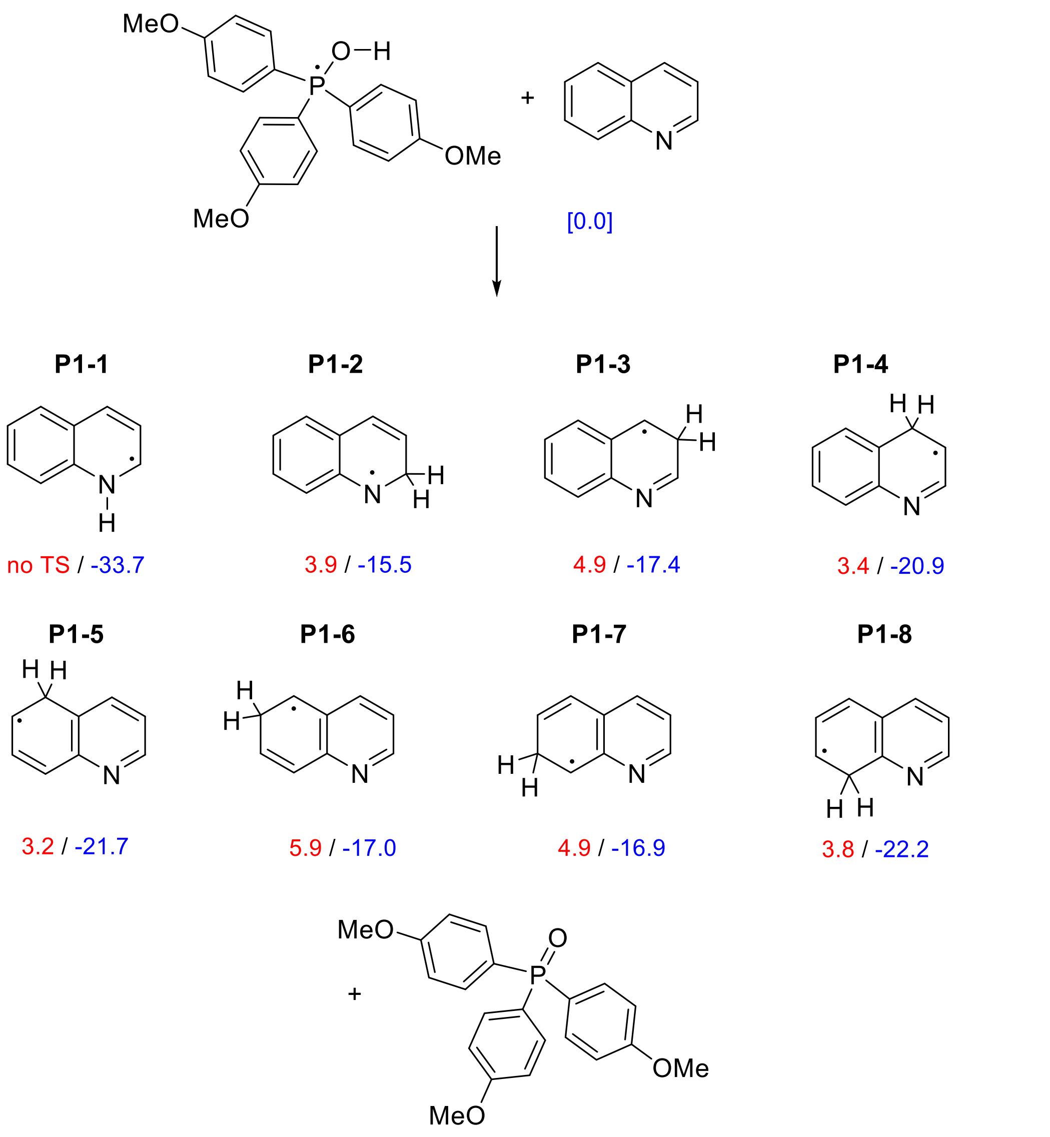

$\Delta G^{\ddagger}_{298}$ / ΔG_{298} (solv) [kcal/mol] (PW6B95-D3//PBE0-D3/def2-TZVP + COSMO-RS(CH_3CN))

Figure S7 DFT calculated activation free energies and reaction free energies of the hydrogen atom transfer from $Ar_3P(OH)$ radical to Ph-quinoline

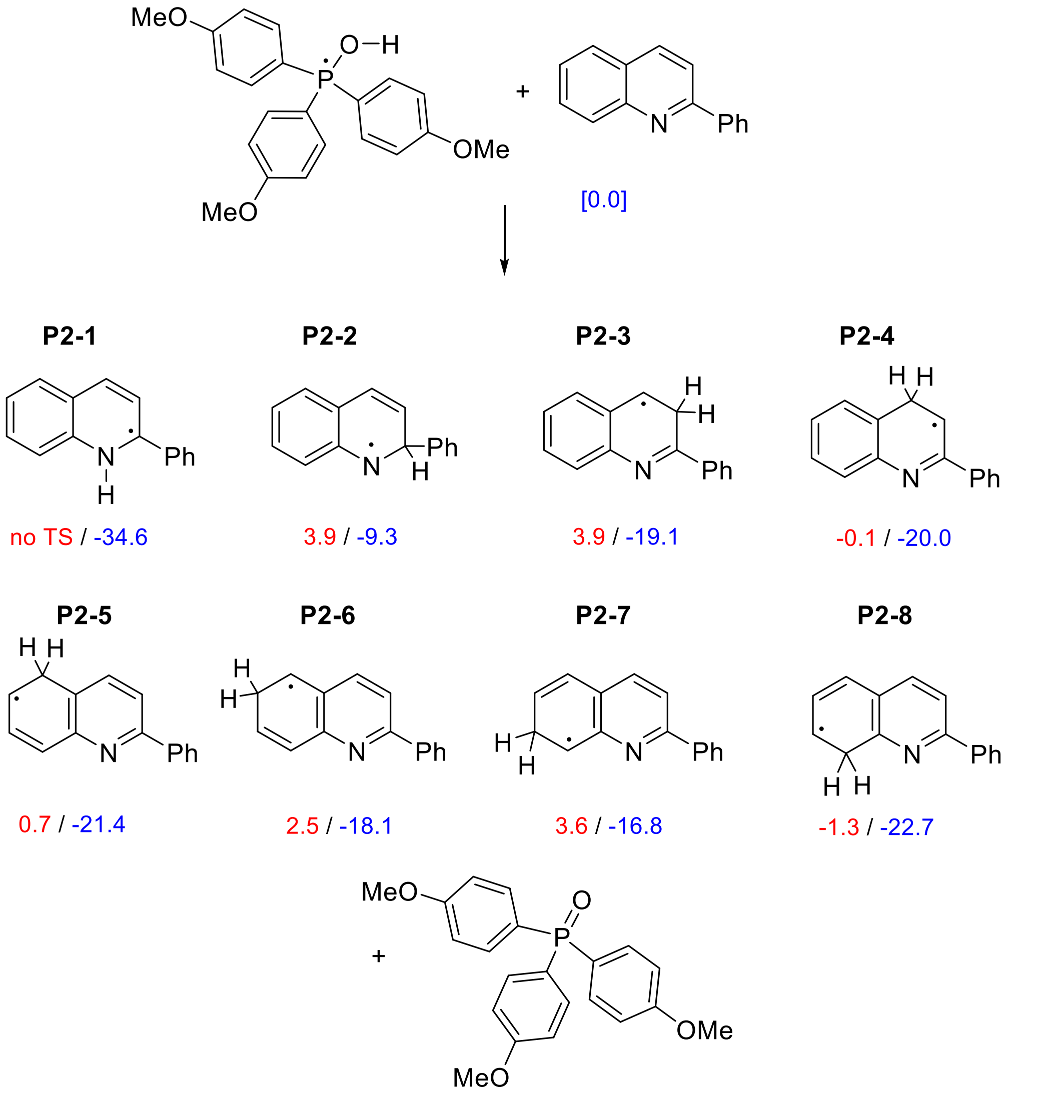

$\Delta G^{\ddagger}_{298}$ / ΔG_{298} (solv) [kcal/mol] (PW6B95-D3//PBE0-D3/def2-TZVP + COSMO-RS(CH_3CN))

Table S2: Relative energies of molecular species in the reactions of (Ph-)quinoline, as calculated with DFT[a] for T = 298.15 K. $\Delta G(298)_{solv} = \Delta E(PW6B95\text{-}D3//TPSS\text{-}D3) + \Delta G^{RRHO}_{298} + \Delta G^{solv}_{298}$

Species	E(PBE0-D3) [E_h]	G^{RRHO}_{298} [kcal/mol]	E(PW6B95-D3) [E_h]	G^{solv}_{298} (CH_3CN) [kcal/mol]	**ΔE_{vac} (PW6B95-D3)** [kcal/mol]	**$\Delta G(298)_{solv}$ (CH_3CN)[b] [kcal/mol]**
(pOMe-Ph)$_3$POH(.)	-1454.733077	209.257	-1457.827367	-17.823		
(pOMe-Ph)$_3$P=O	-1454.211067	204.716	-1457.305835	-18.334		
Quinoline	-401.610315	65.957	-402.609283	-4.301	0.0	0.0
Ph-Quinoline	-632.487977	113.117	-634.066682	-8.162	0.0	0.0
**PR1-1**	-402.185763	71.615	-403.182377	-6.260	-32.4	-33.7
**PR1-2**	-402.158393	70.964	-403.155265	-4.385	-15.3	-15.5
**PR1-3**	-402.161731	70.765	-403.157884	-4.430	-17.0	-17.4
**PR1-4**	-402.167239	71.068	-403.163750	-4.601	-20.7	-20.9
**PR1-5**	-402.169095	71.065	-403.165234	-4.467	-21.6	-21.7
**PR1-6**	-402.161416	70.914	-403.157576	-4.383	-16.8	-17.0
**PR1-7**	-402.161181	70.997	-403.157431	-4.470	-16.7	-16.9
**PR1-8**	-402.170764	70.998	-403.166864	-3.880	-22.6	-22.2
**TS1-2**	-1856.364861	288.961	-1860.451521	-22.685	-9.3	3.9
**TS1-3**	-1856.361582	288.600	-1860.448314	-23.292	-7.3	4.9
**TS1-4**	-1856.364843	289.710	-1860.453545	-22.646	-10.6	3.4
**TS1-5**	-1856.362271	288.295	-1860.449670	-23.785	-8.2	3.2
**TS1-6**	-1856.359090	287.876	-1860.445870	-23.110	-5.8	5.9
**TS1-7**	-1856.363340	288.831	-1860.450515	-22.165	-8.7	4.9
**TS1-8**	-1856.364776	288.858	-1860.451547	-22.658	-9.3	3.8
**PR2-1**	-633.067028	119.251	-634.643507	-9.152	-34.7	-34.6
**PR2-2**	-633.026057	118.205	-634.602373	-8.575	-8.9	-9.3
**PR2-3**	-633.042910	118.165	-634.618948	-7.969	-19.3	-19.1
**PR2-4**	-633.043318	118.181	-634.619601	-8.528	-19.7	-20.0
**PR2-5**	-633.046356	118.227	-634.622221	-8.313	-21.3	-21.4
**PR2-6**	-633.040652	118.100	-634.616723	-8.289	-17.9	-18.1
**PR2-7**	-633.038644	118.154	-634.614656	-8.341	-16.6	-16.8
**PR2-8**	-633.049145	118.143	-634.625048	-7.703	-23.1	-22.7
**TS2-2**	-2087.244265	336.374	-2091.910008	-26.096	-10.0	3.9
**TS2-3**	-2087.243813	336.271	-2091.910233	-25.842	-10.2	3.9
**TS2-4**	-2087.250855	337.160	-2091.918623	-25.480	-15.4	-0.1
**TS2-5**	-2087.249176	336.731	-2091.915559	-26.130	-13.5	0.7

**TS2-6**	-2087.246825	336.794	-2091.913135	-25.970	-12.0	2.5
**TS2-7**	-2087.245902	336.620	-2091.912488	-25.054	-11.6	3.6
**TS2-8**	-2087.254703	337.064	-2091.921037	-25.020	-16.9	-1.3

[a] all calculations were performed with the def2-TZVP basis set
[b] relative free energies refer to (pOMe-Ph)$_3$POH and quinoline / Ph-quinoline, respectively.

Figure S8 Calculated ground state reaction paths for hydrogen transfer from P-OH to the N atom (starting with fixed r(N-H) = 3.5 Å as point # 0) of quinoline (**a**) and Ph-quinoline (**b**). NEB paths (20 points) were calculated with PBEh-3c, electronic energies were recalculated with PW6B95-D3 using the optimized NEB structures. Relative energies are given in kcal/mol with respect to the isolated reactants (Ar$_3$POH and (Ph-)quinoline). Gray dots indicate electronic energies of the seven transition structures of hydrogen transfer to carbon atoms no. 2-8 of the quinoline ring.

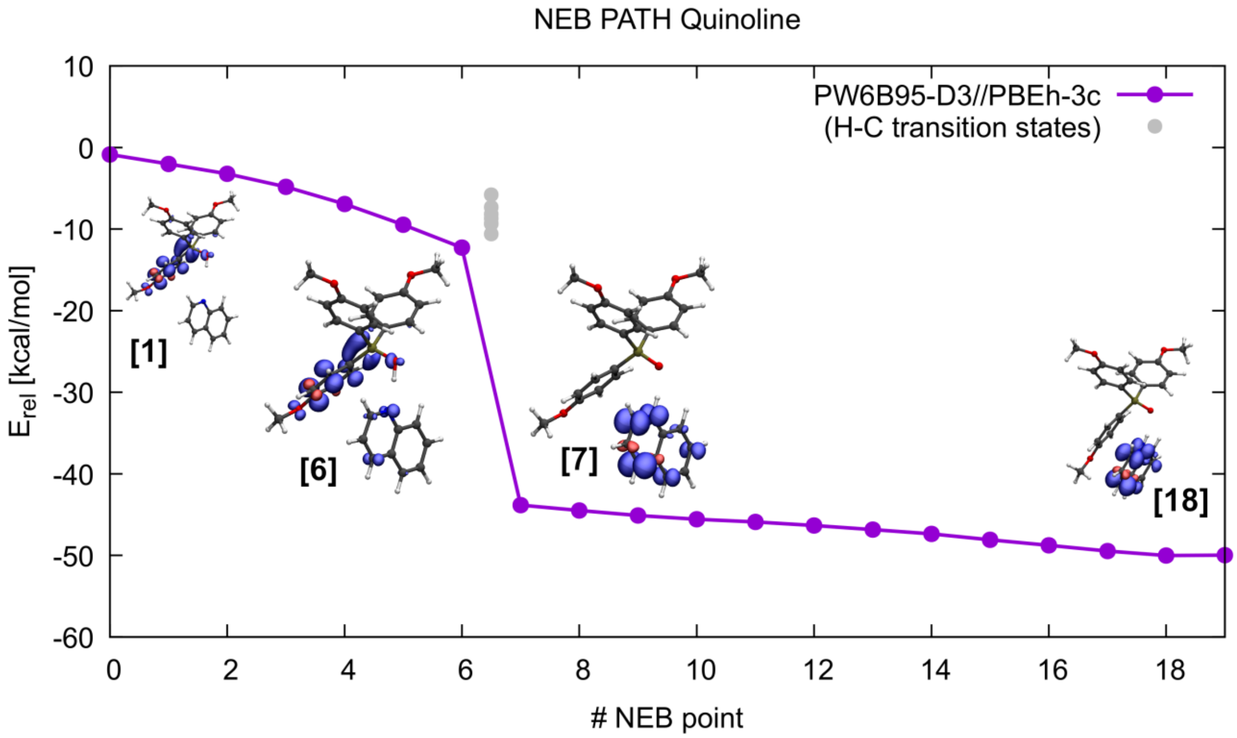

(a)

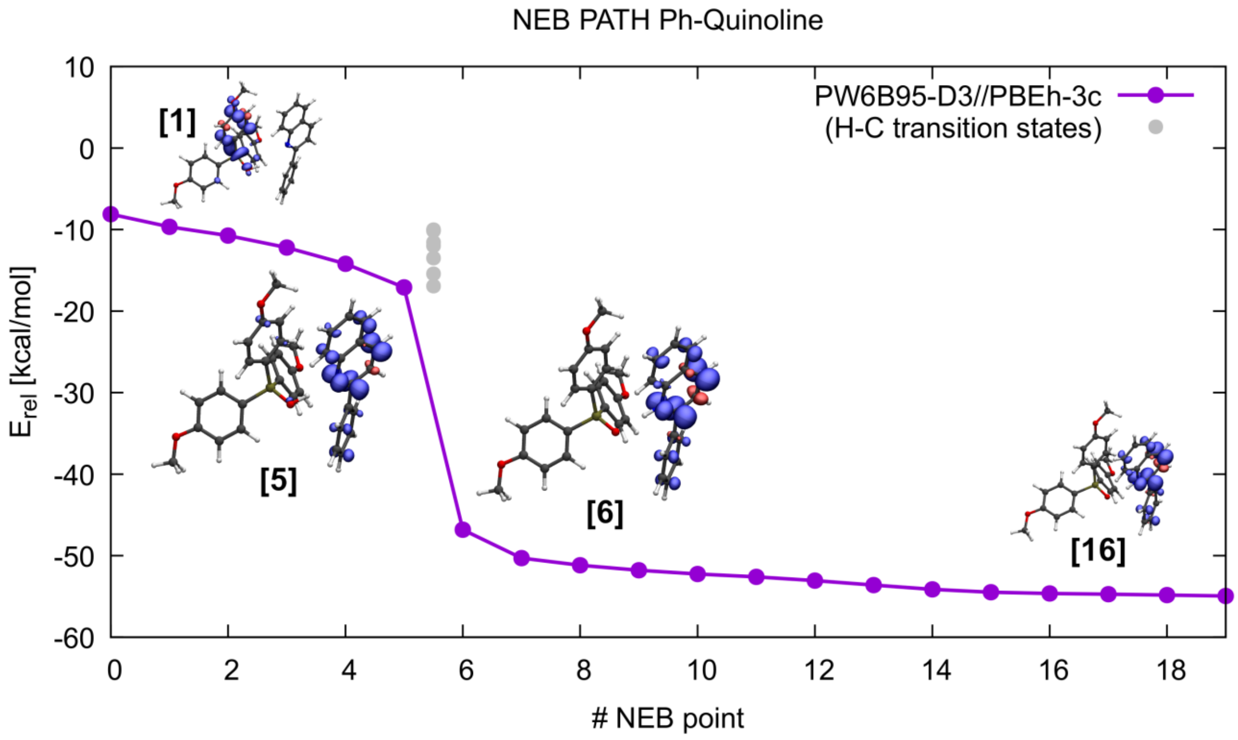
NEB PATH Ph-Quinoline
PW6B95-D3//PBEh-3c
(H-C transition states)
E_{rel} [kcal/mol]
NEB point
[1]
[5]
[6]
[16]
10
0
-10
-20
-30
-40
-50
-60
0
2
4
6
8
10
12
14
16
18

(b)

Figure S9 Molecular structures of HAT transition states optimized with PBE0-D3/def2-TZVP. Spin density (ρ_α-ρ_β) calculated with PW6B95/def2-TZVP with an isosurface value of ±0.005 a.u.

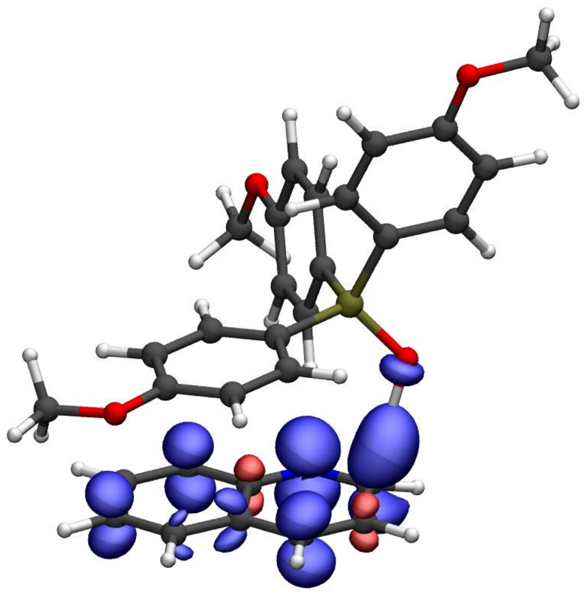

TS1-2

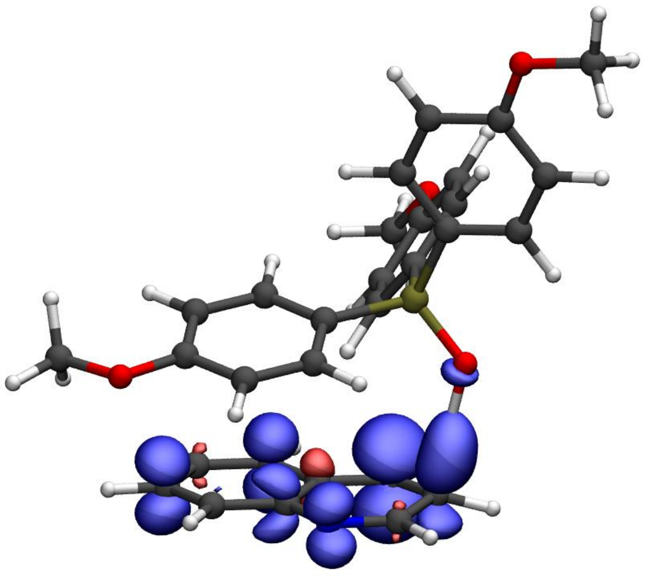

TS1-3

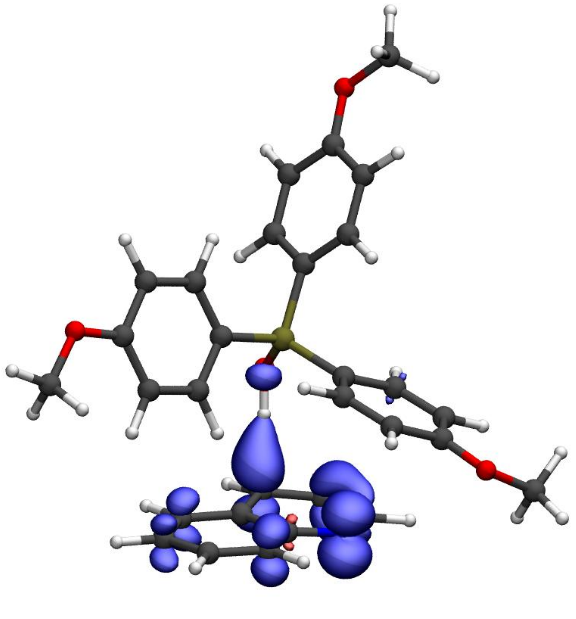

TS1-4

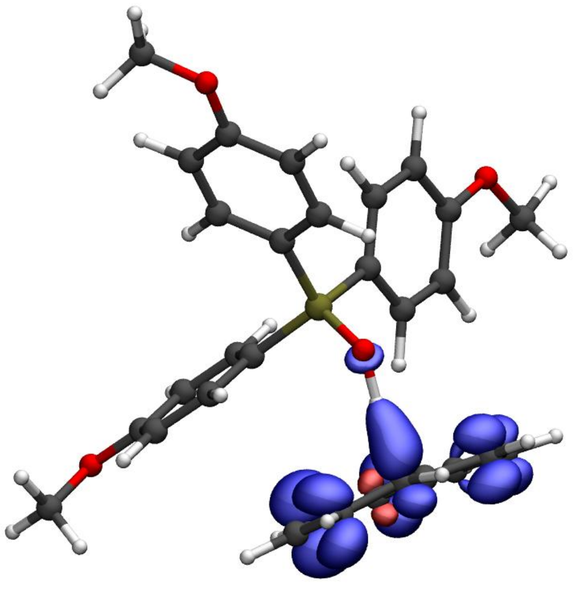

TS1-5

Figure S9 (continued) Molecular structures of HAT transition states optimized with PBE0-D3/def2-TZVP. Spin density (ρ_α-ρ_β) calculated with PW6B95/def2-TZVP with an isosurface value of ±0.005 a.u.

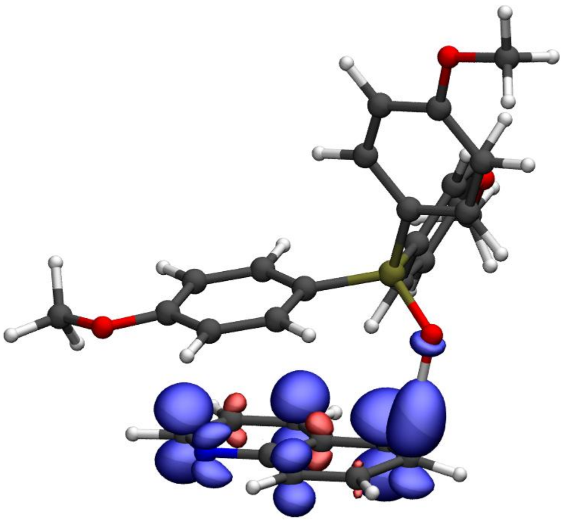

TS1-6

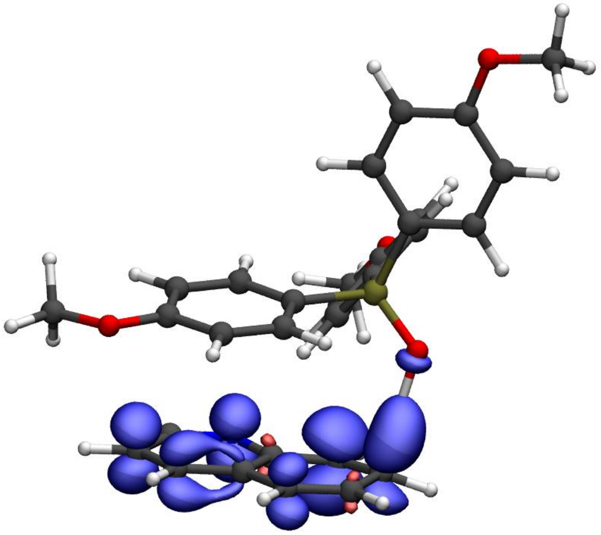

TS1-7

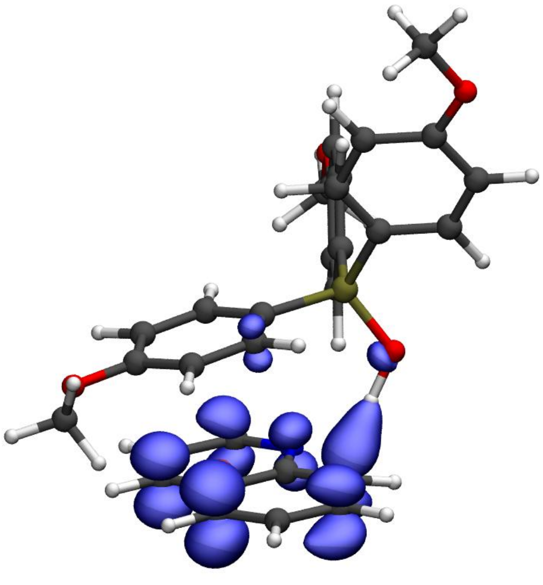

TS1-8

Figure S9 (continued) Molecular structures of HAT transition states optimized with PBE0-D3/def2-TZVP. Spin density (ρ_α-ρ_β) calculated with PW6B95/def2-TZVP with an isosurface value of ±0.005 a.u.

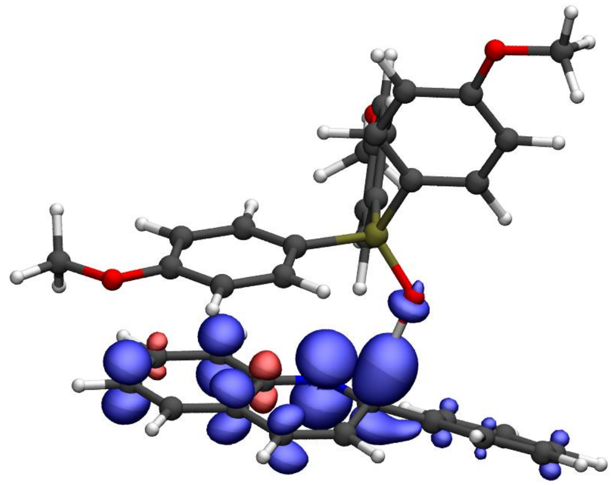

TS2-2

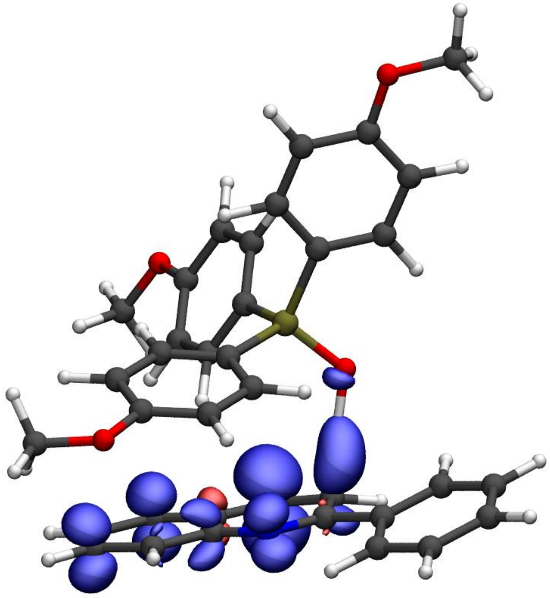

TS2-3

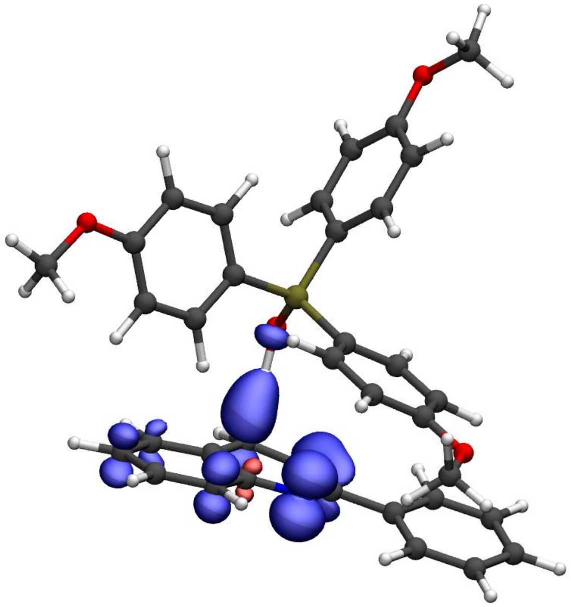

TS2-4

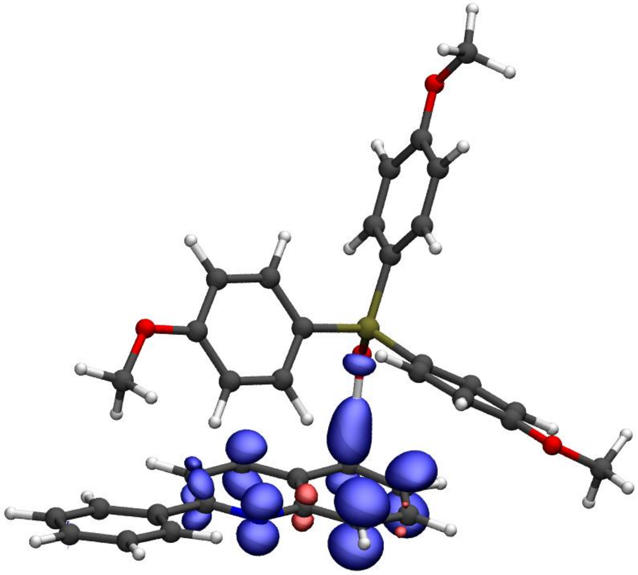

TS2-5

Figure S9 (continued) Molecular structures of HAT transition states optimized with PBE0-D3/def2-TZVP. Spin density (ρ_α-ρ_β) calculated with PW6B95/def2-TZVP with an isosurface value of ±0.005 a.u.

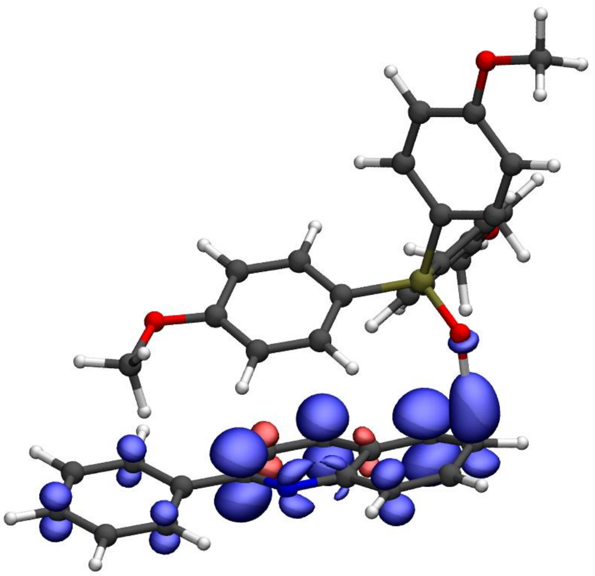

TS2-6

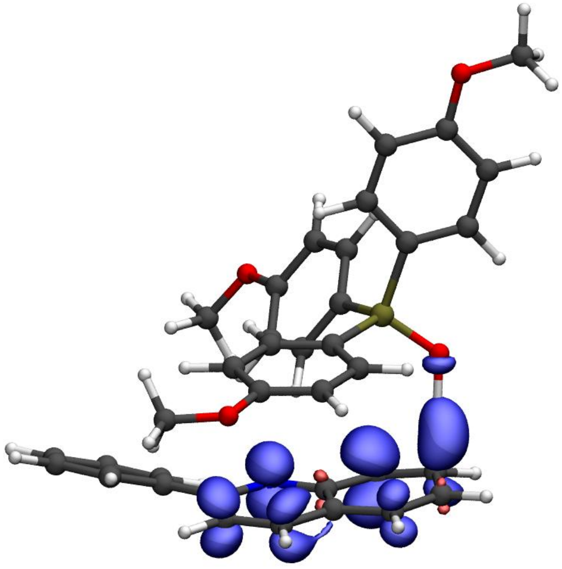

TS2-7

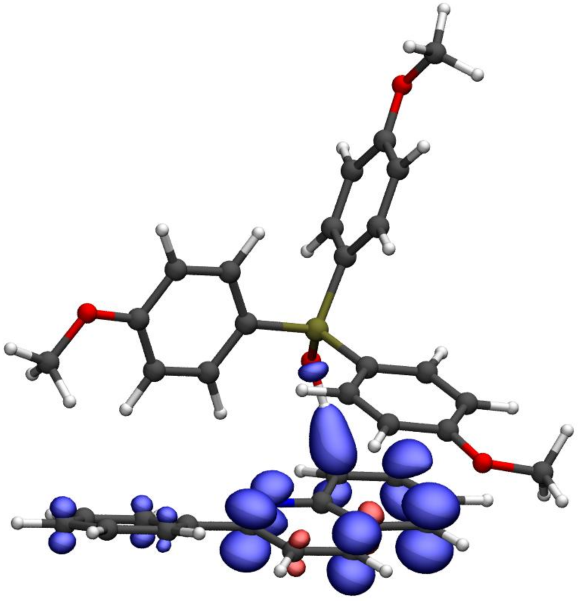

TS2-8

Figure S10 DFT-calculated pathway for epimerization (racemization) of **PhTHQ** (**2a**)

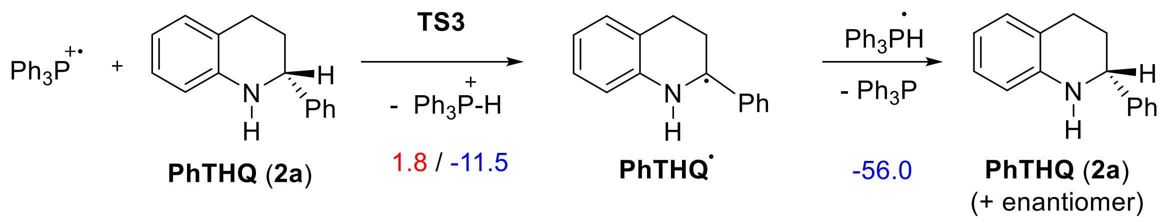

$\Delta G^{\ddagger}_{298}$ / ΔG_{298} (solv) [kcal/mol] (PW6B95-D3//PBE0-D3/def2-TZVP + COSMO-RS(CH_3CN))

Figure S11 Molecular structure of HAT transition state **TS3** optimized with PBE0-D3/def2-TZVP. Spin density (ρ_{α}-ρ_{β}) calculated with PW6B95/def2-TZVP with an isosurface value of ±0.005 a.u.

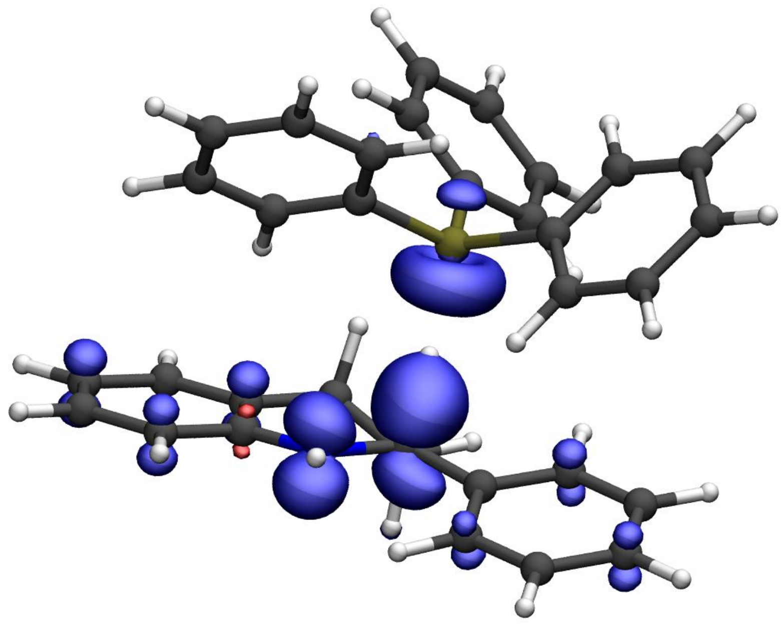

TS3

Table S3: Relative energies of molecular species in the reactions of (Ph-)quinoline (**2a**), as calculated with DFT[a] for T = 298.15 K. $\Delta G(298)_{solv} = \Delta E(PW6B95\text{-}D3//TPSS\text{-}D3) + \Delta G^{RRHO}_{298} + \Delta G^{solv}_{298}$

Species	E(PBE0-D3) [E_h]	G^{RRHO}_{298} [kcal/mol]	E(PW6B95-D3) [E_h]	G^{solv}_{298} (CH_3CN) [kcal/mol]	**$\Delta G(298)_{solv}$ (CH_3CN) [kcal/mol]**
$Ph_3P^{\cdot+}$	-1035.383126	146.507	-1037.547629	-37.256	
PhTHQ (**2a**)	-634.894973	141.677	-636.473247	-7.760	(0.0)
**TS3**	-1670.307246	299.983	-1674.044062	-40.514	+1.8
$PhTHQ^{\cdot}$ (radical)	-634.266822	133.549	-635.842957	-8.643	-11.5
Ph_3PH^+	-1036.022582	152.478	-1038.188323	-39.169	
$Ph_3PH^{\cdot}$	-1036.168177	149.663	-1038.333863	-9.210	
Ph_3P	-1035.635020	146.501	-1037.801400	-9.669	

[a] all calculations were performed with the def2-TZVP basis set

DFT optimized (PBE0-D3/def2-TZVP) cartesian coordinates

(pOMe-Ph)3POH(.)

```
E(PBE0-D3/def2-TZVP) = -1454.733077432 (conv) Lowest Freq. =    19.71 cm^-1

48

(pOMe-Ph)3POH(.) (053c1/opt)

P     1.1233471   -0.1298920   -0.0521003
C     0.6645890    1.3349228    0.8354575
C     1.3680507    2.5357125    0.6193588
H     2.2672142    2.5279084    0.0154303
C     0.9282979    3.7256067    1.1604557
H     1.4713309    4.6480365    0.9922369
C    -0.2209362    3.7634128    1.9449895
C    -0.9422345    2.5846293    2.1583676
H    -1.8502661    2.5869638    2.7462089
C    -0.5121527    1.4011672    1.5932485
H    -1.1071920    0.5066239    1.7316081
C     0.5718853   -1.5558852    0.7619883
C     0.4049334   -2.7750481    0.0459028
H     0.5771062   -2.7957180   -1.0231922
C     0.0228477   -3.9267345    0.6829465
H    -0.1164494   -4.8453277    0.1248758
C    -0.1818657   -3.9503146    2.0680686
C     0.0407002   -2.7847556    2.8040330
H    -0.0691305   -2.7796313    3.8807199
C     0.4268799   -1.6211256    2.1713895
H     0.6247854   -0.7432045    2.7739963
C     0.6402549   -0.1646770   -1.7714144
C    -0.6633938   -0.5674793   -2.0932578
H    -1.3053701   -0.9604109   -1.3127018
C    -1.1385332   -0.4715990   -3.3788508
H    -2.1404889   -0.7948638   -3.6332245
C    -0.3325499    0.0695961   -4.3867834
C     0.9536935    0.4959517   -4.0819752
H     1.5953745    0.9184926   -4.8433115
C     1.4346527    0.3696027   -2.7844289
```

```
H     2.4454275    0.6833817   -2.5609553
O     2.7429152    0.0632263   -0.2969020
H     3.1995150    0.1333396    0.5492185
O    -0.5668503    4.9708008    2.4465449
O    -0.5579554   -5.1422260    2.5991094
O    -0.8915142    0.1390161   -5.6132032
C    -1.7296198    5.0475789    3.2330362
H    -1.8242091    6.0889160    3.5356441
H    -1.6530076    4.4187376    4.1268423
H    -2.6195170    4.7560825    2.6646436
C    -0.7471349   -5.2027754    3.9878812
H     0.1770736   -4.9755179    4.5319180
H    -1.0508546   -6.2240457    4.2124840
H    -1.5327293   -4.5134254    4.3188849
C    -0.1215705    0.6839402   -6.6569453
H     0.1567431    1.7225609   -6.4488104
H    -0.7482646    0.6513761   -7.5462124
H     0.7861726    0.0970736   -6.8332200
```

(pOMe-Ph)3P=O

```
E(PBE0-D3/def2-TZVP) = -1454.211067460 (conv) Lowest Freq. =    18.41 cm^-1
47
(pOMe-Ph)3P=O (061c1/opt)
P     1.2429435   -0.0611013   -0.0226846
C     0.6222379    1.3537838    0.9058321
C    -0.5721451    2.0115511    0.6098263
H    -1.1642811    1.6997901   -0.2434777
C    -1.0034651    3.0710057    1.3806161
H    -1.9264430    3.5919004    1.1565128
C    -0.2405927    3.5019003    2.4659699
C     0.9606423    2.8633114    2.7638224
H     1.5747943    3.1896393    3.5920523
C     1.3801113    1.7976013    1.9827392
H     2.3225748    1.3069344    2.1994461
C     0.4767874   -1.5094781    0.7284925
C     1.1366254   -2.7254800    0.5983432
```

H	2.0933926	-2.7517289	0.0886332
C	0.6021337	-3.8911045	1.1252790
H	1.1417064	-4.8216416	1.0130839
C	-0.6168879	-3.8450306	1.7970510
C	-1.2820071	-2.6275451	1.9425414
H	-2.2208666	-2.6111620	2.4826651
C	-0.7366448	-1.4749929	1.4165278
H	-1.2534332	-0.5317378	1.5549249
C	0.5152823	0.0776541	-1.6660274
C	-0.7394147	-0.4310118	-2.0034763
H	-1.3179231	-0.9770215	-1.2665169
C	-1.2493078	-0.2644865	-3.2742345
H	-2.2200274	-0.6596434	-3.5481370
C	-0.5067858	0.4118434	-4.2422189
C	0.7530018	0.9121578	-3.9227250
H	1.3516243	1.4276435	-4.6613417
C	1.2513672	0.7407949	-2.6404654
H	2.2385376	1.1133006	-2.3901253
O	2.7239488	-0.1350566	-0.0476119
O	-0.7388376	4.5452466	3.1608142
O	-1.2231638	-4.9181153	2.3452568
O	-1.0832732	0.5244790	-5.4565439
C	0.0009387	5.0205098	4.2601834
H	-0.5699090	5.8495618	4.6739519
H	0.9885093	5.3800005	3.9528621
H	0.1213991	4.2472395	5.0260953
C	-0.5842351	-6.1674162	2.2320729
H	-0.4611761	-6.4607088	1.1842739
H	-1.2316579	-6.8852924	2.7319909
H	0.3950006	-6.1620624	2.7219948
C	-0.3666651	1.1903869	-6.4688727
H	-0.1606250	2.2305247	-6.1956221
H	-1.0014245	1.1705071	-7.3526759
H	0.5776334	0.6825498	-6.6910980

Quinoline

```
E(PBE0-D3/def2-TZVP) = -401.6103148113 (conv) Lowest Freq. =   173.24 cm^-1

17

Quinoline (198c1/opt)

H     1.6330327    2.0615459    0.0000000

C     1.5415060   -2.0081916    0.0000000

C     0.2299285   -2.5258861    0.0000000

N    -0.8502813   -1.7903475    0.0000000

C    -0.7000352   -0.4429166    0.0000000

C     0.5761714    0.1823932    0.0000000

C     1.7106088   -0.6536158    0.0000000

C     0.6552782    1.5911996    0.0000000

C    -0.4832042    2.3483792    0.0000000

C    -1.7486927    1.7289088    0.0000000

C    -1.8566713    0.3656490    0.0000000

H     2.3879056   -2.6839921    0.0000000

H     0.0805935   -3.6032317    0.0000000

H     2.7007977   -0.2096141    0.0000000

H    -0.4162646    3.4300190    0.0000000

H    -2.6422454    2.3424249    0.0000000

H    -2.8184277   -0.1327241    0.0000000
```

Ph-Quinoline

```
E(PBE0-D3/def2-TZVP) = -632.4879765957 (conv) Lowest Freq. =    40.98 cm^-1

27

Ph-Quinoline (199c1/opt)

H     0.4542908    3.5718373    2.8060061

C     0.4458983    1.4442235   -0.6686640

C     0.0831099    0.0818939   -0.5040754

N    -0.1999932   -0.4260199    0.6739536

C    -0.1618734    0.3701328    1.7646260

C     0.1760298    1.7474637    1.6931397

C     0.4888988    2.2626603    0.4194491

C     0.1936680    2.5205966    2.8713409

C    -0.1131195    1.9509726    4.0769505

C    -0.4486646    0.5848528    4.1504665
```

```
C    -0.4733232   -0.1894057    3.0230697
H     0.7106542    1.8210902   -1.6474174
H     0.7713438    3.3057075    0.3199217
H    -0.0989892    2.5490937    4.9804314
H    -0.6896352    0.1465732    5.1120973
H    -0.7306044   -1.2410816    3.0575265
C     0.0276065   -0.8329866   -1.6638521
C    -0.1267730   -0.3603225   -2.9663410
C     0.1232912   -2.2095236   -1.4596040
C     0.0783231   -3.0859461   -2.5287767
C    -0.1791145   -1.2389316   -4.0365279
C    -0.0718695   -2.6045137   -3.8224723
H     0.2324424   -2.5715871   -0.4453453
H    -0.2349322    0.7016502   -3.1511508
H     0.1617334   -4.1524070   -2.3535128
H    -0.3095594   -0.8543665   -5.0414167
H    -0.1088390   -3.2916565   -4.6598223
```

PR1-1

```
E(PBE0-D3/def2-TZVP) = -402.1857628701 (conv) Lowest Freq. =   142.94 cm^-1
18
PR1-1 (189c1/opt)
H     1.5888595    2.2648387    0.0000000
C     1.7448365   -1.8328137    0.0000000
C     0.5279327   -2.4522704    0.0000000
N    -0.6205792   -1.6965687    0.0000000
C    -0.6107463   -0.3156736    0.0000000
C     0.6443345    0.3446659    0.0000000
C     1.8335496   -0.4389602    0.0000000
C     0.6366501    1.7455774    0.0000000
C    -0.5472407    2.4639316    0.0000000
C    -1.7641643    1.7976815    0.0000000
C    -1.7940013    0.4081266    0.0000000
H     2.6380920   -2.4440506    0.0000000
H     0.3967326   -3.5241410    0.0000000
H     2.7954308    0.0569512    0.0000000
```

```
H    -1.5117202   -2.1594504    0.0000000
H    -0.5213149    3.5470990    0.0000000
H    -2.6936107    2.3539456    0.0000000
H    -2.7430409   -0.1188888    0.0000000
```

PR1-2

```
E(PBE0-D3/def2-TZVP) = -402.1583934332 (conv) Lowest Freq. =   117.14 cm^-1
18
PR1-2 (186c1/opt)
H     1.4705477    2.2866855    0.2769544
C     1.6882559   -1.7238665   -0.0641303
C     0.3848084   -2.4261814   -0.1948263
N    -0.8121505   -1.6437597   -0.1866025
C    -0.7128931   -0.3273038   -0.0651855
C     0.5319580    0.3797804    0.0612074
C     1.7445545   -0.3940048    0.0562527
C     0.5246018    1.7629597    0.1821347
C    -0.6646457    2.4744286    0.1832206
C    -1.8867353    1.7989573    0.0613830
C    -1.9151873    0.4337997   -0.0597423
H     2.5911685   -2.3257905   -0.0699760
H     0.3801977   -3.0341074   -1.1160086
H     2.6931308    0.1244867    0.1518148
H     0.2912041   -3.1886332    0.5975742
H    -0.6498074    3.5535285    0.2784384
H    -2.8132648    2.3614255    0.0629812
H    -2.8457432   -0.1124047   -0.1554899
```

PR1-3

```
E(PBE0-D3/def2-TZVP) = -402.1617310220 (conv) Lowest Freq. =   103.77 cm^-1
18
PR1-3 (187c1/opt)
H     1.3916976    2.3005188    0.0000000
C     1.6148128   -1.8671606    0.0000000
C     0.2218300   -2.4090532    0.0000000
N    -0.8547207   -1.7310591    0.0000000
```

C   -0.7806383   -0.3392290   0.0000000

C   0.4697515   0.3503180   0.0000000

C   1.6488499   -0.3872656   0.0000000

C   0.4475626   1.7659324   0.0000000

C   -0.7426458   2.4536075   0.0000000

C   -1.9592154   1.7639315   0.0000000

C   -1.9669423   0.3765941   0.0000000

H   2.1561182   -2.2852493   -0.8652517

H   0.1183816   -3.4952194   0.0000000

H   2.6061557   0.1218411   0.0000000

H   2.1561182   -2.2852493   0.8652517

H   -0.7384371   3.5375609   0.0000000

H   -2.8936256   2.3118211   0.0000000

H   -2.8950529   -0.1826400   0.0000000

PR1-4

E(PBE0-D3/def2-TZVP) = -402.1672388632 (conv) Lowest Freq. =   105.88 cm^-1

18

PR1-4 (188c1/opt)

H   1.3732309   2.1821821   -0.1931218

C   1.4969586   -1.9740104   -0.1714335

C   0.2440013   -2.5032080   -0.0076308

N   -0.8811025   -1.7971630   0.1262133

C   -0.7788934   -0.4290225   0.1012279

C   0.4397420   0.2628783   -0.0597123

C   1.7108051   -0.5110311   -0.2164348

C   0.4348881   1.6497904   -0.0695061

C   -0.7449748   2.3617955   0.0759270

C   -1.9511960   1.6833700   0.2350058

C   -1.9663334   0.3060738   0.2471189

H   2.3514424   -2.6331866   -0.2718766

H   0.1219668   -3.5832119   0.0196544

H   2.2064946   -0.2336933   -1.1597787

H   2.4316922   -0.2127396   0.5604035

H   -0.7268536   3.4452687   0.0658117

H   -2.8751134   2.2381122   0.3490559

H   -2.8867549   -0.2522045   0.3690762

PR1-5

E(PBE0-D3/def2-TZVP) = -402.1690954924 (conv) Lowest Freq. =   86.08 cm^-1

18

PR1-5 (182c1/opt)

H   -0.4977049   2.2065858   0.8588738

C   0.4134325   0.1456799   -2.5716845

C   0.0591360   -1.1854411   -2.3753407

N   -0.2394162   -1.6973561   -1.1966528

C   -0.1993238   -0.8874252   -0.1221583

C   0.1466886   0.4767511   -0.2149997

C   0.4540303   0.9797262   -1.4672589

C   0.1781459   1.3499956   1.0057578

C   -0.1705142   0.6541371   2.2721350

C   -0.4978820   -0.6706412   2.3054491

C   -0.5225950   -1.4534752   1.1485202

H   0.6492111   0.5113321   -3.5632787

H   0.0163061   -1.8687601   -3.2197066

H   0.7253612   2.0254070   -1.5777593

H   1.1734337   1.8106295   1.0999976

H   -0.1592678   1.2372092   3.1858086

H   -0.7460817   -1.1310472   3.2557870

H   -0.7829596   -2.5033074   1.1765101

PR1-6

E(PBE0-D3/def2-TZVP) = -402.1614158351 (conv) Lowest Freq. =   124.04 cm^-1

18

PR1-6 (183c1/opt)

H   1.6271685   1.9448904   0.0000000

C   1.6529613   -2.1224036   0.0000000

C   0.3713016   -2.6793481   0.0000000

N   -0.7419736   -1.9528766   0.0000000

C   -0.6323908   -0.6296560   0.0000000

C   0.6257280   0.0538113   0.0000000

C   1.7843634   -0.7557282   0.0000000

C	0.6642382	1.4445189	0.0000000
C	-0.5610055	2.2791710	0.0000000
C	-1.8255035	1.4921875	0.0000000
C	-1.8464732	0.1559987	0.0000000
H	2.5228843	-2.7681663	0.0000000
H	0.2416006	-3.7573924	0.0000000
H	2.7622146	-0.2858682	0.0000000
H	-0.5547299	2.9649413	0.8641578
H	-0.5547299	2.9649413	-0.8641578
H	-2.7572375	2.0484953	0.0000000
H	-2.7784164	-0.3975163	0.0000000

PR1-7

E(PBE0-D3/def2-TZVP) = -402.1611806782 (conv) Lowest Freq. = 123.58 cm^-1

18

PR1-7 (184c1/opt)

H	1.6678145	2.0233617	-0.0270613
C	1.7471190	-2.0624362	-0.0364844
C	0.4713689	-2.6379000	-0.0137681
N	-0.6499739	-1.9535978	0.0079048
C	-0.5681086	-0.5964091	0.0090548
C	0.6889556	0.0834185	-0.0127797
C	1.8445197	-0.6811756	-0.0359913
C	0.7021583	1.5271684	-0.0101015
C	-0.4241355	2.2469799	0.0124743
C	-1.7743367	1.6191929	0.0364389
C	-1.7505779	0.1382005	0.0325304
H	2.6283312	-2.6910931	-0.0539543
H	0.3707829	-3.7208575	-0.0138276
H	2.8128522	-0.1913152	-0.0533893
H	-2.3369428	1.9862459	0.9117366
H	-0.3758683	3.3307673	0.0137337
H	-2.3694911	1.9903263	-0.8152781
H	-2.6844673	-0.4108769	0.0487620

PR1-8

E(PBE0-D3/def2-TZVP) = -402.1707642975 (conv) Lowest Freq. = 95.03 cm^-1

18

PR1-8 (185c1/opt)

H	1.6514994	2.1311548	0.0000000
C	1.7823415	-1.9527868	0.0000000
C	0.5220877	-2.5389705	0.0000000
N	-0.6071693	-1.8384465	0.0000000
C	-0.5337310	-0.5152589	0.0000000
C	0.6918784	0.1868537	0.0000000
C	1.8653383	-0.5772758	0.0000000
C	0.6994449	1.6133722	0.0000000
C	-0.4990317	2.3333291	0.0000000
C	-1.7118660	1.7075044	0.0000000
C	-1.8372319	0.2271691	0.0000000
H	2.6724682	-2.5699933	0.0000000
H	0.4164043	-3.6199278	0.0000000
H	2.8280576	-0.0766656	0.0000000
H	-2.4278896	-0.1094427	0.8643655
H	-0.4596357	3.4172826	0.0000000
H	-2.6250755	2.2915449	0.0000000
H	-2.4278896	-0.1094427	-0.8643655

TS1-2

E(PBE0-D3/def2-TZVP) = -1856.364860979 (conv) Lowest Freq. = -540.44 cm^-1

65

TS1-2 (178TSc1/opt)

P	0.0295599	-0.7340576	-1.0201210
C	0.2383546	0.9071864	-0.3864115
C	0.9696447	1.8331196	-1.1407759
H	1.4292219	1.5298096	-2.0745203
C	1.1215953	3.1232367	-0.6955362
H	1.6975899	3.8481290	-1.2568621
C	0.5540615	3.5166072	0.5195881
C	-0.1741459	2.6035645	1.2770526
H	-0.5891881	2.8788082	2.2352546

C	-0.3207338	1.3056636	0.8226477
H	-0.8667816	0.5918407	1.4266222
C	-0.8734920	-1.7203202	0.1554073
C	-2.2269989	-2.0168818	-0.0298481
H	-2.7450393	-1.6660539	-0.9147416
C	-2.9114812	-2.7617008	0.9046428
H	-3.9594588	-3.0017899	0.7744249
C	-2.2540738	-3.2279413	2.0449819
C	-0.9037164	-2.9396671	2.2333201
H	-0.3725969	-3.2935175	3.1066278
C	-0.2185545	-2.1880452	1.2940463
H	0.8394200	-1.9610431	1.4551656
C	-0.9333379	-0.6313384	-2.5197570
C	-1.8518616	0.4009516	-2.7231618
H	-1.9568344	1.1826368	-1.9793776
C	-2.6170754	0.4403214	-3.8677845
H	-3.3297420	1.2371880	-4.0408888
C	-2.4757759	-0.5527501	-4.8390591
C	-1.5569049	-1.5833919	-4.6484384
H	-1.4251673	-2.3568157	-5.3924401
C	-0.7954867	-1.6158503	-3.4919997
H	-0.0736479	-2.4114010	-3.3483447
O	1.3808872	-1.3763864	-1.4057704
H	2.2487445	-1.2298804	-0.7268289
O	0.7671156	4.7927128	0.8767070
O	-3.0024198	-3.9456258	2.9019579
O	-3.2604034	-0.4296710	-5.9237270
C	0.2384393	5.2326624	2.1089197
H	0.5554544	6.2676641	2.2197936
H	0.6308690	4.6368768	2.9371914
H	-0.8555700	5.1869974	2.1083451
C	-2.3842906	-4.4403476	4.0688692
H	-1.5640893	-5.1232074	3.8265404
H	-3.1558153	-4.9820835	4.6121561
H	-2.0040899	-3.6265258	4.6941411

C	-3.1504093	-1.4008093	-6.9398359
H	-2.1425016	-1.4189046	-7.3662555
H	-3.8618340	-1.1123996	-7.7107450
H	-3.4051286	-2.3980957	-6.5673958
C	1.8405357	1.4289673	4.0037546
C	2.4788583	2.5703297	3.5227473
C	3.1930182	2.5010963	2.3327222
C	3.2943637	1.3125266	1.6136059
C	2.6537754	0.1281594	2.1093390
C	1.9223154	0.2349714	3.3158874
N	2.7318956	-1.0664754	1.4953771
C	3.4159325	-1.1347764	0.3026592
C	4.1203597	-0.0202326	-0.2156658
C	4.0375821	1.1948601	0.3944989
H	1.2846562	1.4738466	4.9347980
H	2.4395245	3.4973123	4.0837439
H	3.6930940	3.3848868	1.9472507
H	1.4470457	-0.6622153	3.6967080
H	3.7308515	-2.1367785	0.0075243
H	4.6976609	-0.1440345	-1.1270172
H	4.5362197	2.0680817	-0.0117099

TS1-3

E(PBE0-D3/def2-TZVP) = -1856.361582224 (conv) Lowest Freq. = -850.16 cm^-1

65

TS1-3 (179TSc1/opt)

P	0.0175448	-0.5947154	-1.0624435
C	0.2276770	1.0451564	-0.4208163
C	1.1362856	1.8972033	-1.0597716
H	1.7287935	1.5339731	-1.8902807
C	1.3146536	3.1831448	-0.6113917
H	2.0453203	3.8375222	-1.0684871
C	0.5973504	3.6456181	0.4926346
C	-0.3269740	2.8151612	1.1222875
H	-0.8824293	3.1512407	1.9858885
C	-0.5034298	1.5230174	0.6628104

H	-1.2115396	0.8775033	1.1668188
C	-0.8374974	-1.5852431	0.1400110
C	-1.8980652	-2.4196057	-0.2286913
H	-2.2564808	-2.4175626	-1.2513990
C	-2.4968484	-3.2425684	0.6973406
H	-3.3204601	-3.8906881	0.4249817
C	-2.0451391	-3.2532360	2.0208367
C	-0.9933793	-2.4243382	2.4009322
H	-0.6331790	-2.4078282	3.4205480
C	-0.3948919	-1.5976706	1.4635996
H	0.4190133	-0.9544903	1.7743613
C	-0.9876337	-0.5210812	-2.5362374
C	-2.0774776	0.3497458	-2.6135418
H	-2.2947241	1.0176538	-1.7873118
C	-2.8709244	0.3773614	-3.7374519
H	-3.7160943	1.0501766	-3.8139278
C	-2.5877899	-0.4652406	-4.8158087
C	-1.4985071	-1.3304078	-4.7518903
H	-1.2556286	-1.9828643	-5.5791573
C	-0.7069755	-1.3518335	-3.6134939
H	0.1477382	-2.0157979	-3.5640053
O	1.3663758	-1.2078187	-1.4980200
H	2.2637488	-1.1877494	-0.8179244
O	0.8720058	4.8994007	0.8873910
O	-2.6904832	-4.0888283	2.8537704
O	-3.4154410	-0.3653452	-5.8707270
C	0.2485419	5.3770340	2.0582378
H	0.6530527	6.3723565	2.2305711
H	0.4781789	4.7353916	2.9138936
H	-0.8370943	5.4457569	1.9325277
C	-2.2670981	-4.1445703	4.1973748
H	-1.2235313	-4.4657790	4.2729917
H	-2.9061253	-4.8777611	4.6850808
H	-2.3842348	-3.1755201	4.6926983
C	-3.1677805	-1.1845217	-6.9909289

H	-2.1856938	-0.9765382	-7.4271799
H	-3.9418641	-0.9449645	-7.7169032
H	-3.2318736	-2.2458356	-6.7305987
C	2.0112796	1.2308338	4.0260583
C	2.4999627	2.3951901	3.4405858
C	3.1259564	2.3309131	2.1991194
C	3.2693964	1.1258268	1.5214219
C	2.7727871	-0.0802088	2.1178176
C	2.1476557	0.0153373	3.3832541
C	2.9330912	-1.2843413	1.4169531
C	3.4985543	-1.2505131	0.1039787
C	4.0357157	-0.0103570	-0.3224617
N	3.9126330	1.1293801	0.2959219
H	1.5319079	1.2733468	4.9984533
H	2.4135061	3.3438740	3.9583251
H	3.5258424	3.2207034	1.7264619
H	1.7916357	-0.8950779	3.8575325
H	2.6107706	-2.2195525	1.8606755
H	3.8904037	-2.1603919	-0.3442657
H	4.5599104	0.0210229	-1.2790301

TS1-4

E(PBE0-D3/def2-TZVP) = -1856.364843179 (conv) Lowest Freq. = -95.65 cm^-1

65

TS1-4 (180TSc1/opt)

P	-0.2252380	0.9799753	0.2407713
C	-1.5683190	1.9898337	0.8286989
C	-1.4482884	3.3832992	0.8534183
H	-0.5117482	3.8461463	0.5664339
C	-2.5073693	4.1664652	1.2533092
H	-2.4283338	5.2461913	1.2821360
C	-3.7116298	3.5745853	1.6418650
C	-3.8384418	2.1869106	1.6273271
H	-4.7581522	1.7074718	1.9324984
C	-2.7680024	1.4074623	1.2198777
H	-2.8668384	0.3277995	1.2148738

C	-0.2960317	-0.6194406	0.9744173
C	-0.5364470	-1.7814437	0.2283071
H	-0.6816835	-1.7173876	-0.8421096
C	-0.5680228	-3.0070007	0.8427558
H	-0.7194655	-3.9159566	0.2749125
C	-0.3542355	-3.1054919	2.2210926
C	-0.1677929	-1.9540410	2.9817191
H	-0.0066462	-2.0130764	4.0489196
C	-0.1242781	-0.7221559	2.3587055
H	0.0625500	0.1665281	2.9502090
C	-0.3320739	0.8471231	-1.5225693
C	-1.3136918	1.5248594	-2.2547152
H	-2.0525743	2.1284676	-1.7413006
C	-1.3509727	1.4283736	-3.6266042
H	-2.1063472	1.9452459	-4.2054554
C	-0.4023315	0.6542265	-4.3017707
C	0.5777421	-0.0246775	-3.5825680
H	1.3281475	-0.6293371	-4.0722003
C	0.6087409	0.0733083	-2.2031476
H	1.3840493	-0.4607713	-1.6664259
O	1.0615311	1.7566530	0.6861477
H	1.9537644	1.2414037	0.7506742
O	-4.6865222	4.4196285	2.0186303
O	-0.3558369	-4.3496131	2.7253726
O	-0.5149795	0.6227458	-5.6413892
C	-5.9225508	3.8741758	2.4215974
H	-6.5568953	4.7205041	2.6762880
H	-5.8068880	3.2314652	3.2999947
H	-6.3887708	3.3030771	1.6125108
C	0.0334546	-4.5190615	4.0693603
H	1.0303664	-4.1030024	4.2422604
H	0.0533470	-5.5927475	4.2449629
H	-0.6827415	-4.0560962	4.7564475
C	0.4271655	-0.1365511	-6.3661054
H	1.4448895	0.2312641	-6.2028475

H	0.1648803	-0.0204767	-7.4156435
H	0.3784472	-1.1955761	-6.0943881
C	3.8182283	-1.0620853	-2.4617838
C	3.3471776	-2.3380190	-2.1648625
C	2.9984002	-2.6671661	-0.8645651
C	3.1118366	-1.7360595	0.1827542
C	3.5381508	-0.4055397	-0.1372198
C	3.8975408	-0.1064601	-1.4595499
C	3.5658775	0.5498211	0.9250988
C	3.3567608	0.0795359	2.2356173
C	2.9935417	-1.2390217	2.4272724
N	2.8380747	-2.1392942	1.4538341
H	4.1174653	-0.8108589	-3.4735266
H	3.2730894	-3.0851444	-2.9477843
H	2.6595756	-3.6640635	-0.6072840
H	4.2468111	0.8956039	-1.6895176
H	4.0148367	1.5233575	0.7487370
H	3.4587019	0.7428723	3.0870093
H	2.8149963	-1.5987637	3.4385162

TS1-5

E(PBE0-D3/def2-TZVP) = -1856.362270736 (conv) Lowest Freq. = -547.29 cm^-1

65

TS1-5 (174TSc1/opt)

P	-0.1675093	0.9241619	0.2304099
C	-1.5174009	1.9286863	0.8206185
C	-1.3893995	3.3206794	0.8533218
H	-0.4491445	3.7780830	0.5694887
C	-2.4441615	4.1083851	1.2563986
H	-2.3585696	5.1874253	1.2916139
C	-3.6526668	3.5216595	1.6388447
C	-3.7880824	2.1349704	1.6154478
H	-4.7115522	1.6595444	1.9156181
C	-2.7208466	1.3513237	1.2067307
H	-2.8270735	0.2723920	1.1970202
C	-0.2650181	-0.6712578	0.9915041

C	-0.5790459	-1.8297574	0.2721348
H	-0.7212736	-1.7787752	-0.7998034
C	-0.6920229	-3.0389722	0.9138699
H	-0.9096625	-3.9468950	0.3661553
C	-0.4948415	-3.1189707	2.2958239
C	-0.2036364	-1.9699530	3.0259169
H	-0.0431157	-2.0140262	4.0941001
C	-0.0811341	-0.7568304	2.3721107
H	0.1712277	0.1299990	2.9415352
C	-0.3189633	0.7566776	-1.5288657
C	-1.3591158	1.3635825	-2.2407403
H	-2.1121792	1.9384787	-1.7152364
C	-1.4358767	1.2352450	-3.6088439
H	-2.2362943	1.6985379	-4.1723358
C	-0.4686381	0.4992171	-4.2993338
C	0.5697853	-0.1100684	-3.6000435
H	1.3364084	-0.6829246	-4.1022162
C	0.6400452	0.0210491	-2.2246855
H	1.4569899	-0.4597010	-1.7013802
O	1.1235487	1.6826275	0.6400739
H	2.0688861	1.1691585	0.7346913
O	-4.6233581	4.3704399	2.0188220
O	-0.6092689	-4.3465611	2.8331121
O	-0.6211956	0.4329557	-5.6334573
C	-5.8621794	3.8300265	2.4194272
H	-6.4909860	4.6784965	2.6807140
H	-5.7496066	3.1805262	3.2932542
H	-6.3330766	3.2674171	1.6070656
C	-0.3629294	-4.4940843	4.2122937
H	0.6540076	-4.1815760	4.4696728
H	-0.4792960	-5.5541007	4.4283598
H	-1.0803864	-3.9226048	4.8102911
C	0.3369841	-0.2919984	-6.3728275
H	1.3381891	0.1325076	-6.2503501
H	0.0357252	-0.2133557	-7.4153426

```
H     0.3534176   -1.3456131   -6.0775512
C     4.0155082   -0.5814543   -2.6305363
C     3.6029441   -1.8921288   -2.4529126
N     3.2352858   -2.4070869   -1.2823524
C     3.2748267   -1.6056237   -0.1937006
C     3.6400887   -0.2189847   -0.2771282
C     4.0204273    0.2704057   -1.5277894
C     3.5678449    0.5845772    0.9089115
C     3.3829149   -0.0416153    2.1649051
C     3.0685134   -1.3825110    2.2315148
C     2.9753439   -2.1527826    1.0725290
H     4.3199946   -0.2310882   -3.6099515
H     3.5727066   -2.5731928   -3.3013626
H     4.3231604    1.3077443   -1.6272283
H     4.0133987    1.5763278    0.8723788
H     3.4520226    0.5506156    3.0707719
H     2.8902956   -1.8451364    3.1958854
H     2.7190167   -3.2042932    1.1126376
```

TS1-6

```
E(PBE0-D3/def2-TZVP) = -1856.359089683 (conv) Lowest Freq. =  -998.91 cm^-1
65
TS1-6 (175TSc1/opt)
P     0.0181488   -0.6063759   -1.0067035
C     0.2158321    1.0421105   -0.3846716
C     1.0203480    1.9254179   -1.1163481
H     1.5336619    1.5778786   -2.0052372
C     1.1825625    3.2243125   -0.7020065
H     1.8202656    3.9119776   -1.2425924
C     0.5627530    3.6701741    0.4671717
C    -0.2510405    2.8051880    1.1958896
H    -0.7288567    3.1267040    2.1099557
C    -0.4191340    1.5017240    0.7649362
H    -1.0475768    0.8349591    1.3412788
C    -0.8630865   -1.5864120    0.1888075
C    -1.9911234   -2.3321365   -0.1679204
```

H	-2.3779861	-2.2768614	-1.1785141
C	-2.6199402	-3.1372385	0.7546091
H	-3.4951267	-3.7176082	0.4901734
C	-2.1327076	-3.2185346	2.0620974
C	-1.0124285	-2.4781182	2.4304810
H	-0.6218875	-2.5171385	3.4382221
C	-0.3851883	-1.6695347	1.4969599
H	0.4837693	-1.0964806	1.7959640
C	-0.9694930	-0.5322859	-2.4931602
C	-2.0363130	0.3635094	-2.6002100
H	-2.2464604	1.0512589	-1.7886053
C	-2.8150530	0.3899288	-3.7347051
H	-3.6421020	1.0819974	-3.8342160
C	-2.5394606	-0.4788332	-4.7938785
C	-1.4729552	-1.3695711	-4.7003045
H	-1.2362997	-2.0429301	-5.5125067
C	-0.6968809	-1.3897946	-3.5514504
H	0.1403063	-2.0737930	-3.4787139
O	1.3669656	-1.2326177	-1.4147842
H	2.2930833	-1.1555892	-0.7540387
O	0.8039506	4.9433559	0.8074304
O	-2.8114882	-4.0301044	2.8925189
O	-3.3507333	-0.3776075	-5.8614376
C	0.3128269	5.4034788	2.0464192
H	0.6906115	6.4171843	2.1624726
H	0.6793531	4.7804126	2.8664781
H	-0.7821604	5.4224455	2.0589988
C	-2.3559642	-4.1515607	4.2210856
H	-1.3365652	-4.5484082	4.2579721
H	-3.0322752	-4.8500402	4.7094715
H	-2.3892521	-3.1901739	4.7434245
C	-3.1088016	-1.2221582	-6.9638412
H	-2.1161213	-1.0462493	-7.3901493
H	-3.8669528	-0.9762619	-7.7043781
H	-3.2028200	-2.2768675	-6.6863165

C	1.9330290	1.1971729	4.0736130
C	2.3867123	2.3654342	3.4790634
N	3.0028976	2.3981191	2.2892347
C	3.1879566	1.2501062	1.6420034
C	2.7603211	-0.0252132	2.1587921
C	2.1188350	-0.0084704	3.4224780
C	2.9877836	-1.1840988	1.4120732
C	3.5477503	-1.1266293	0.1011762
C	4.0583352	0.1281498	-0.3351725
C	3.8625733	1.2704600	0.3783959
H	1.4517341	1.2364085	5.0448035
H	2.2711741	3.3194306	3.9868931
H	1.8033983	-0.9434157	3.8756368
H	2.6967337	-2.1438059	1.8280389
H	3.9681433	-2.0334889	-0.3282542
H	4.5808234	0.1754190	-1.2861741
H	4.2115953	2.2316897	0.0212707

TS1-7

E(PBE0-D3/def2-TZVP) = -1856.363339936 (conv) Lowest Freq. = -973.83 cm^-1

65

TS1-7 (176TSc1/opt)

P	0.0081208	-0.6652303	-0.9663900
C	0.2176324	0.9833934	-0.3415120
C	1.1217780	1.8238695	-1.0016647
H	1.7037855	1.4443025	-1.8326665
C	1.3000080	3.1193979	-0.5792938
H	2.0138631	3.7742306	-1.0629768
C	0.5899356	3.6021137	0.5213926
C	-0.3282188	2.7824108	1.1714474
H	-0.8795884	3.1336427	2.0314988
C	-0.5076340	1.4812255	0.7354632
H	-1.2131001	0.8457952	1.2551622
C	-0.8659895	-1.6482381	0.2321236
C	-1.8828726	-2.5202388	-0.1737855
H	-2.1916835	-2.5452602	-1.2122312

C	-2.5016803	-3.3483140	0.7346083
H	-3.2905593	-4.0254710	0.4315794
C	-2.1150681	-3.3269132	2.0776901
C	-1.1091453	-2.4594261	2.4943804
H	-0.7877664	-2.4148449	3.5260420
C	-0.4910010	-1.6273201	1.5760458
H	0.2842802	-0.9637597	1.9433908
C	-0.9977384	-0.5870179	-2.4427355
C	-2.1098761	0.2562385	-2.5055516
H	-2.3499470	0.8975410	-1.6646804
C	-2.8983842	0.2902268	-3.6327541
H	-3.7607841	0.9421152	-3.6976623
C	-2.5889783	-0.5197164	-4.7287114
C	-1.4783106	-1.3577545	-4.6789987
H	-1.2156245	-1.9855737	-5.5191850
C	-0.6912205	-1.3846136	-3.5372047
H	0.1798050	-2.0276981	-3.4974703
O	1.3536739	-1.2726773	-1.4090065
H	2.2646625	-1.2613752	-0.7144027
O	0.8643385	4.8650807	0.8884707
O	-2.7719056	-4.1718048	2.8915926
O	-3.4147639	-0.4168560	-5.7854521
C	0.2352305	5.3687606	2.0456537
H	0.6388361	6.3677428	2.1978999
H	0.4601591	4.7459986	2.9163072
H	-0.8496754	5.4343140	1.9124741
C	-2.4045724	-4.1988577	4.2532029
H	-1.3543025	-4.4799858	4.3765859
H	-3.0374191	-4.9500612	4.7212447
H	-2.5770834	-3.2296588	4.7313729
C	-3.1429852	-1.2065979	-6.9208111
H	-2.1647352	-0.9658747	-7.3490669
H	-3.9201797	-0.9717059	-7.6450363
H	-3.1815207	-2.2743229	-6.6822778
C	1.7339410	1.1983539	3.8757895

C	2.3591683	2.3731300	3.4847939
C	3.1366617	2.3529985	2.3241361
C	3.2774935	1.1697309	1.6160901
C	2.6228339	-0.0057599	2.1236559
N	1.8374050	0.0398582	3.2399755
C	2.7888717	-1.2194993	1.4553949
C	3.4646799	-1.2957558	0.2009391
C	4.1501336	-0.1293302	-0.2467956
C	4.0437381	1.0563135	0.4113919
H	1.1035084	1.1950863	4.7635555
H	2.2464746	3.2744475	4.0753080
H	3.6281383	3.2523415	1.9669082
H	2.3414540	-2.1049899	1.8911486
H	3.8187383	-2.2649782	-0.1444133
H	4.7425782	-0.1861824	-1.1549843
H	4.5423866	1.9430045	0.0330046

TS1-8

E(PBE0-D3/def2-TZVP) = -1856.364776345 (conv) Lowest Freq. = -366.76 cm^-1

65

TS1-8 (177TSc1/opt)

P	-0.1374848	1.1106738	-0.0330088
C	-1.4588804	2.0757411	0.6738020
C	-1.3198588	3.4618594	0.7892984
H	-0.3859335	3.9315383	0.5040325
C	-2.3552742	4.2274715	1.2757400
H	-2.2609830	5.3015053	1.3775760
C	-3.5567897	3.6238941	1.6546259
C	-3.7036611	2.2424479	1.5474766
H	-4.6211863	1.7540920	1.8450868
C	-2.6530681	1.4800915	1.0611513
H	-2.7640118	0.4036768	0.9927456
C	-0.1795441	-0.5116118	0.6643611
C	-0.1873249	-1.6553497	-0.1369089
H	-0.1897960	-1.5611409	-1.2158289
C	-0.1526227	-2.9043927	0.4378861

H	-0.1259526	-3.7995876	-0.1690102
C	-0.1012026	-3.0313612	1.8280380
C	-0.1243555	-1.8973712	2.6365338
H	-0.0769243	-1.9804932	3.7130759
C	-0.1615834	-0.6444153	2.0563393
H	-0.1417299	0.2349375	2.6893619
C	-0.3526182	1.0465810	-1.7947680
C	-1.5980845	1.2645488	-2.3922108
H	-2.4595175	1.5058111	-1.7802587
C	-1.7380911	1.1876203	-3.7588506
H	-2.6946668	1.3581962	-4.2370255
C	-0.6322848	0.8906679	-4.5602887
C	0.6123155	0.6773094	-3.9728683
H	1.4827848	0.4501409	-4.5728345
C	0.7500248	0.7558652	-2.5968105
H	1.7277959	0.5784599	-2.1521381
O	1.1941254	1.8315321	0.3123304
H	2.0279497	1.2119672	0.5694586
O	-4.5096564	4.4521083	2.1157555
O	-0.0215191	-4.2903782	2.2973446
O	-0.8655514	0.8352456	-5.8840683
C	-5.7419630	3.8950218	2.5135899
H	-6.3571837	4.7290727	2.8443918
H	-5.6121737	3.1906775	3.3413238
H	-6.2378390	3.3881242	1.6797015
C	0.1173637	-4.4704602	3.6873624
H	1.0111873	-3.9632999	4.0640671
H	0.2154067	-5.5426006	3.8456075
H	-0.7618159	-4.1048583	4.2282921
C	0.2182127	0.5404737	-6.7364550
H	1.0050640	1.2970244	-6.6568022
H	-0.1837250	0.5435908	-7.7475465
H	0.6407793	-0.4451358	-6.5174151
C	3.2213243	-1.7865815	-1.9709901
C	3.0740967	-3.0459918	-1.4180038

```
C     3.0447370   -3.1660647   -0.0363348
C     3.1652612   -2.0180893    0.7637344
C     3.3222439   -0.7609845    0.0872737
N     3.3491735   -0.6667040   -1.2504311
C     3.4317189    0.4305504    0.8718004
C     3.4464285    0.3425392    2.2832321
C     3.2803637   -0.8711817    2.9170357
C     3.1495128   -2.0449593    2.1747993
H     3.2464125   -1.6636723   -3.0513034
H     2.9854696   -3.9154464   -2.0590819
H     2.9319034   -4.1352131    0.4386197
H     3.8501580    1.3029765    0.3746681
H     3.5874730    1.2443134    2.8693210
H     3.2751811   -0.9163300    4.0008716
H     3.0603899   -3.0046726    2.6735301
```

PR2-1

```
E(PBE0-D3/def2-TZVP) = -633.0670276545 (conv) Lowest Freq. =    50.37 cm^-1
28
PR2-1 (197c1/opt)
H     1.5320689    4.3167327    0.2100585
C     1.6726954    0.2310363    0.2534592
C     0.4605466   -0.4254576    0.0867715
N    -0.6616927    0.3616901   -0.0864820
C    -0.6498299    1.7382889   -0.0816006
C     0.5858315    2.4028110    0.0859971
C     1.7596805    1.6061015    0.2533039
C     0.5867918    3.8003929    0.0825822
C    -0.5854608    4.5204745   -0.0722658
C    -1.7916526    3.8507671   -0.2287323
C    -1.8249245    2.4639128   -0.2355045
H     2.5702514   -0.3654117    0.3460593
H     2.7179915    2.0970194    0.3675831
H    -1.5587267   -0.0922643   -0.0701735
H    -0.5612175    5.6033983   -0.0688558
H    -2.7133005    4.4076285   -0.3476906
```

H	-2.7661997	1.9385622	-0.3612605
C	0.2978346	-1.8614199	0.0351022
C	-0.8204595	-2.4511999	-0.5760743
C	1.2500279	-2.7202436	0.6081011
C	1.0932826	-4.0902863	0.5643970
C	-0.9746966	-3.8264314	-0.6122385
C	-0.0221238	-4.6579703	-0.0437253
H	2.1075431	-2.3013476	1.1201087
H	-1.5579748	-1.8351844	-1.0786127
H	1.8425261	-4.7262210	1.0220154
H	-1.8439929	-4.2515862	-1.1009840
H	-0.1448193	-5.7337917	-0.0713389

PR2-2

E(PBE0-D3/def2-TZVP) = -633.0260569329 (conv) Lowest Freq. = 15.51 cm^-1

28

PR2-2 (194c1/opt)

H	1.4857683	3.8234104	-1.0334585
C	1.5287359	-0.2086838	-1.1230534
C	0.2029873	-0.8802428	-0.9780692
N	-0.9609054	-0.0328443	-0.9294521
C	-0.8038792	1.2826486	-0.9183170
C	0.4682334	1.9453797	-1.0000340
C	1.6412603	1.1227237	-1.1230374
C	0.5199257	3.3321258	-0.9722427
C	-0.6374419	4.0885861	-0.8711518
C	-1.8866610	3.4568334	-0.7984464
C	-1.9728151	2.0890789	-0.8225988
H	2.4003756	-0.8482371	-1.2115653
H	0.0594628	-1.5486630	-1.8429054
H	2.6086212	1.6055423	-1.2178453
H	-0.5764230	5.1701065	-0.8508345
H	-2.7872030	4.0550615	-0.7221370
H	-2.9238314	1.5737504	-0.7649266
C	0.1868366	-1.7873497	0.2465025
C	-0.1813297	-3.1208680	0.1431564

```
C     0.5218513   -1.2696642    1.4925608
C     0.4893224   -2.0758545    2.6183547
C    -0.2124396   -3.9316236    1.2689070
C     0.1228972   -3.4104891    2.5088074
H     0.8097229   -0.2271870    1.5767203
H    -0.4519064   -3.5267599   -0.8259924
H     0.7514467   -1.6624070    3.5855297
H    -0.5013226   -4.9722897    1.1762841
H     0.0987106   -4.0420838    3.3892448
```

PR2-3

```
E(PBE0-D3/def2-TZVP) = -633.0429104264 (conv) Lowest Freq. =    34.94 cm^-1
28
PR2-3 (195c1/opt)
H     1.2858485    4.3557738    0.6103824
C     1.5209053    0.1840598    0.6743327
C     0.2504776   -0.3918475    0.1085732
N    -0.7326636    0.3191451   -0.3102829
C    -0.6819536    1.6971078   -0.2685658
C     0.4572113    2.3994855    0.2317370
C     1.5379542    1.6633518    0.6917430
C     0.4240697    3.8155271    0.2327139
C    -0.6751284    4.4944377   -0.2329878
C    -1.7865739    3.7945621   -0.7191787
C    -1.7808115    2.4095229   -0.7333608
H     2.3771649   -0.2091271    0.1036728
H     2.4159558    2.1704901    1.0764099
H     1.6747740   -0.2182071    1.6885969
H    -0.6834474    5.5782816   -0.2244451
H    -2.6502782    4.3367305   -1.0845970
H    -2.6284010    1.8464455   -1.1057375
C     0.1275026   -1.8580941    0.0302425
C    -1.0255004   -2.4320056   -0.5135446
C     1.1395811   -2.7037652    0.4869646
C     1.0041085   -4.0812655    0.4039465
C    -1.1585542   -3.8039692   -0.5965950
```

C	-0.1436804	-4.6362480	-0.1377597
H	2.0456813	-2.2931634	0.9143187
H	-1.8061463	-1.7702802	-0.8658035
H	1.8004477	-4.7218995	0.7645769
H	-2.0590112	-4.2321303	-1.0218571
H	-0.2495323	-5.7129188	-0.2034956

PR2-4

E(PBE0-D3/def2-TZVP) = -633.0433175769 (conv) Lowest Freq. = 40.98 cm^-1

28

PR2-4 (196c1/opt)

H	1.4033267	4.2751942	0.2344847
C	1.5592073	0.1152010	0.2343358
C	0.3121868	-0.4493090	0.0605144
N	-0.7986617	0.2748935	-0.1238523
C	-0.7112406	1.6397303	-0.1116649
C	0.4923930	2.3464706	0.0820759
C	1.7584760	1.5785036	0.2741900
C	0.4745163	3.7329150	0.0849375
C	-0.7076832	4.4316945	-0.1004511
C	-1.9008668	3.7389664	-0.2943852
C	-1.9019010	2.3617384	-0.2995131
H	2.4321048	-0.5197181	0.3203772
H	2.4986398	1.8743022	-0.4853424
H	2.2307791	1.8632953	1.2280829
H	-0.7019617	5.5152847	-0.0942229
H	-2.8265303	4.2832294	-0.4403792
H	-2.8133395	1.7941526	-0.4437663
C	0.1394342	-1.9206034	0.0341874
C	-0.9370459	-2.4753943	-0.6554881
C	1.0257777	-2.7749560	0.6873943
C	0.8489545	-4.1483541	0.6419806
C	-1.1105582	-3.8479472	-0.7061563
C	-0.2175610	-4.6906952	-0.0593834
H	1.8480962	-2.3651330	1.2623779
H	-1.6336183	-1.8077836	-1.1463175

```
H     1.5423798   -4.7971318    1.1646501
H    -1.9492866   -4.2634973   -1.2529637
H    -0.3560174   -5.7650485   -0.0957020
```

PR2-5

```
E(PBE0-D3/def2-TZVP) = -633.0463563008 (conv) Lowest Freq. =    40.71 cm^-1
28
PR2-5 (190c1/opt)
H    -0.4779565    3.3741523    2.6489749
C     0.4435218    1.3366054   -0.7989810
C     0.0800563   -0.0054130   -0.6348469
N    -0.2071063   -0.5064173    0.5586895
C    -0.1673707    0.2894335    1.6384428
C     0.1715619    1.6550639    1.5610104
C     0.4828905    2.1592094    0.3104065
C     0.2003464    2.5180260    2.7875993
C    -0.1460355    1.8092039    4.0473155
C    -0.4658260    0.4821866    4.0681259
C    -0.4858110   -0.2908976    2.9045323
H     0.7141495    1.7198322   -1.7736558
H     0.7669683    3.2018704    0.2033328
H     1.1943396    2.9808882    2.8864336
H    -0.1395514    2.3836934    4.9664488
H    -0.7123179    0.0122628    5.0142724
H    -0.7417195   -1.3420514    2.9238773
C     0.0191105   -0.9354678   -1.7827740
C    -0.1486953   -0.4781095   -3.0889317
C     0.1260924   -2.3084785   -1.5648664
C     0.0813530   -3.1968045   -2.6250257
C    -0.1993643   -1.3678079   -4.1497272
C    -0.0798368   -2.7306051   -3.9225260
H     0.2432456   -2.6595612   -0.5476097
H    -0.2680525    0.5814662   -3.2816038
H     0.1732207   -4.2607822   -2.4388275
H    -0.3398565   -0.9947464   -5.1576789
H    -0.1173563   -3.4267518   -4.7524076
```

PR2-6

E(PBE0-D3/def2-TZVP) = -633.0406522921 (conv) Lowest Freq. = 32.57 cm^-1

28

PR2-6 (191c1/opt)

H	1.6040903	4.0988332	0.2344856
C	1.6726218	0.0323117	0.2196869
C	0.4071446	-0.5658667	0.0579910
N	-0.6933743	0.1814973	-0.0961002
C	-0.6034521	1.4987461	-0.0791721
C	0.6334150	2.2022329	0.0941886
C	1.7869053	1.3948540	0.2385531
C	0.6559571	3.5869078	0.1048978
C	-0.5681562	4.4078690	-0.0561754
C	-1.8110347	3.6066065	-0.2321974
C	-1.8165343	2.2703930	-0.2418544
H	2.5585222	-0.5823510	0.3079225
H	2.7561670	1.8679833	0.3552163
H	-0.6876950	5.0868386	0.8049123
H	-0.4532973	5.0995174	-0.9077025
H	-2.7407347	4.1525386	-0.3557849
H	-2.7339982	1.7076450	-0.3702469
C	0.2294489	-2.0241281	0.0326890
C	-0.9693057	-2.5682740	-0.4375992
C	1.2256074	-2.9010823	0.4704844
C	1.0335914	-4.2714601	0.4315290
C	-1.1578240	-3.9375279	-0.4797774
C	-0.1573611	-4.7978365	-0.0472980
H	2.1555500	-2.5148746	0.8691972
H	-1.7444671	-1.8892782	-0.7678511
H	1.8174544	-4.9325522	0.7829591
H	-2.0928784	-4.3386934	-0.8540123
H	-0.3063624	-5.8708495	-0.0789411

PR2-7

E(PBE0-D3/def2-TZVP) = -633.0386443778 (conv) Lowest Freq. = 39.71 cm^-1

28

PR2-7 (192c1/opt)

H	1.5944817	4.1654354	0.0946899
C	1.7057792	0.0774114	0.0745625
C	0.4366463	-0.5322306	0.0280316
N	-0.6795434	0.1712025	-0.0245838
C	-0.6132427	1.5242383	-0.0183933
C	0.6331717	2.2184190	0.0422324
C	1.7898220	1.4562275	0.0833885
C	0.6343672	3.6603977	0.0487900
C	-0.4982693	4.3700534	0.0006797
C	-1.8405169	3.7283968	-0.0631980
C	-1.8027274	2.2476816	-0.0693598
H	2.6035733	-0.5253531	0.0754357
H	2.7569617	1.9478264	0.1135076
H	-2.4615499	4.0873010	0.7752329
H	-0.4609736	5.4541902	0.0071693
H	-2.3836639	4.0965289	-0.9503105
H	-2.7309156	1.6904222	-0.1125066
C	0.2989719	-2.0046446	0.0145392
C	-0.8658443	-2.5831997	-0.4890102
C	1.3035008	-2.8411881	0.4992283
C	1.1516494	-4.2181469	0.4746189
C	-1.0146301	-3.9584856	-0.5199281
C	-0.0059170	-4.7825731	-0.0395778
H	2.2045006	-2.4168461	0.9257894
H	-1.6477977	-1.9287523	-0.8523352
H	1.9391378	-4.8525780	0.8647840
H	-1.9233434	-4.3919267	-0.9218655
H	-0.1236285	-5.8598077	-0.0616111

PR2-8

E(PBE0-D3/def2-TZVP) = -633.0491449110 (conv) Lowest Freq. = 35.98 cm^-1

28

PR2-8 (193c1/opt)

H	1.5517361	4.2368594	0.2123153

C	1.7199674	0.1522796	0.1965074
C	0.4736996	-0.4705260	0.0598125
N	-0.6446868	0.2502116	-0.0736289
C	-0.5879510	1.5684312	-0.0602025
C	0.6196002	2.2868841	0.0888996
C	1.7884886	1.5243609	0.2127319
C	0.6121476	3.7086817	0.0991324
C	-0.5878747	4.4175169	-0.0348629
C	-1.7837767	3.7794385	-0.1826577
C	-1.8912511	2.2975184	-0.2110880
H	2.6244999	-0.4371192	0.2666526
H	2.7450841	2.0268354	0.3120815
H	-2.5773623	1.9463070	0.5729076
H	-0.5611818	5.5016888	-0.0200945
H	-2.6976307	4.3534708	-0.2847920
H	-2.3689701	1.9648927	-1.1441298
C	0.3283753	-1.9364630	0.0368170
C	-0.8350462	-2.5090478	-0.4805833
C	1.3250247	-2.7837310	0.5239918
C	1.1679397	-4.1589951	0.4863188
C	-0.9892529	-3.8832090	-0.5220185
C	0.0118865	-4.7157549	-0.0406612
H	2.2250180	-2.3692867	0.9618625
H	-1.6117342	-1.8504540	-0.8471236
H	1.9503360	-4.7992849	0.8771780
H	-1.8970577	-4.3092807	-0.9337851
H	-0.1100277	-5.7922243	-0.0715809

TS2-2

E(PBE0-D3/def2-TZVP) = -2087.244265400 (conv) Lowest Freq. = -1258.68 cm^-1

75

TS2-2 (254TSc1/opt)

P	-0.5633474	-0.1354489	-0.9474363
C	-0.4068463	1.5138074	-0.3010887
C	0.2989754	2.4602077	-1.0534731
H	0.7436190	2.1752137	-2.0003153

C	0.4475498	3.7452013	-0.5914610
H	1.0022867	4.4857929	-1.1539516
C	-0.1014043	4.1140375	0.6398497
C	-0.8052537	3.1815955	1.3950234
H	-1.2109951	3.4392747	2.3623579
C	-0.9479516	1.8884751	0.9218776
H	-1.4765467	1.1594076	1.5237778
C	-1.4588042	-1.1356363	0.2289888
C	-2.8262415	-1.3854148	0.0875116
H	-3.3665392	-0.9871844	-0.7634651
C	-3.4974171	-2.1475700	1.0181995
H	-4.5560677	-2.3533054	0.9190033
C	-2.8112306	-2.6806860	2.1108554
C	-1.4469736	-2.4388383	2.2571575
H	-0.8932129	-2.8455831	3.0924344
C	-0.7773021	-1.6695999	1.3203670
H	0.2873703	-1.4749044	1.4548347
C	-1.5394895	-0.0378931	-2.4416760
C	-2.4916518	0.9645493	-2.6365778
H	-2.6239646	1.7356765	-1.8859802
C	-3.2565323	0.9894650	-3.7822653
H	-3.9952430	1.7636283	-3.9493818
C	-3.0803407	0.0111517	-4.7625623
C	-2.1269848	-0.9891410	-4.5808499
H	-1.9681779	-1.7504800	-5.3320357
C	-1.3663585	-1.0070518	-3.4233847
H	-0.6166854	-1.7776535	-3.2845910
O	0.7918430	-0.7372905	-1.3130147
H	1.7366877	-0.6455005	-0.5434253
O	0.1085108	5.3868400	1.0152018
O	-3.5468356	-3.4149849	2.9655302
O	-3.8679336	0.1180091	-5.8476055
C	-0.4143929	5.8052123	2.2564156
H	-0.1130402	6.8439609	2.3757438
H	-0.0048048	5.2083280	3.0759463

H	-1.5074254	5.7416126	2.2673294
C	-2.8969417	-3.9846169	4.0793408
H	-2.1077224	-4.6766107	3.7693954
H	-3.6613087	-4.5317598	4.6273696
H	-2.4679071	-3.2142696	4.7279096
C	-3.7255562	-0.8412265	-6.8707127
H	-2.7177928	-0.8223064	-7.2975315
H	-4.4466746	-0.5715336	-7.6394623
H	-3.9459530	-1.8493866	-6.5056863
C	1.3812046	2.0568507	4.0426039
C	2.0079812	3.1894213	3.5149546
C	2.6813266	3.0948553	2.3079976
C	2.7565202	1.8892324	1.6155678
C	2.1307609	0.7198027	2.1527293
C	1.4360299	0.8519952	3.3807863
N	2.1906182	-0.4835142	1.5697638
C	2.8289786	-0.6158363	0.3445477
C	3.5432861	0.5168405	-0.2002068
C	3.4777769	1.7302712	0.3883095
H	0.8542031	2.1254938	4.9885728
H	1.9836368	4.1289758	4.0548929
H	3.1700503	3.9686720	1.8875740
H	0.9657592	-0.0352016	3.7895921
H	4.1171174	0.3852971	-1.1101066
H	3.9915300	2.5854954	-0.0379453
C	3.2692624	-1.9917988	-0.0017099
C	3.1512661	-3.0269538	0.9279562
C	3.7592363	-2.3056817	-1.2731076
C	4.1323944	-3.5982325	-1.5952899
C	3.5211932	-4.3218095	0.6014055
C	4.0166784	-4.6184775	-0.6596862
H	3.8296560	-1.5330709	-2.0305330
H	2.7744784	-2.7868655	1.9138649
H	4.5111037	-3.8133999	-2.5885582
H	3.4288168	-5.1067829	1.3445354

H	4.3081427	-5.6311485	-0.9129976

TS2-3

E(PBE0-D3/def2-TZVP) = -2087.243813257 (conv) Lowest Freq. = -787.06 cm^-1

75

TS2-3 (255TSc1/opt)

P	-0.7388611	-0.6905221	-0.6170094
C	-0.4435312	0.9534267	-0.0238333
C	0.4855758	1.7519755	-0.7022487
H	1.0448868	1.3487703	-1.5386636
C	0.7294735	3.0373587	-0.2848925
H	1.4783332	3.6480583	-0.7719487
C	0.0604476	3.5515512	0.8263203
C	-0.8868716	2.7761025	1.4923427
H	-1.4054265	3.1528106	2.3621182
C	-1.1296793	1.4847487	1.0650431
H	-1.8515512	0.8800543	1.5991883
C	-1.6567144	-1.5950536	0.6049965
C	-2.8050124	-2.3205654	0.2719485
H	-3.1898028	-2.2884651	-0.7405724
C	-3.4555098	-3.0752097	1.2212671
H	-4.3474859	-3.6387539	0.9774692
C	-2.9681998	-3.1262619	2.5309088
C	-1.8259263	-2.4076563	2.8741923
H	-1.4344553	-2.4243002	3.8821452
C	-1.1767196	-1.6484824	1.9147228
H	-0.2910696	-1.0915690	2.1968822
C	-1.7095013	-0.6101964	-2.1115527
C	-2.7795186	0.2822754	-2.2163697
H	-3.0015881	0.9583949	-1.3980151
C	-3.5467521	0.3207220	-3.3578724
H	-4.3764364	1.0097239	-3.4562398
C	-3.2543478	-0.5304917	-4.4271283
C	-2.1834530	-1.4159662	-4.3357892
H	-1.9334328	-2.0747143	-5.1558548
C	-1.4198812	-1.4500474	-3.1789879

H	-0.5797791	-2.1302124	-3.1074066
O	0.5809879	-1.3967530	-1.0054522
H	1.4782118	-1.3059596	-0.3579488
O	0.4008526	4.7985207	1.1916739
O	-3.6695453	-3.8886652	3.3882568
O	-4.0548280	-0.4179906	-5.5010867
C	-0.1606722	5.3175699	2.3763138
H	0.2963630	6.2939479	2.5239601
H	0.0654132	4.6740181	3.2316002
H	-1.2452781	5.4374250	2.2849755
C	-3.2147600	-3.9805668	4.7196901
H	-2.2076497	-4.4063453	4.7679206
H	-3.9106365	-4.6422125	5.2312809
H	-3.2179429	-3.0021142	5.2103008
C	-3.7943661	-1.2420988	-6.6150094
H	-2.7984754	-1.0513642	-7.0270556
H	-4.5462827	-0.9892871	-7.3595109
H	-3.8834533	-2.3019267	-6.3561318
C	1.3085527	0.9553965	4.5877889
C	1.7674729	2.1543101	4.0497104
C	2.3715450	2.1600010	2.7958209
C	2.5204929	0.9889825	2.0594996
C	2.0601901	-0.2509733	2.6101557
C	1.4554820	-0.2273442	3.8871767
C	2.2426560	-1.4118209	1.8460649
C	2.7685743	-1.3051468	0.5236605
C	3.2498625	-0.0310783	0.1112546
N	3.1169179	1.0614973	0.8218881
H	0.8440588	0.9448229	5.5681615
H	1.6715528	3.0757190	4.6130988
H	2.7435983	3.0785768	2.3562551
H	1.1223586	-1.1649274	4.3234235
H	1.9607062	-2.3783203	2.2486182
H	3.1782450	-2.1924542	0.0496685
C	3.8756784	0.1359943	-1.2229088

C	4.5755011	1.3087126	-1.5096823
C	3.7698596	-0.8336775	-2.2198409
C	4.3495042	-0.6390019	-3.4638354
C	5.1587191	1.5012351	-2.7501707
C	5.0486923	0.5279888	-3.7343209
H	3.2102295	-1.7418570	-2.0342566
H	4.6439754	2.0575769	-0.7309345
H	4.2513120	-1.4027832	-4.2271594
H	5.7050051	2.4160851	-2.9515459
H	5.5041089	0.6787837	-4.7065282

TS2-4

E(PBE0-D3/def2-TZVP) = -2087.250855405 (conv) Lowest Freq. = -326.71 cm^-1

75

TS2-4 (256TSc1/opt)

P	-0.4672220	1.3554268	-0.3522763
C	-1.8251307	2.3669608	0.2075094
C	-1.6783583	3.7550763	0.2809235
H	-0.7226723	4.2050607	0.0401125
C	-2.7341428	4.5472800	0.6719433
H	-2.6343965	5.6234911	0.7402672
C	-3.9625028	3.9684381	0.9998634
C	-4.1168548	2.5850963	0.9339720
H	-5.0553420	2.1155252	1.1939683
C	-3.0480219	1.7967948	0.5392629
H	-3.1677258	0.7199341	0.5006058
C	-0.5959190	-0.2356113	0.4129200
C	-0.7850268	-1.4035496	-0.3178066
H	-0.8467682	-1.3651393	-1.3975477
C	-0.8786311	-2.6270912	0.3190029
H	-0.9940943	-3.5237770	-0.2731924
C	-0.7966648	-2.6884086	1.7078406
C	-0.6393413	-1.5171393	2.4530916
H	-0.5535765	-1.5936013	3.5295083
C	-0.5248838	-0.3088792	1.8119657
H	-0.3599693	0.5897495	2.3954763

C	-0.5900151	1.1803973	-2.1180206
C	-1.6083825	1.8040550	-2.8455596
H	-2.3591693	2.3924434	-2.3319676
C	-1.6663502	1.6778600	-4.2153716
H	-2.4491607	2.1564491	-4.7906717
C	-0.7026276	0.9256608	-4.8910431
C	0.3156388	0.3003353	-4.1754469
H	1.0810877	-0.2841581	-4.6661579
C	0.3665045	0.4297022	-2.7993150
H	1.1707127	-0.0625334	-2.2653556
O	0.8161237	2.1145339	0.0668863
H	1.7823993	1.6165299	0.1685359
O	-4.9321481	4.8207023	1.3731213
O	-0.8589974	-3.8262820	2.4147511
O	-0.8363334	0.8606777	-6.2271469
C	-6.1892075	4.2879109	1.7250934
H	-6.8132269	5.1383664	1.9912235
H	-6.1097450	3.6144587	2.5842092
H	-6.6447875	3.7536083	0.8853734
C	-0.8649667	-5.0466459	1.7113173
H	-1.7670686	-5.1538949	1.0998776
H	-0.8496111	-5.8297511	2.4666046
H	0.0204009	-5.1369219	1.0745668
C	0.1211211	0.1215529	-6.9529601
H	1.1265108	0.5319922	-6.8177860
H	-0.1649964	0.2034195	-7.9994630
H	0.1185020	-0.9320864	-6.6570646
C	3.6569201	-0.4860759	-3.0819768
C	3.2080905	-1.7802717	-2.8309788
C	2.8289314	-2.1507677	-1.5525336
C	2.8886192	-1.2444744	-0.4767864
C	3.2976909	0.0965838	-0.7527840
C	3.6881716	0.4402163	-2.0491627
C	3.2552481	1.0285830	0.3427762
C	3.0743098	0.4934586	1.6373655

C	2.7099482	-0.8312038	1.8038678
N	2.5740999	-1.6842513	0.7668088
H	3.9761285	-0.1995191	-4.0777646
H	3.1726702	-2.5080263	-3.6347707
H	2.5044609	-3.1617600	-1.3335997
H	4.0200425	1.4561369	-2.2411802
H	3.7564569	1.9865950	0.2259998
H	3.1965480	1.1393554	2.4975755
C	2.4492838	-1.3936056	3.1412771
C	2.2146572	-0.5940074	4.2635699
C	2.3809738	-2.7794054	3.3031019
C	2.0862710	-3.3419105	4.5335347
C	1.9199268	-1.1557522	5.4938017
C	1.8504910	-2.5361776	5.6379168
H	2.5505506	-3.3954094	2.4294343
H	2.2321246	0.4855081	4.1720149
H	2.0349312	-4.4209805	4.6308050
H	1.7337993	-0.5112444	6.3460143
H	1.6136912	-2.9756127	6.6000318

TS2-5

E(PBE0-D3/def2-TZVP) = -2087.249176124 (conv) Lowest Freq. = -872.55 cm^-1

75

TS2-5 (250TSc1/opt)

P	-0.6573645	1.2191762	1.0953307
C	-1.9508234	2.3857498	1.4891504
C	-1.6827323	3.7568845	1.4679874
H	-0.6776208	4.1004124	1.2549450
C	-2.6828599	4.6670850	1.7292380
H	-2.4893065	5.7326200	1.7200162
C	-3.9749379	4.2256655	2.0212164
C	-4.2501160	2.8598505	2.0519174
H	-5.2409411	2.4958710	2.2859635
C	-3.2371891	1.9530046	1.7857598
H	-3.4555688	0.8914318	1.8206207
C	-0.9120417	-0.2677329	2.0362972

C	-1.5763811	-1.3821674	1.5160862
H	-1.9089016	-1.3800776	0.4851117
C	-1.8003008	-2.4906431	2.3000559
H	-2.3018480	-3.3658156	1.9065234
C	-1.3622640	-2.5095906	3.6270880
C	-0.7061684	-1.4022683	4.1580139
H	-0.3513956	-1.4008023	5.1792313
C	-0.4822595	-0.2929857	3.3604959
H	0.0446390	0.5597506	3.7707764
C	-0.7634670	0.8297939	-0.6308358
C	-1.5002582	1.6275430	-1.5132332
H	-2.0738167	2.4663004	-1.1366171
C	-1.5053515	1.3534857	-2.8617683
H	-2.0705552	1.9627906	-3.5561595
C	-0.7691991	0.2749594	-3.3634501
C	-0.0389195	-0.5312729	-2.4911798
H	0.5484284	-1.3630095	-2.8533629
C	-0.0391850	-0.2486361	-1.1388944
H	0.5439978	-0.8788576	-0.4790289
O	0.6588561	1.9124193	1.5045085
H	1.6441787	1.3693818	1.5580661
O	-4.8834907	5.1862463	2.2651984
O	-1.6164503	-3.6381059	4.3118171
O	-0.8237977	0.0894787	-4.6883045
C	-6.2033187	4.7941824	2.5672743
H	-6.7624212	5.7139286	2.7255747
H	-6.2404723	4.1873488	3.4775750
H	-6.6525655	4.2356375	1.7399499
C	-1.1604443	-3.7259202	5.6427130
H	-0.0716887	-3.6280652	5.6971139
H	-1.4513548	-4.7126526	5.9968667
H	-1.6255637	-2.9629192	6.2750819
C	-0.0743798	-0.9789086	-5.2415998
H	0.9929513	-0.8678410	-5.0372533
H	-0.2464625	-0.9385912	-6.3148045

H	-0.4165110	-1.9422976	-4.8535152
C	3.5786499	0.4483738	-2.0039181
C	3.2813581	-0.9060426	-2.1429384
N	2.9509087	-1.6634500	-1.0900186
C	2.9129659	-1.1166046	0.1368126
C	3.1976907	0.2674734	0.3673881
C	3.5360091	1.0325833	-0.7501091
C	3.0608689	0.8018015	1.6909632
C	2.8697111	-0.0928285	2.7826540
C	2.5898361	-1.4201173	2.5447283
C	2.5700320	-1.9291098	1.2460840
H	3.8200693	1.0544335	-2.8669413
H	3.7564826	2.0882166	-0.6290954
H	3.5107194	1.7742297	1.8833655
H	2.8966134	0.2898463	3.7968037
H	2.3827392	-2.0841178	3.3769393
H	2.3550822	-2.9733566	1.0546503
C	3.2597631	-1.5668028	-3.4606309
C	3.7447160	-0.9551728	-4.6214711
C	2.7144101	-2.8499906	-3.5815868
C	2.6340710	-3.4805363	-4.8117598
C	3.6672390	-1.5878688	-5.8505560
C	3.1040695	-2.8532205	-5.9576755
H	2.3613302	-3.3337731	-2.6794512
H	4.2012070	0.0252682	-4.5680595
H	2.2032529	-4.4739233	-4.8766297
H	4.0539391	-1.0897758	-6.7327210
H	3.0439087	-3.3473725	-6.9203839

TS2-6

E(PBE0-D3/def2-TZVP) = -2087.246825132 (conv) Lowest Freq. = -1024.84 cm^-1

75

TS2-6 (251TSc1/opt)

P	-0.4182581	-1.2014256	-1.4743539
C	-0.4788487	0.3779637	-0.6705632
C	0.5826708	1.2653290	-0.8224264

H	1.4680214	0.9557734	-1.3650818
C	0.5407828	2.5260045	-0.2548307
H	1.4005898	3.1741746	-0.3439240
C	-0.5845568	2.9213160	0.4667723
C	-1.6497262	2.0297787	0.6335118
H	-2.5040866	2.3496757	1.2170182
C	-1.5950253	0.7745876	0.0763094
H	-2.4189526	0.0876596	0.2336671
C	-1.1888208	-2.3844890	-0.3836691
C	-1.9008914	-3.4738177	-0.8944670
H	-2.0501922	-3.5673429	-1.9641407
C	-2.4236549	-4.4270617	-0.0505501
H	-2.9790429	-5.2745755	-0.4324883
C	-2.2472696	-4.3106497	1.3309806
C	-1.5461555	-3.2251269	1.8520115
H	-1.4035330	-3.1120447	2.9179706
C	-1.0212284	-2.2729695	0.9941255
H	-0.4762315	-1.4322213	1.4068240
C	-1.3728292	-1.1627795	-2.9875996
C	-2.5994635	-0.4975725	-3.0604528
H	-2.9808717	0.0376893	-2.1987014
C	-3.3270169	-0.4975621	-4.2282064
H	-4.2755655	0.0200707	-4.2998380
C	-2.8428289	-1.1643804	-5.3567164
C	-1.6189563	-1.8251512	-5.2969890
H	-1.2195582	-2.3384772	-6.1607988
C	-0.8933229	-1.8175472	-4.1151206
H	0.0665722	-2.3175401	-4.0693420
O	1.0046717	-1.5937236	-1.8952784
H	1.8295346	-1.9138470	-1.1459445
O	-0.7373451	4.1214960	1.0372070
O	-2.7931215	-5.2860749	2.0759778
O	-3.6216370	-1.1067507	-6.4513208
C	0.2878893	5.0834111	0.8653501
H	-0.0467615	5.9722090	1.3947205

H	0.4249741	5.3165343	-0.1953956
H	1.2296840	4.7431040	1.2995165
C	-2.6338736	-5.2236949	3.4760761
H	-1.5766946	-5.2514871	3.7576587
H	-3.1364608	-6.1018092	3.8758409
H	-3.0964258	-4.3220961	3.8896299
C	-3.1727820	-1.7517877	-7.6215696
H	-2.2240392	-1.3310749	-7.9693616
H	-3.9396763	-1.5800704	-8.3739066
H	-3.0558777	-2.8286515	-7.4633403
C	1.5880171	0.8072147	3.2962839
C	2.3436880	1.7955373	2.6476180
N	3.0938919	1.4975150	1.5644472
C	3.0766966	0.2709882	1.0707792
C	2.2947269	-0.7994325	1.6363641
C	1.5709752	-0.4772962	2.8163281
C	2.2834096	-2.0455667	1.0236504
C	2.9432005	-2.2674427	-0.2215320
C	3.8210881	-1.2413184	-0.6839903
C	3.8676652	-0.0195214	-0.0904523
H	1.0390793	1.0497993	4.1979286
H	1.0174401	-1.2546095	3.3339768
H	1.7120706	-2.8505685	1.4746596
H	3.1243633	-3.2896704	-0.5435188
H	4.4221734	-1.4299702	-1.5681908
H	4.4934030	0.7768330	-0.4756981
C	2.3726121	3.1817764	3.1060135
C	3.3750418	4.0493694	2.6465419
C	1.4075600	3.7085866	3.9755420
C	1.4452932	5.0344023	4.3686687
C	3.4107510	5.3740032	3.0436058
C	2.4466694	5.8803633	3.9073293
H	0.5953444	3.0820332	4.3236857
H	4.1179104	3.6493188	1.9685238
H	0.6779669	5.4155015	5.0336720

```
H     4.2011616    6.0202983    2.6771073
H     2.4739927    6.9188813    4.2158653
```

TS2-7

```
E(PBE0-D3/def2-TZVP) = -2087.245902315 (conv) Lowest Freq. = -1219.18 cm^-1
75
TS2-7 (252TSc1/opt)
P     0.0876716   -0.8510763   -1.8006942
C     0.2834785    0.8042000   -1.1793173
C     1.1536334    1.6613533   -1.8630366
H     1.7203449    1.2923393   -2.7094482
C     1.3183583    2.9609266   -1.4475450
H     2.0060864    3.6283942   -1.9514781
C     0.6269285    3.4327755   -0.3302777
C    -0.2563646    2.5965486    0.3455164
H    -0.7914666    2.9348755    1.2213158
C    -0.4230299    1.2917715   -0.0849857
H    -1.1029527    0.6468977    0.4561415
C    -0.7698099   -1.8227595   -0.5780990
C    -1.8220163   -2.6686298   -0.9470658
H    -2.1396844   -2.7182350   -1.9819085
C    -2.4706933   -3.4347251   -0.0047627
H    -3.2899279   -4.0877129   -0.2791114
C    -2.0846620   -3.3696918    1.3367255
C    -1.0346772   -2.5371231    1.7141224
H    -0.7163972   -2.4558265    2.7442104
C    -0.3829157   -1.7739834    0.7612306
H     0.4226399   -1.1321583    1.1035410
C    -0.9531172   -0.7795155   -3.2545684
C    -2.1146771   -0.0030596   -3.2646412
H    -2.3789443    0.5864293   -2.3934934
C    -2.9237276    0.0296475   -4.3770030
H    -3.8248888    0.6298328   -4.4010361
C    -2.5852716   -0.7135538   -5.5112995
C    -1.4261870   -1.4843602   -5.5138418
H    -1.1417565   -2.0605916   -6.3834322
```

C	-0.6192735	-1.5114452	-4.3856152
H	0.2881422	-2.1033152	-4.3842539
O	1.4278265	-1.4404316	-2.2697149
H	2.3982278	-1.3881500	-1.6137924
O	0.8865480	4.7022728	0.0276108
O	-2.7846431	-4.1357486	2.1911797
O	-3.4344086	-0.6169458	-6.5501009
C	0.2572614	5.2035550	1.1849074
H	0.6305020	6.2170168	1.3173927
H	0.5129975	4.6023819	2.0623288
H	-0.8308870	5.2307517	1.0674800
C	-2.4413338	-4.0813378	3.5589168
H	-1.4145370	-4.4218001	3.7247029
H	-3.1306535	-4.7509028	4.0691776
H	-2.5540298	-3.0684191	3.9578829
C	-3.1349582	-1.3394972	-7.7225183
H	-2.1802785	-1.0217189	-8.1536846
H	-3.9369556	-1.1215616	-8.4246813
H	-3.1051716	-2.4167884	-7.5303808
C	1.7876542	1.0121984	2.9329909
C	2.4255711	2.1943044	2.5636354
C	3.2329646	2.2002666	1.4277736
C	3.4034983	1.0362416	0.6933221
C	2.7702633	-0.1557904	1.1829504
N	1.9695724	-0.1355551	2.2800544
C	2.9571742	-1.3572673	0.4983508
C	3.6093341	-1.3960244	-0.7718834
C	4.2745546	-0.2090406	-1.2021205
C	4.1646567	0.9596254	-0.5146413
H	2.3102874	3.0917635	3.1573950
H	3.7202828	3.1143268	1.1045307
H	2.5167212	-2.2554836	0.9150133
H	3.9844469	-2.3508516	-1.1357859
H	4.8539787	-0.2377198	-2.1198105
H	4.6462095	1.8610911	-0.8796403

C	0.8449988	0.9600977	4.0691860
C	0.2072237	2.0994864	4.5611756
C	0.5426958	-0.2684828	4.6609612
C	-0.3591950	-0.3520211	5.7077736
C	-0.6973920	2.0170800	5.6074666
C	-0.9868896	0.7899778	6.1871499
H	1.0425241	-1.1491313	4.2773088
H	0.4068703	3.0639062	4.1088434
H	-0.5693011	-1.3150338	6.1612078
H	-1.1846334	2.9159809	5.9684991
H	-1.6944202	0.7251499	7.0056984

TS2-8

E(PBE0-D3/def2-TZVP) = -2087.254703051 (conv) Lowest Freq. = -576.49 cm^-1

75

TS2-8 (253TSc1/opt)

P	-0.5286751	1.3234881	0.6269335
C	-1.8254836	2.3009190	1.3662528
C	-1.6419771	3.6767722	1.5294520
H	-0.6948748	4.1259462	1.2546492
C	-2.6493488	4.4569663	2.0501635
H	-2.5199768	5.5230771	2.1897819
C	-3.8677688	3.8789919	2.4147309
C	-4.0595509	2.5078280	2.2584366
H	-4.9909512	2.0386909	2.5436389
C	-3.0363731	1.7304826	1.7388350
H	-3.1838758	0.6615464	1.6323230
C	-0.6233313	-0.3164431	1.3006457
C	-0.7182365	-1.4429236	0.4818796
H	-0.7534372	-1.3289848	-0.5944629
C	-0.7346176	-2.7025236	1.0365746
H	-0.7781558	-3.5875639	0.4152744
C	-0.6513386	-2.8581466	2.4199060
C	-0.5788396	-1.7381606	3.2464307
H	-0.5010218	-1.8405485	4.3194149
C	-0.5637322	-0.4769859	2.6840934

H	-0.4739268	0.3888765	3.3300494
C	-0.7838289	1.2610796	-1.1276971
C	-2.0371707	1.5112803	-1.6947011
H	-2.8675995	1.8142415	-1.0677132
C	-2.2239138	1.3837879	-3.0523730
H	-3.1869233	1.5780792	-3.5081947
C	-1.1599827	0.9961169	-3.8716816
C	0.0949229	0.7550057	-3.3162279
H	0.9349457	0.4530922	-3.9270281
C	0.2791115	0.8907054	-1.9513678
H	1.2610291	0.6936544	-1.5269574
O	0.8185464	1.9953206	0.9689249
H	1.6772343	1.3709438	1.2730346
O	-4.7908914	4.7198502	2.9118942
O	-0.6311535	-4.1259731	2.8682918
O	-1.4368838	0.8828880	-5.1809132
C	-6.0404513	4.1892803	3.2917909
H	-6.6277306	5.0303887	3.6539860
H	-5.9331626	3.4513887	4.0931323
H	-6.5533495	3.7296393	2.4409800
C	-0.4794881	-4.3382056	4.2532772
H	0.4518692	-3.8973281	4.6219045
H	-0.4486099	-5.4169459	4.3919562
H	-1.3234817	-3.9257900	4.8157110
C	-0.3918964	0.4769768	-6.0416017
H	0.4312513	1.1976313	-6.0341057
H	-0.8257260	0.4302139	-7.0384283
H	-0.0019940	-0.5048683	-5.7602296
C	2.7698881	-1.6019696	-1.2380657
C	2.5861737	-2.8483998	-0.6361691
C	2.5676825	-2.9484478	0.7372324
C	2.7157096	-1.7914559	1.5293871
C	2.9051434	-0.5529169	0.8261898
N	2.9398571	-0.4830884	-0.5027531
C	3.0266853	0.6551406	1.5938682

```
C     3.0620919    0.5814100    3.0095107
C     2.8768941   -0.6149726    3.6624443
C     2.7117813   -1.7973778    2.9314676
H     2.4940201   -3.7394474   -1.2450363
H     2.4453223   -3.9121528    1.2206267
H     3.4882108    1.5018574    1.0893263
H     3.2254828    1.4895786    3.5799975
H     2.8820377   -0.6476843    4.7463673
H     2.5988397   -2.7466724    3.4458459
C     2.8279183   -1.4416926   -2.6926189
C     3.4420890   -0.3098369   -3.2457565
C     2.2724074   -2.3767454   -3.5753173
C     2.3375947   -2.1950351   -4.9454517
C     3.5066504   -0.1299908   -4.6167161
C     2.9568752   -1.0704209   -5.4798392
H     1.7611014   -3.2477571   -3.1826823
H     3.8657295    0.4198695   -2.5673875
H     1.8962555   -2.9353151   -5.6041754
H     3.9967655    0.7508849   -5.0175046
H     3.0116132   -0.9311198   -6.5534560
```

Ph3P.+

```
E(PBE0-D3/def2-TZVP) = -1035.383125836 (conv) Lowest Freq. =    35.84 cm^-1
34
Ph3P.cat (035c1/opt)
P     0.7365581    0.0961722    0.1294817
C    -0.0224858    1.4744616    0.9302931
C     0.3992123    2.7645759    0.5792314
H     1.1713135    2.9034257   -0.1692711
C    -0.1705461    3.8594899    1.1983307
H     0.1504908    4.8579705    0.9289057
C    -1.1438774    3.6775341    2.1743803
H    -1.5841494    4.5393595    2.6613244
C    -1.5533513    2.3992787    2.5330288
H    -2.3165023    2.2652363    3.2896905
C    -0.9948994    1.2930519    1.9204223
H    -1.3216946    0.2964743    2.1899222
C     0.3680070   -1.4650479    0.8674543
C    -0.1986374   -2.5049059    0.1215808
H    -0.4499576   -2.3560969   -0.9211665
C    -0.4588091   -3.7159132    0.7353639
H    -0.9106000   -4.5193015    0.1667394
C    -0.1498702   -3.8994444    2.0773953
H    -0.3536985   -4.8525691    2.5503721
C     0.4225332   -2.8709415    2.8173918
H     0.6688403   -3.0227227    3.8609104
```

C	0.6908595	-1.6561275	2.2181746
H	1.1533201	-0.8583992	2.7885986
C	0.6041568	0.1150452	-1.6309899
C	-0.2202577	1.0354883	-2.2882144
H	-0.8198781	1.7356786	-1.7200905
C	-0.2819671	1.0250774	-3.6691742
H	-0.9290632	1.7255722	-4.1824567
C	0.4759742	0.1150004	-4.3955108
H	0.4244112	0.1152103	-5.4776562
C	1.3030673	-0.7936677	-3.7448040
H	1.8974626	-1.4953494	-4.3166050
C	1.3781716	-0.7939310	-2.3660061
H	2.0358667	-1.4896849	-1.8570471

PhTHQ (2a)

E(PBE0-D3/def2-TZVP) = -634.8949729106 (conv) Lowest Freq. = 32.69 cm^-1
31
PhTHQ (153c1/opt)

C	1.8051546	-0.0416081	0.4063635
C	2.9829483	0.6909273	0.5779914
C	4.2047884	0.0540059	0.6781273
C	4.2801094	-1.3302764	0.6029561
C	3.1124338	-2.0555760	0.4314571
C	1.8713067	-1.4399913	0.3384544
C	0.6111959	-2.2450816	0.1791848
C	-0.5315371	-1.4073351	-0.3683152
C	-0.6472518	-0.0966198	0.4049090
N	0.5984646	0.6211742	0.2616814
H	2.9277871	1.7741765	0.6257044
H	5.1044202	0.6438142	0.8121623
H	5.2348971	-1.8362007	0.6753554
H	3.1567866	-3.1389199	0.3690754
H	0.3205693	-2.6638643	1.1504813
H	0.7994170	-3.1003078	-0.4757798
H	-1.4784000	-1.9476006	-0.3062844
H	-0.3594044	-1.1653282	-1.4215710
C	-1.6900449	1.4952857	-1.2489848
H	-0.7428298	1.5162725	-1.7760340
H	0.5859068	1.5608366	0.6245736
H	-0.8409169	-0.3490194	1.4609917
C	-1.8000459	0.7372107	-0.0872250
C	-3.0124672	0.7228846	0.5921130
H	-3.1039904	0.1416805	1.5044035
C	-4.0987168	1.4444231	0.1200057
H	-5.0366150	1.4251147	0.6631372
C	-3.9812300	2.1960299	-1.0393105
H	-4.8267368	2.7651629	-1.4078402
C	-2.7727767	2.2205538	-1.7210986
H	-2.6732220	2.8081762	-2.6266849

TS3

E(PBE0-D3/def2-TZVP) = -1670.307245797 (conv) Lowest Freq. = -721.48 cm^-1
65
TS3 (164TSc1/opt)

C	2.6517430	0.2385171	-1.7382127
C	3.8962798	0.7993266	-1.4495265
C	5.0198299	-0.0009908	-1.3984547
C	4.9126151	-1.3659493	-1.6367463
C	3.6733520	-1.9157843	-1.9266853
C	2.5278956	-1.1344264	-1.9795712
C	1.1727242	-1.7235753	-2.2385907
C	0.1954290	-0.6819523	-2.7586482

```
C     0.2433018    0.5901769   -1.9408172
N     1.5311613    1.0518264   -1.7563073
H     3.9739694    1.8660616   -1.2663294
H     5.9837776    0.4411456   -1.1784627
H     5.7922015   -1.9965268   -1.6052042
H     3.5876863   -2.9803375   -2.1176314
H     0.7832836   -2.1704815   -1.3156633
H     1.2547626   -2.5436423   -2.9549732
H    -0.8182710   -1.0804089   -2.7717339
H     0.4434957   -0.4134902   -3.7927681
C    -0.4975751    2.9737298   -2.3095930
H     0.5300360    3.3151625   -2.3578086
H     1.6513077    1.9848750   -1.3964704
H    -0.1516532    0.1560032   -0.7028220
C    -0.7954614    1.6169340   -2.1753837
C    -2.1382670    1.2296846   -2.1894994
H    -2.3996738    0.1839517   -2.0764926
C    -3.1475561    2.1636969   -2.3270526
H    -4.1813320    1.8401328   -2.3335643
C    -2.8383798    3.5116171   -2.4469996
H    -3.6277622    4.2452663   -2.5553827
C    -1.5106001    3.9106837   -2.4422007
H    -1.2574860    4.9576179   -2.5592714
P    -0.5656886   -0.2996977    0.9028507
C    -0.8653091   -2.0624281    0.9710458
C    -1.8385568   -2.5961833    0.1216508
H    -2.4185697   -1.9425466   -0.5216227
C    -2.0745788   -3.9585532    0.1104915
H    -2.8339076   -4.3691133   -0.5439069
C    -1.3349925   -4.7978089    0.9349119
H    -1.5177135   -5.8655080    0.9214496
C    -0.3655585   -4.2717934    1.7754098
H     0.2060175   -4.9259793    2.4225167
C    -0.1270103   -2.9063044    1.7980135
H     0.6265190   -2.4971250    2.4597575
C    -2.0229475    0.5841597    1.4461275
C    -3.0465272   -0.0578055    2.1418681
H    -2.9723910   -1.1146740    2.3681213
C    -4.1584679    0.6633068    2.5457068
H    -4.9529752    0.1652692    3.0880075
C    -4.2510955    2.0183864    2.2618869
H    -5.1228823    2.5777364    2.5796154
C    -3.2323350    2.6593481    1.5691454
H    -3.3080853    3.7151863    1.3400489
C    -2.1219918    1.9464137    1.1548840
H    -1.3399110    2.4468255    0.5945833
C     0.8250593    0.1307398    1.9437491
C     0.6608174    0.9293461    3.0752331
H    -0.3247382    1.2818173    3.3542779
C     1.7634562    1.2666077    3.8435688
H     1.6340692    1.8840226    4.7241552
C     3.0259220    0.8124288    3.4902508
H     3.8846526    1.0798140    4.0943180
C     3.1922203    0.0179456    2.3630256
H     4.1768909   -0.3334756    2.0799068
C     2.0985130   -0.3199441    1.5866646
H     2.2412615   -0.9292578    0.7011539
```

Ph3PH+

```
E(PBE0-D3/def2-TZVP) = -1036.022582340 (conv) Lowest Freq. =    18.20 cm^-1
35
Ph3PHcat (151c1/opt)
```

```
P     0.8961390    0.1035905    0.1562562
C     0.0677854    1.4631962    0.9479093
C     0.5796707    2.7525684    0.7920008
H     1.4875410    2.9231851    0.2231201
C    -0.0824569    3.8192744    1.3714261
H     0.3111513    4.8220012    1.2605630
C    -1.2472068    3.6017161    2.0976675
H    -1.7609570    4.4406434    2.5515259
C    -1.7538089    2.3195466    2.2506975
H    -2.6596069    2.1560340    2.8211821
C    -1.0979234    1.2418692    1.6777888
H    -1.4846109    0.2372337    1.8025107
C     0.4201984   -1.4582035    0.8604207
C    -0.4187058   -2.3168608    0.1536359
H    -0.7535692   -2.0583638   -0.8440533
C    -0.8140092   -3.5100861    0.7365254
H    -1.4645875   -4.1840736    0.1932462
C    -0.3723573   -3.8407911    2.0091364
H    -0.6803456   -4.7770788    2.4588484
C     0.4668164   -2.9835683    2.7108584
H     0.8128259   -3.2502241    3.7017442
C     0.8658667   -1.7883347    2.1415402
H     1.5189538   -1.1183565    2.6907296
C     0.6668451    0.1285329   -1.6067703
C    -0.3816355    0.8563174   -2.1649758
H    -1.0384734    1.4437089   -1.5342437
C    -0.5709307    0.8284086   -3.5372754
H    -1.3824748    1.3927892   -3.9794728
C     0.2805253    0.0842092   -4.3404870
H     0.1313817    0.0692934   -5.4134515
C     1.3275299   -0.6385754   -3.7811770
H     1.9923920   -1.2128977   -4.4142633
C     1.5251572   -0.6214990   -2.4127493
H     2.3411512   -1.1878166   -1.9763740
H     2.2717288    0.2626119    0.3959598
```

PhTHQ.

```
E(PBE0-D3/def2-TZVP) = -634.2668215662 (conv) Lowest Freq. =    40.74 cm^-1
30
PhTHQ. (154c1/opt)
C     1.8411239   -0.1136470    0.1119372
C     3.0578035    0.5023491   -0.1815011
C     4.2473152   -0.1486371    0.0881973
C     4.2396432   -1.4220028    0.6419472
C     3.0253959   -2.0292733    0.9308859
C     1.8204948   -1.3921481    0.6842825
C     0.4888330   -1.9893353    1.0171667
C    -0.5718749   -1.5489127    0.0104465
C    -0.5847519   -0.0659238   -0.1377510
N     0.6485021    0.5345178   -0.1407060
H     3.0636456    1.4924420   -0.6265809
H     5.1867059    0.3393561   -0.1439444
H     5.1699922   -1.9375885    0.8451726
H     3.0087634   -3.0242657    1.3646175
H     0.1816609   -1.6660964    2.0193611
H     0.5583619   -3.0793500    1.0440352
H    -1.5531061   -1.9017988    0.3256462
H    -0.3565127   -2.0197882   -0.9600867
C    -1.7121734    2.1362012   -0.2778127
H    -0.7963647    2.6517740   -0.0110609
H     0.7107085    1.4686900   -0.5095689
C    -1.7471577    0.7206358   -0.3284765
```

```
C    -3.0021229    0.1262471   -0.6012852
H    -3.0814032   -0.9514042   -0.6679511
C    -4.1271827    0.8922154   -0.8163623
H    -5.0696825    0.4001807   -1.0293912
C    -4.0658697    2.2828541   -0.7721092
H    -4.9530536    2.8798997   -0.9427309
C    -2.8458384    2.8908994   -0.4968790
H    -2.7818556    3.9719092   -0.4394978
```

Ph3PH.

```
E(PBE0-D3/def2-TZVP) = -1036.168177436 (conv) Lowest Freq. =    35.14 cm^-1
35
Ph3PH. (152c1/opt)
P     0.7777863    0.0309019    0.0410437
C     0.2763687    1.4314038    1.0279269
C     1.1568522    2.5113365    1.1801704
H     2.1725348    2.4307752    0.8048831
C     0.7431922    3.6703703    1.8109668
H     1.4415331    4.4901184    1.9370881
C    -0.5587043    3.7876139    2.2826465
H    -0.8842555    4.7007501    2.7660007
C    -1.4474978    2.7273190    2.1147458
H    -2.4664535    2.8135216    2.4748462
C    -1.0443429    1.5679352    1.4848019
H    -1.7454672    0.7520598    1.3461319
C     0.1679931   -1.4433297    0.8116151
C     0.0940704   -2.6502901    0.0898196
H     0.3187340   -2.6553505   -0.9697173
C    -0.2697032   -3.8223881    0.7207198
H    -0.3379811   -4.7369638    0.1425134
C    -0.5401396   -3.8408807    2.0863245
H    -0.8187159   -4.7652133    2.5773344
C    -0.4240969   -2.6619363    2.8176840
H    -0.6130390   -2.6658513    3.8852587
C    -0.0615449   -1.4821598    2.2004813
H     0.0415962   -0.5767407    2.7859072
C     0.4830709   -0.0869769   -1.7152212
C    -0.7651600   -0.4834028   -2.2218451
H    -1.5281829   -0.8455874   -1.5414119
C    -1.0185476   -0.4225755   -3.5766895
H    -1.9820367   -0.7446624   -3.9552572
C    -0.0504222    0.0567611   -4.4572634
H    -0.2591536    0.1141511   -5.5186545
C     1.1785251    0.4750755   -3.9613112
H     1.9374659    0.8501259   -4.6387376
C     1.4423008    0.4159255   -2.6051449
H     2.4010453    0.7538868   -2.2236724
H     2.1823760    0.3042775   -0.0039837
```

Ph3P

```
E(PBE0-D3/def2-TZVP) = -1035.635020045 (conv) Lowest Freq. =    25.93 cm^-1
34
Ph3P (158c1/opt)
P     0.0000000   -0.0000000    1.4257010
C    -1.5140142    0.6226722    0.6112737
C    -2.1030909    1.7636051    1.1570680
H    -1.6599338    2.2243714    2.0342549
C    -3.2422430    2.3118955    0.5909892
H    -3.6843800    3.2023703    1.0230175
C    -3.8229827    1.7132286   -0.5186709
H    -4.7202394    2.1346849   -0.9569774
C    -3.2550887    0.5692119   -1.0586402
```

H	-3.7062529	0.0947922	-1.9228355
C	-2.1053906	0.0285777	-0.5006399
H	-1.6642510	-0.8617973	-0.9330416
C	0.2177571	-1.6225109	0.6112737
C	1.0279463	-1.8376105	-0.5006399
H	1.5784638	-1.0103850	-0.9330416
C	1.1345924	-3.1035954	-1.0586402
H	1.7710340	-3.2571053	-1.9228355
C	0.4277919	-4.1674145	-0.5186709
H	0.5114283	-5.1551897	-0.9569774
C	-0.3810387	-3.9638126	0.5909892
H	-0.9311441	-4.7919518	1.0230175
C	-0.4757814	-2.7031327	1.1570680
H	-1.0963952	-2.5497305	2.0342549
C	1.2962571	0.9998387	0.6112737
C	1.0774443	1.8090329	-0.5006399
H	0.0857872	1.8721823	-0.9330416
C	2.1204963	2.5343836	-1.0586402
H	1.9352189	3.1623131	-1.9228355
C	3.3951909	2.4541858	-0.5186709
H	4.2088111	3.0205048	-0.9569774
C	3.6232818	1.6519171	0.5909892
H	4.6155240	1.5895815	1.0230175
C	2.5788722	0.9395276	1.1570680
H	2.7563290	0.3253591	2.0342549

16. NMR spectra

^{1}H NMR (300 MHz, $CDCl_3$) and ^{13}C NMR (76 MHz, $CDCl_3$) spectra for **1i**

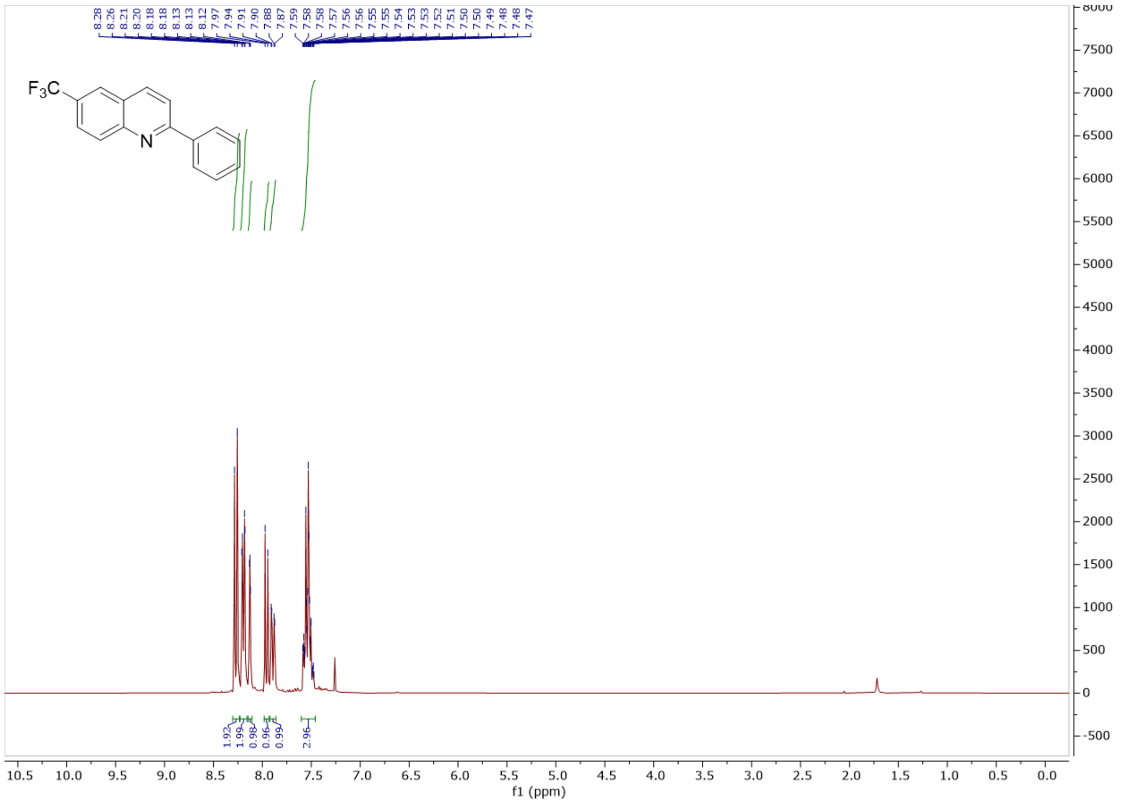

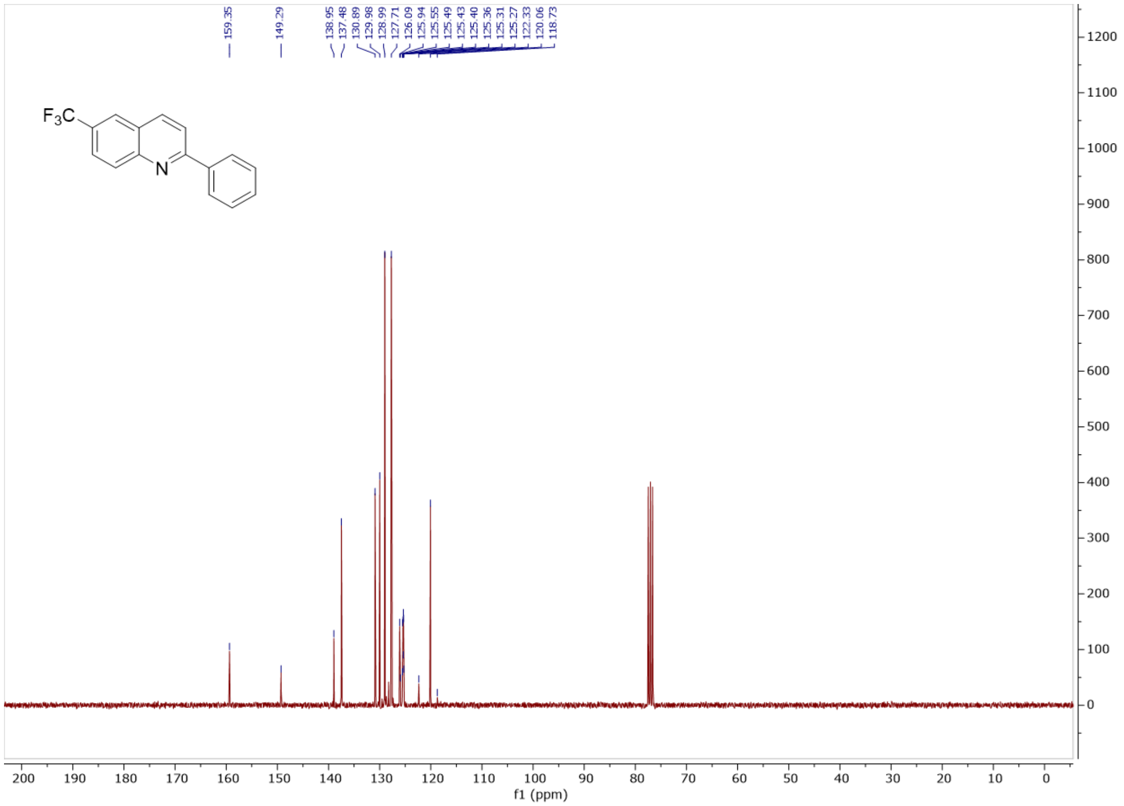

^{1}H NMR (400 MHz, $CDCl_3$) and ^{13}C NMR (101 MHz, $CDCl_3$) spectra for **1k**

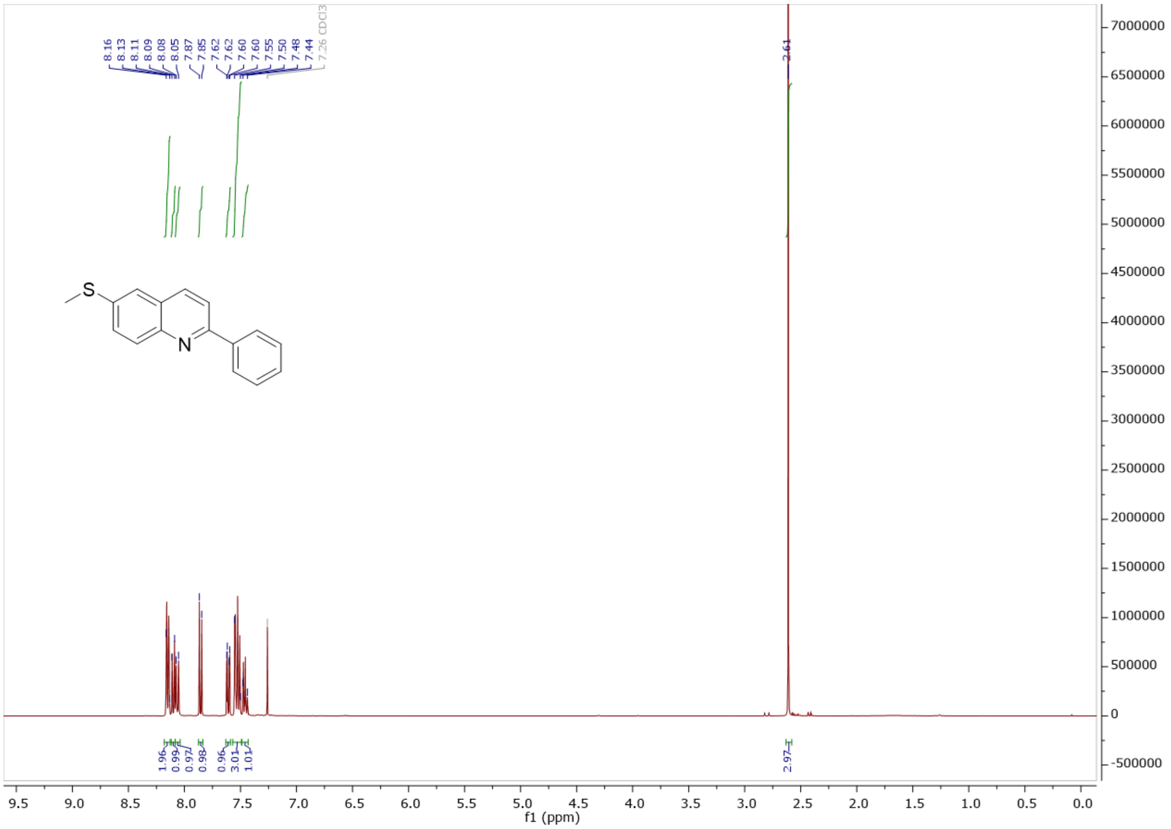

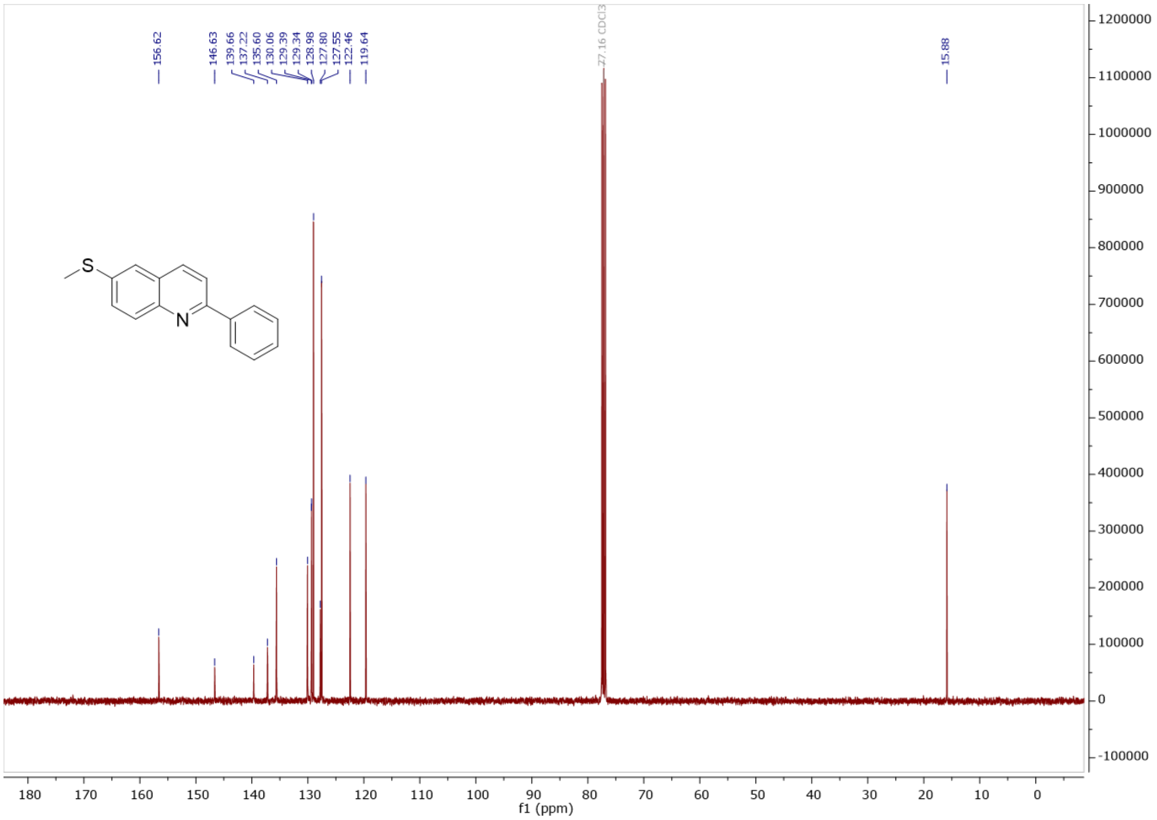

^{1}H NMR (300 MHz, $CDCl_3$) and ^{13}C NMR (76 MHz, $CDCl_3$) spectra for **1m**

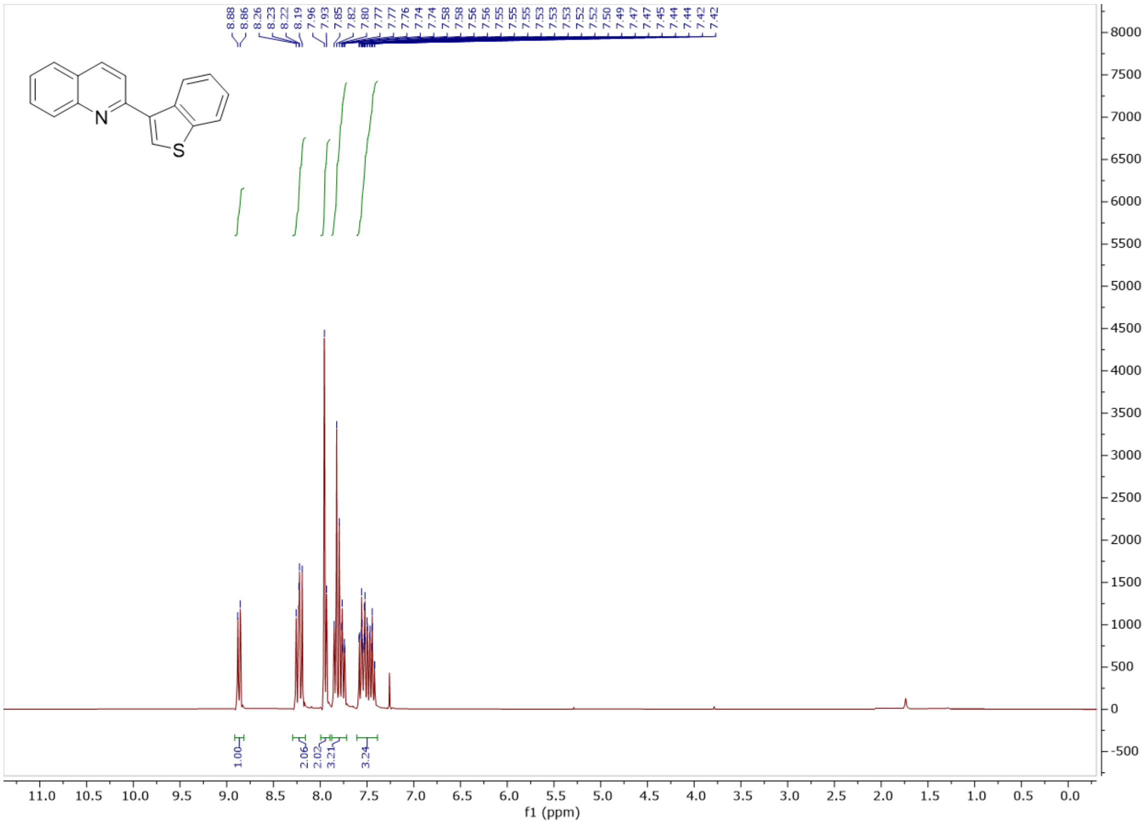

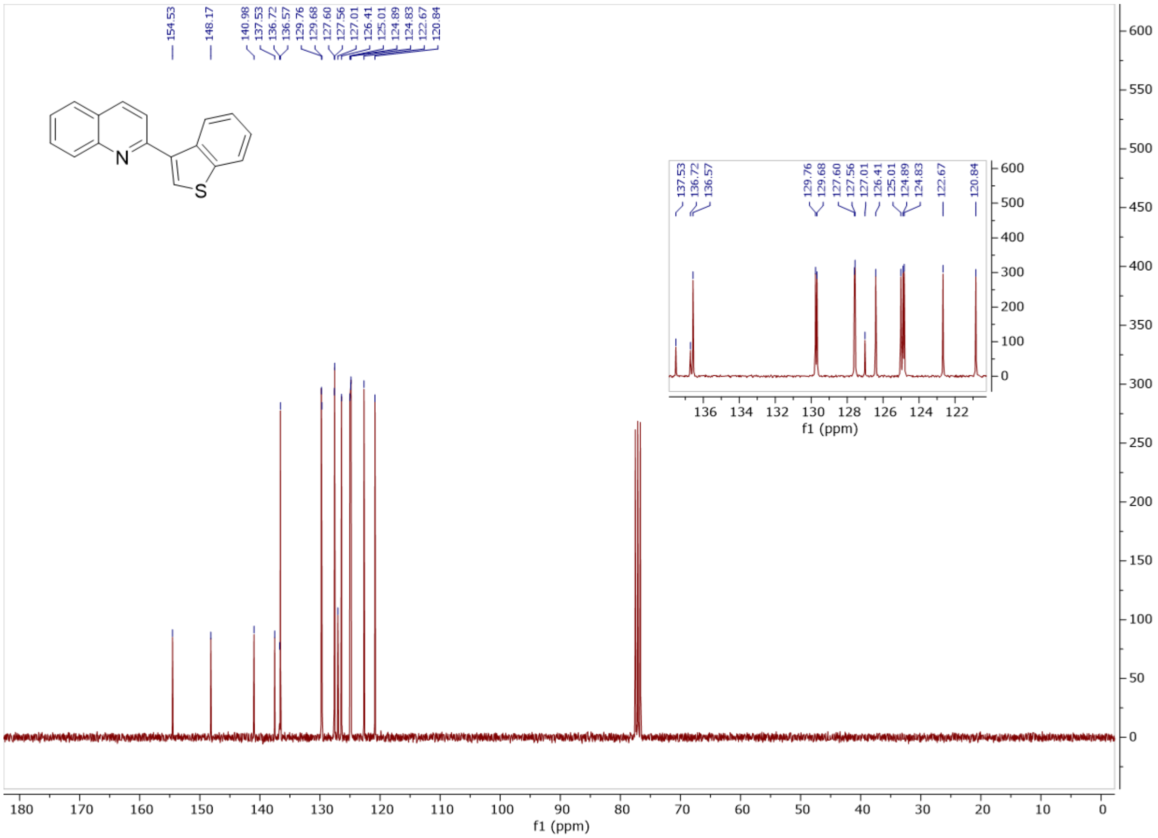

^{1}H NMR (300 MHz, $CDCl_3$) and ^{13}C NMR (76 MHz, $CDCl_3$) spectra for **1w**

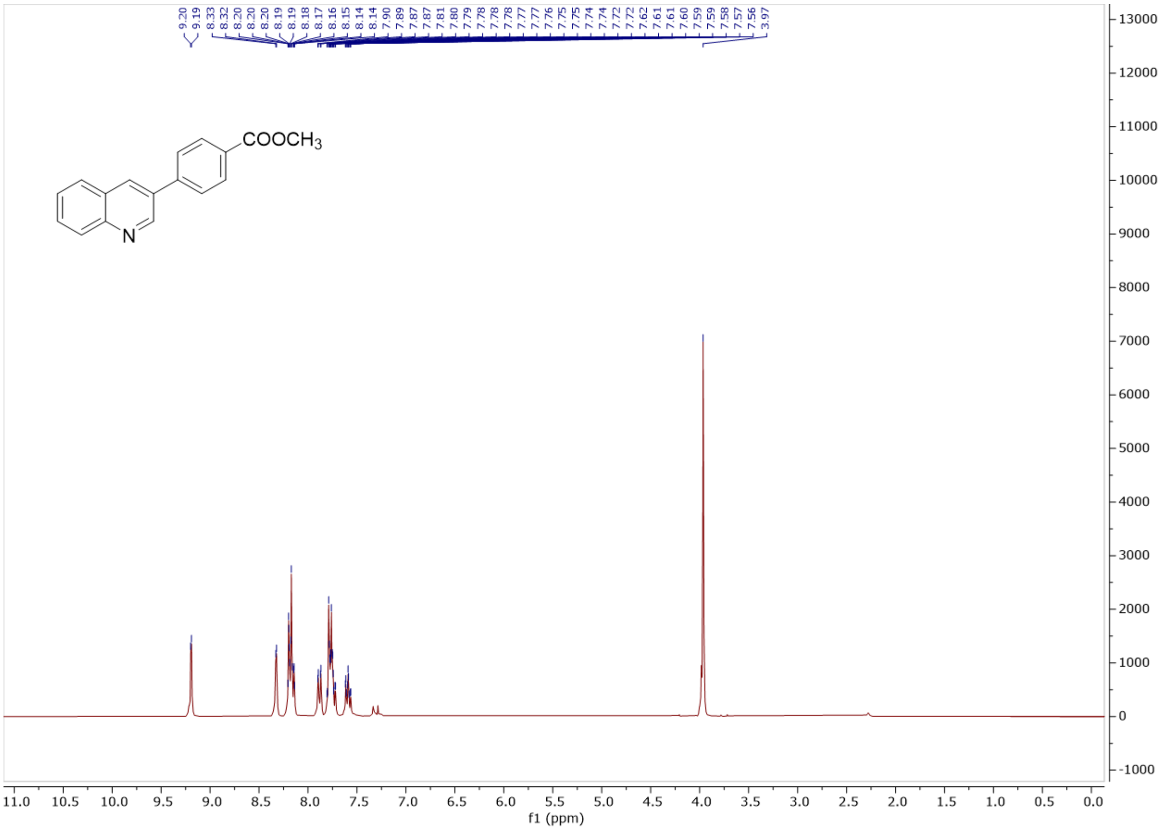

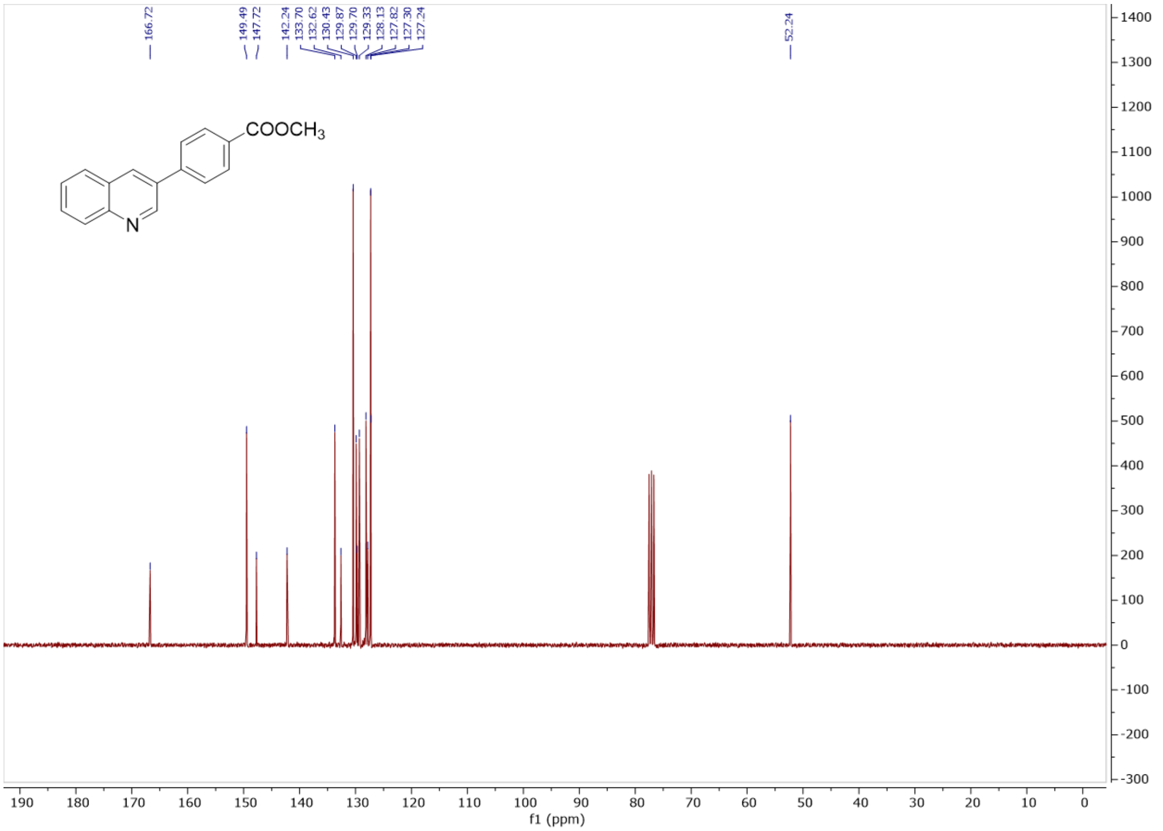

^{1}H NMR (300 MHz, $CDCl_3$) and ^{13}C NMR (76 MHz, $CDCl_3$) spectra for **1ab**

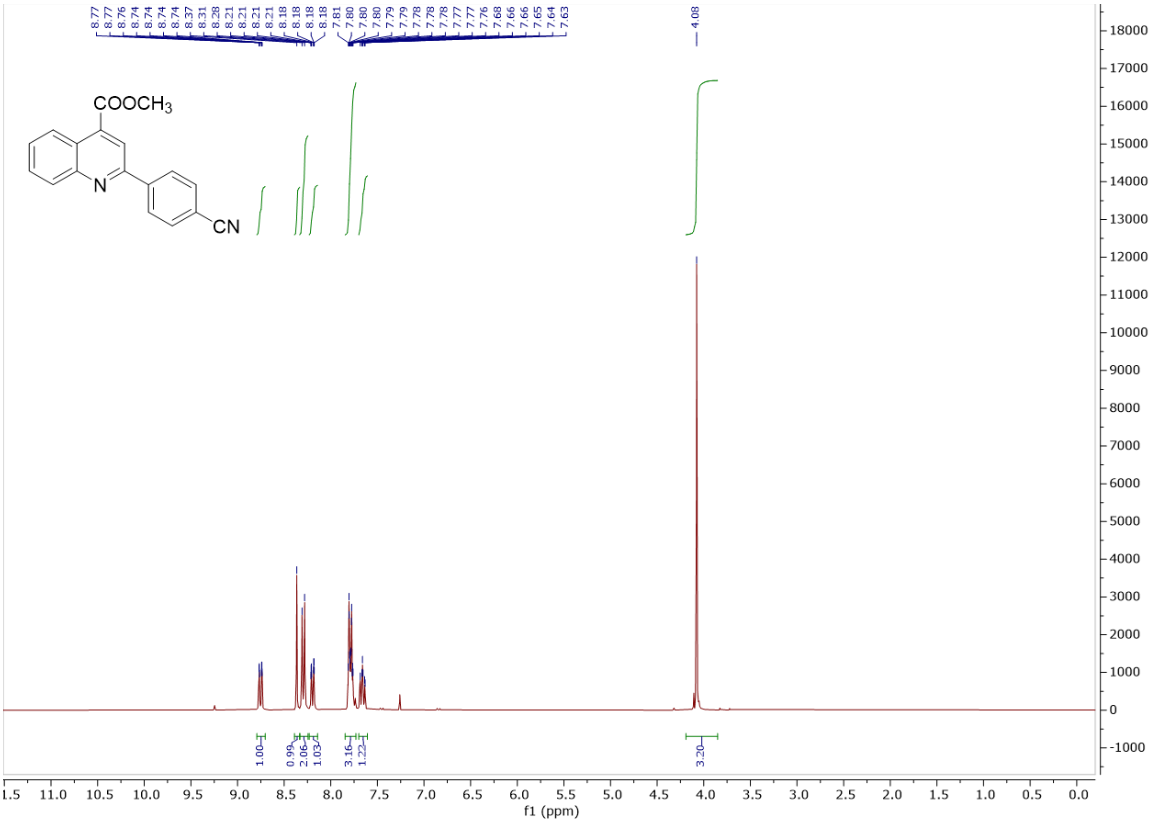

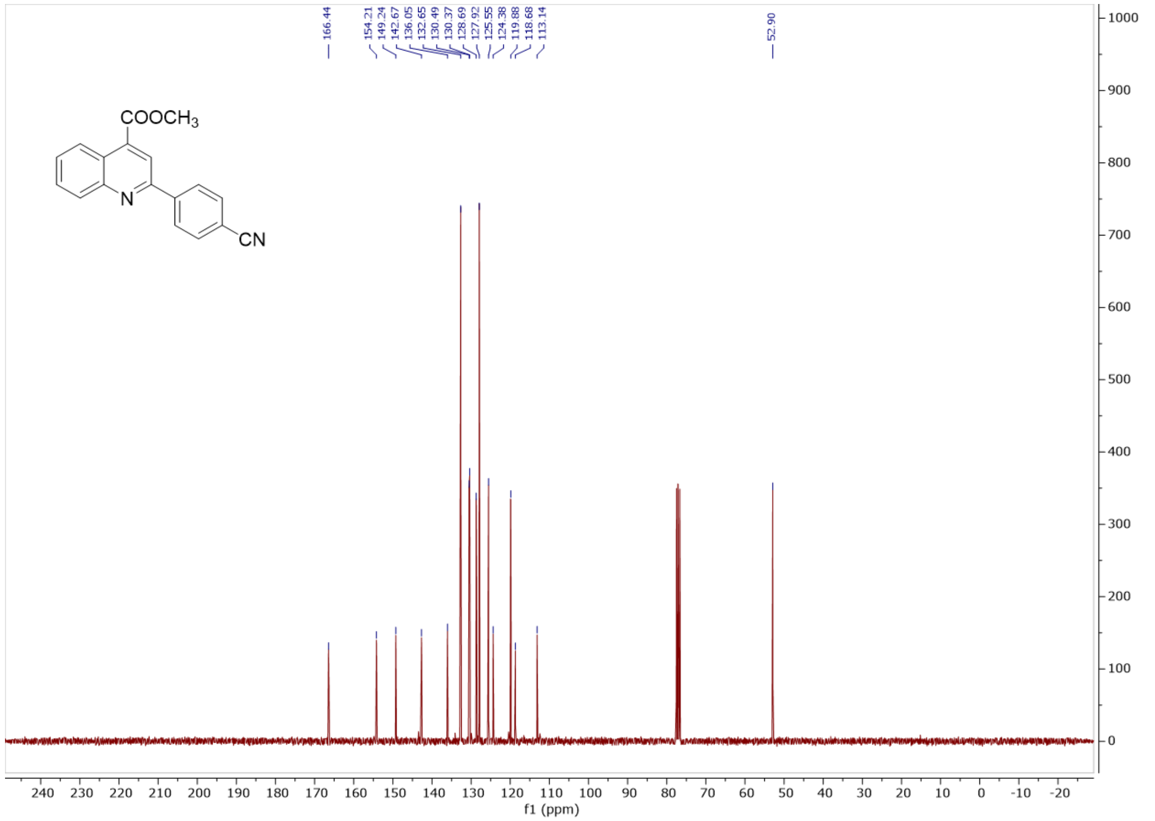

^1H NMR (300 MHz, $CDCl_3$) and ^{13}C NMR (76 MHz, $CDCl_3$) spectra for **1ad**

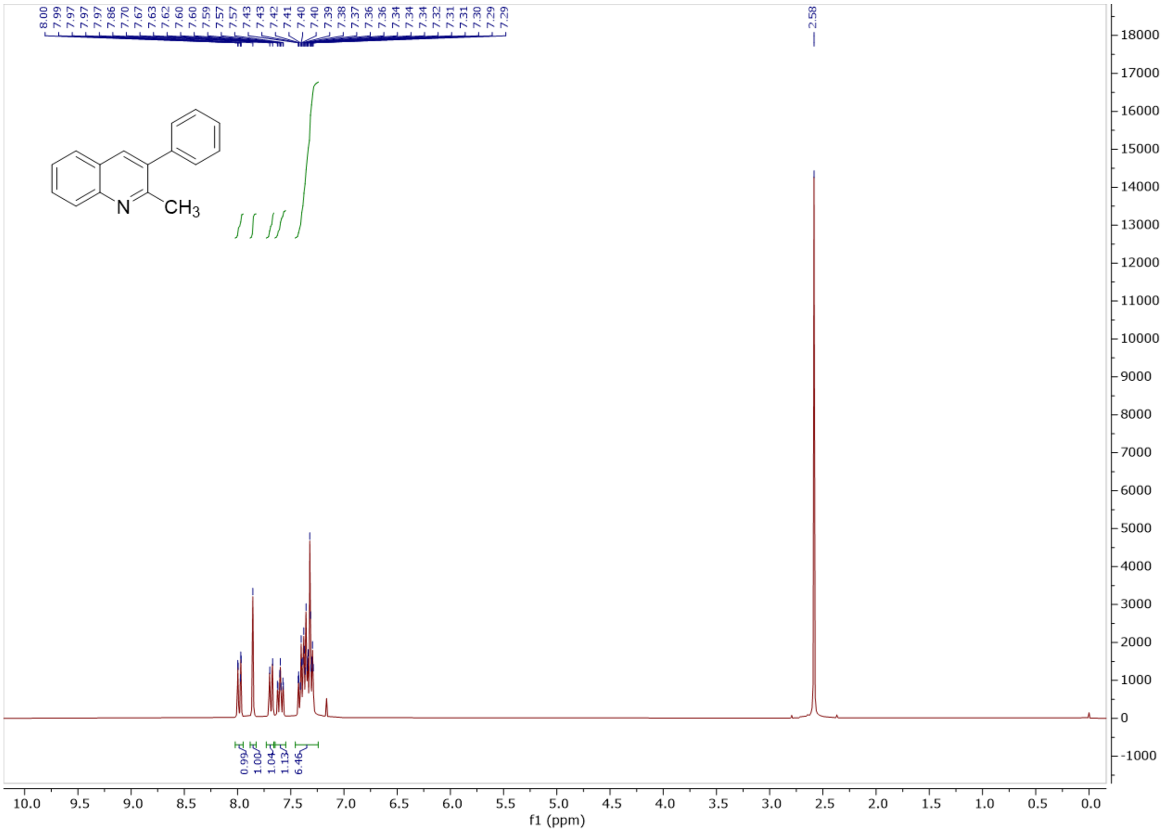

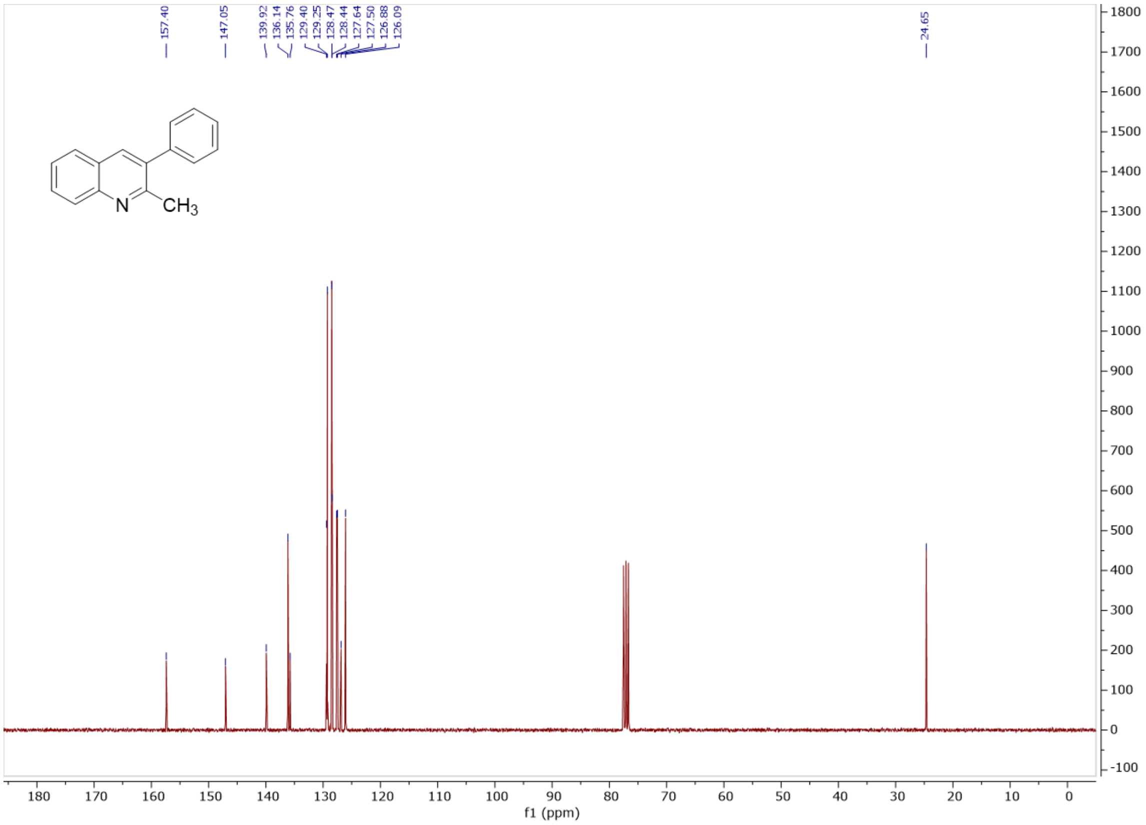

^{1}H NMR (300 MHz, $CDCl_3$) and ^{13}C NMR (76 MHz, $CDCl_3$) spectra for **1ae**

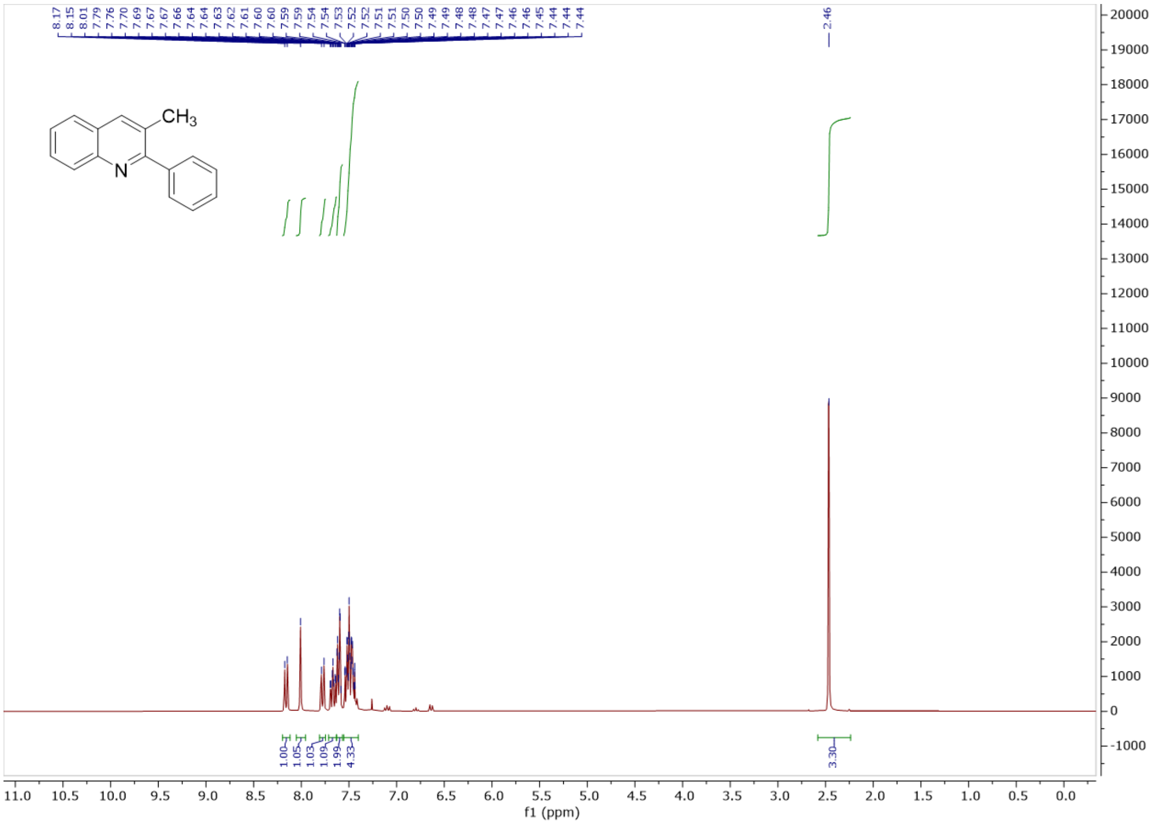

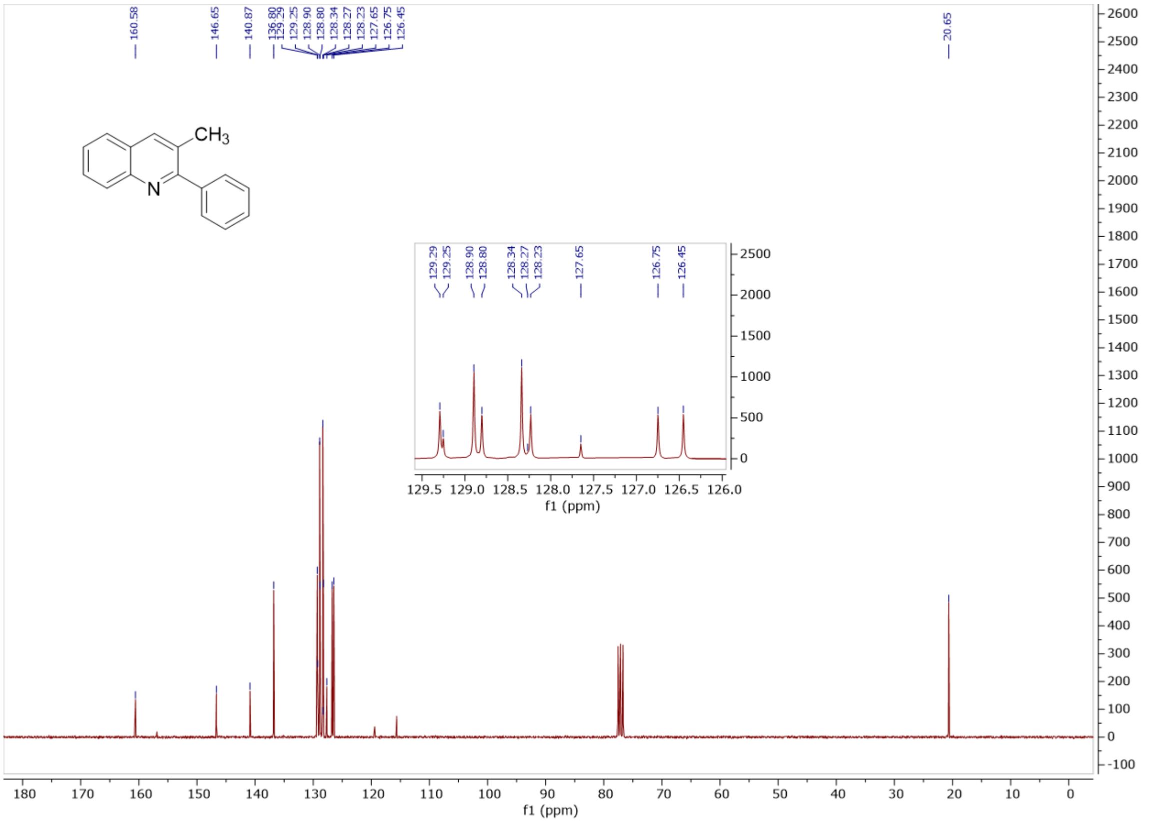

^{1}H NMR (300 MHz, $CDCl_3$) and ^{13}C NMR (76 MHz, $CDCl_3$) spectra for **1am**

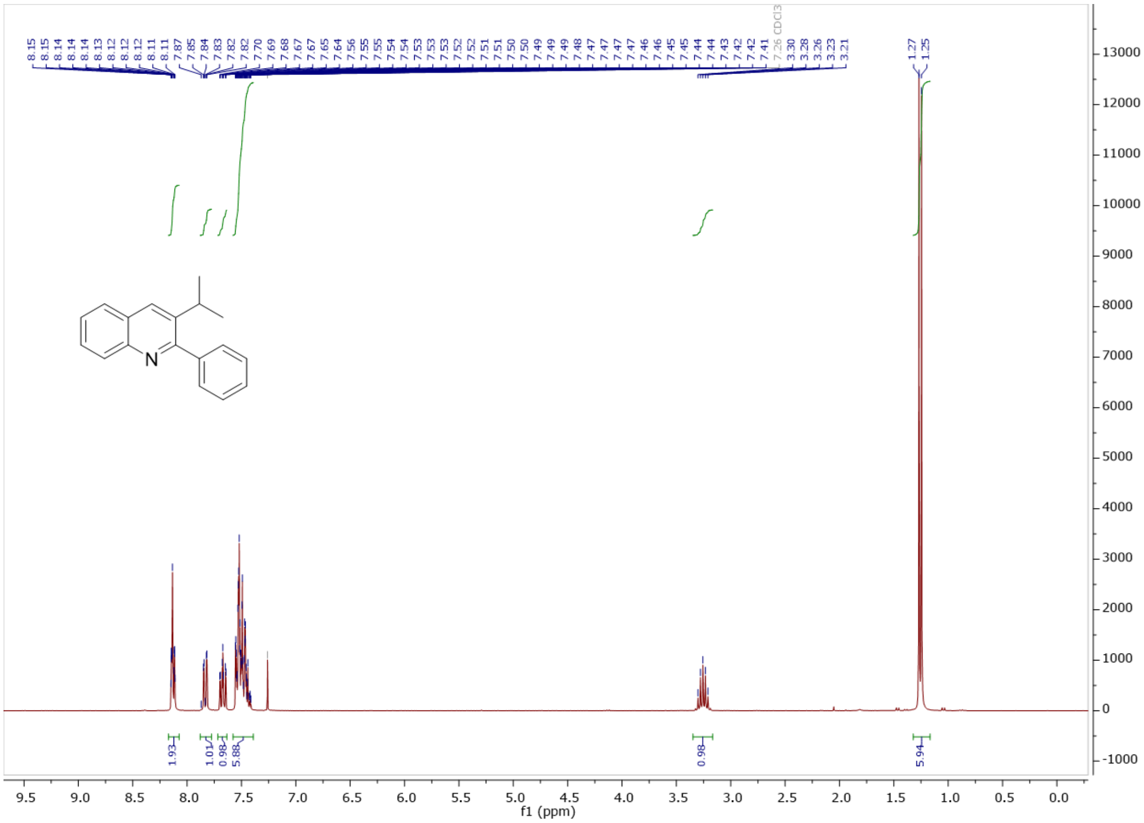

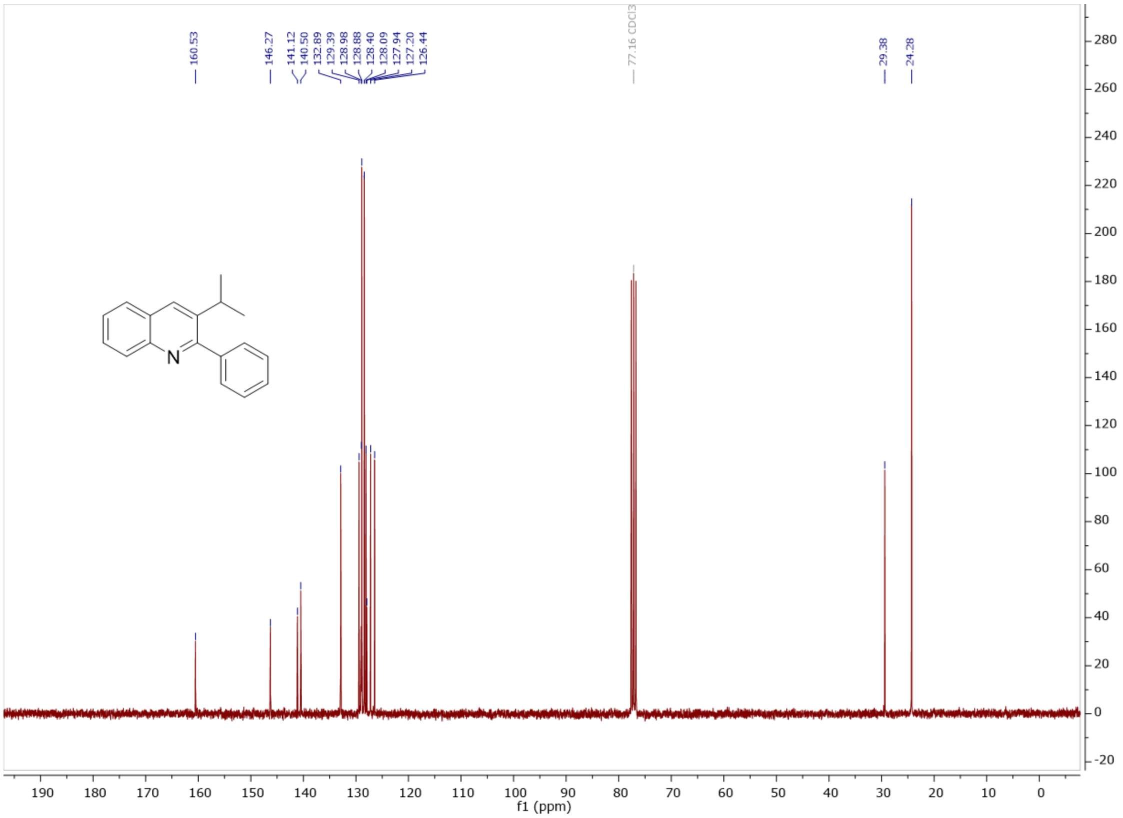

^{1}H NMR (300 MHz, $CDCl_3$) and ^{13}C NMR (76 MHz, $CDCl_3$) spectra for **1an**

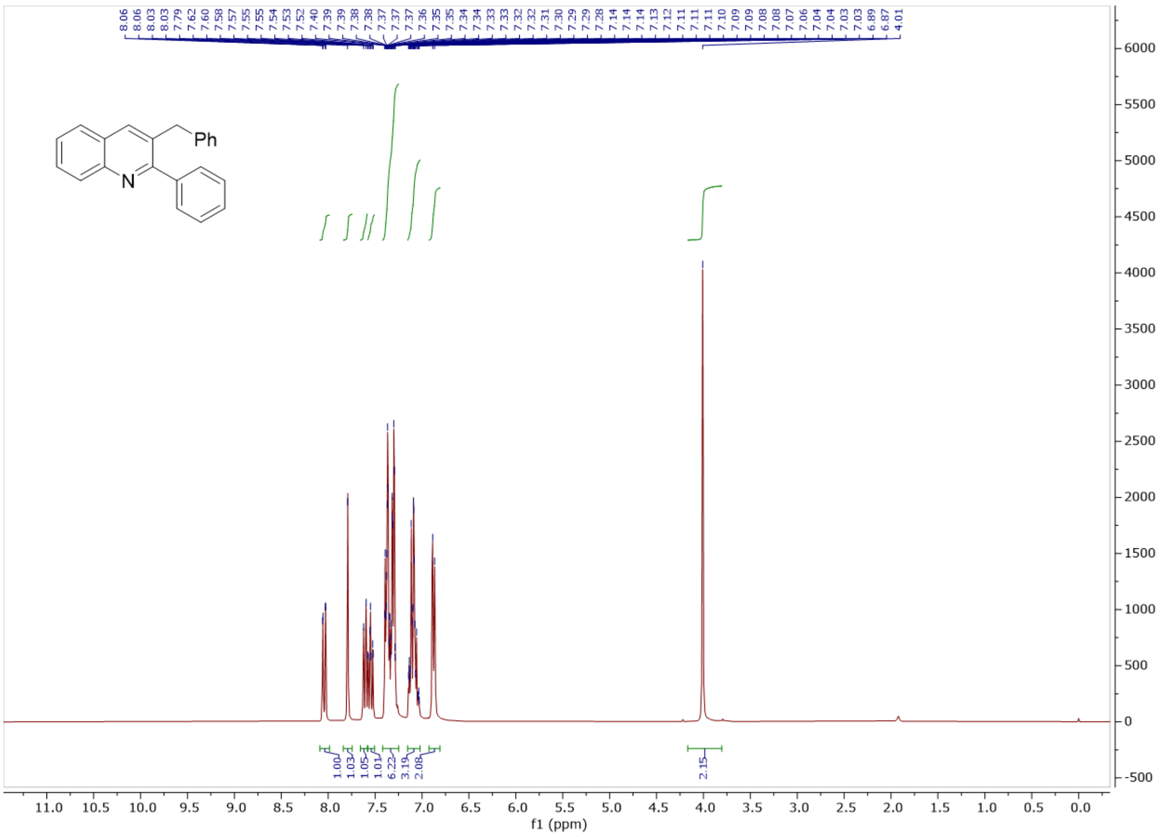

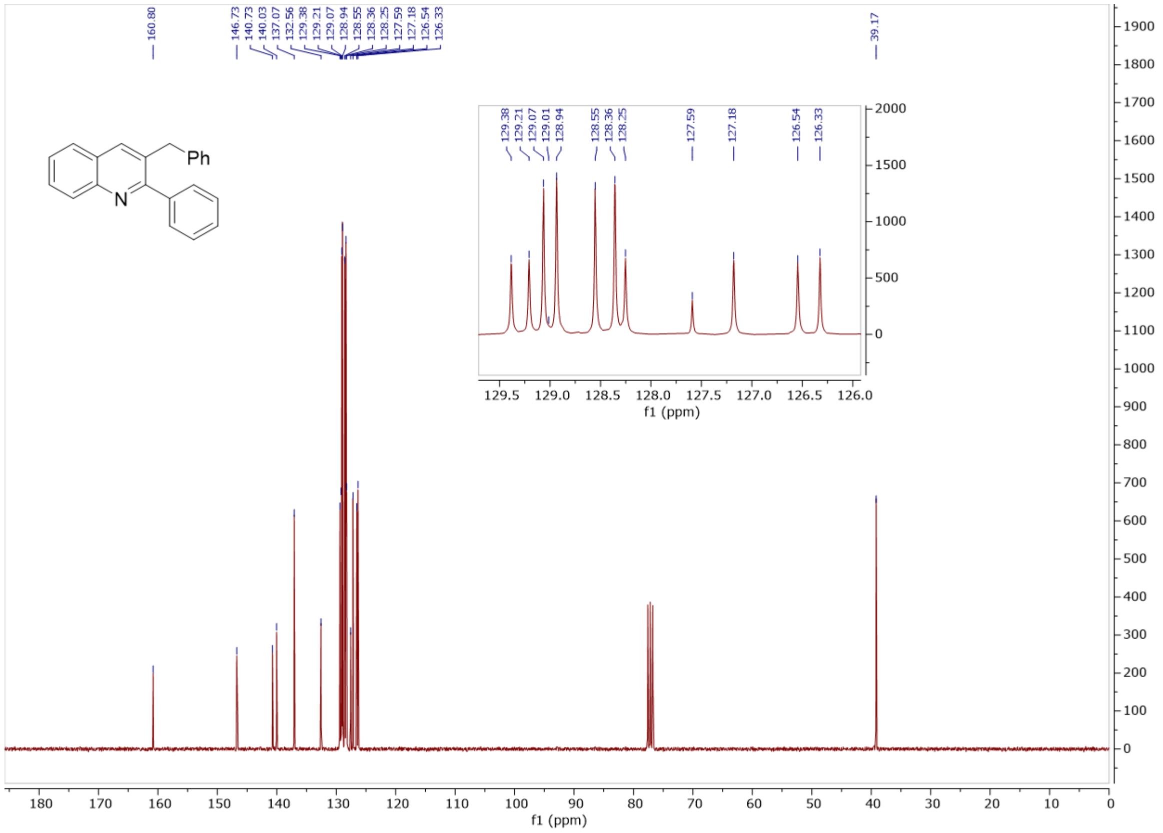

^{1}H NMR (300 MHz, $CDCl_3$) and ^{13}C NMR (76 MHz, $CDCl_3$) spectra for **1ao**

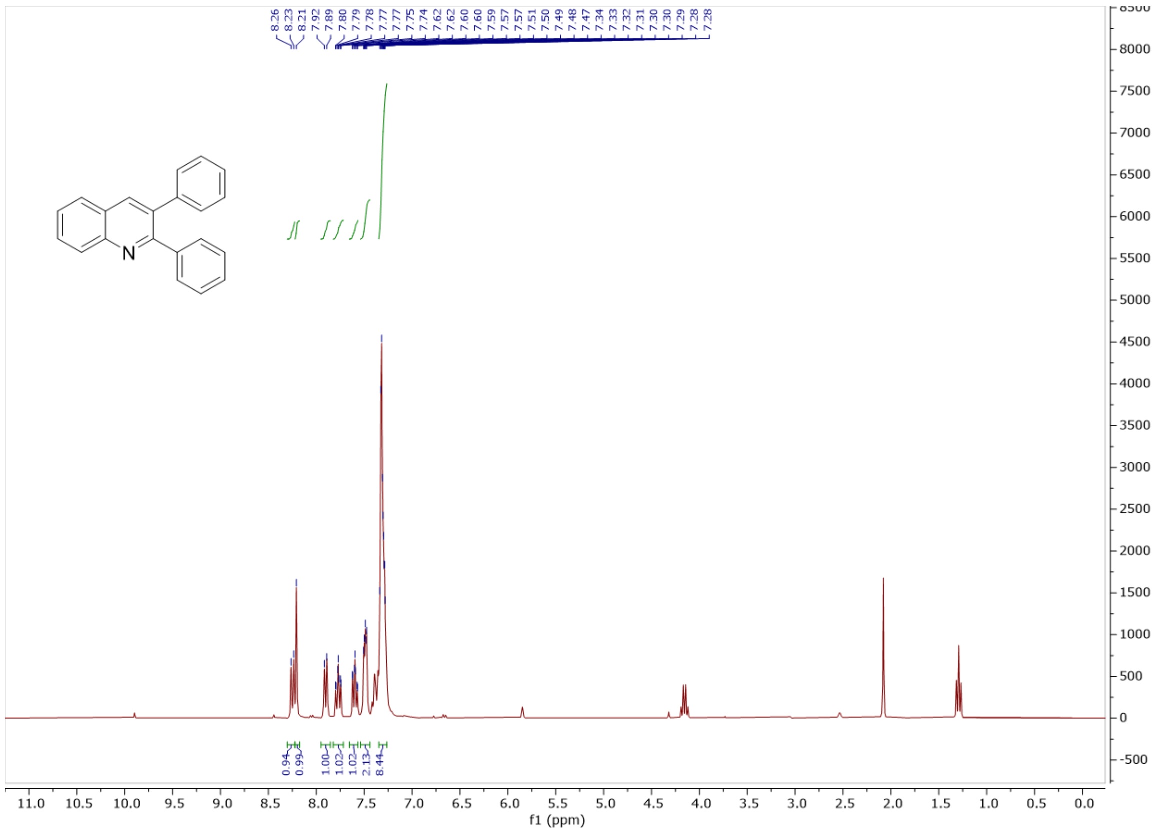

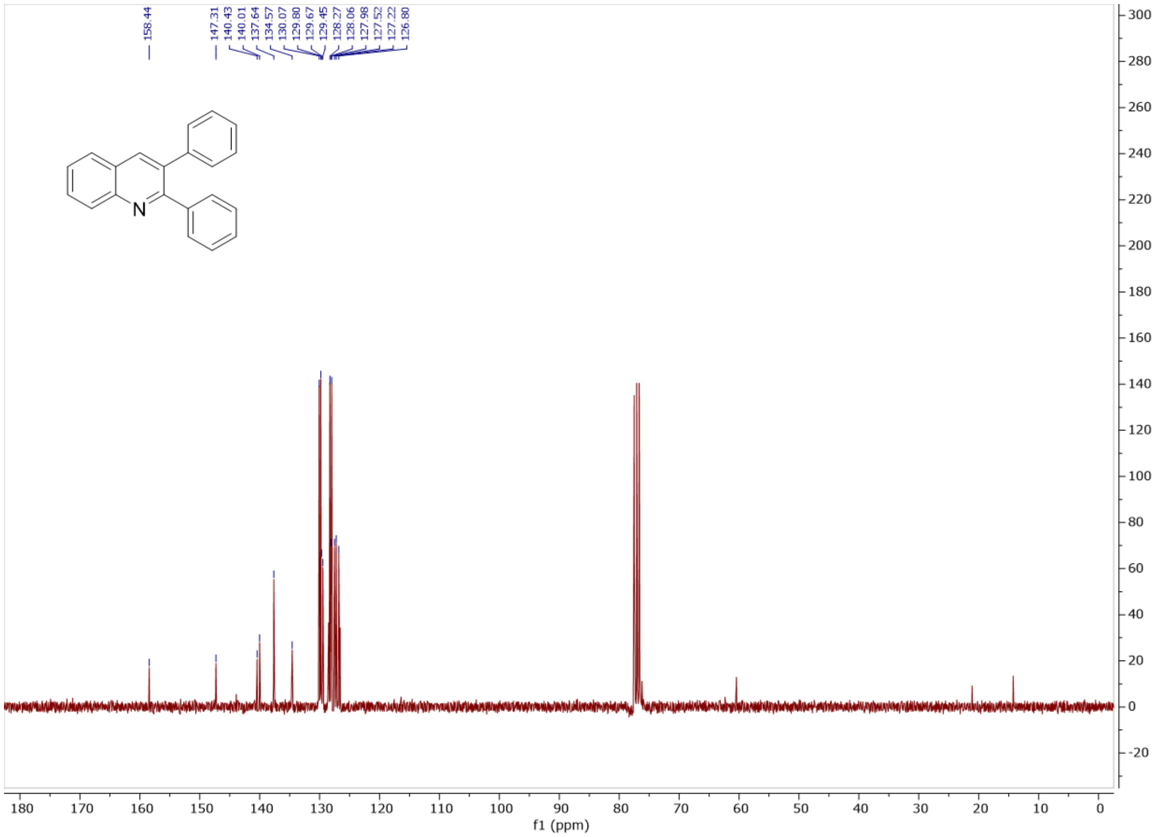

^{1}H NMR (300 MHz, $CDCl_3$) and ^{13}C NMR (76 MHz, $CDCl_3$) spectra for **1ap**

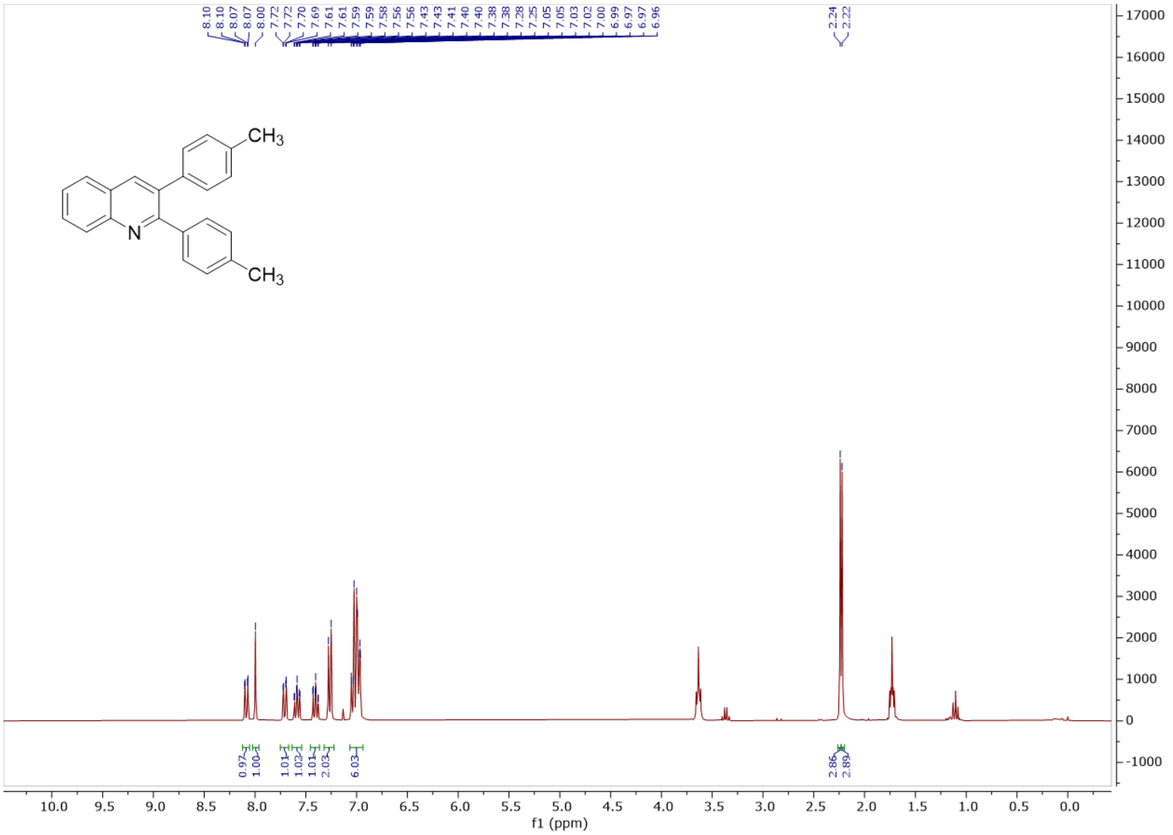

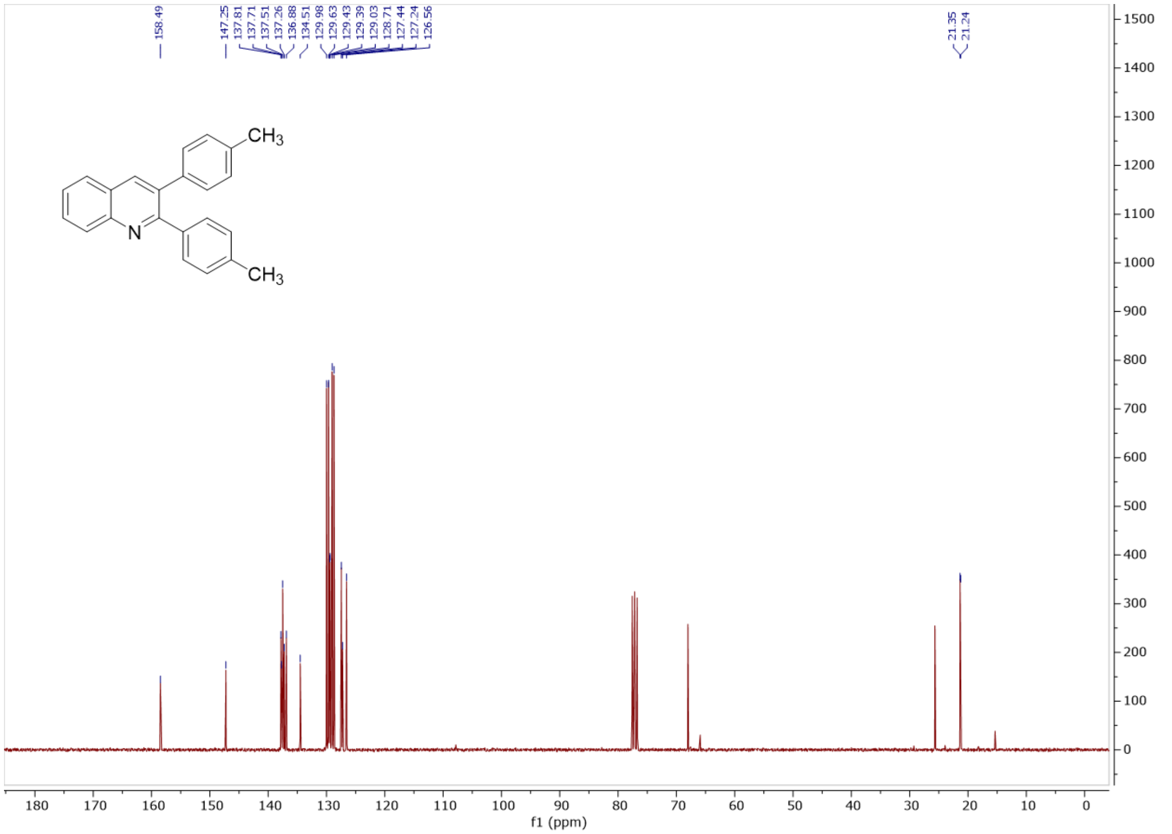

^{1}H NMR (300 MHz, $CDCl_3$) and ^{13}C NMR (76 MHz, $CDCl_3$) spectra for **1at**

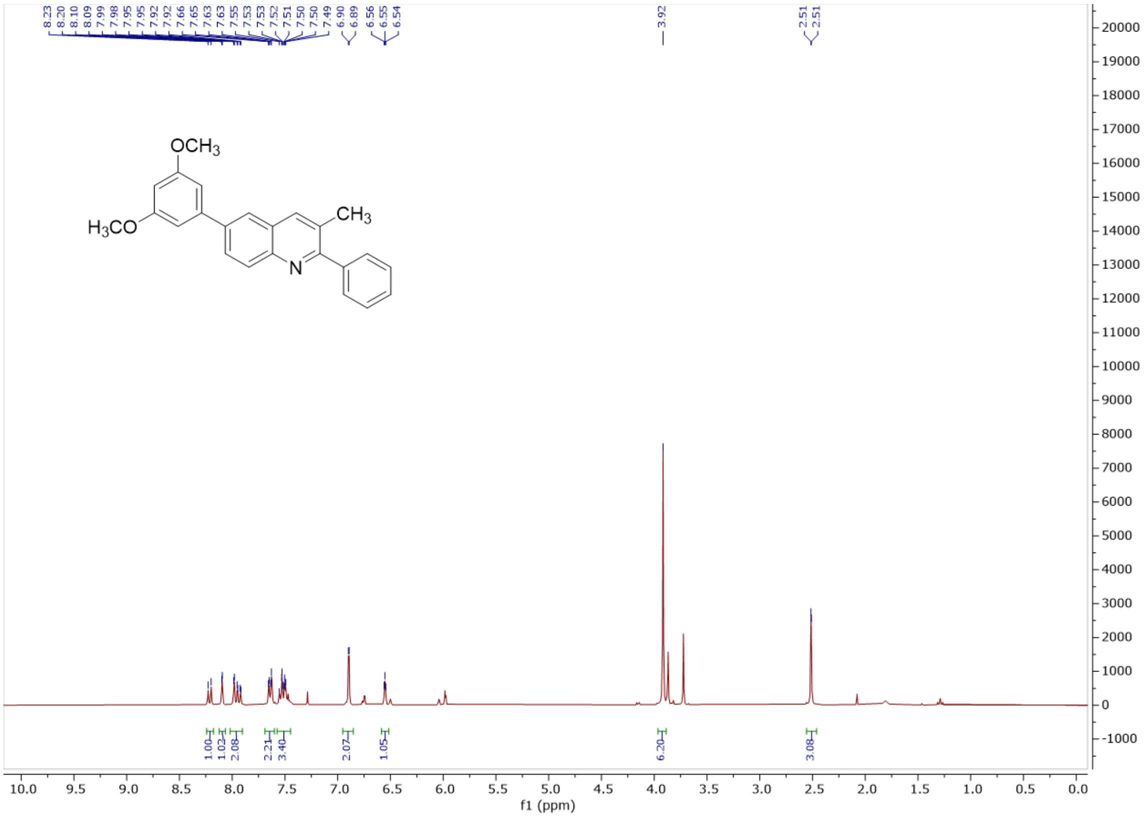

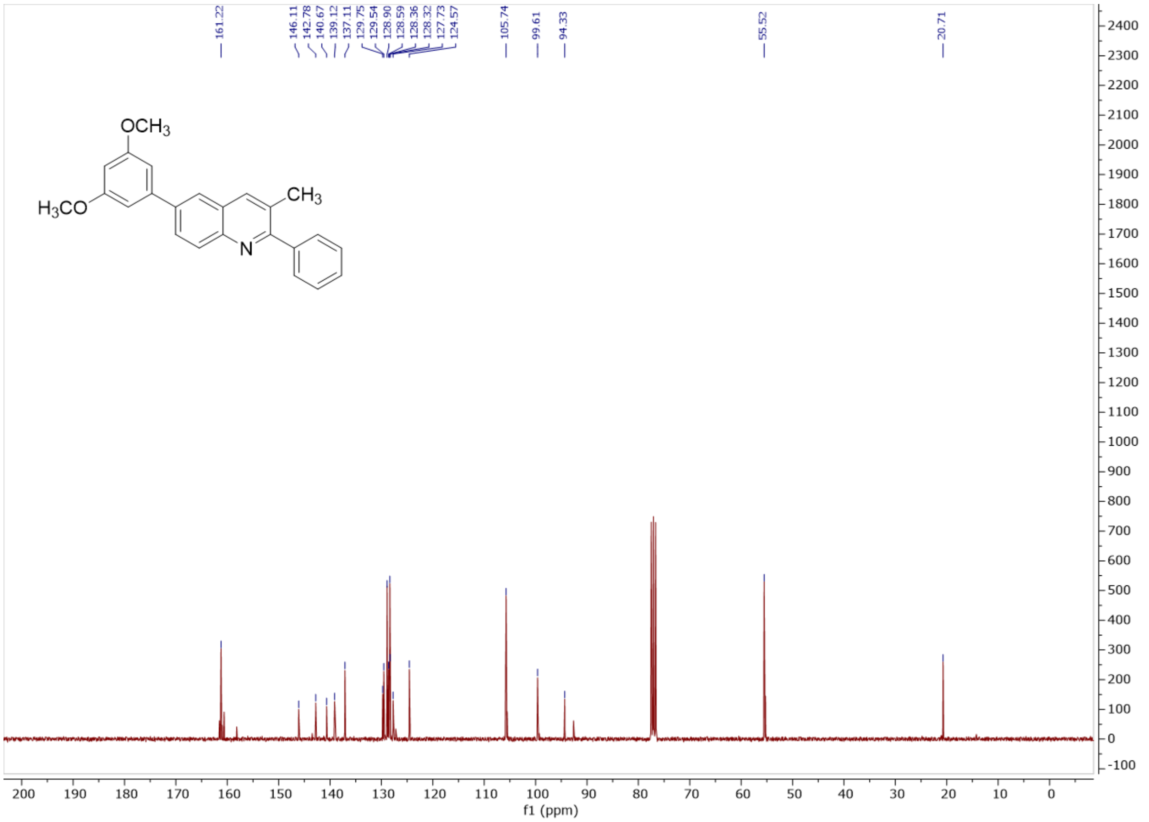

^{1}H NMR (300 MHz, $CDCl_3$) and ^{13}C NMR (76 MHz, $CDCl_3$) spectra for **2a'**

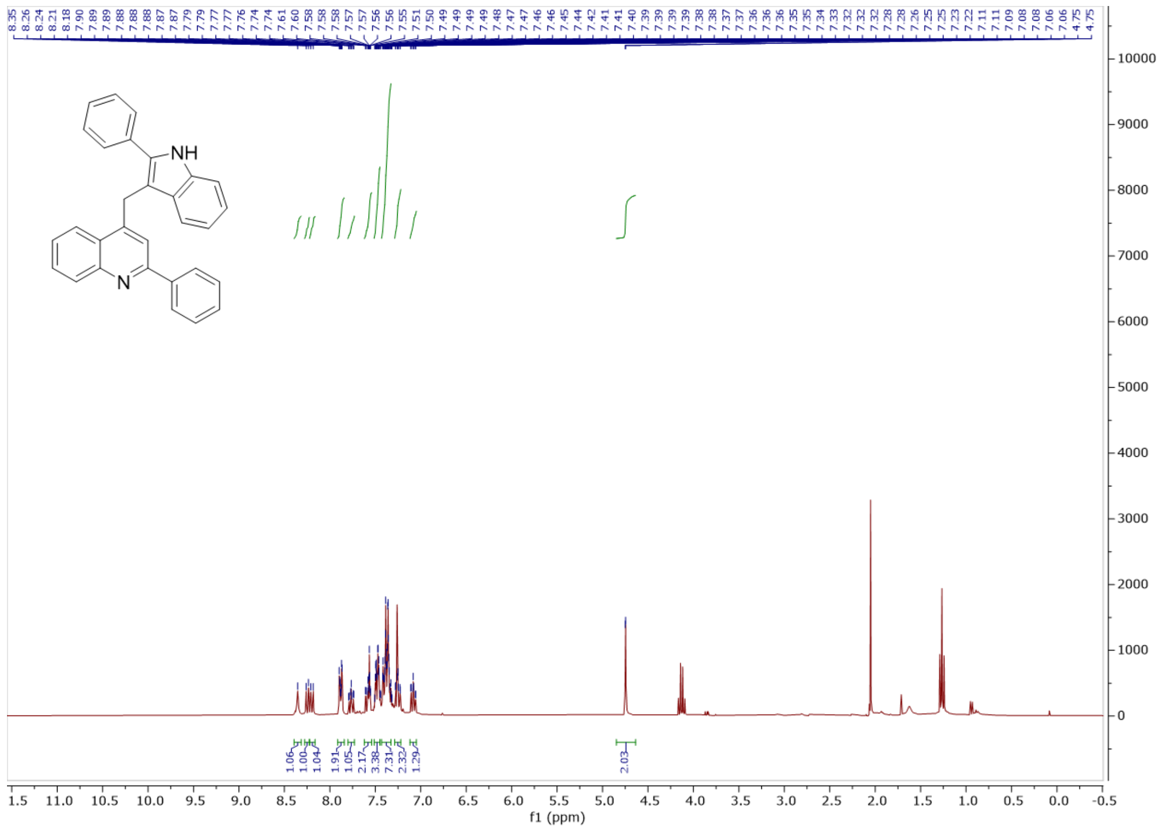

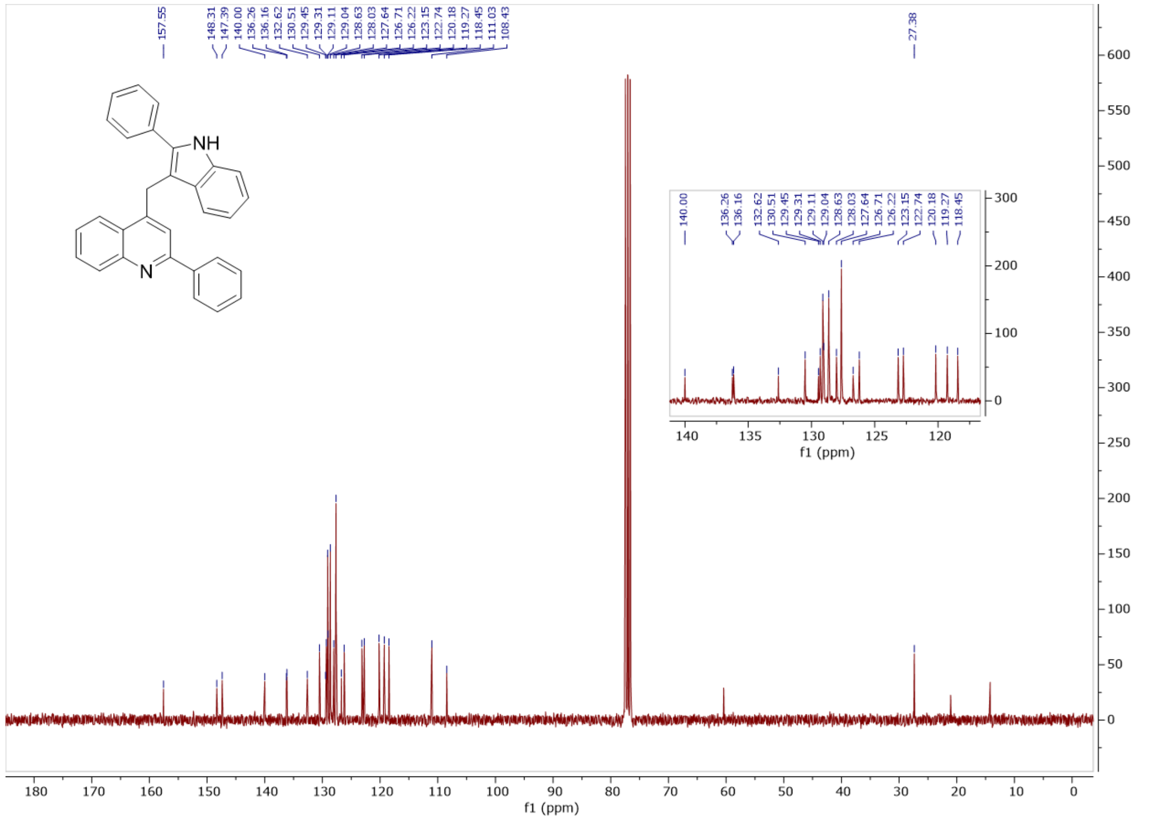

^{1}H NMR (300 MHz, $CDCl_3$) and ^{13}C NMR (76 MHz, $CDCl_3$) spectra for **2a**

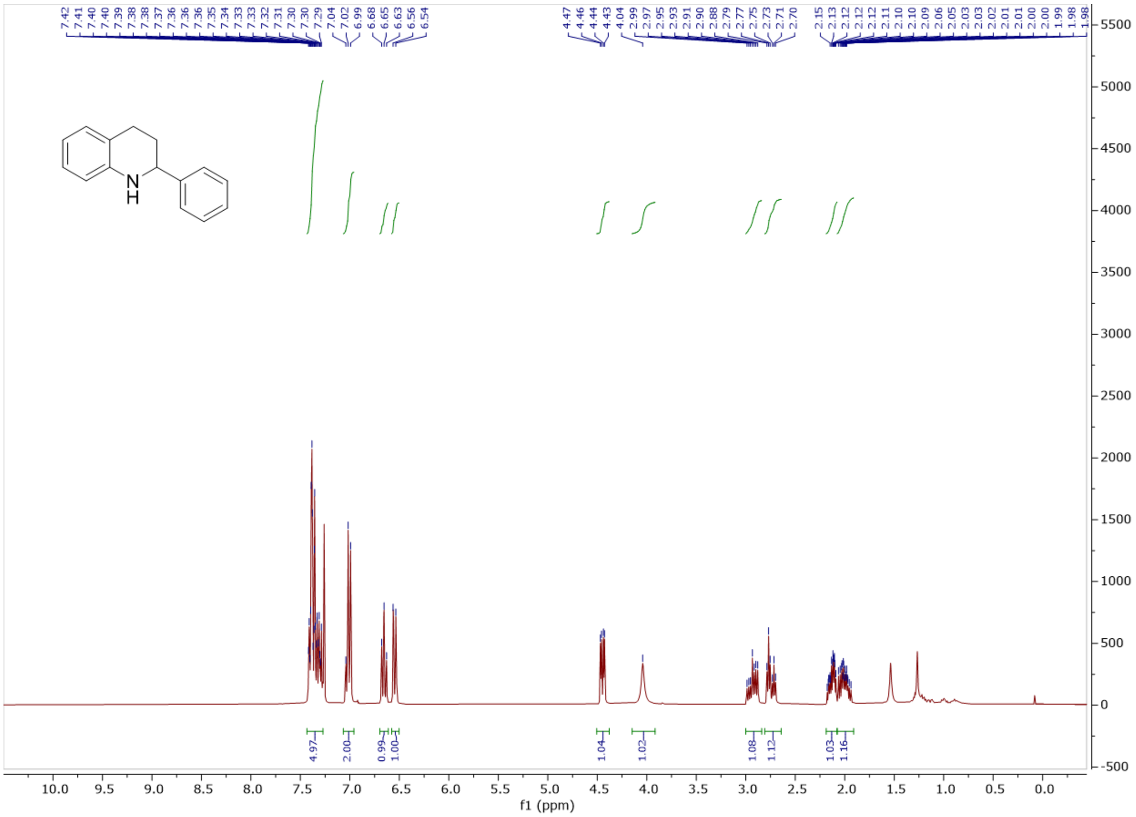

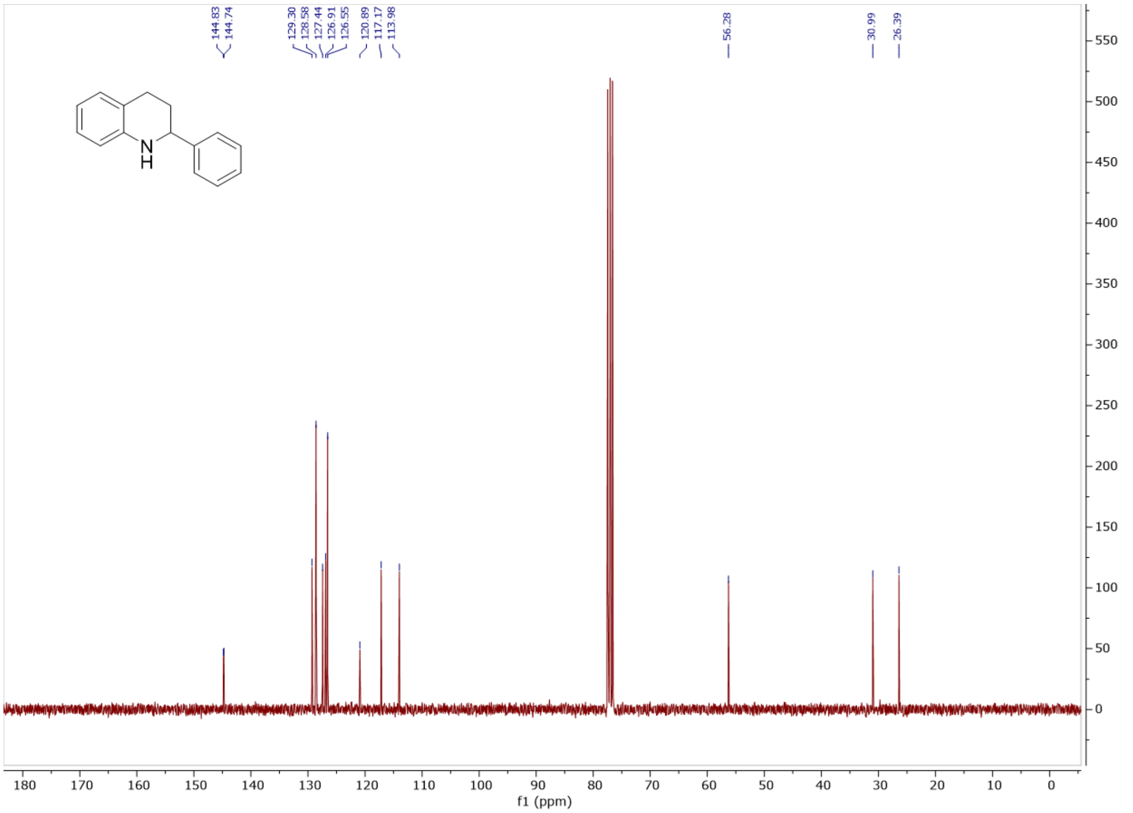

^{1}H NMR (300 MHz, $CDCl_3$) and ^{13}C NMR (76 MHz, $CDCl_3$) spectra for **2c**

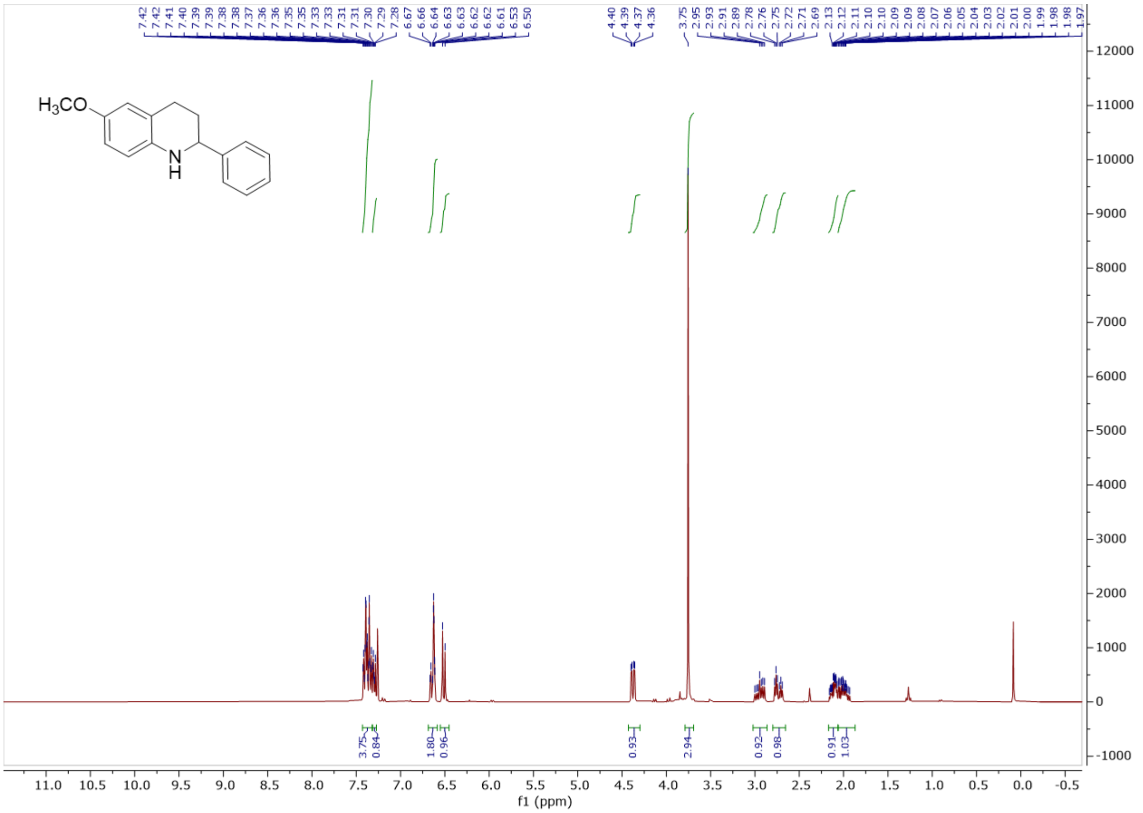

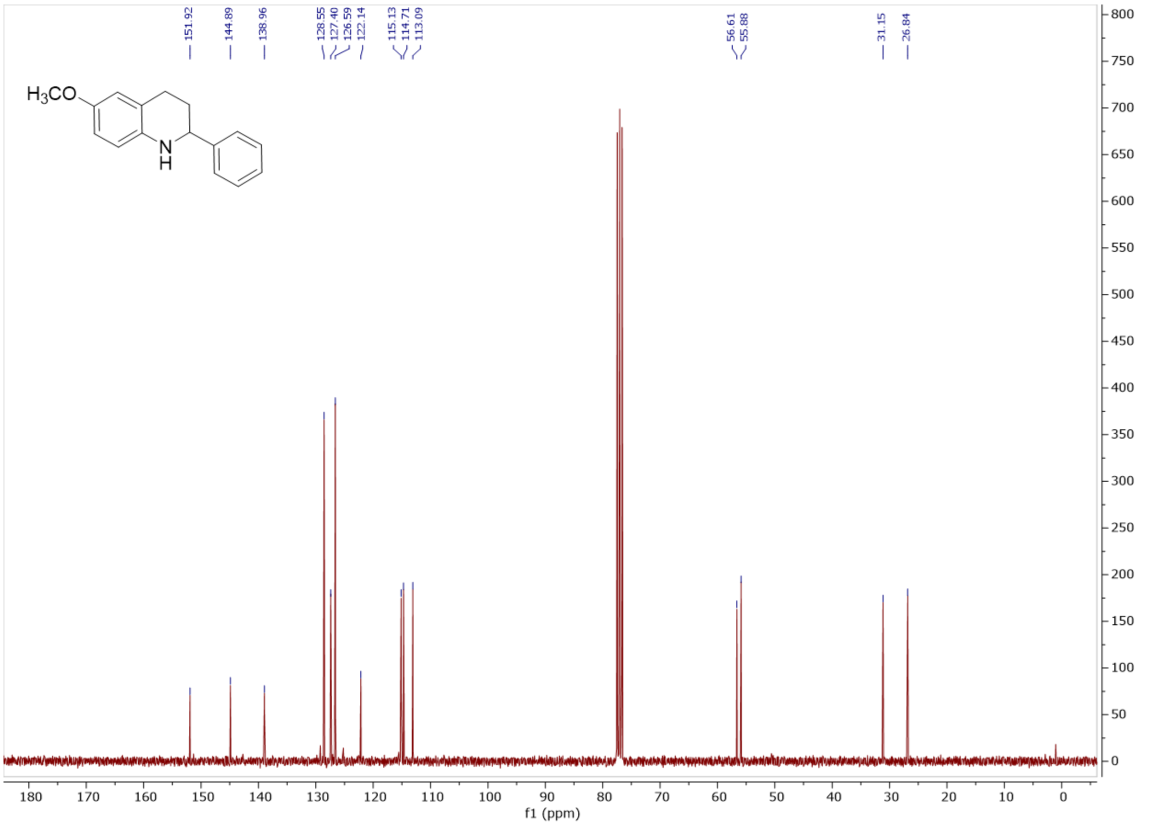

^{1}H NMR (300 MHz, $CDCl_3$) and ^{13}C NMR (76 MHz, $CDCl_3$) spectra for **2d**

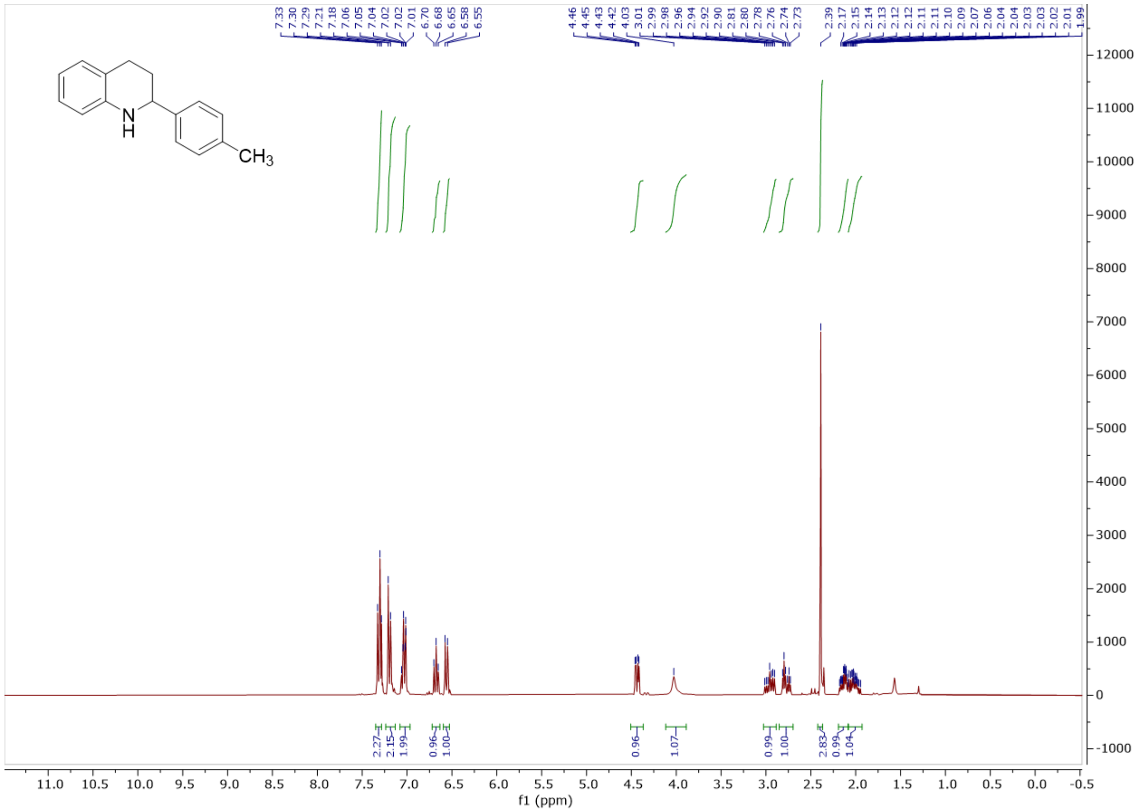

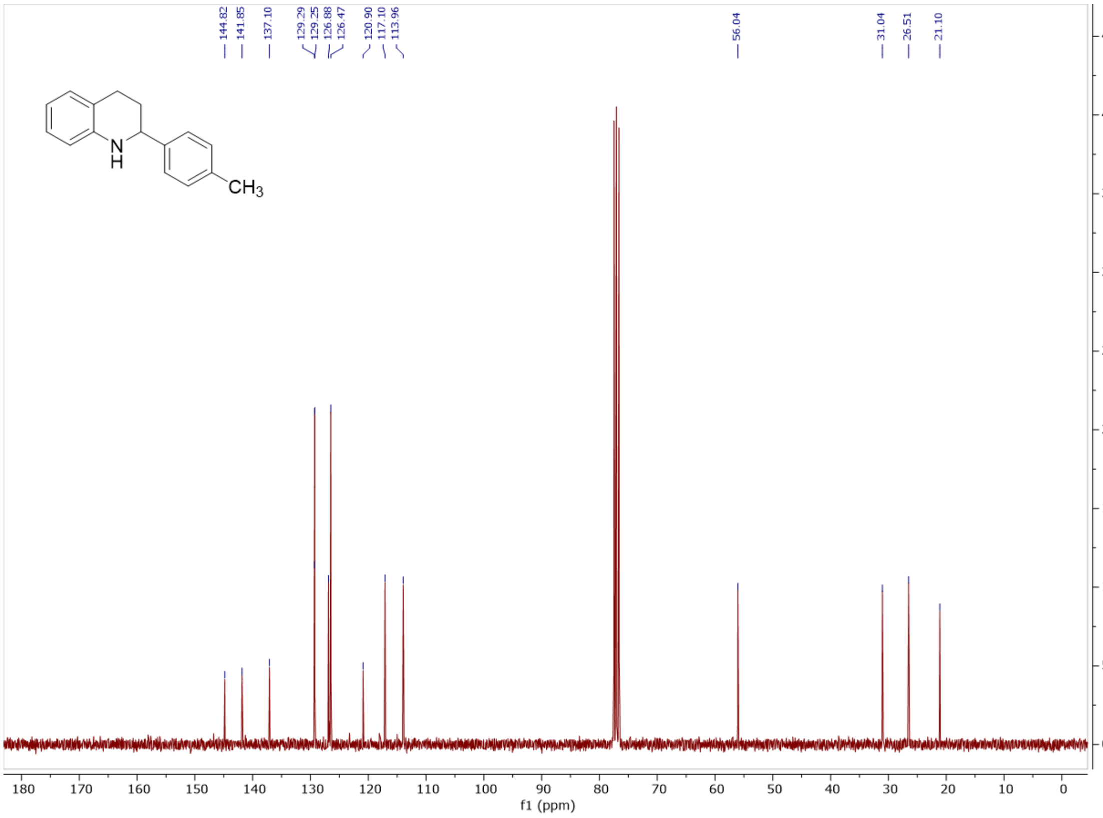

^{1}H NMR (300 MHz, $CDCl_3$) spectrum for **2e**

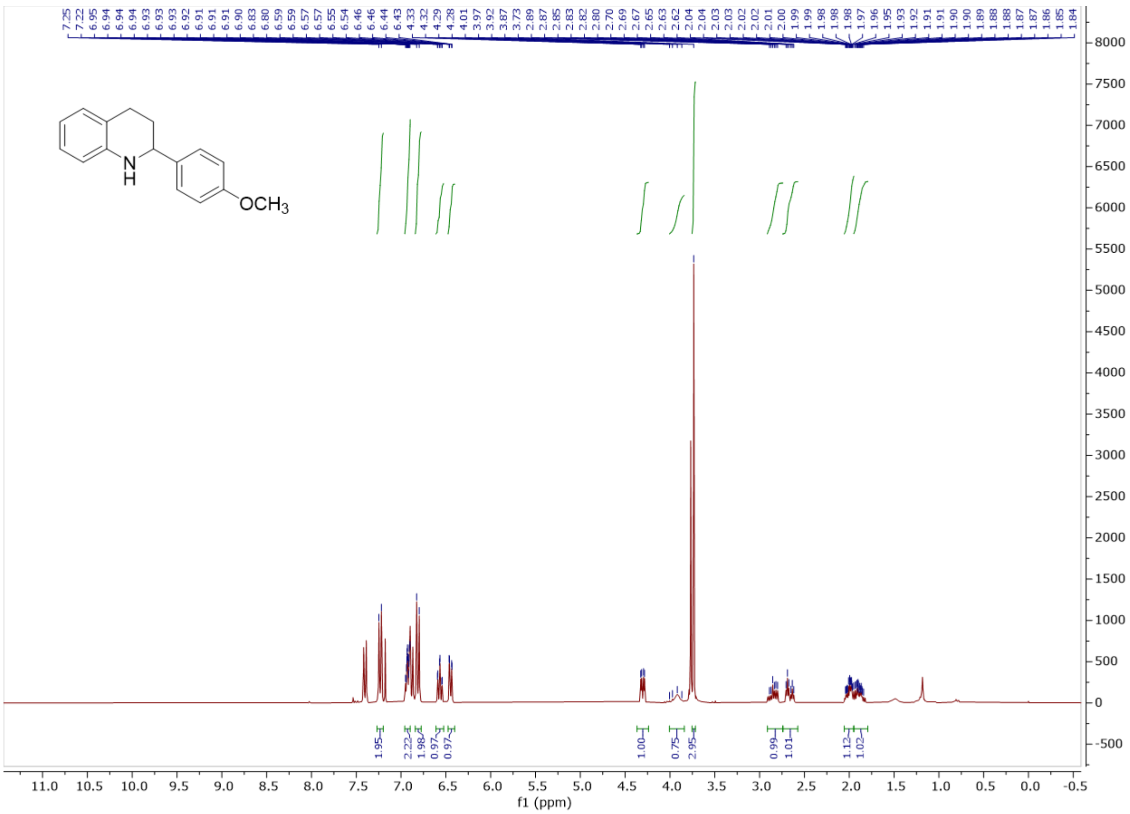

^{1}H NMR (300 MHz, $CDCl_3$) spectrum for **2f**

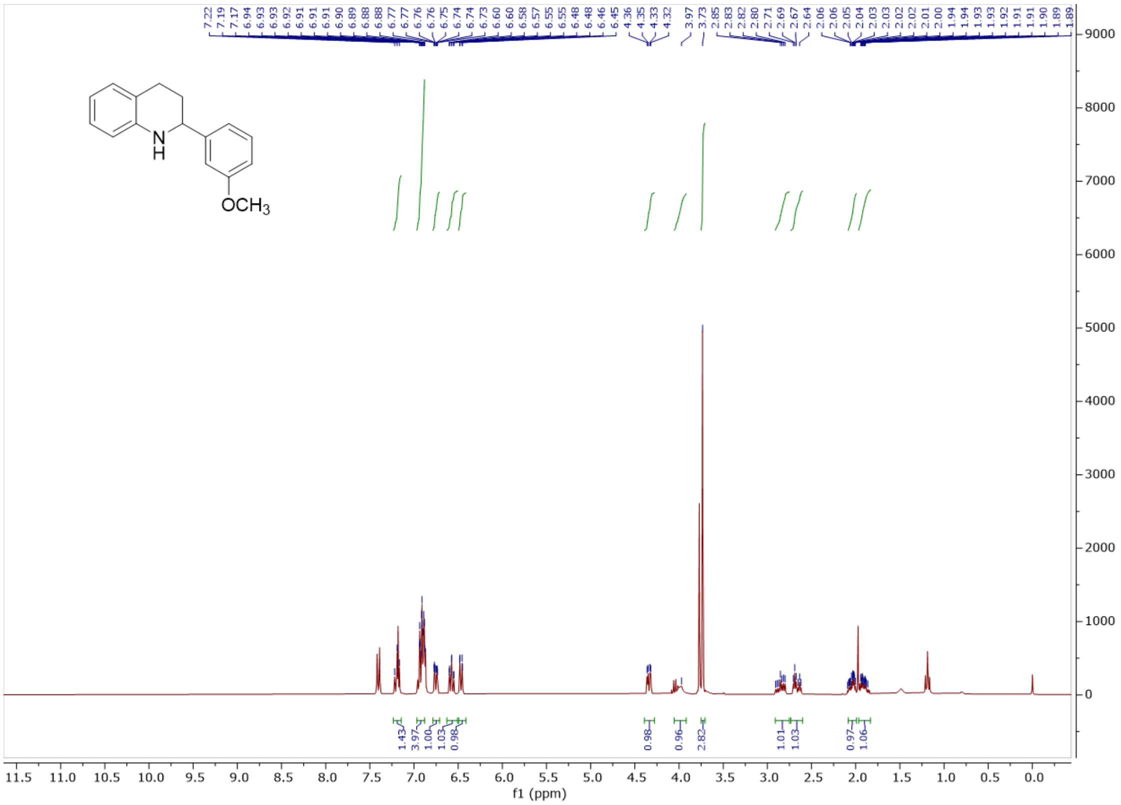

^{1}H NMR (300 MHz, $CDCl_3$) and ^{19}F NMR (282 MHz, $CDCl_3$) spectra for **2g**

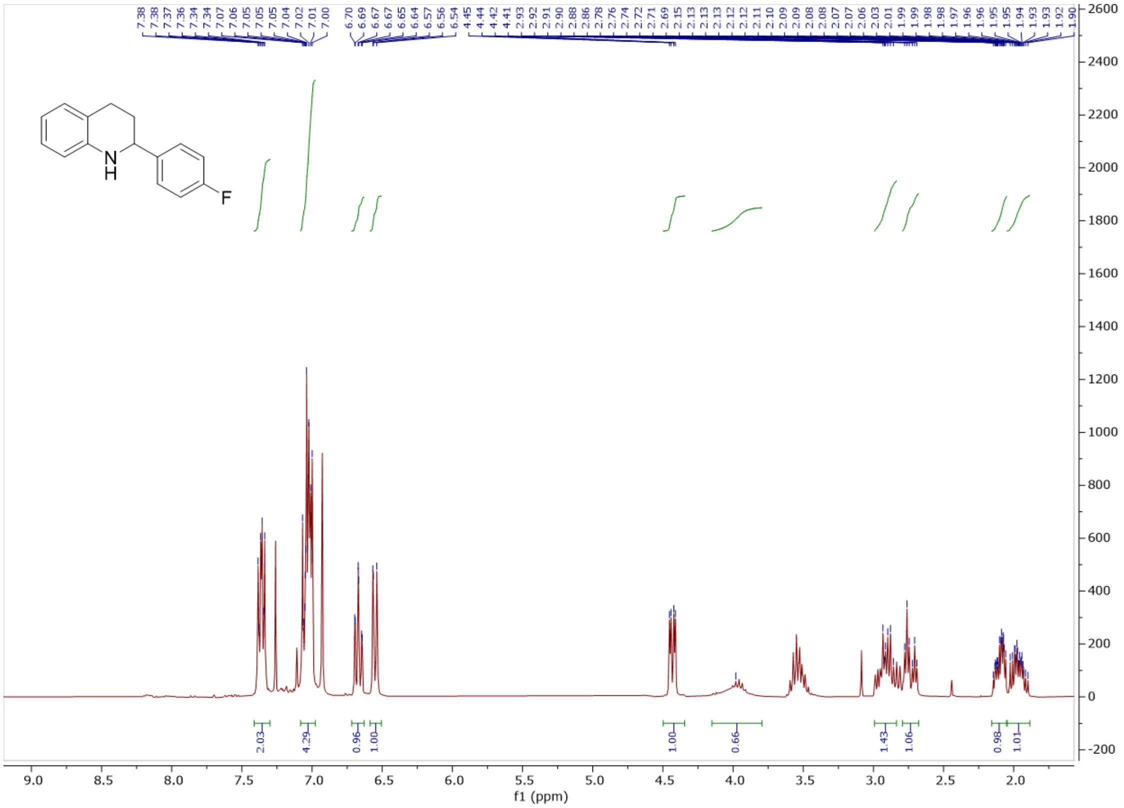

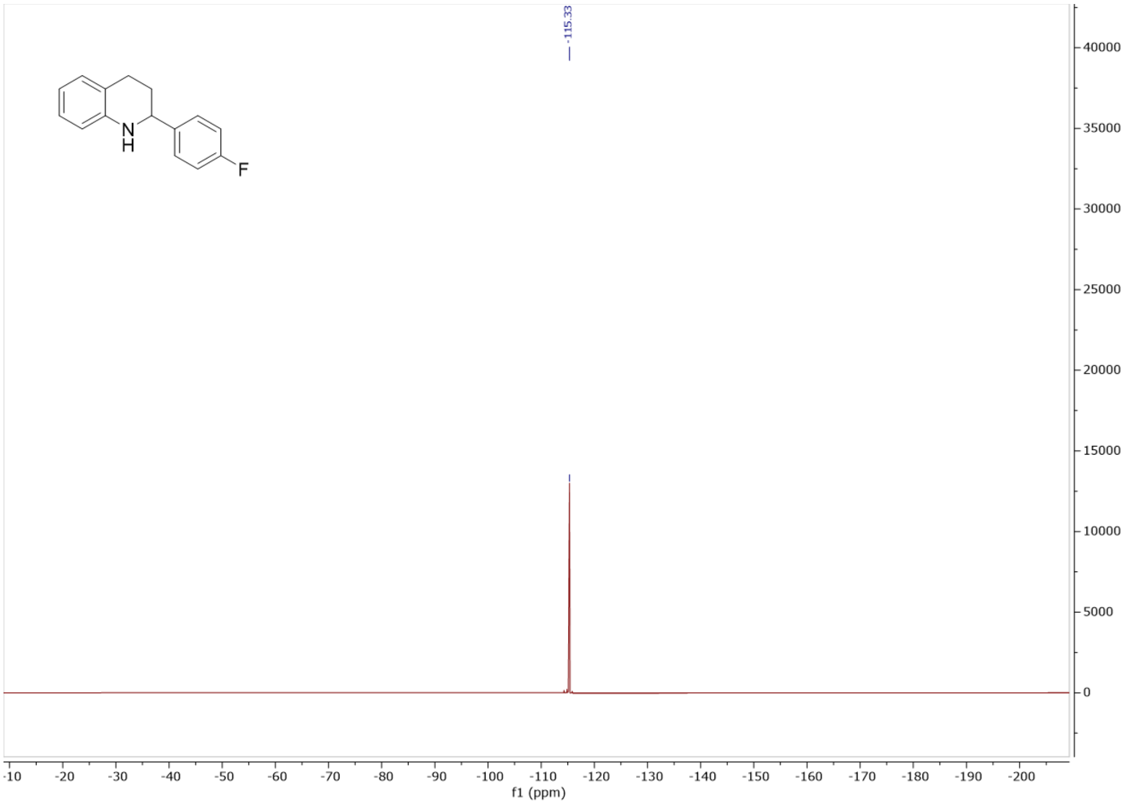

^{1}H NMR (300 MHz, $CDCl_3$) spectrum for **2h**

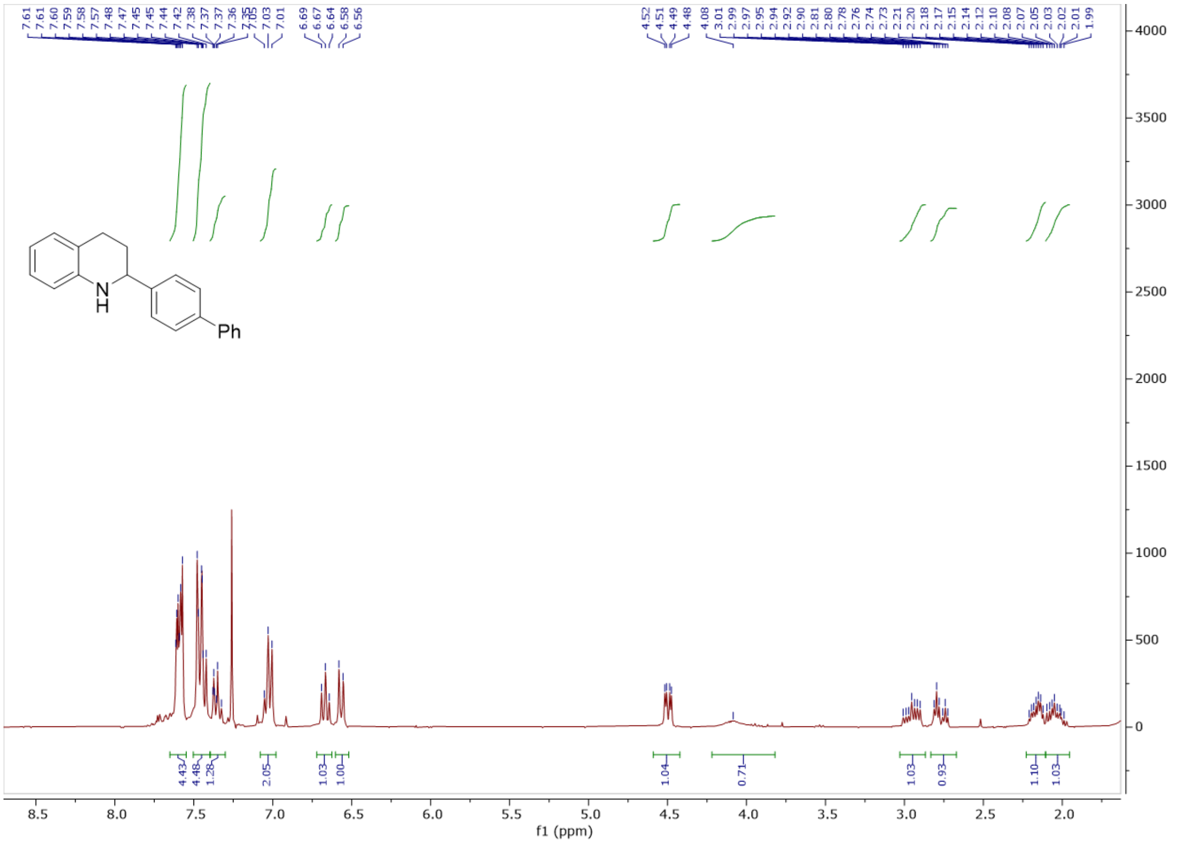

^{1}H NMR (300 MHz, $CDCl_3$) and ^{19}F NMR (282 MHz, $CDCl_3$) spectra for **2i**

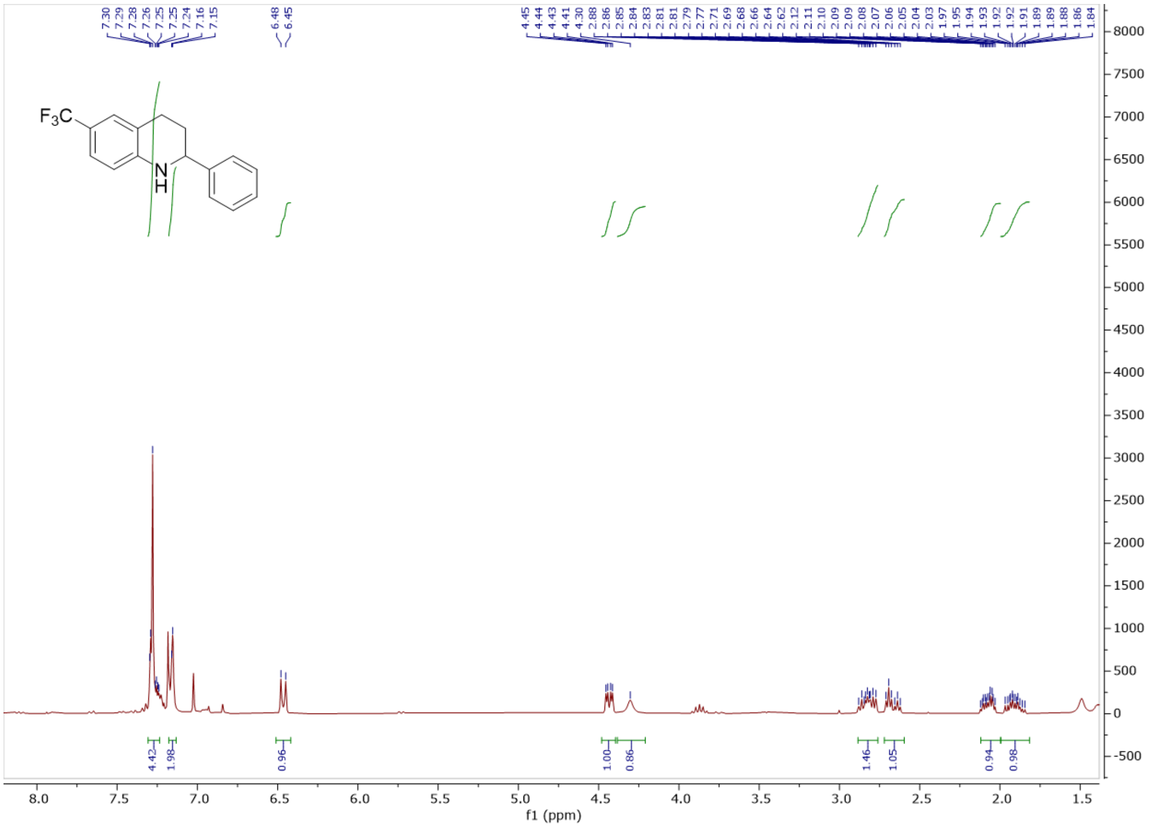

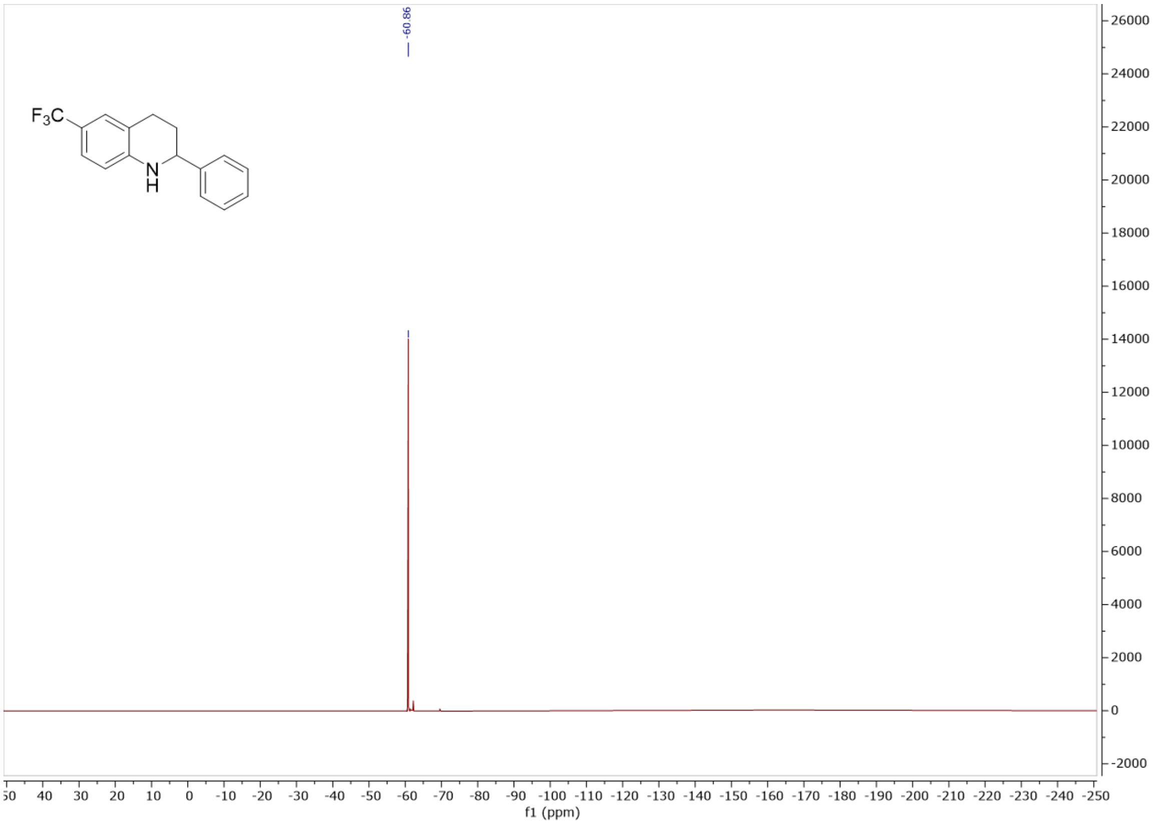

^{1}H NMR (300 MHz, $CDCl_3$) and ^{19}F NMR (282 MHz, $CDCl_3$) spectra for **2i'**

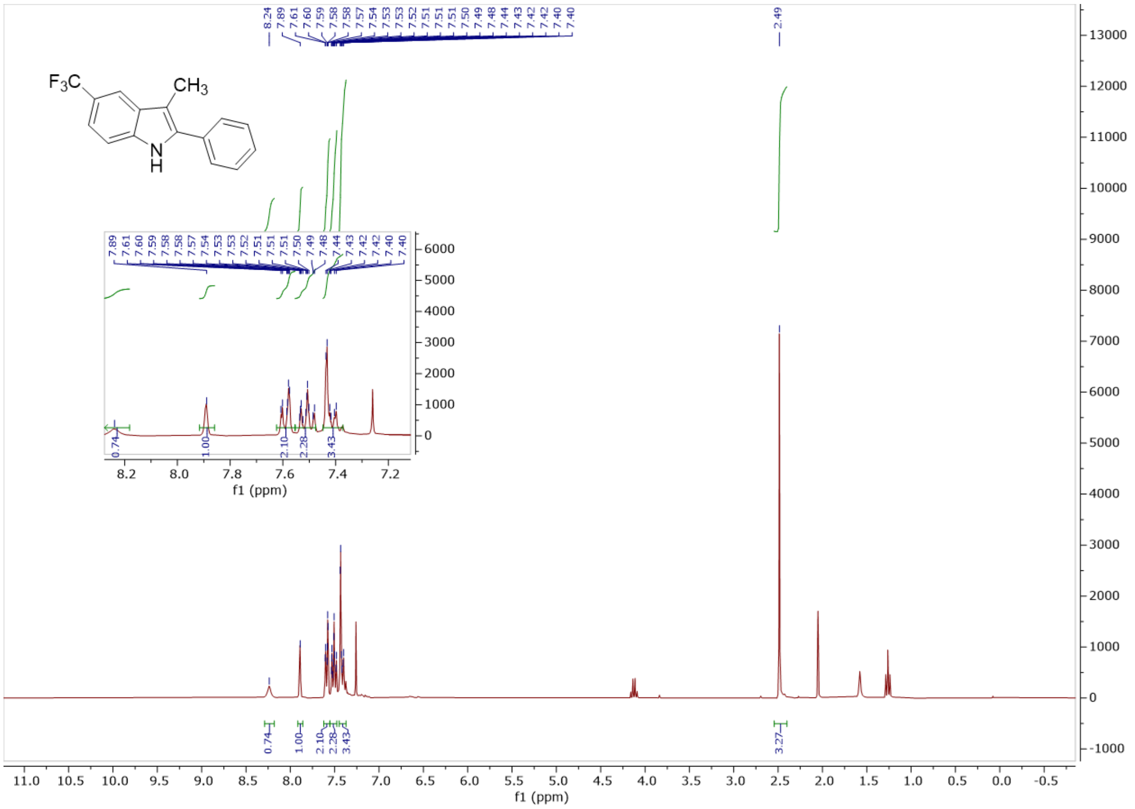

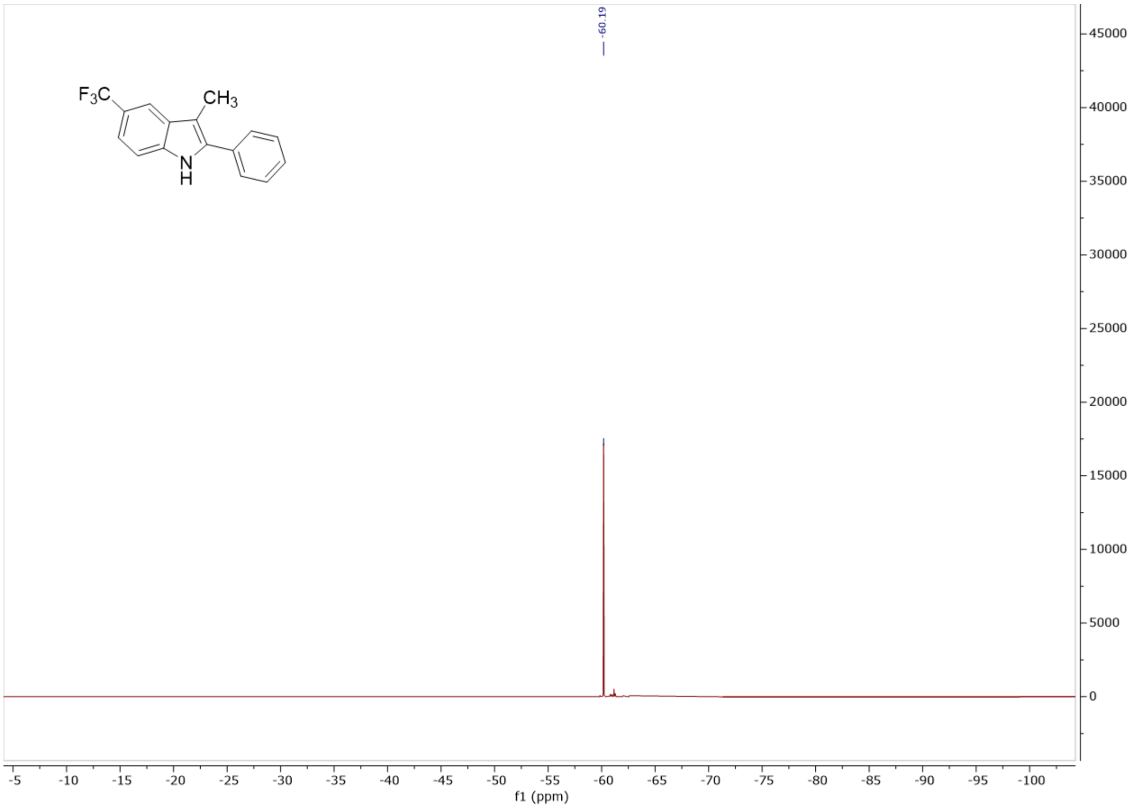

^{1}H NMR (500 MHz, $CDCl_3$) and ^{19}F NMR (126 MHz, $CDCl_3$) spectra for **2j'**

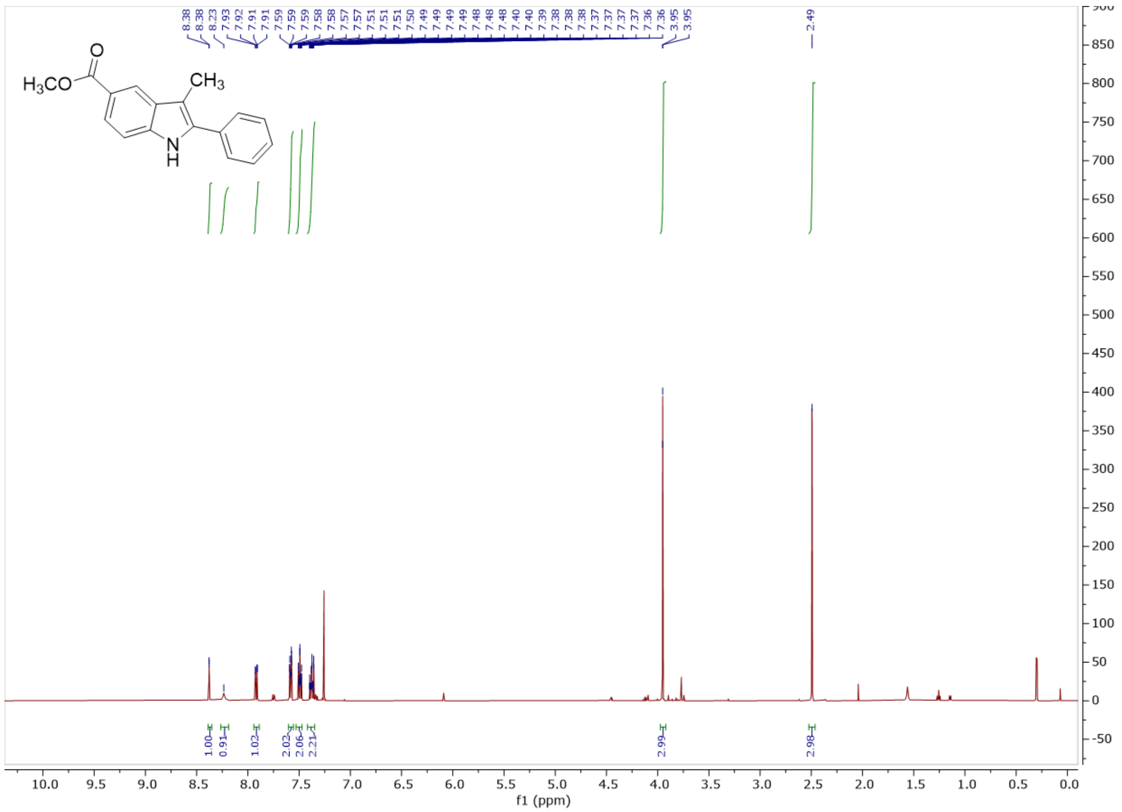

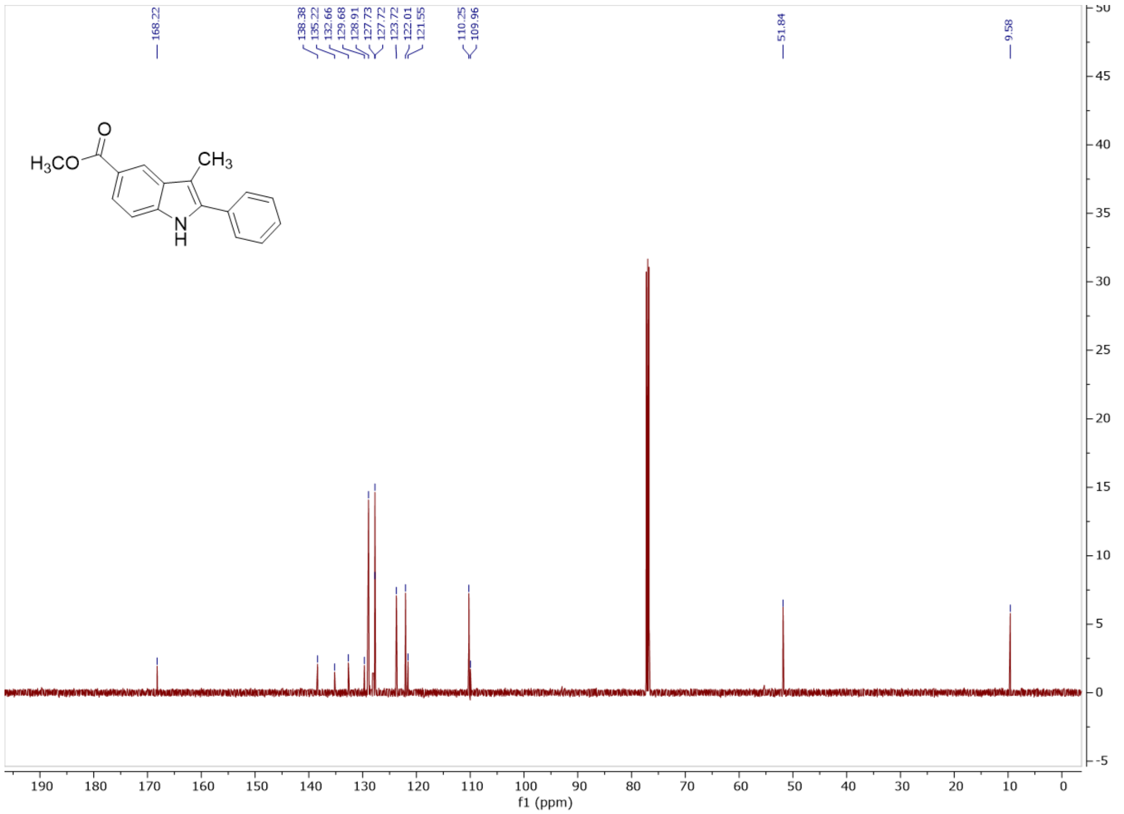

^{1}H NMR (599 MHz, $CDCl_3$) and ^{13}C NMR (101 MHz, $CDCl_3$) spectra for **2k**

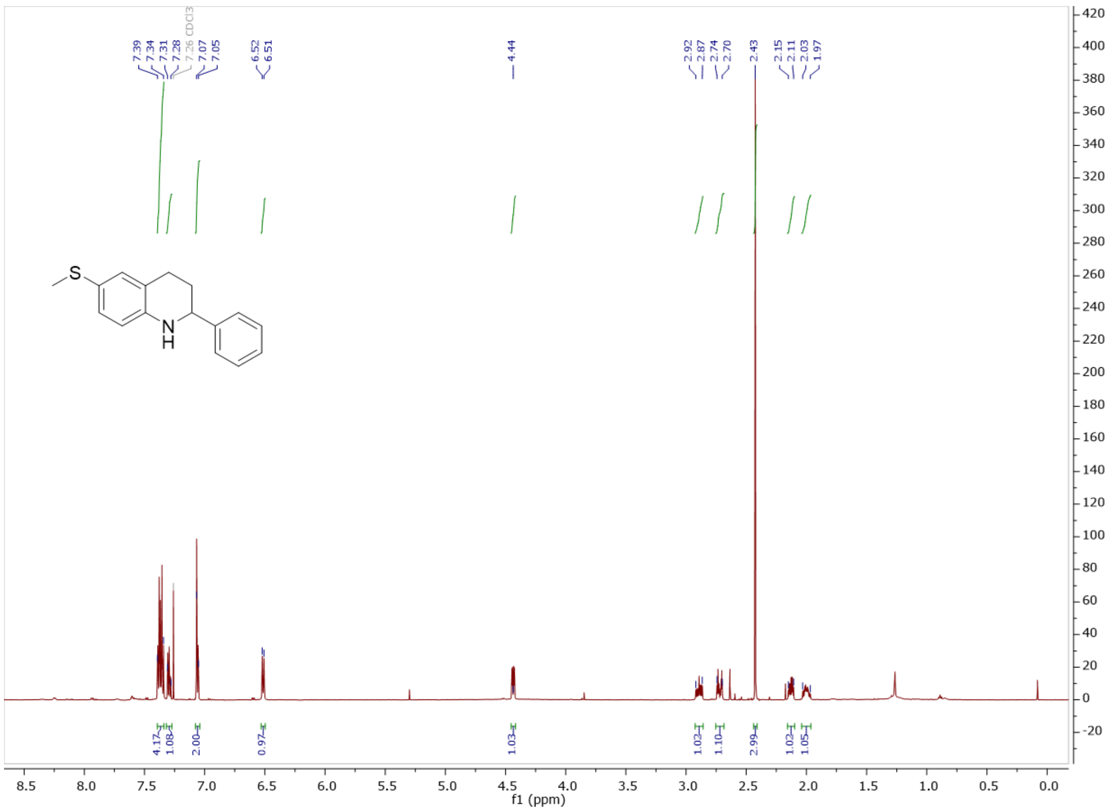

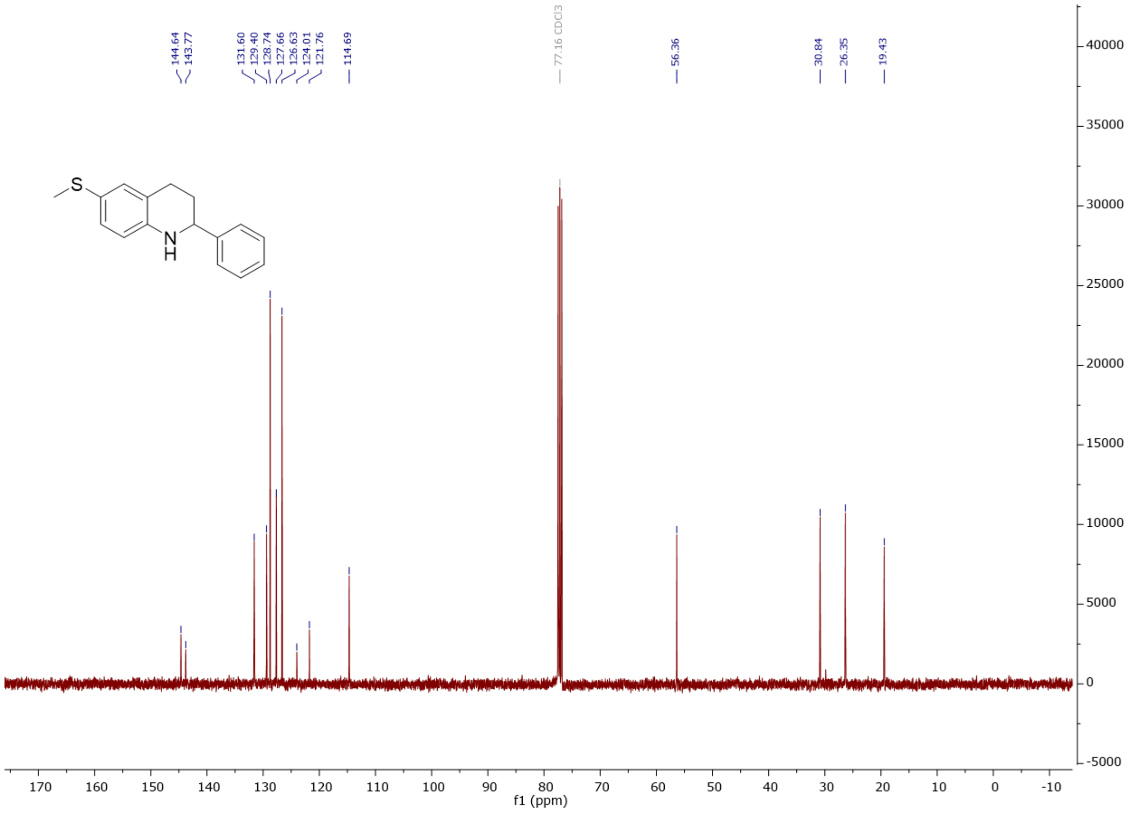

^{1}H NMR (300 MHz, $CDCl_3$) and ^{13}C NMR (76 MHz, $CDCl_3$) spectra for **2l**

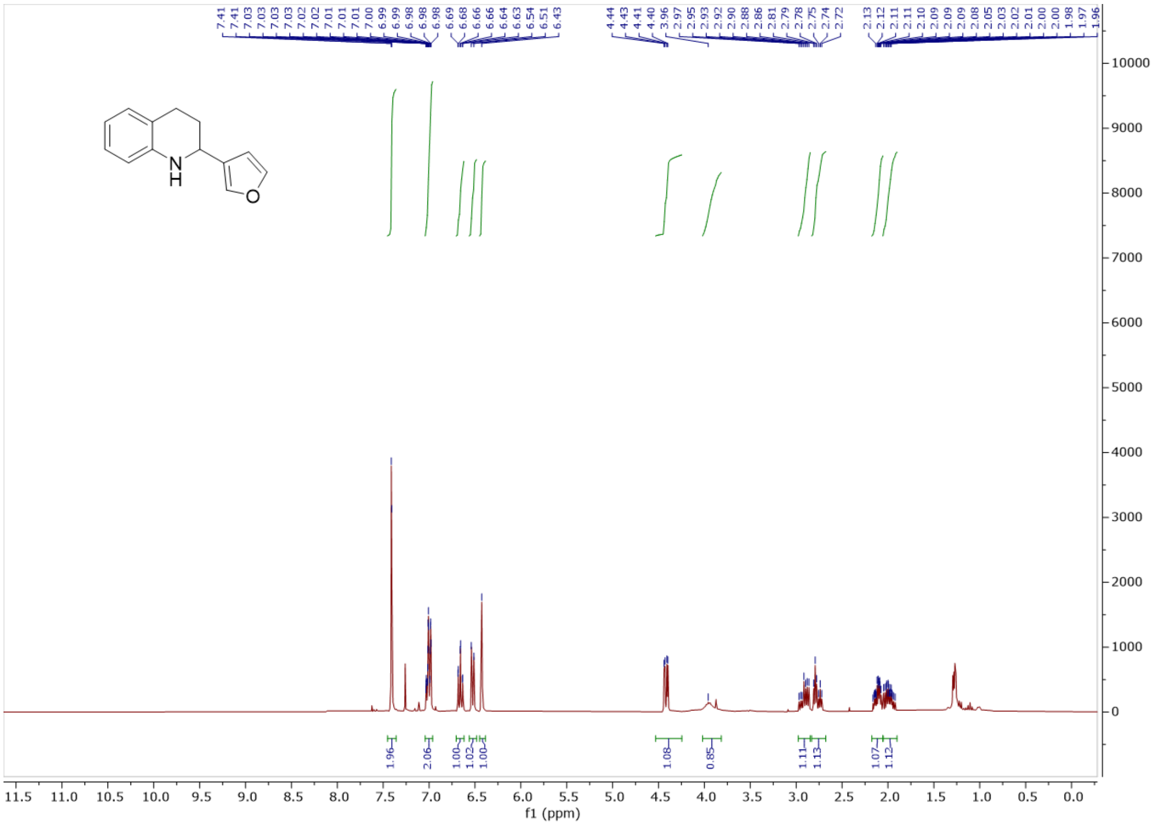

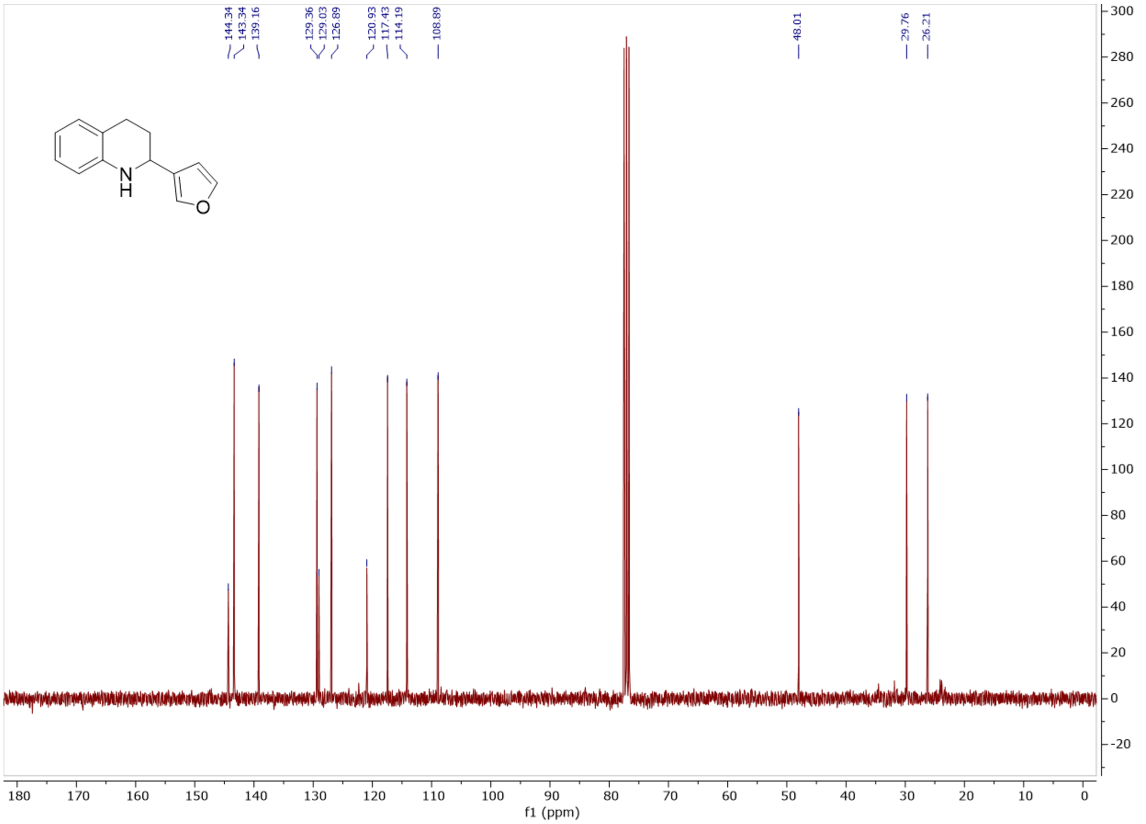

^{1}H NMR (400 MHz, $CDCl_3$) and ^{13}C NMR (101 MHz, $CDCl_3$) spectra for **2m**

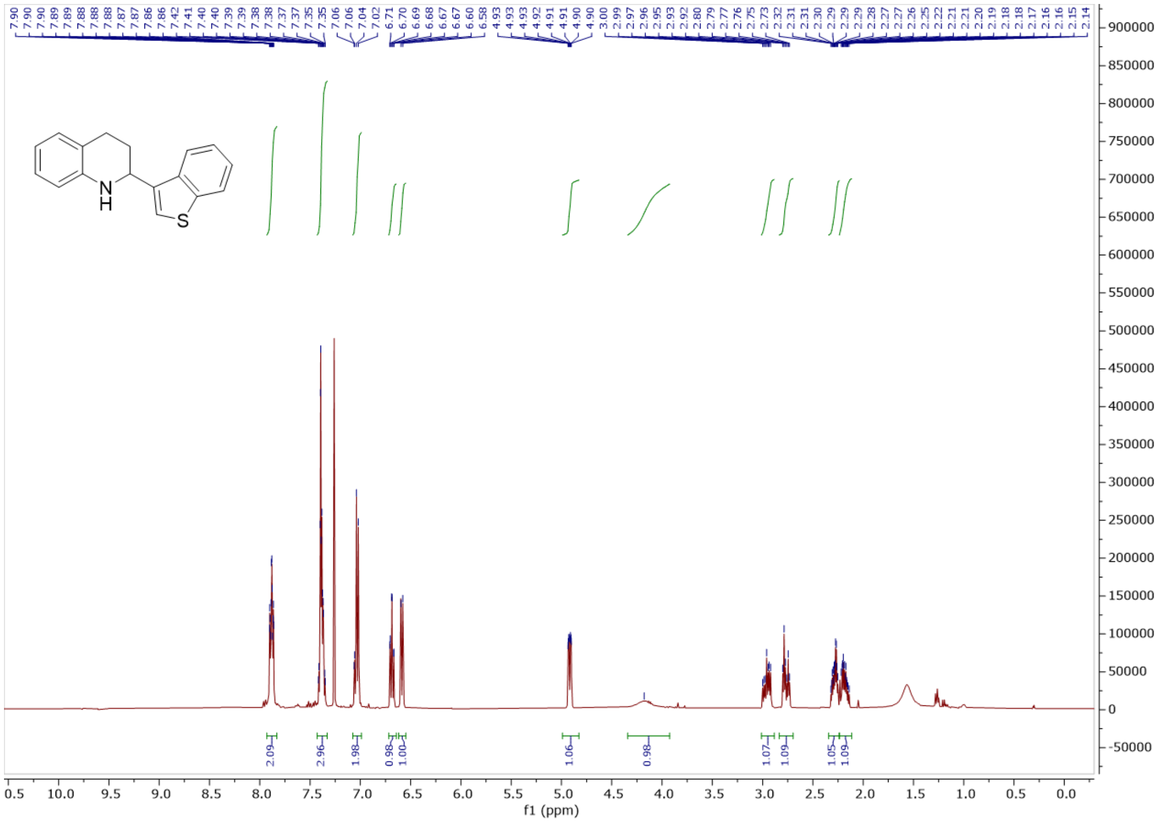

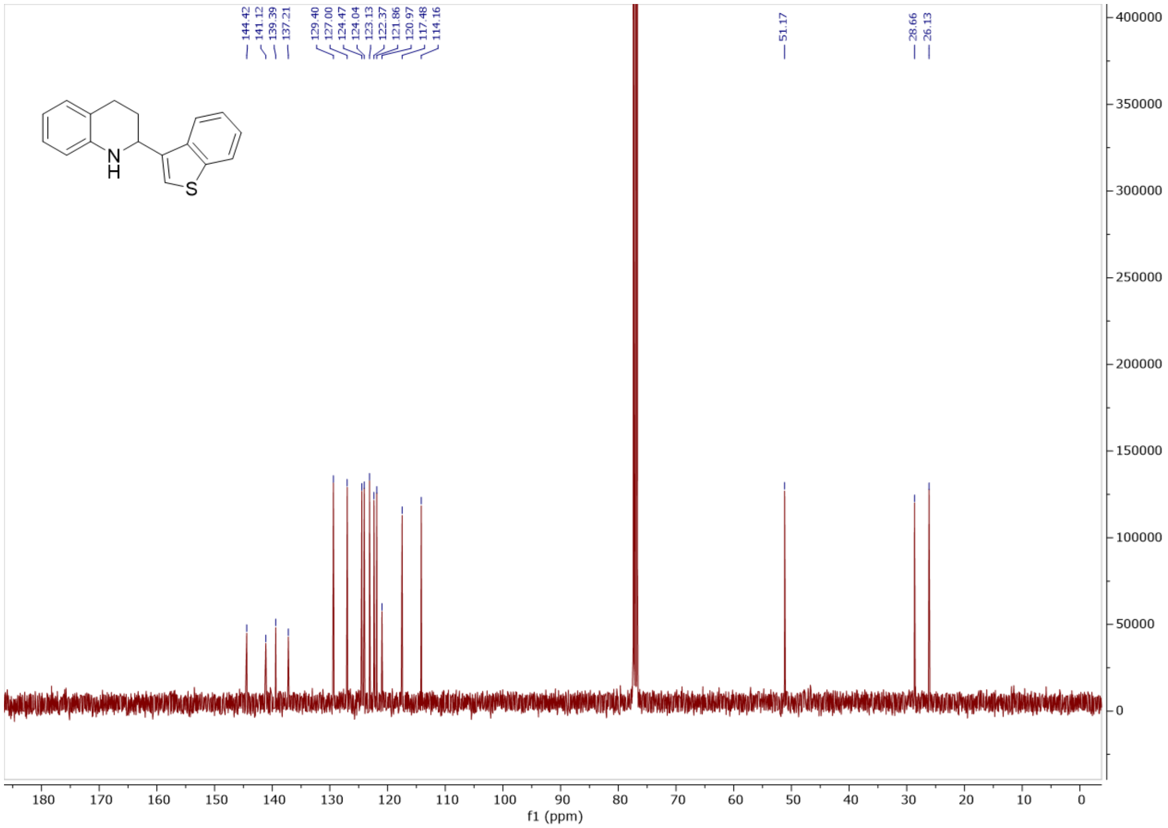

^{1}H NMR (300 MHz, $CDCl_3$) and ^{13}C NMR (76 MHz, $CDCl_3$) spectra for **2n**

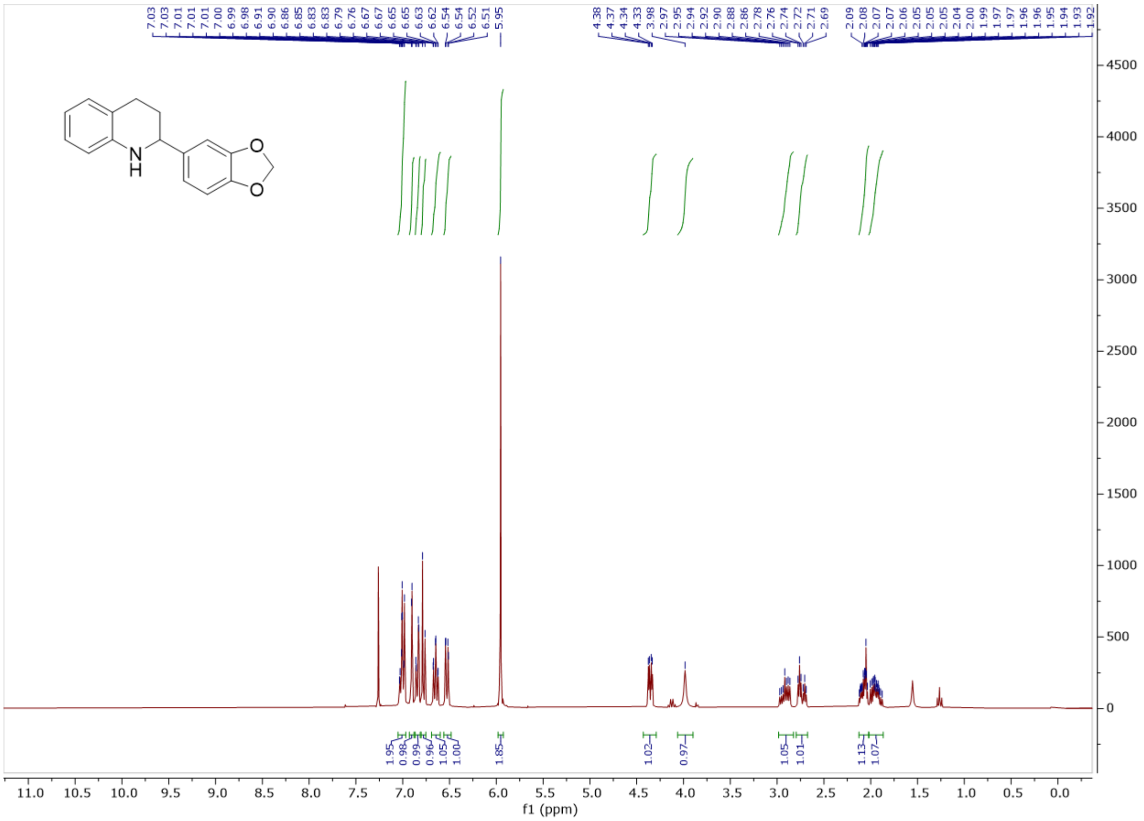

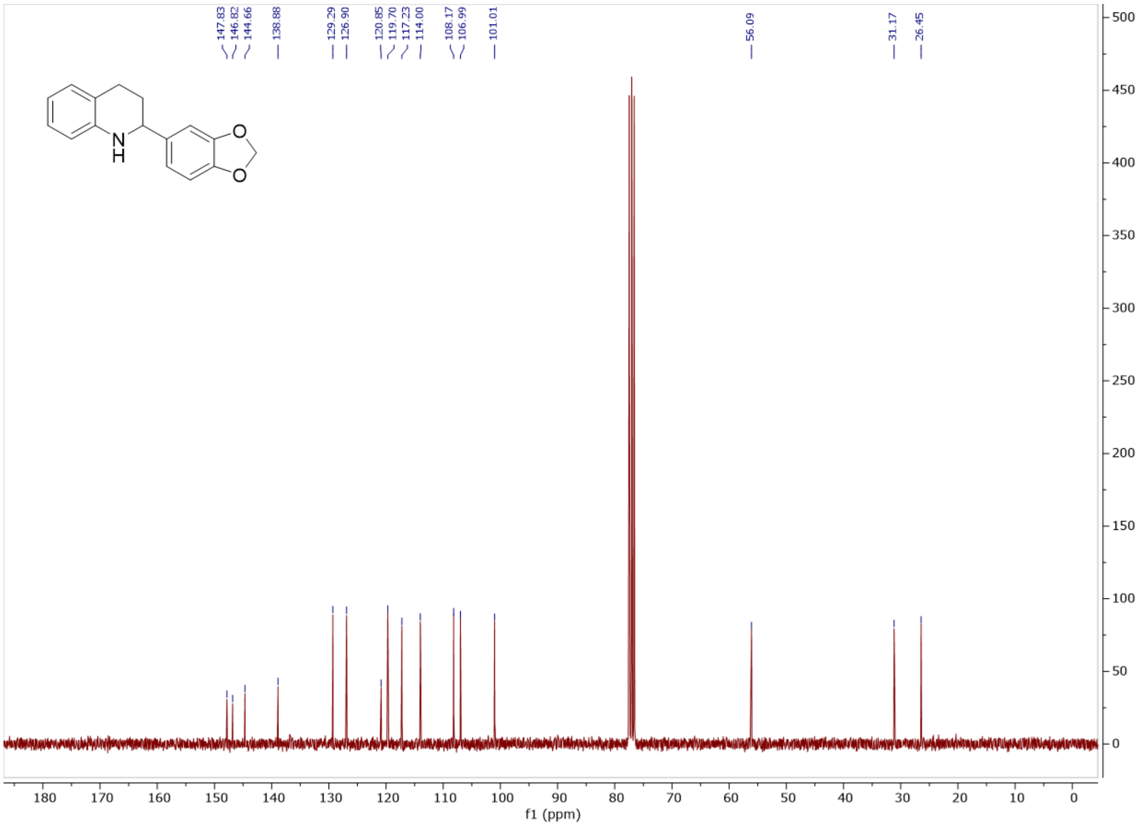

^{1}H NMR (300 MHz, $CDCl_3$) and ^{13}C NMR (151 MHz, $CDCl_3$) spectrum for **2m'**

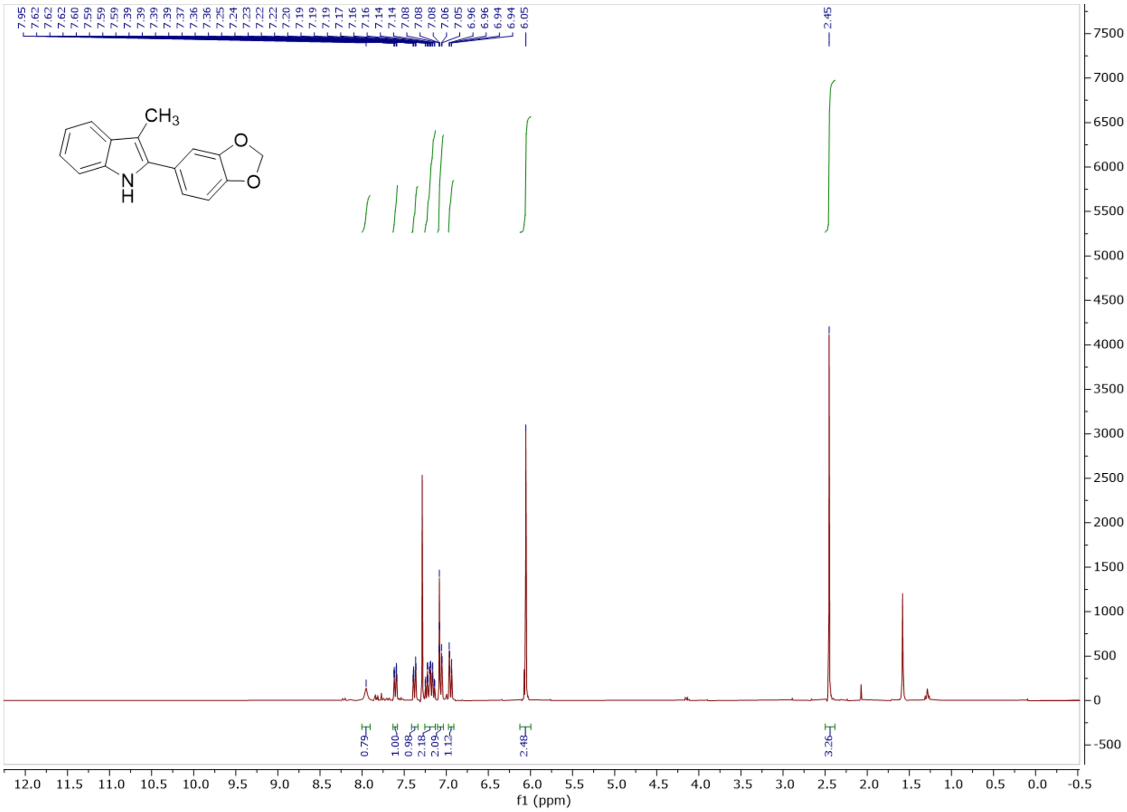

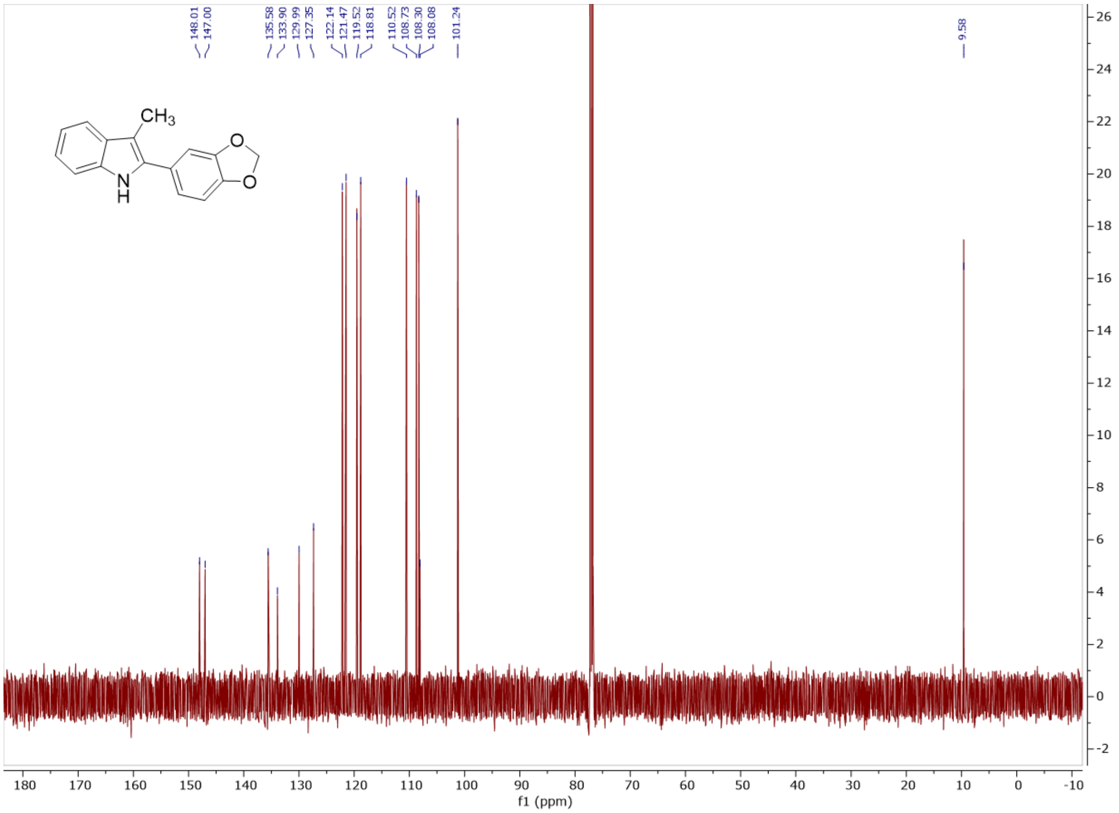

^{1}H NMR (300 MHz, $CDCl_3$) and ^{13}C NMR (76 MHz, $CDCl_3$) spectra for **2o**

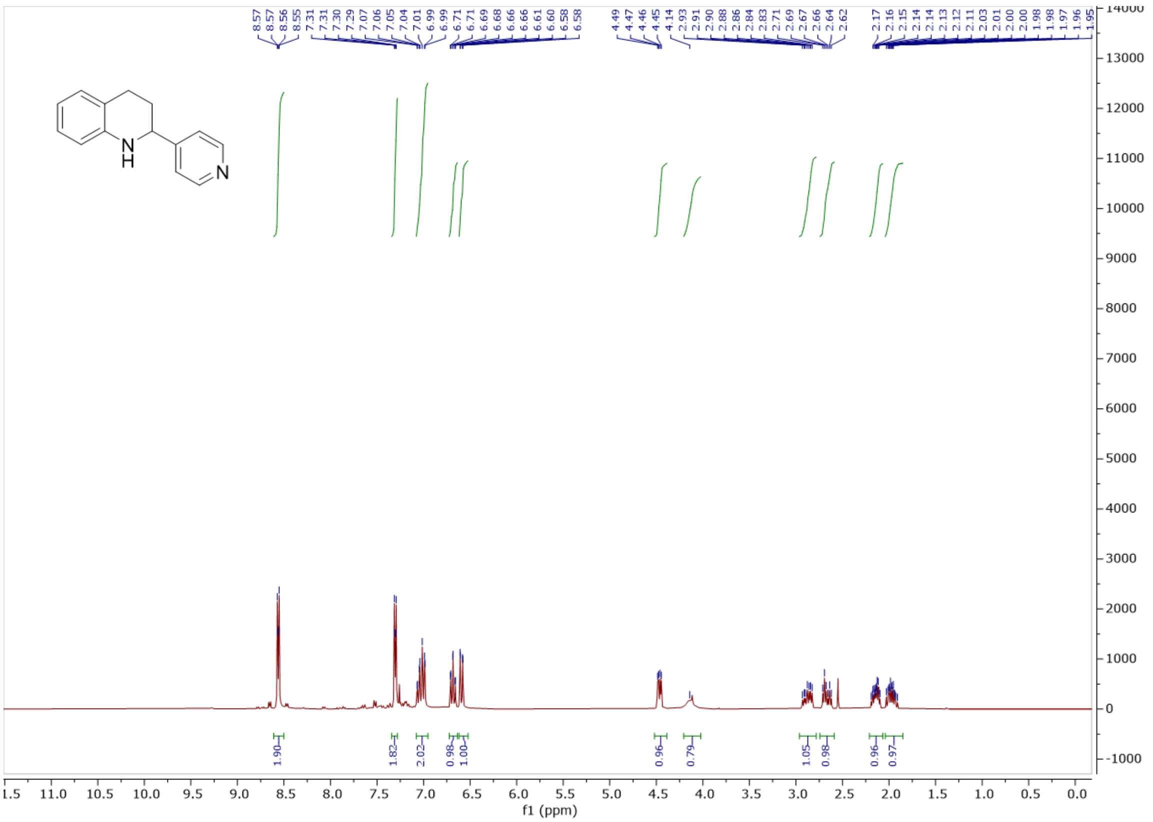

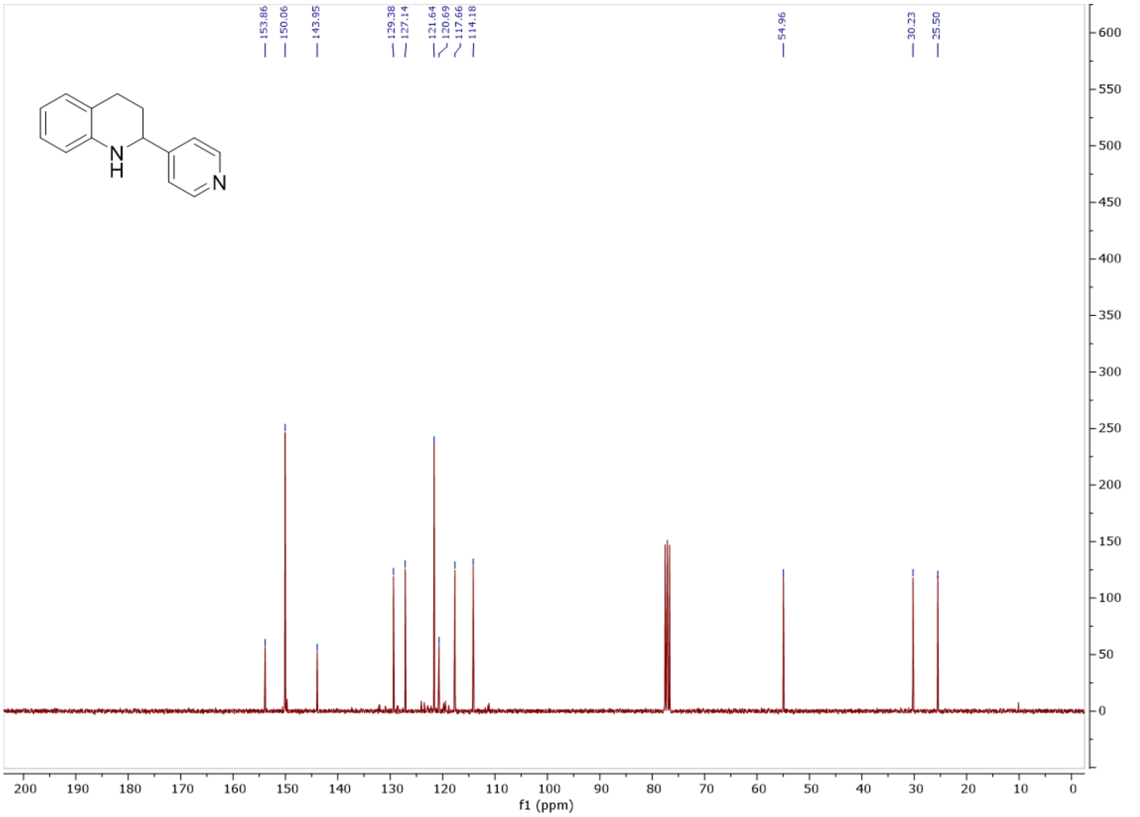

^{1}H NMR (300 MHz, $CDCl_3$) and ^{13}C NMR (76 MHz, $CDCl_3$) spectra for **2p**

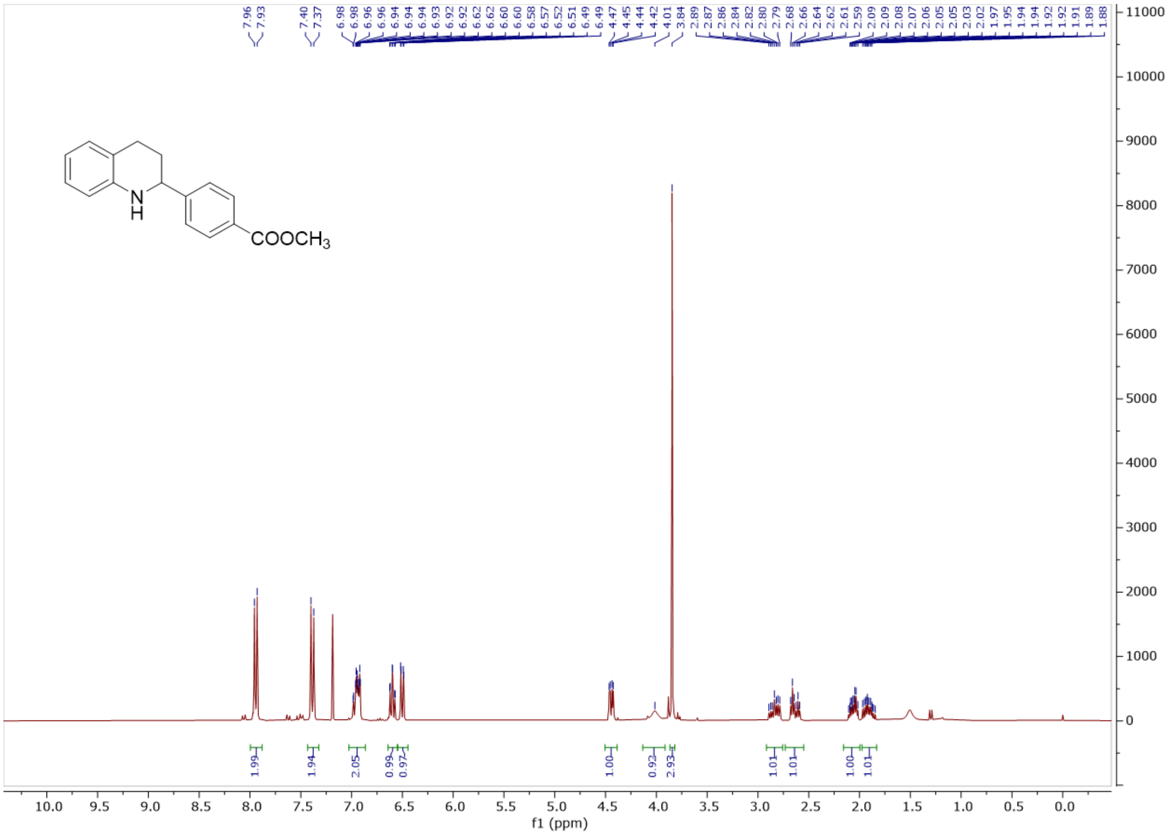

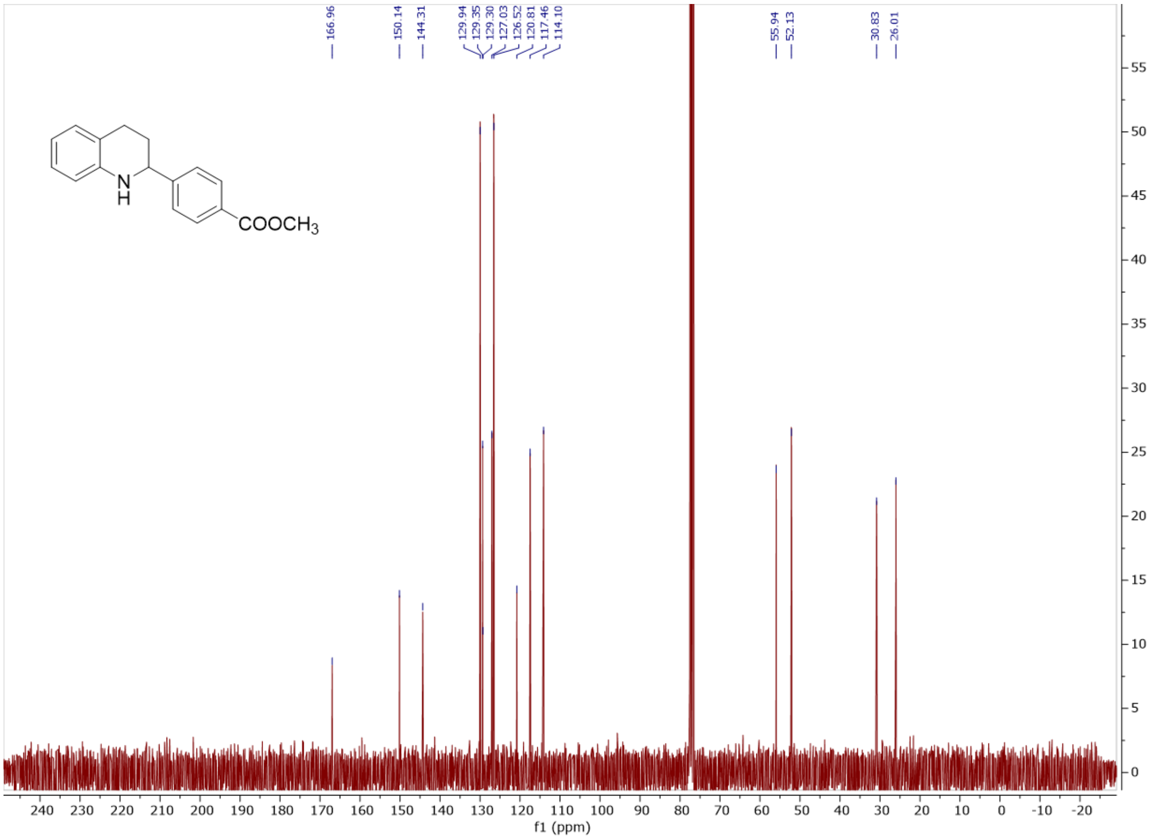

^{1}H NMR (400 MHz, $CDCl_3$) and ^{13}C NMR (101 MHz, $CDCl_3$) spectra for **2q**

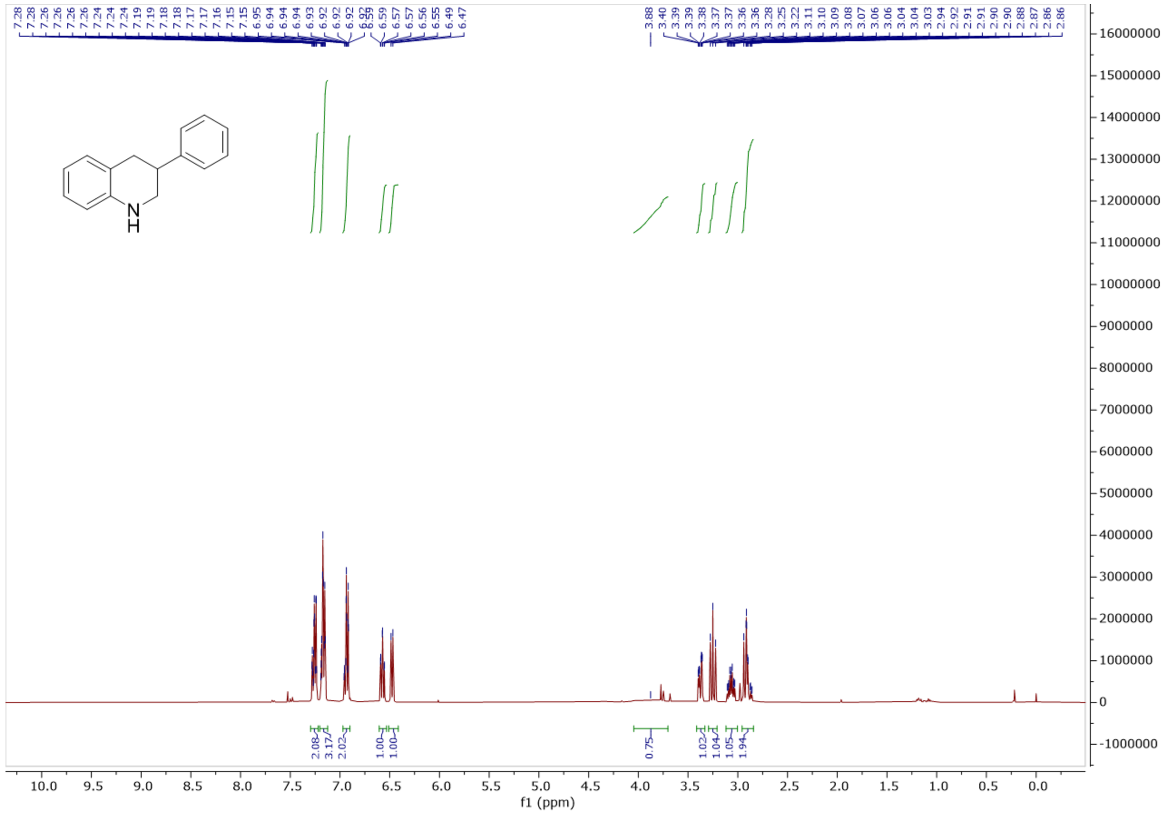

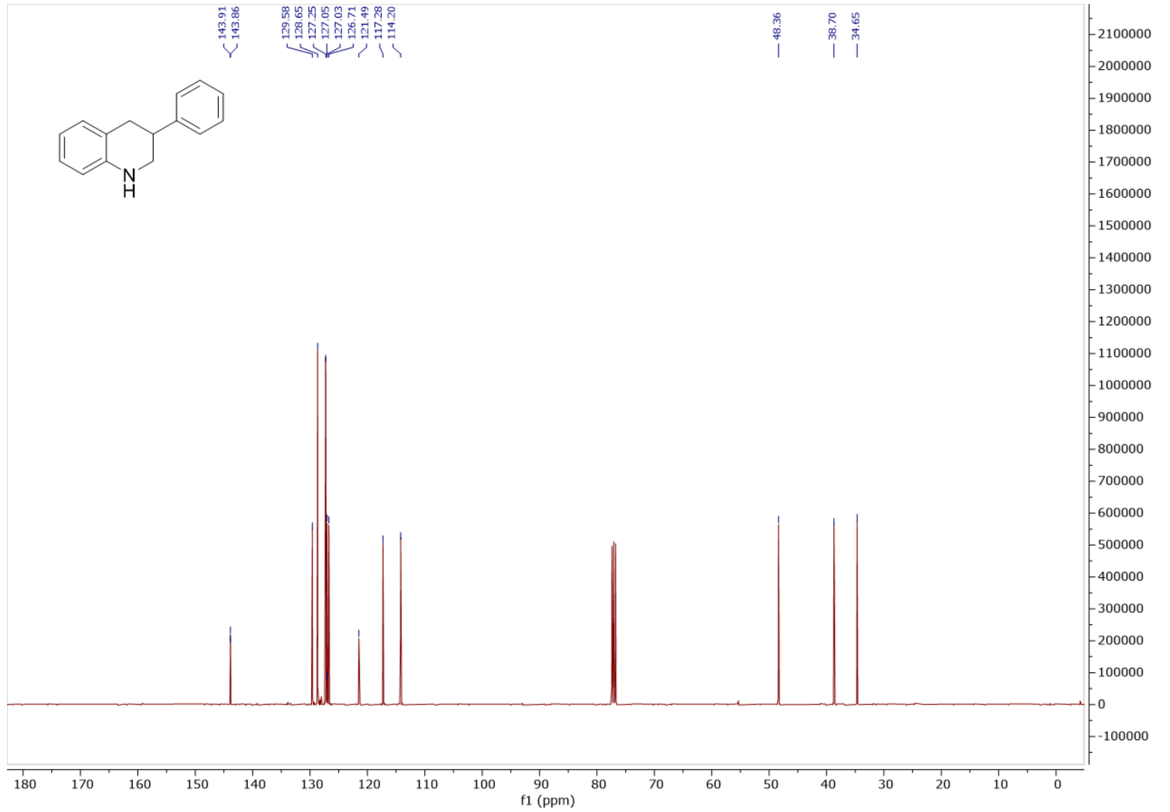

^{1}H NMR (300 MHz, $CDCl_3$) and ^{13}C NMR (76 MHz, $CDCl_3$) spectra for **2r**

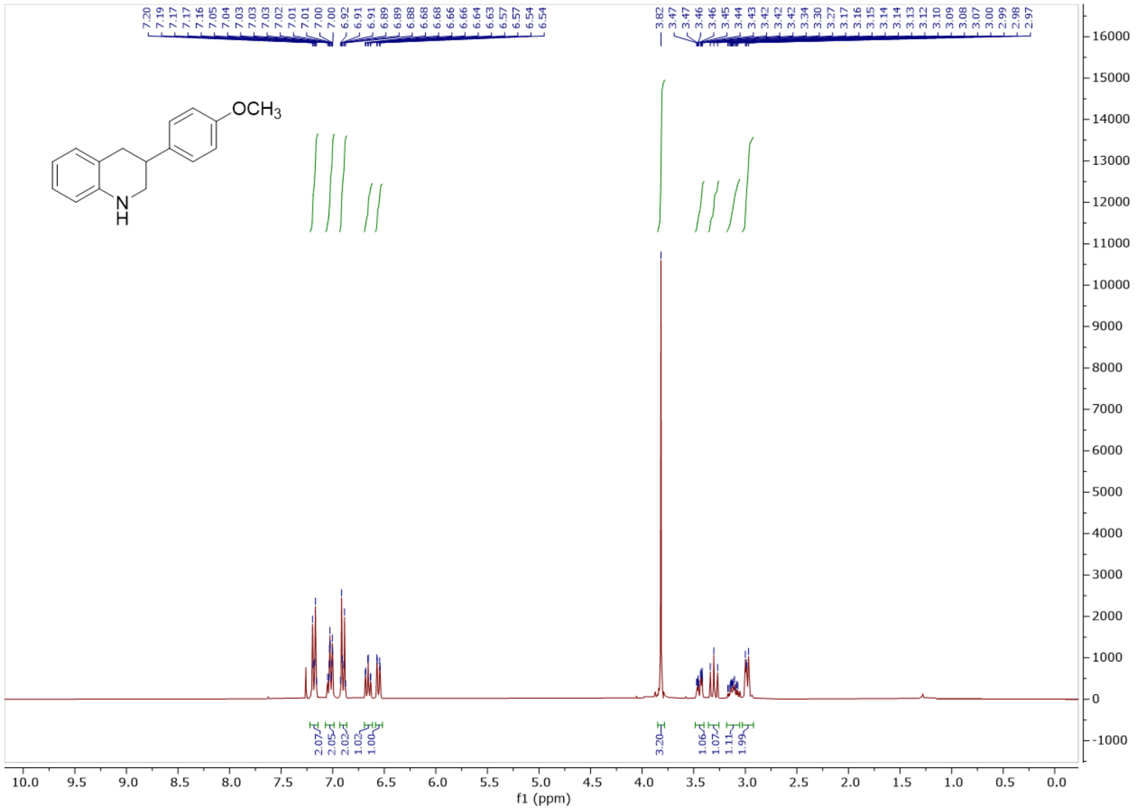

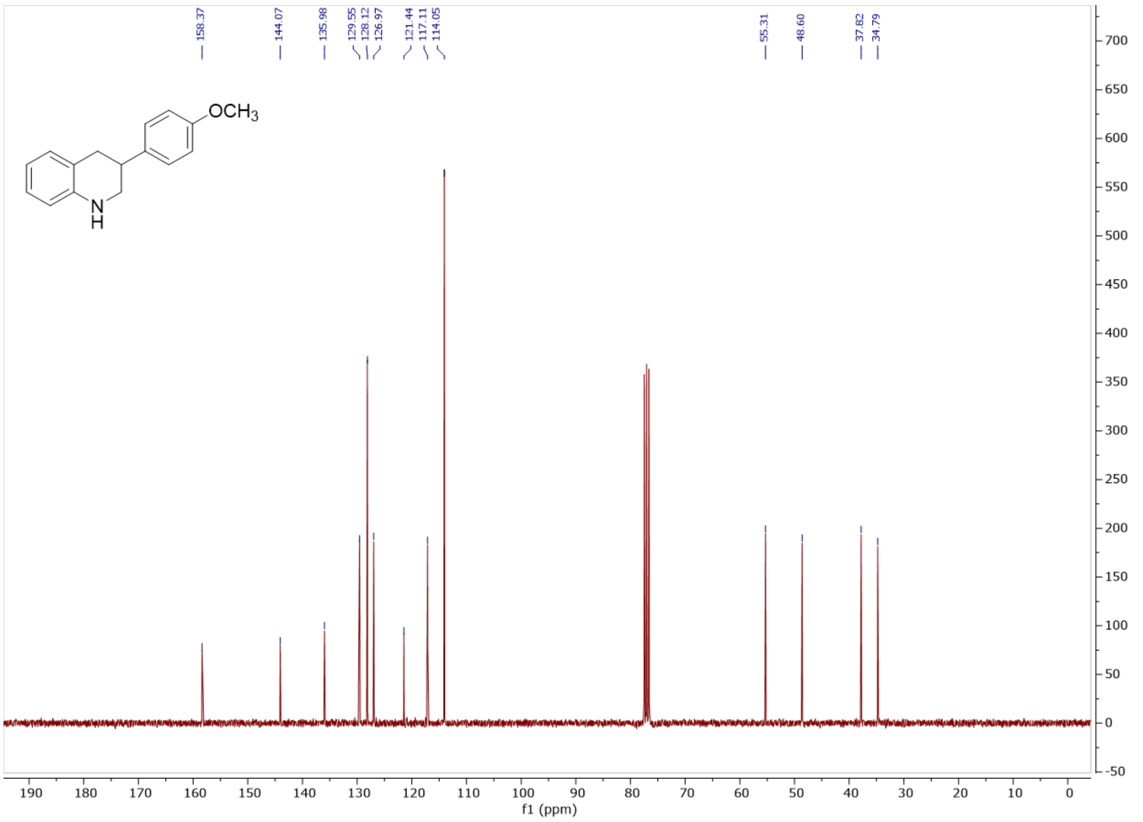

^{1}H NMR (300 MHz, $CDCl_3$) and ^{13}C NMR (76 MHz, $CDCl_3$) spectra for **2s**

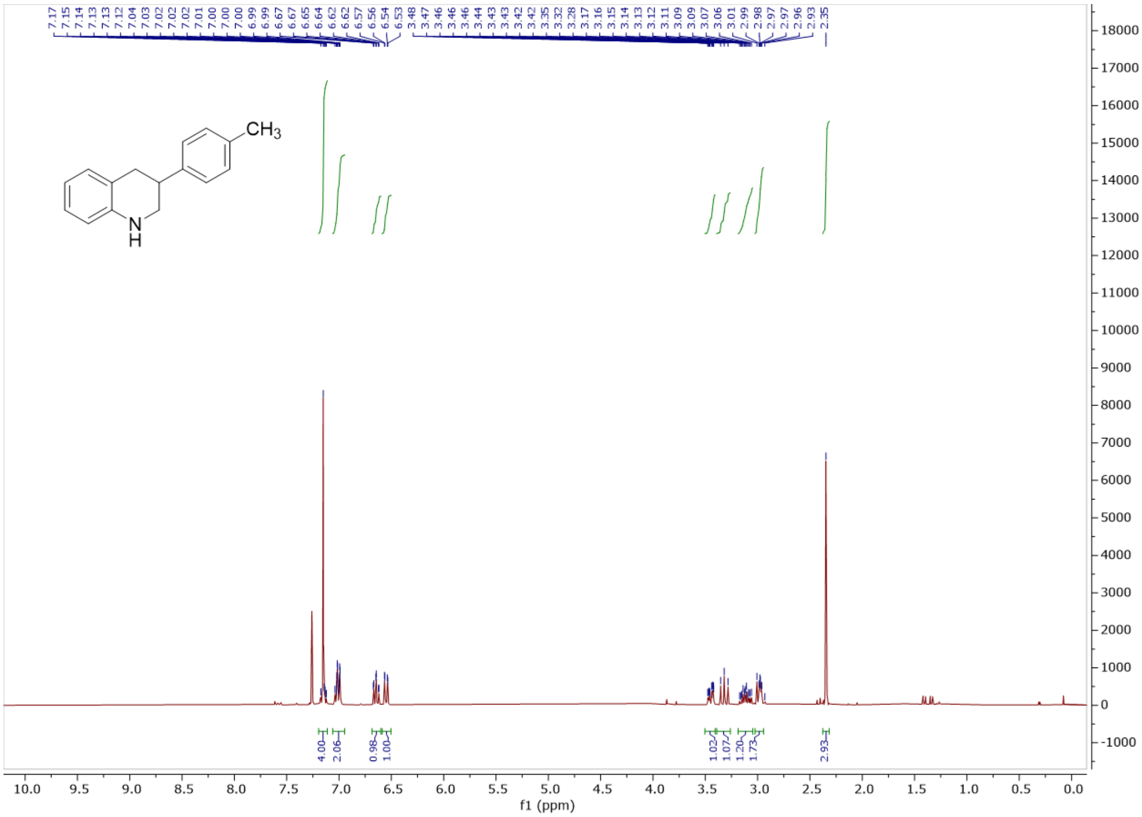

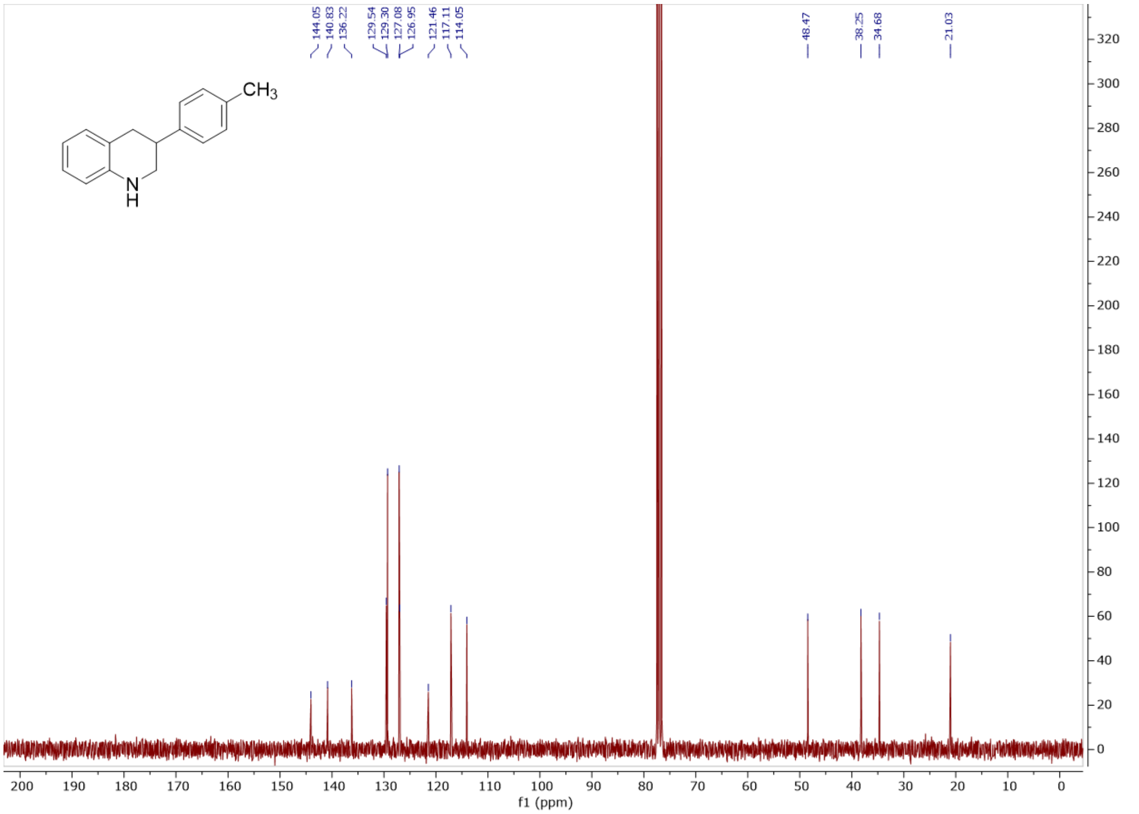

^{1}H NMR (300 MHz, $CDCl_3$), ^{19}F NMR (282 MHz, $CDCl_3$) and ^{13}C NMR (76 MHz, $CDCl_3$) spectra for **2t**

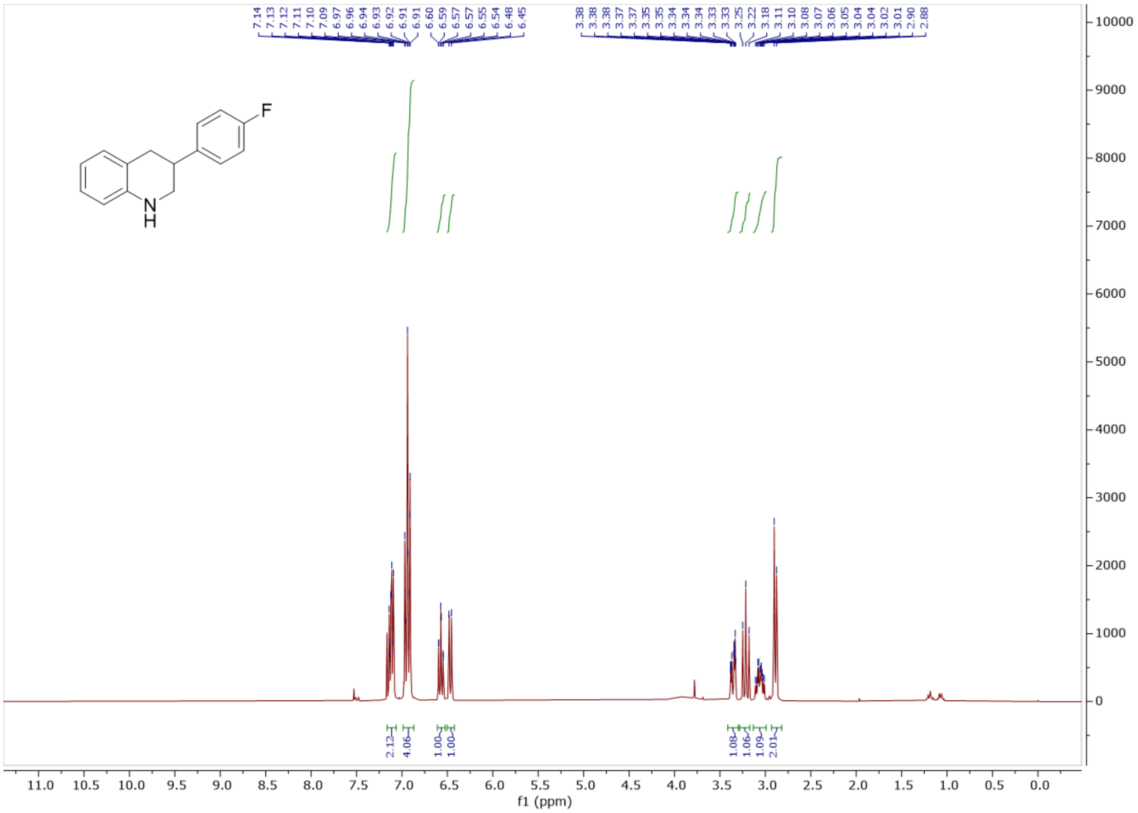

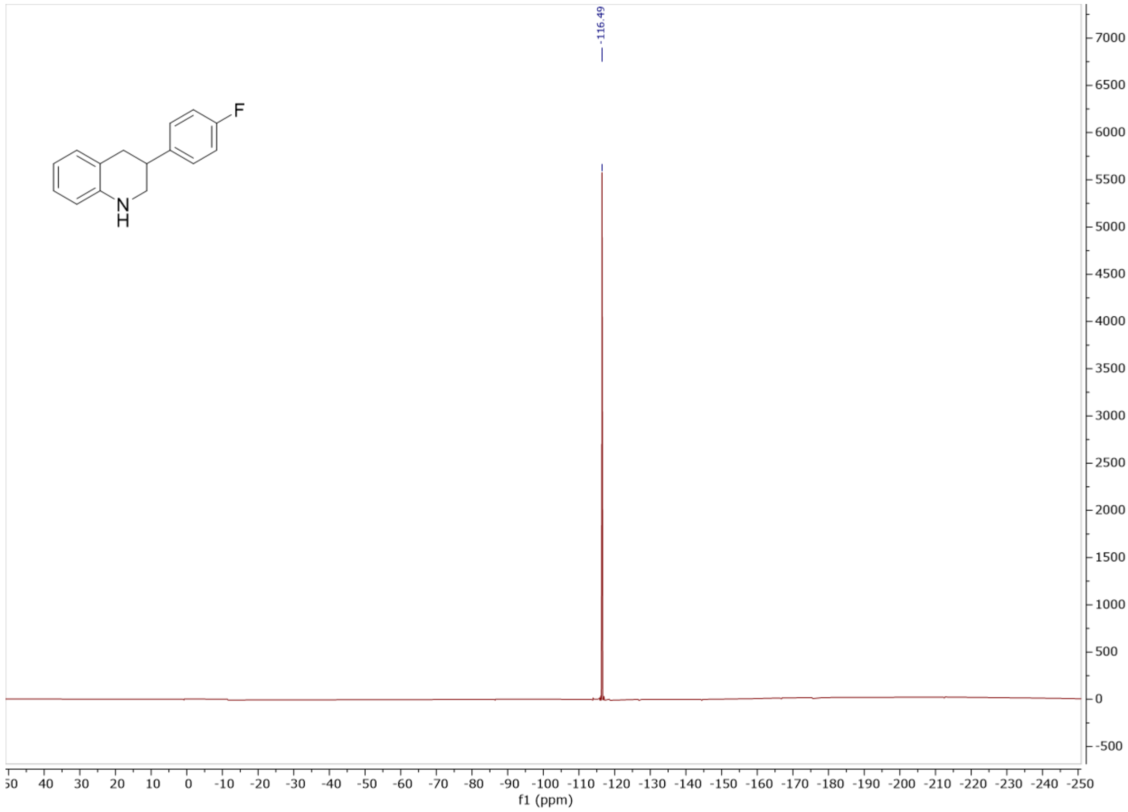

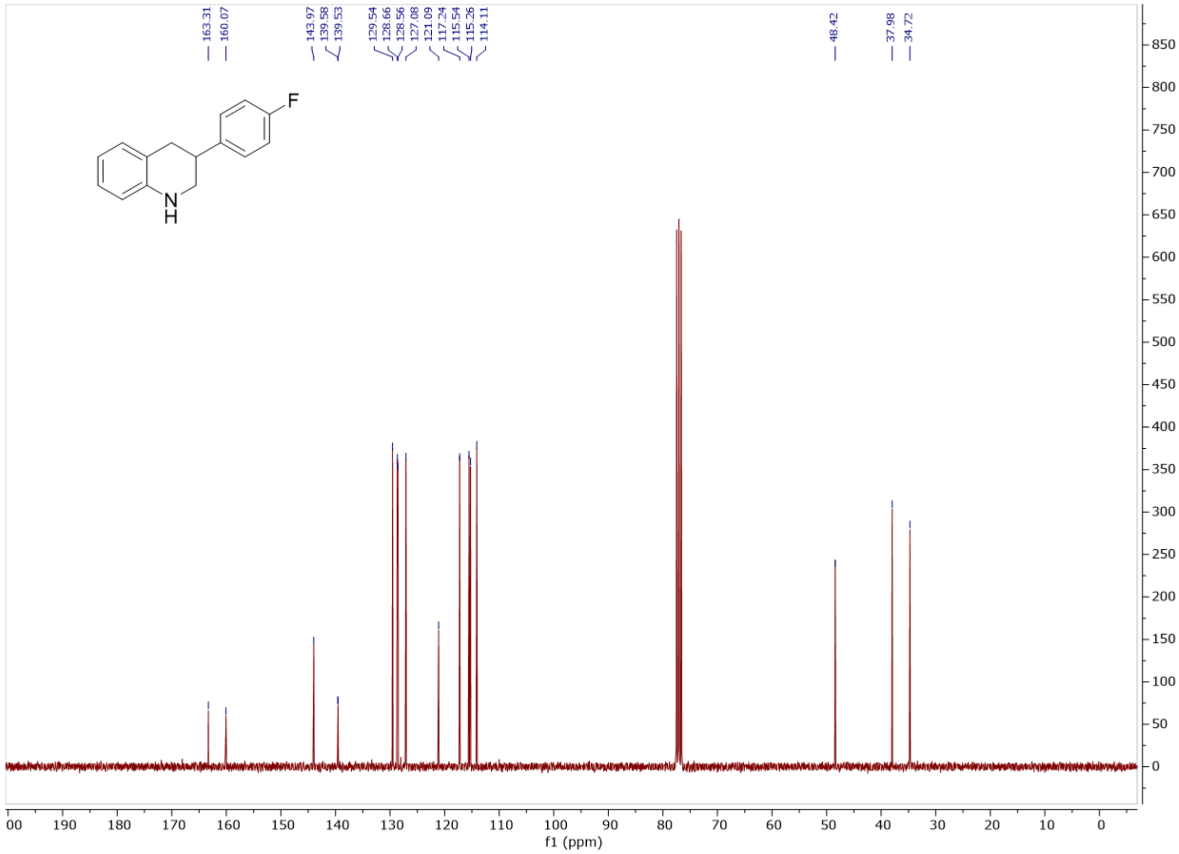
163.31
160.07
143.97
139.58
139.53
129.54
128.66
128.56
127.08
121.09
117.24
115.54
115.26
114.11
48.42
37.98
34.72
F
N
H
f1 (ppm)

^1H NMR (300 MHz, $CDCl_3$) and ^{13}C NMR (76 MHz, $CDCl_3$) spectra for **2u**

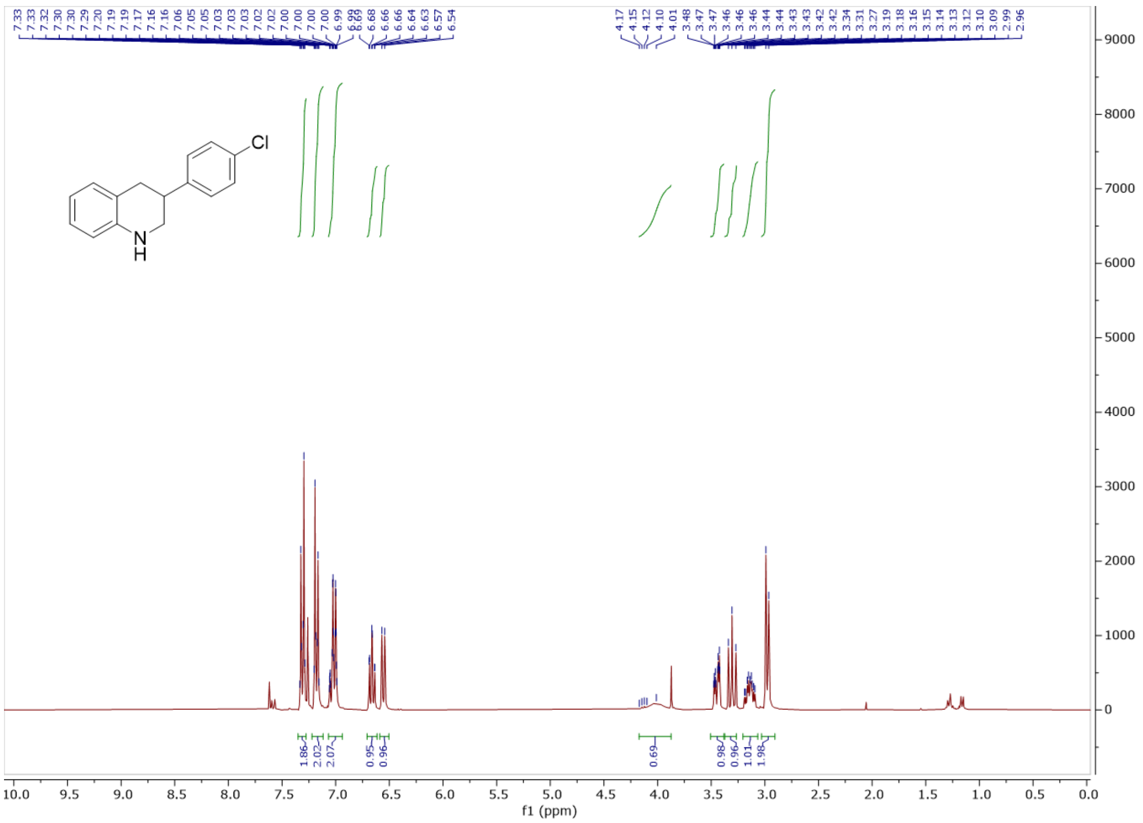

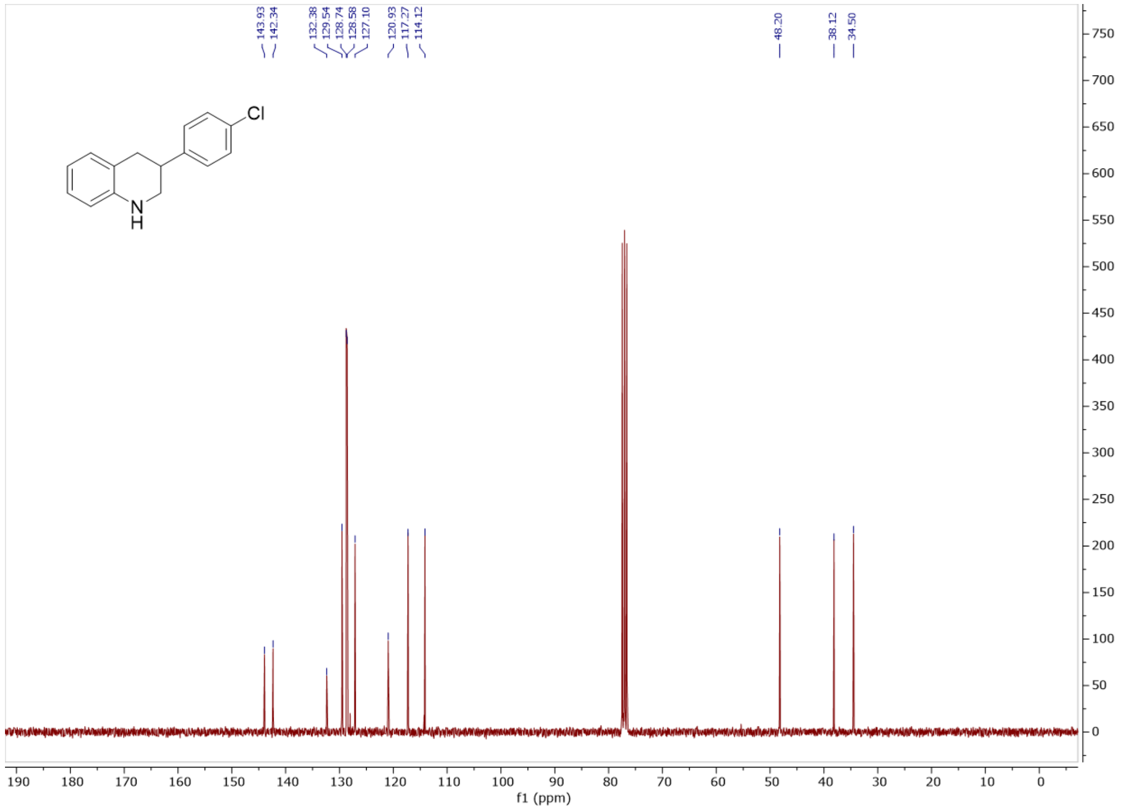

^{1}H NMR (400 MHz, $CDCl_3$) and ^{19}F NMR (376 MHz, $CDCl_3$) spectra for **2v**

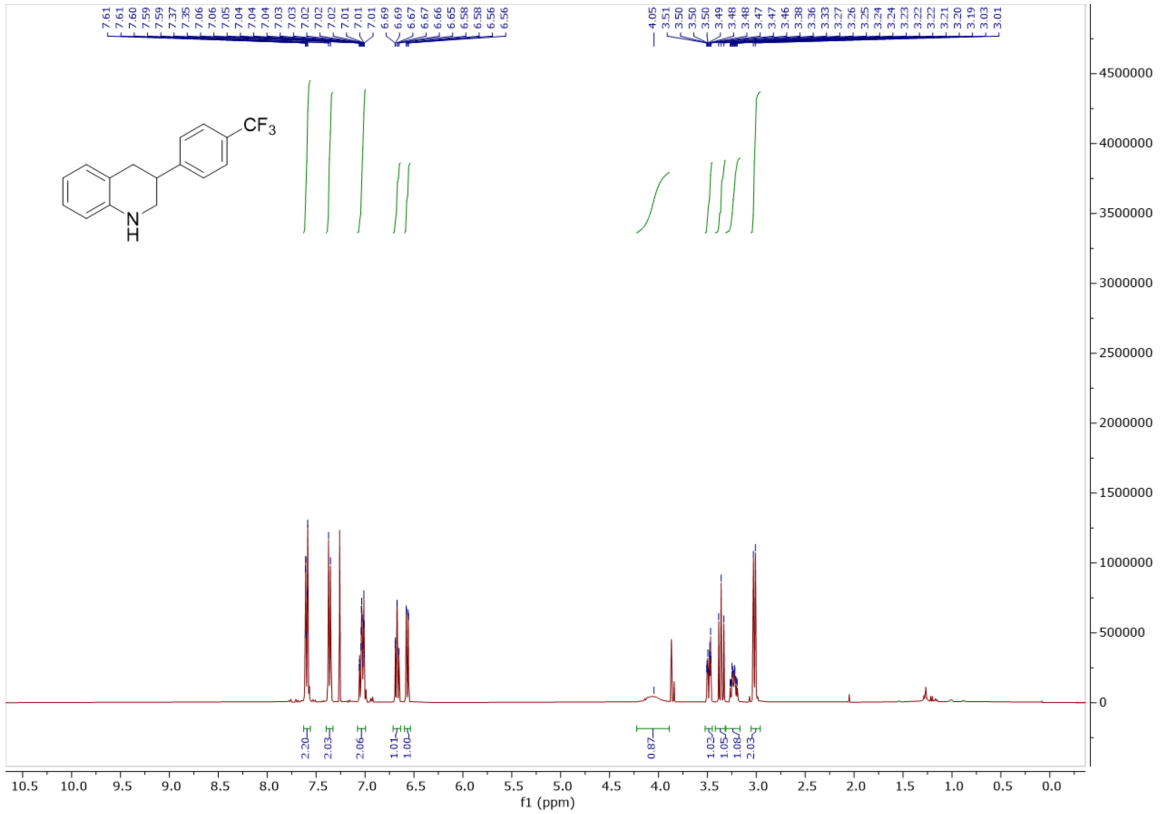

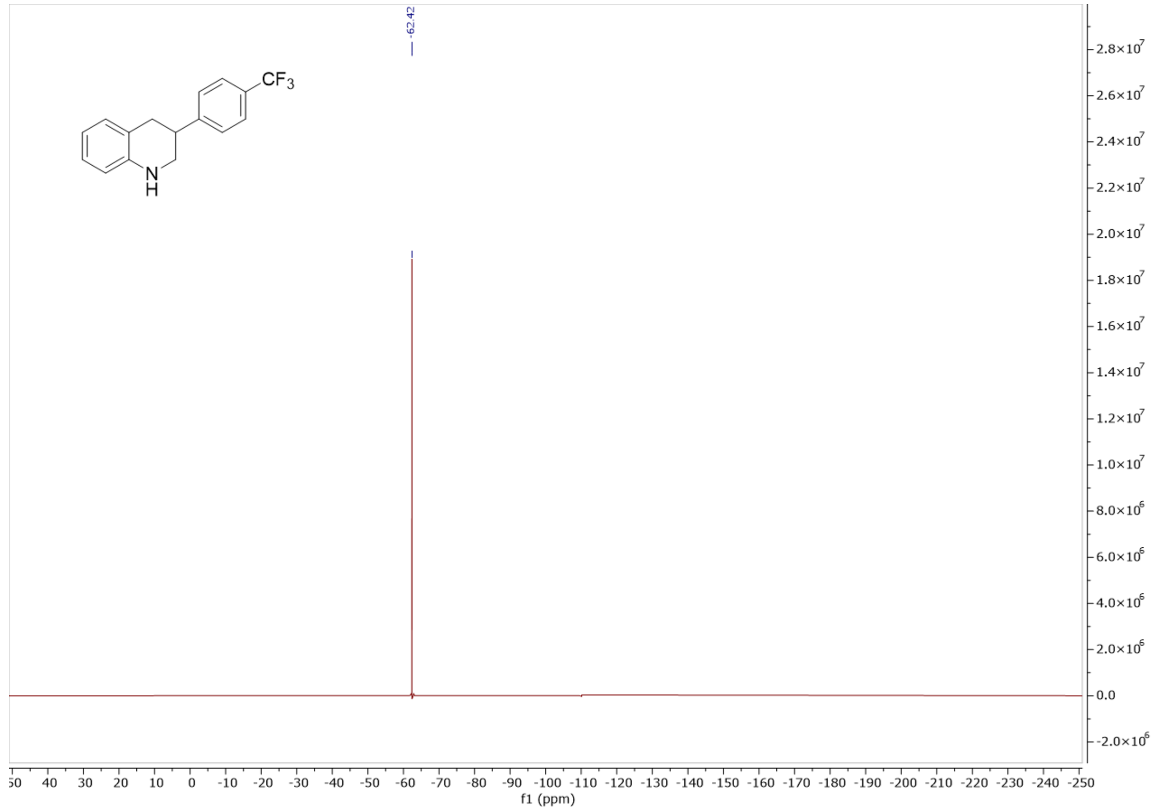

^{1}H NMR (300 MHz, $CDCl_3$) and ^{13}C NMR (76 MHz, $CDCl_3$) spectra for **2w**

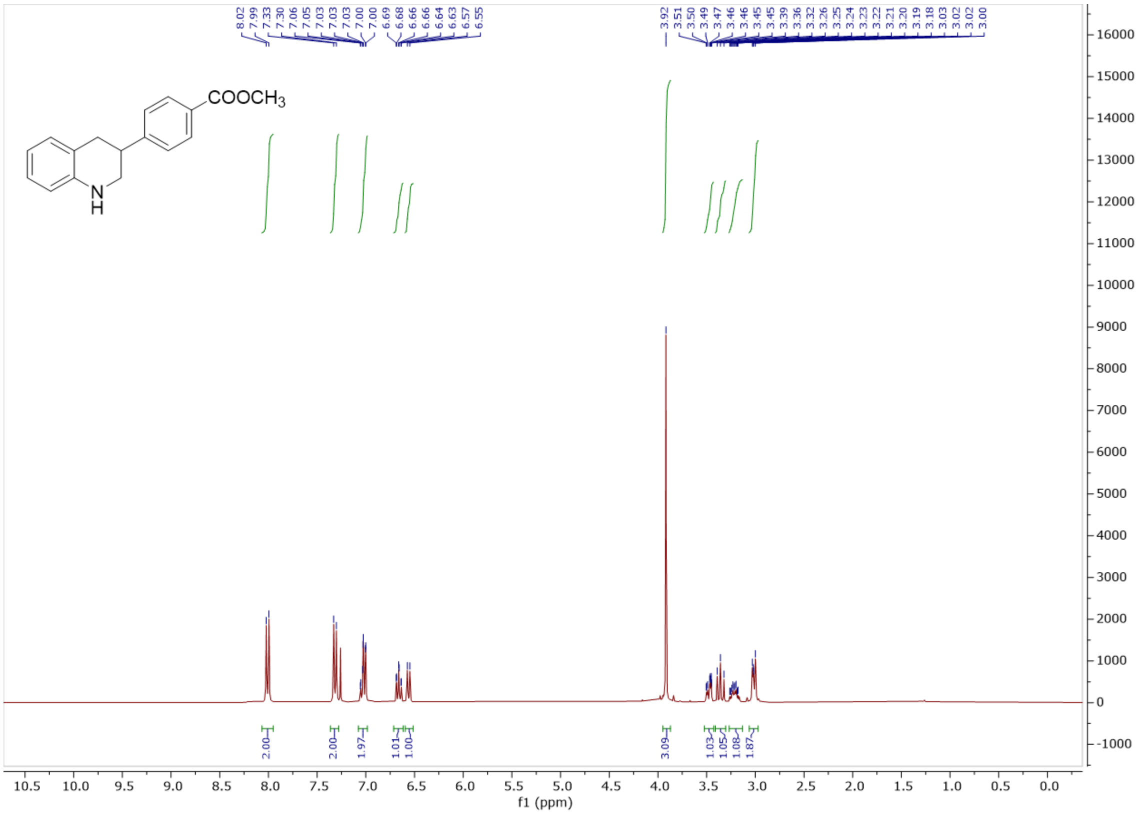

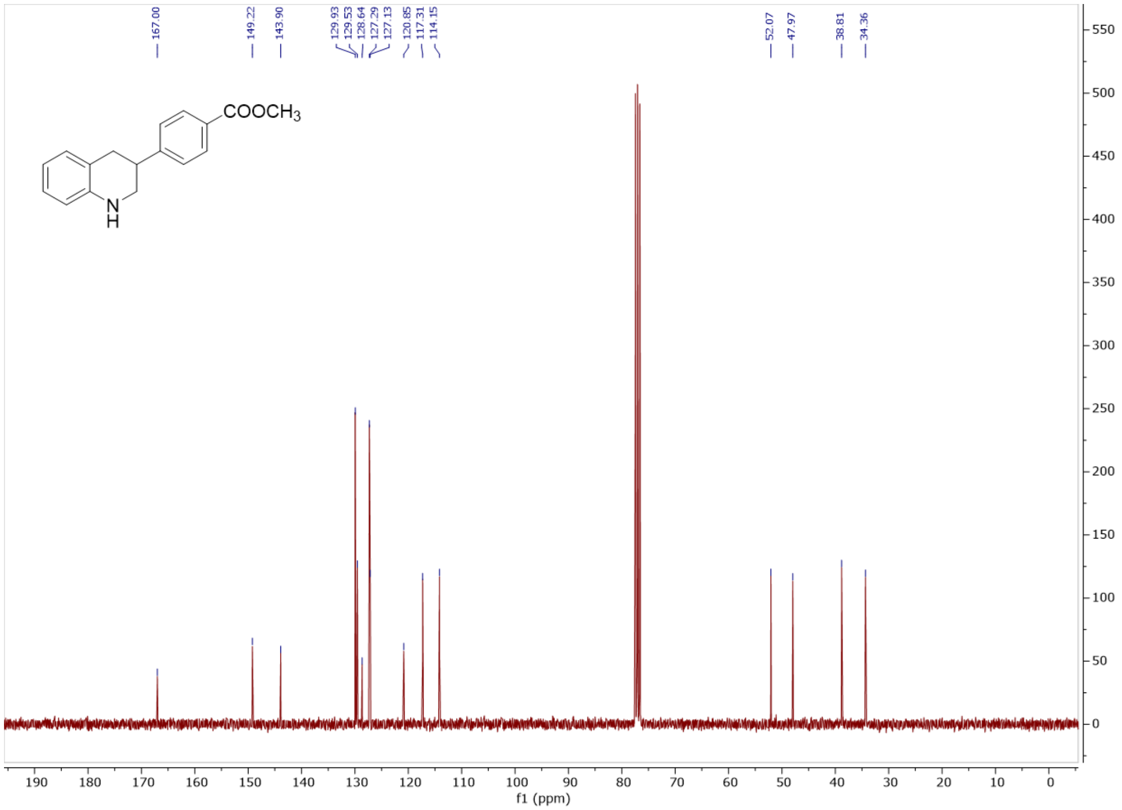

^{1}H NMR (300 MHz, $CDCl_3$) and ^{13}C NMR (76 MHz, $CDCl_3$) spectra for **2x**

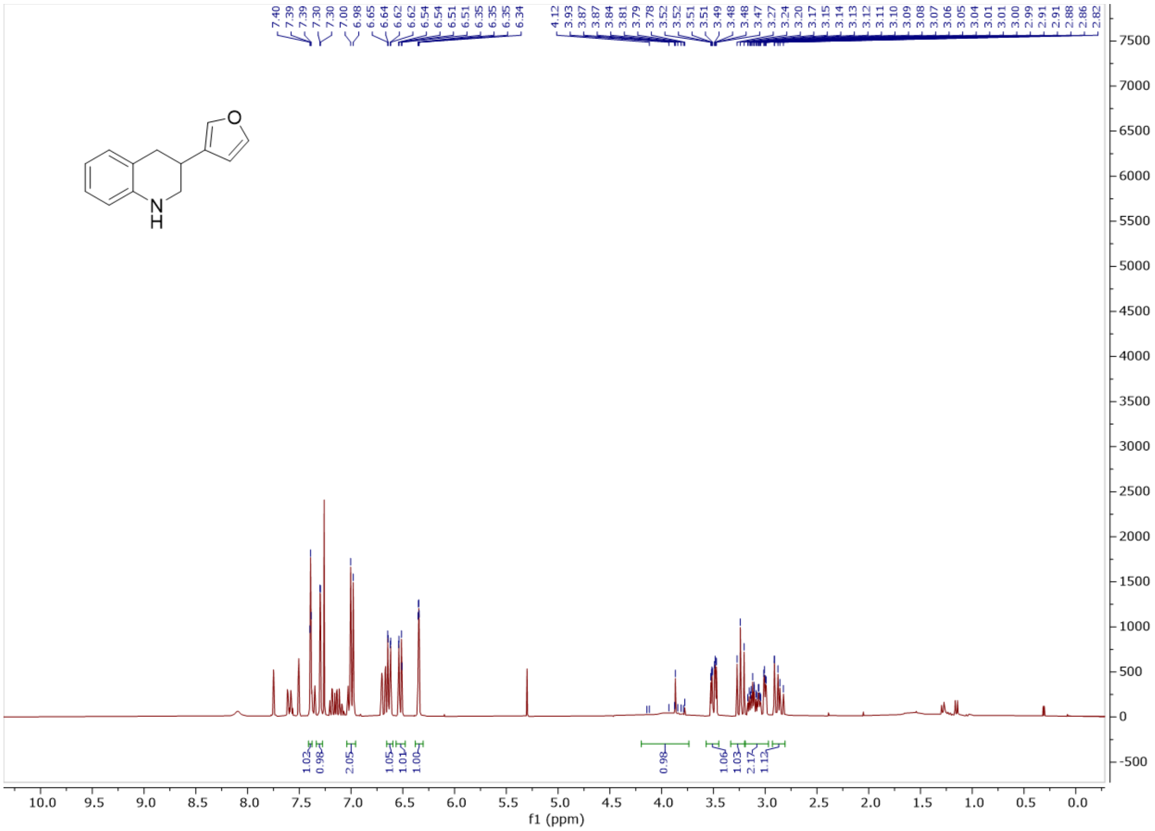

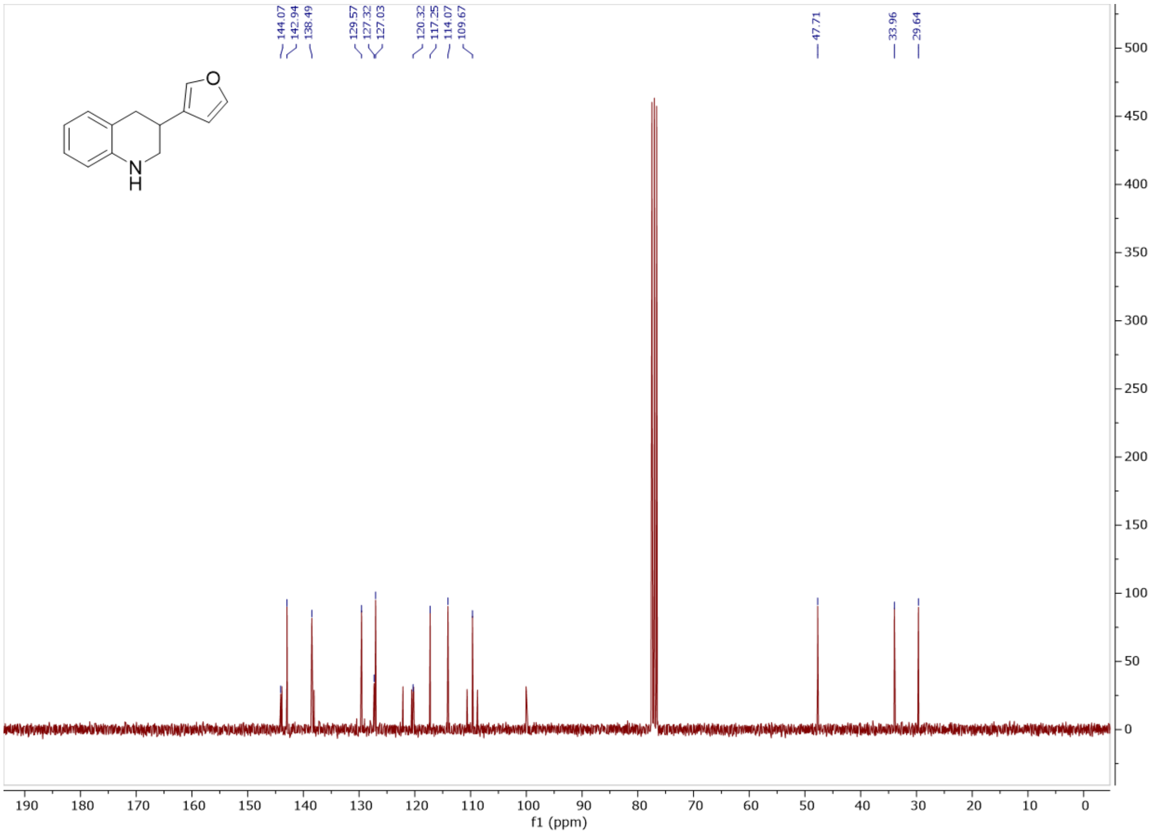

^{1}H NMR (300 MHz, $CDCl_3$) and ^{13}C NMR (76 MHz, $CDCl_3$) spectra for **2y**

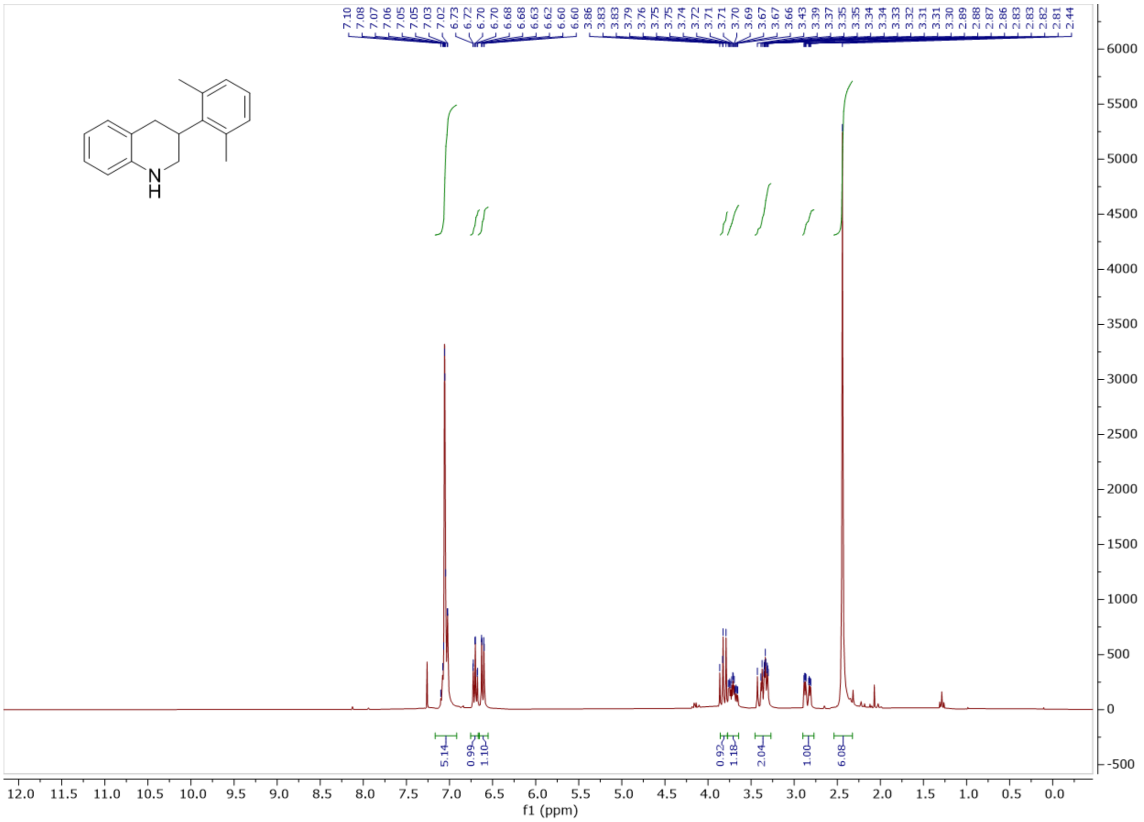

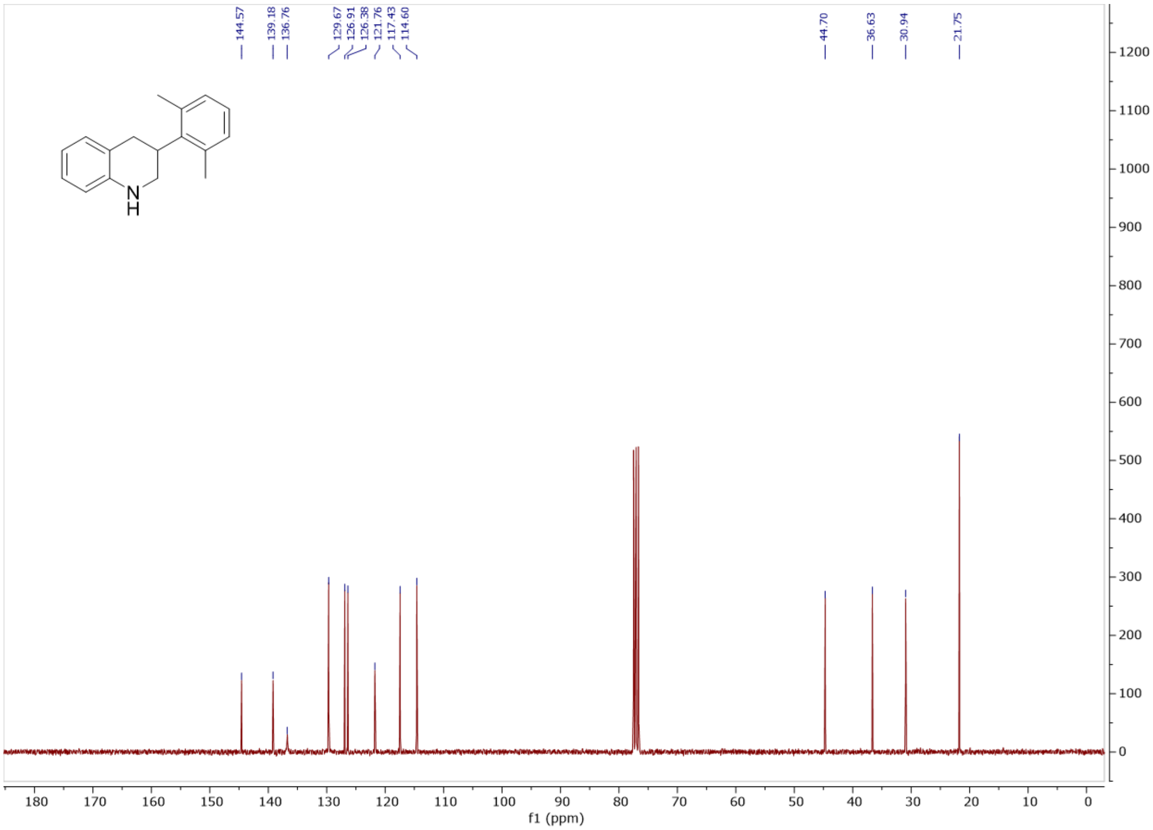

^{1}H NMR (300 MHz, $CDCl_3$) and ^{13}C NMR (76 MHz, $CDCl_3$) spectra for **2z**

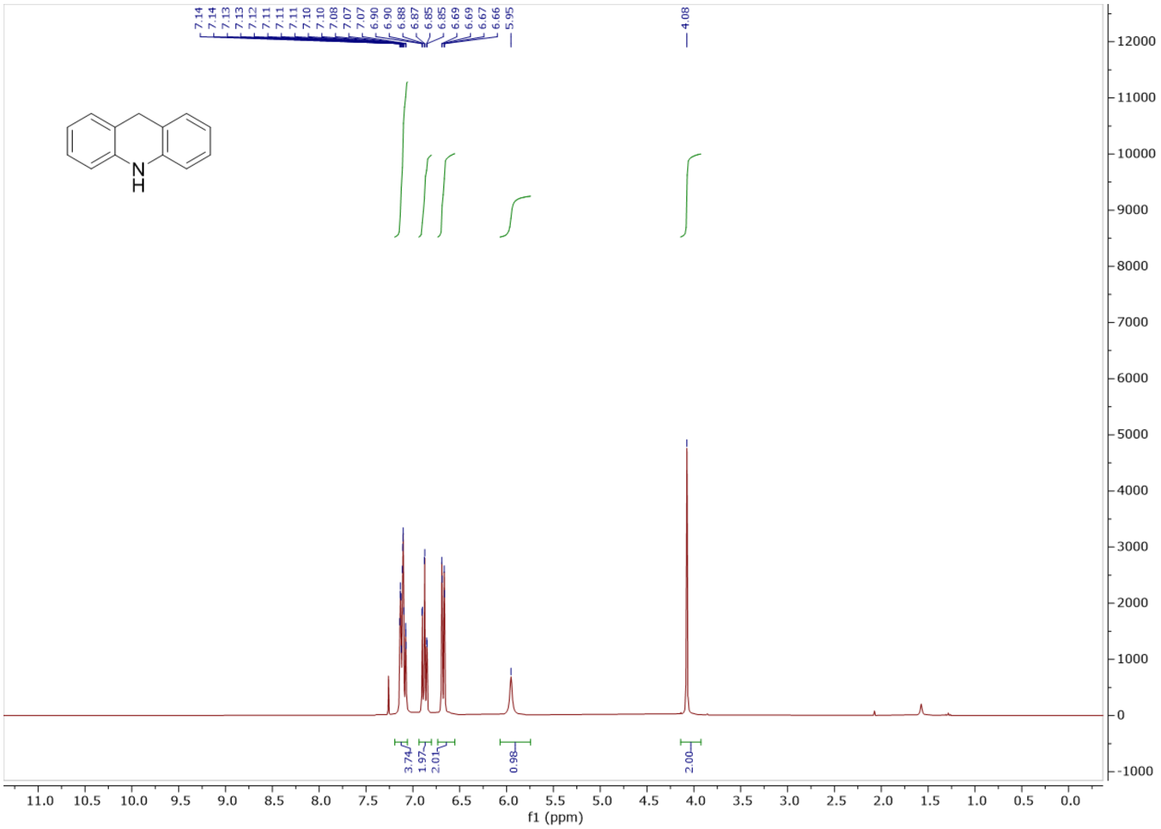

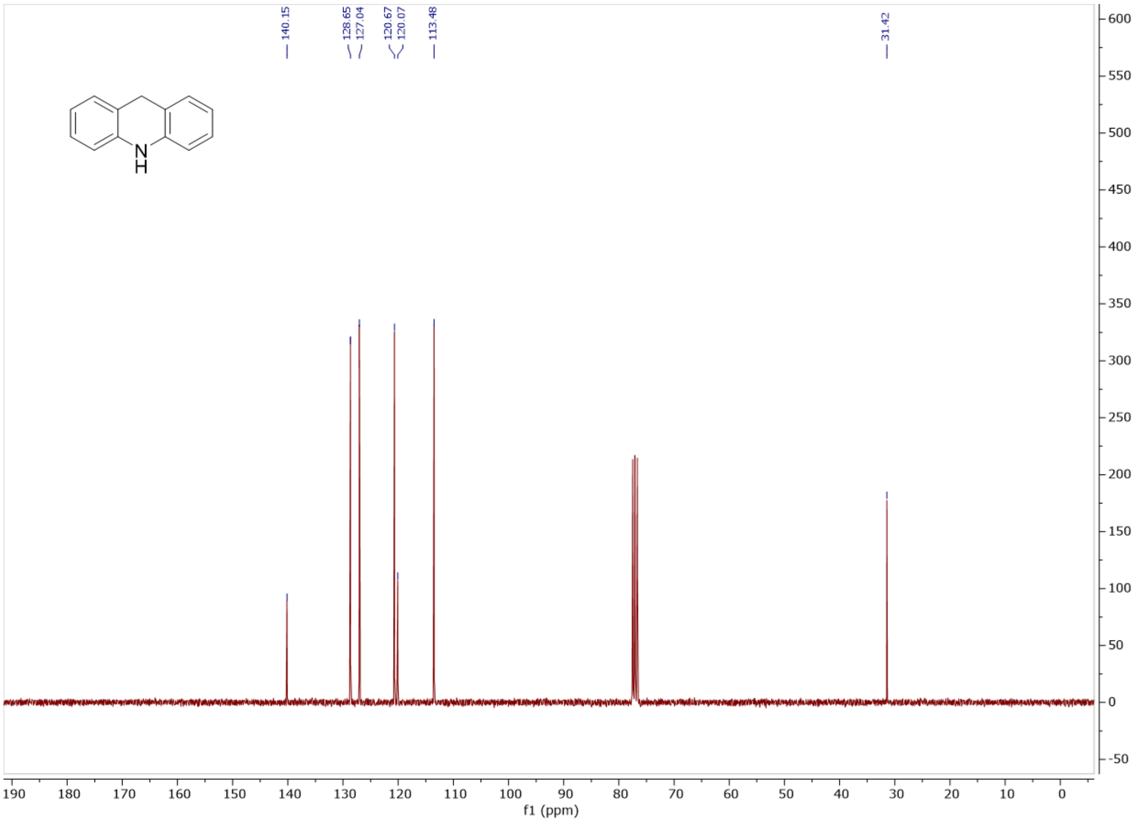

^{1}H NMR (300 MHz, $CDCl_3$) and ^{13}C NMR (76 MHz, $CDCl_3$) spectra for **2aa**

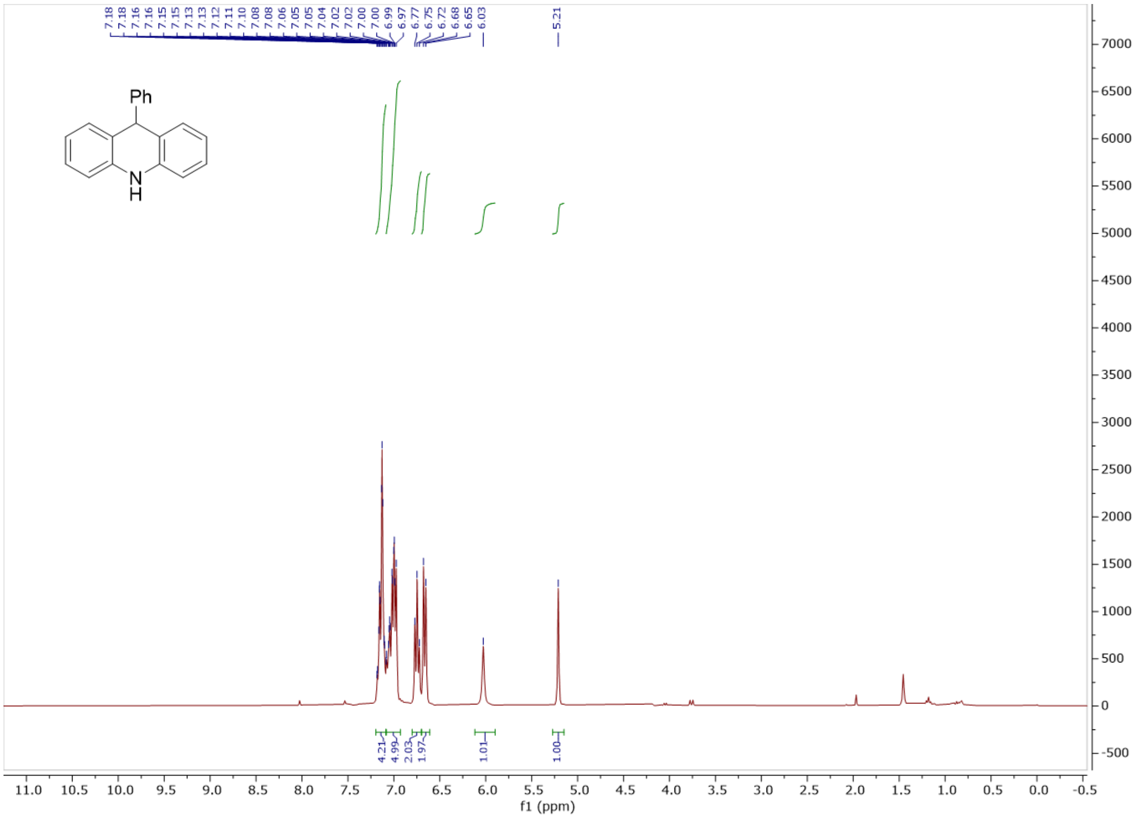

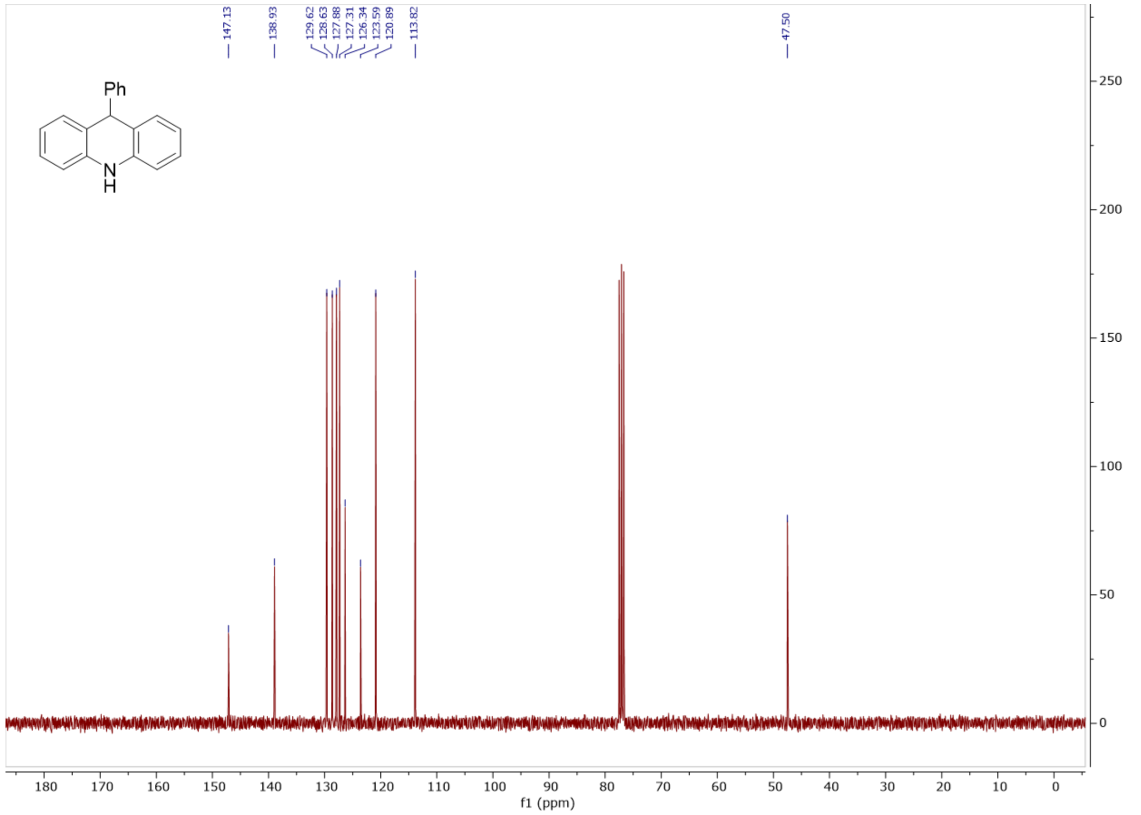

^{1}H NMR (300 MHz, $CDCl_3$) and ^{13}C NMR (76 MHz, $CDCl_3$) spectra for **3a**

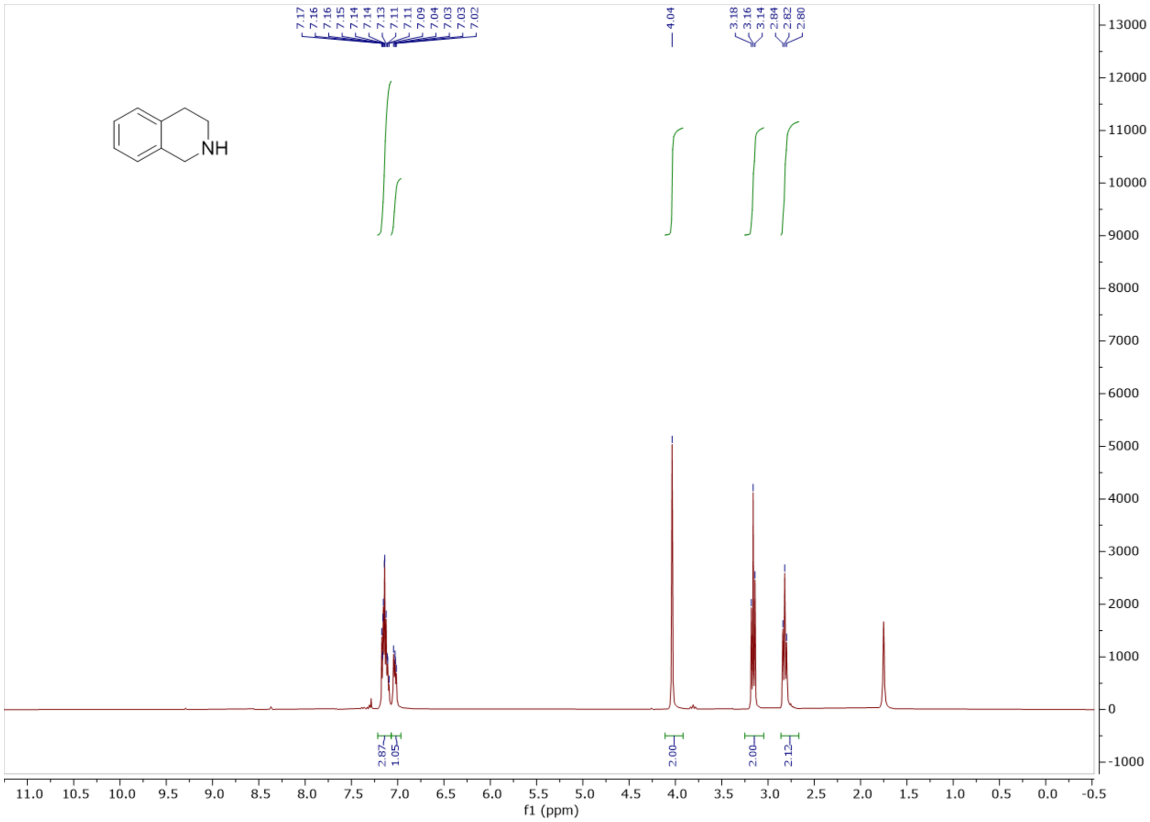

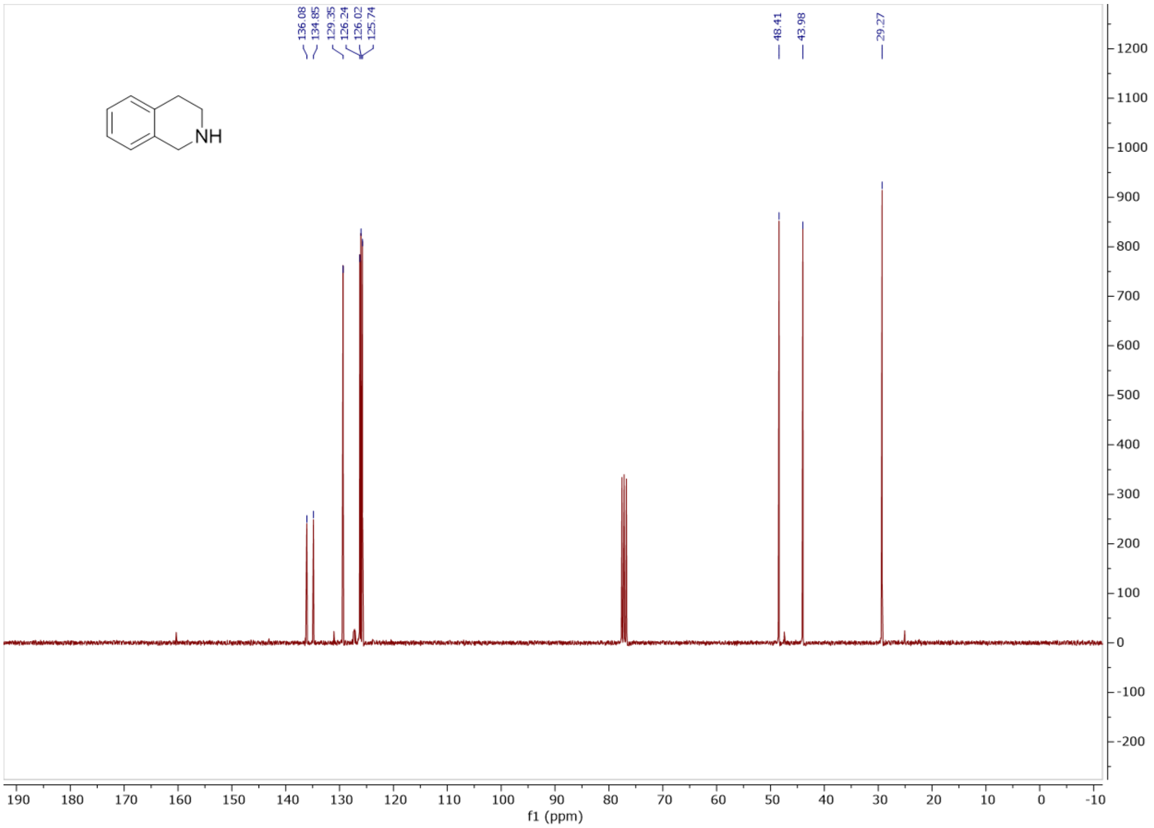

^{1}H NMR (300 MHz, $CDCl_3$) and ^{13}C NMR (76 MHz, $CDCl_3$) spectra for **3b**

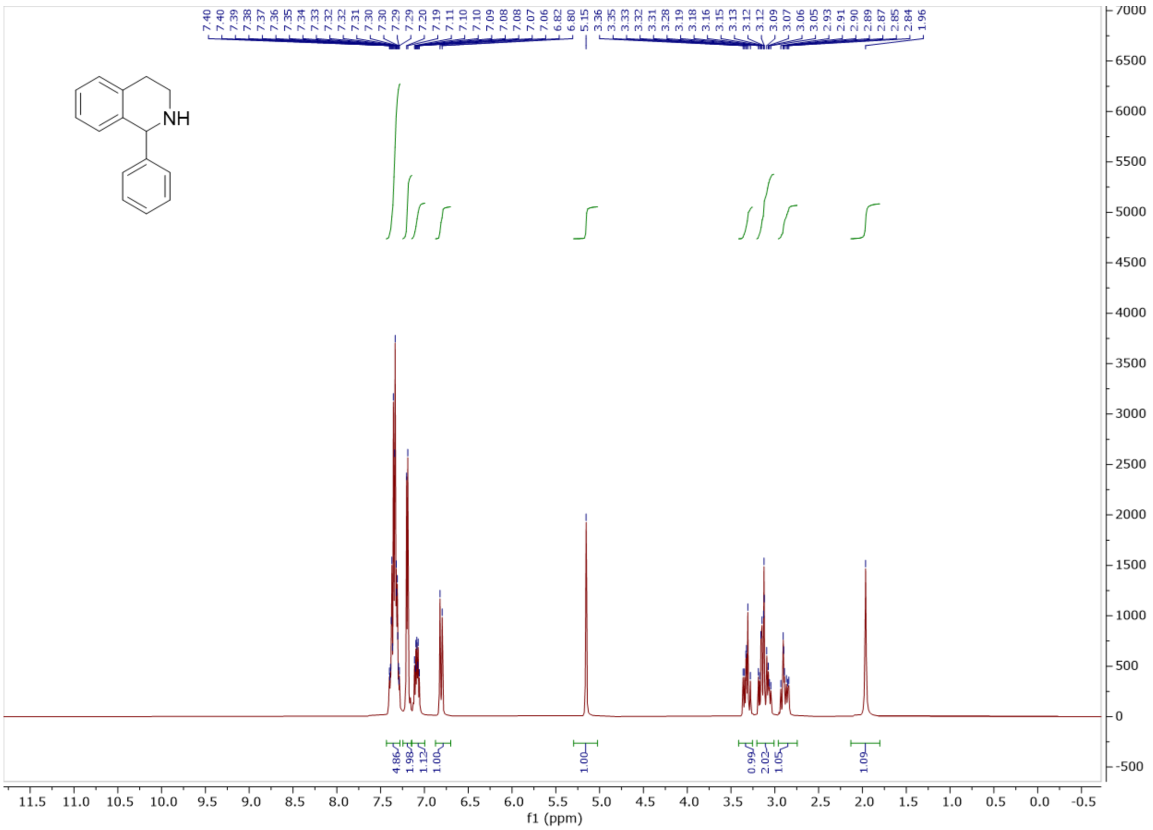

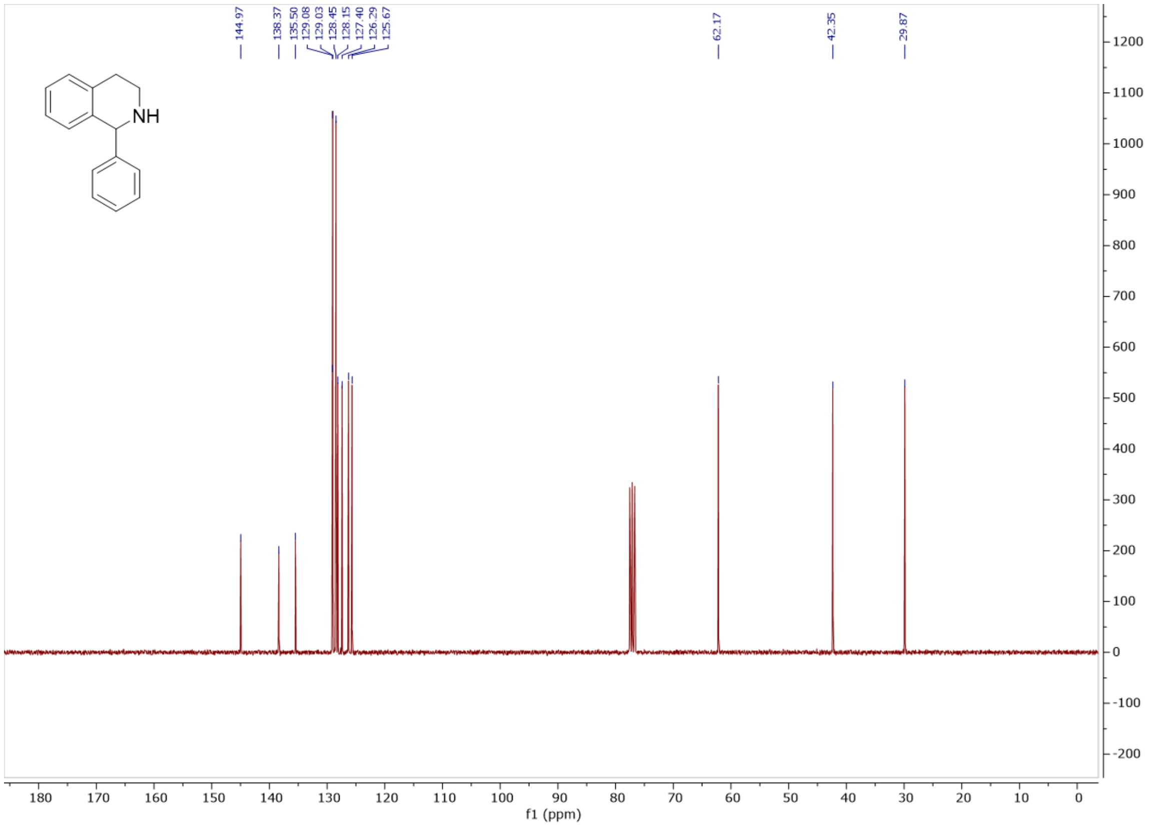

^1H NMR (300 MHz, $CDCl_3$) and ^{13}C NMR (76 MHz, $CDCl_3$) spectra for **2ab**

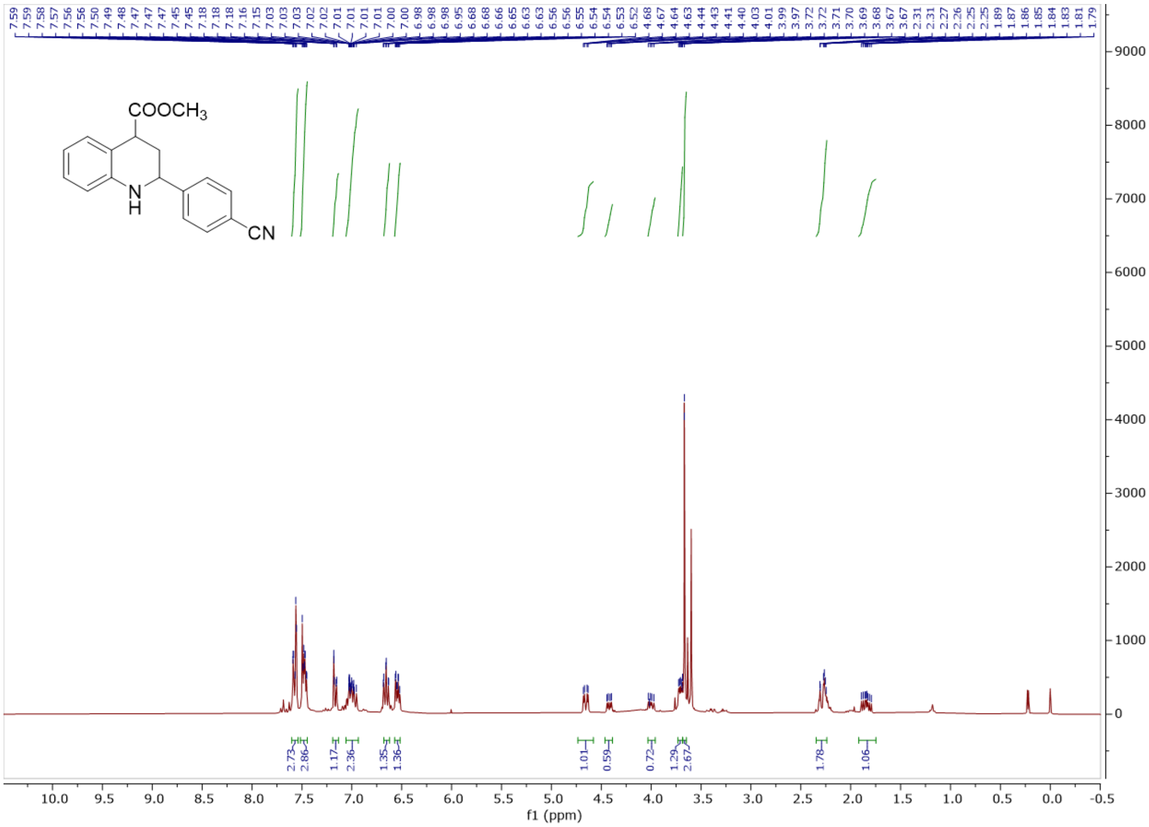

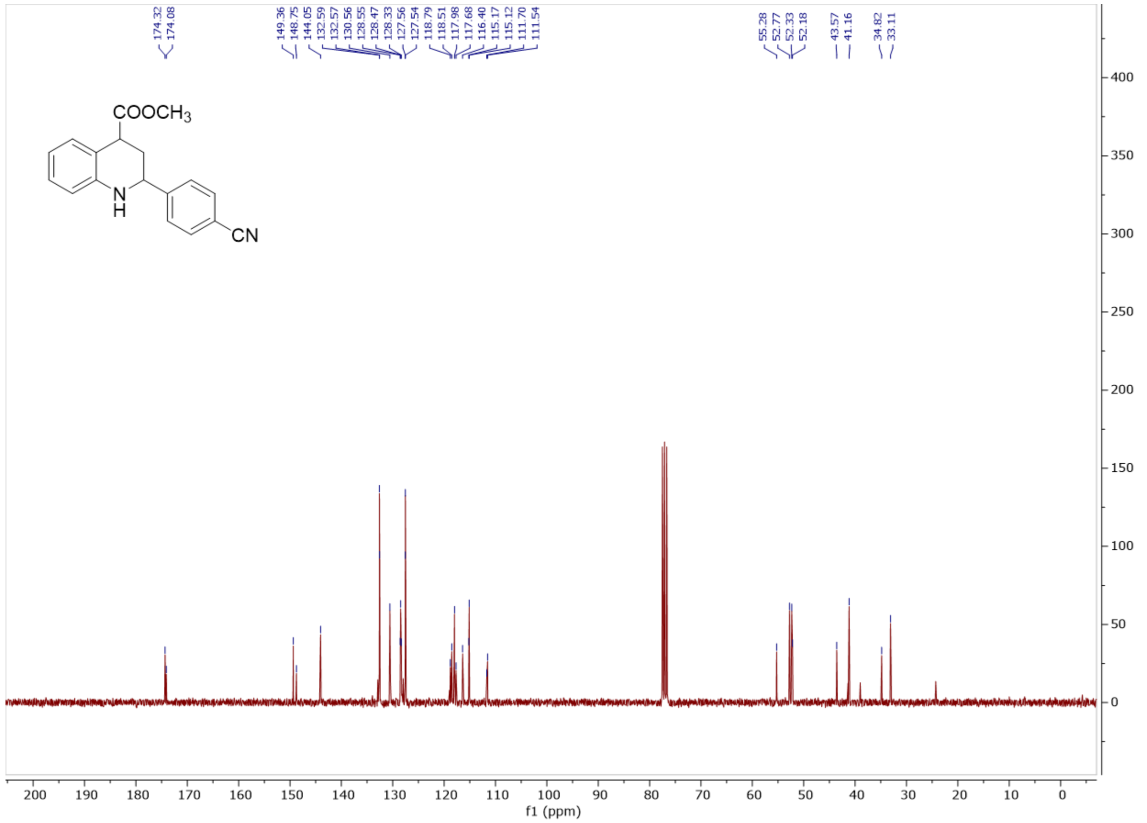

^{1}H NMR (300 MHz, $CDCl_3$) and ^{13}C NMR (76 MHz, $CDCl_3$) spectra for **2ac**

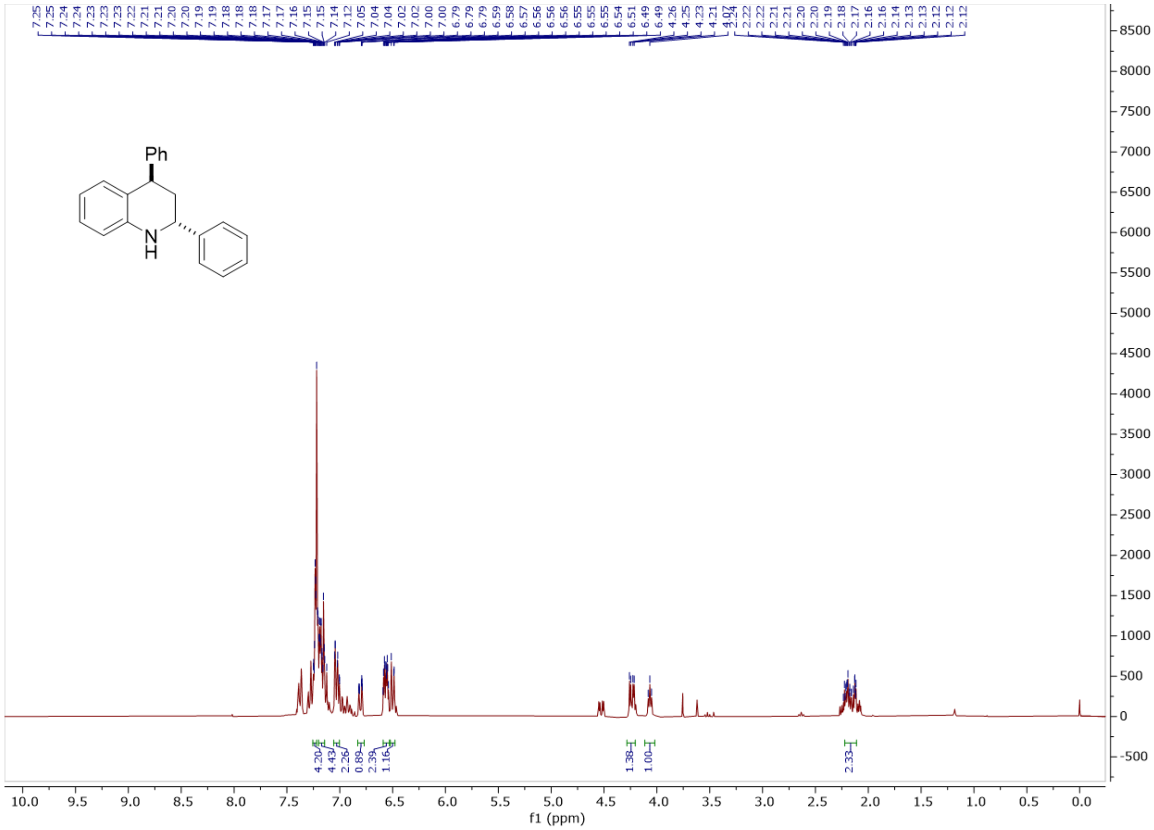

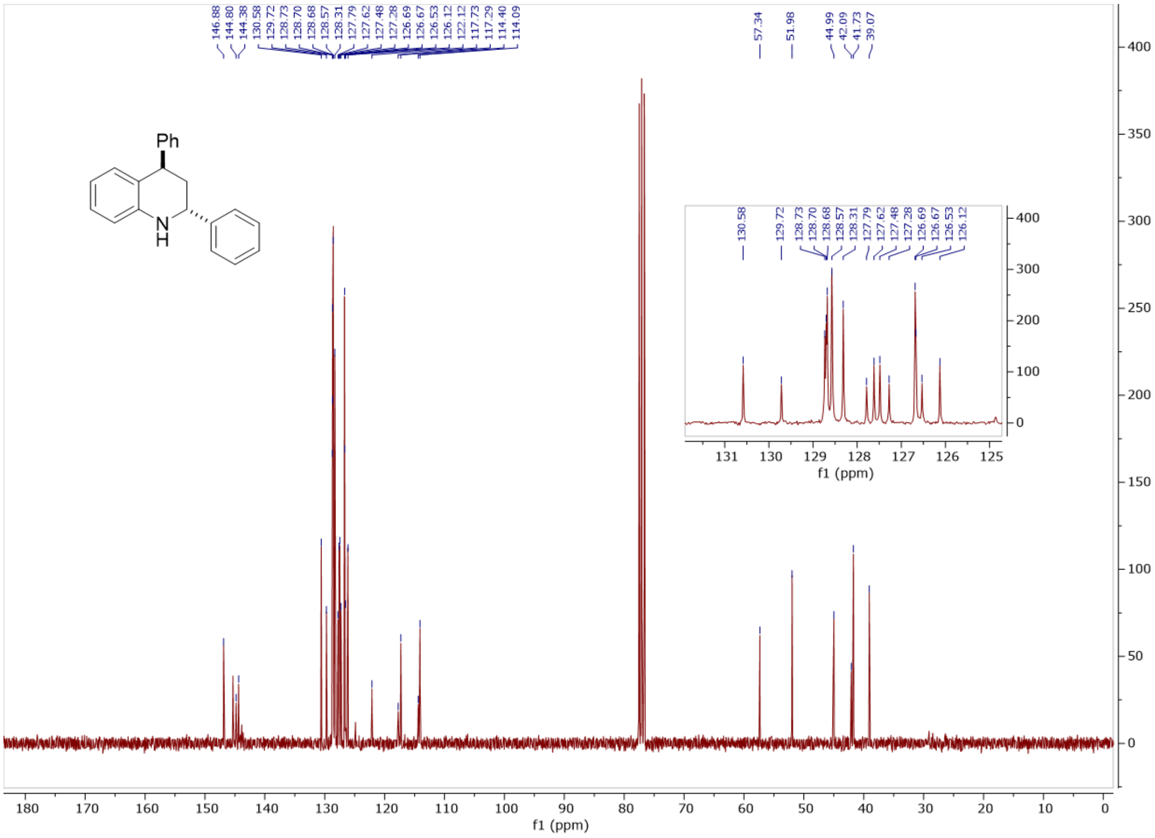

^{1}H NMR (500 MHz, $CDCl_3$) and ^{13}C NMR (126 MHz, $CDCl_3$) spectra for **2ad**

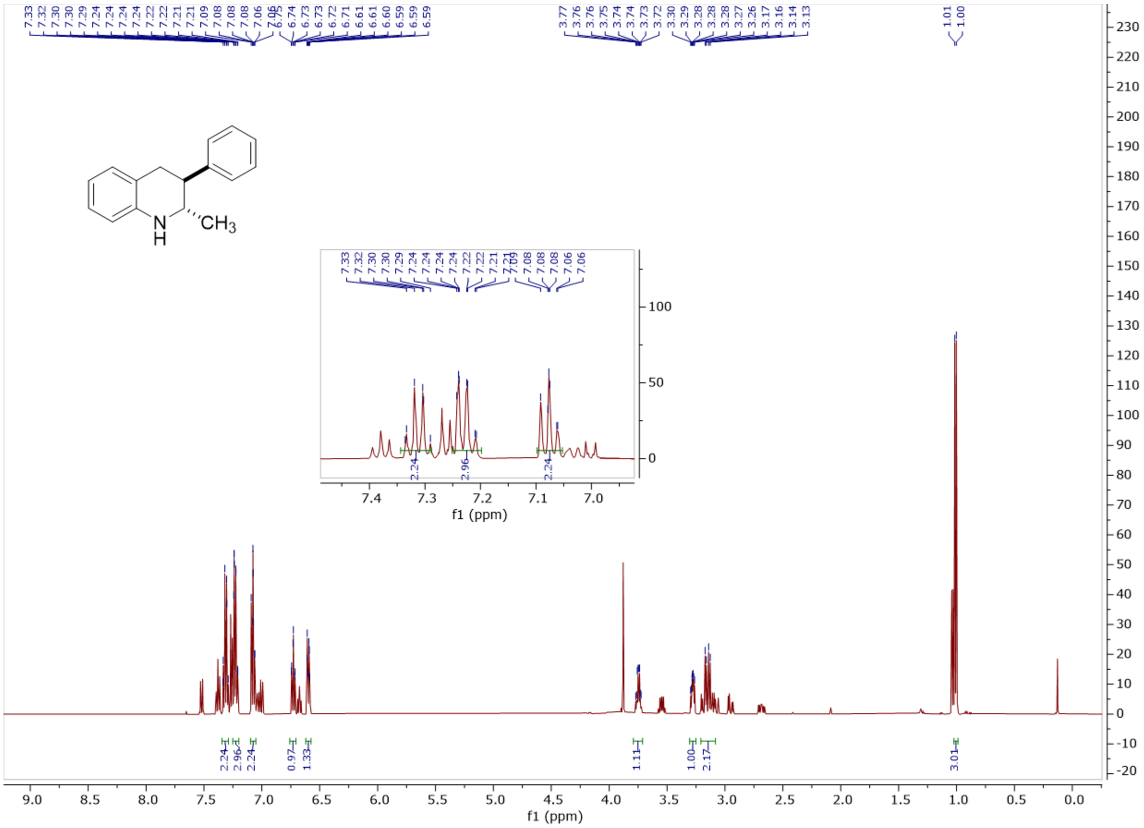

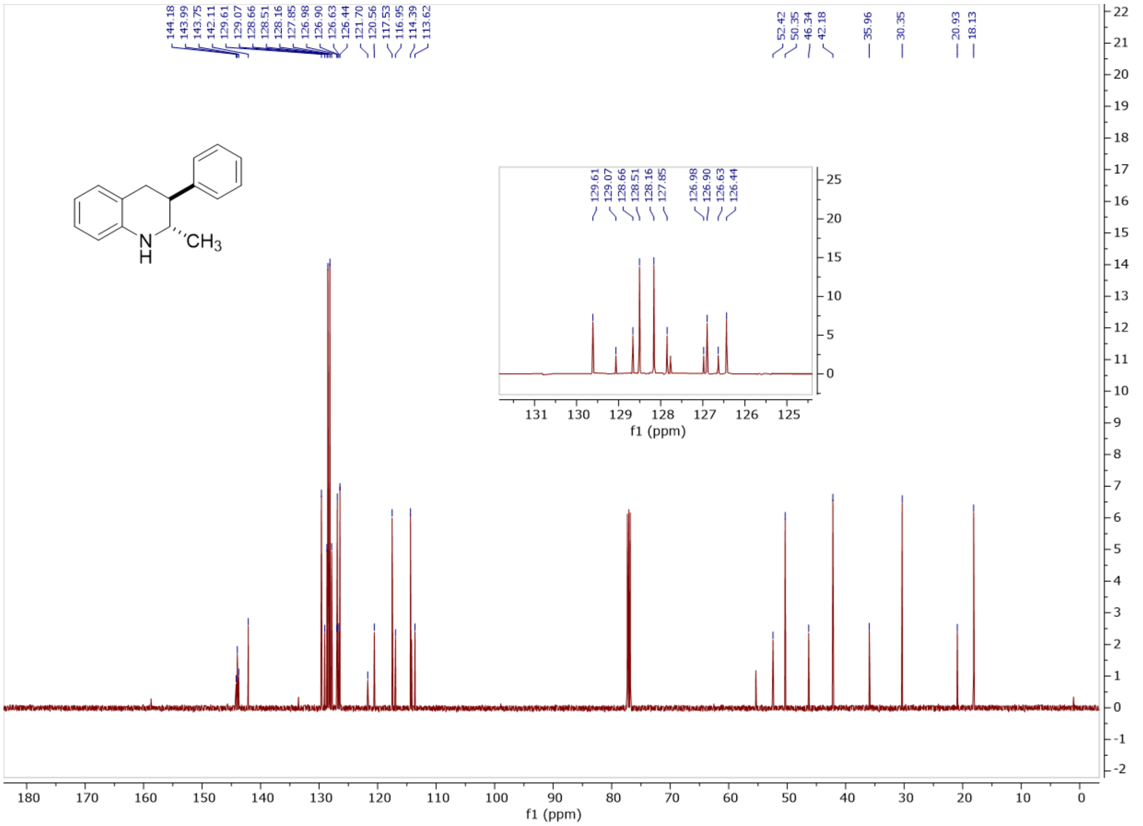

^{1}H NMR (300 MHz, $CDCl_3$) and ^{13}C NMR (76 MHz, $CDCl_3$) spectra for **2ae**

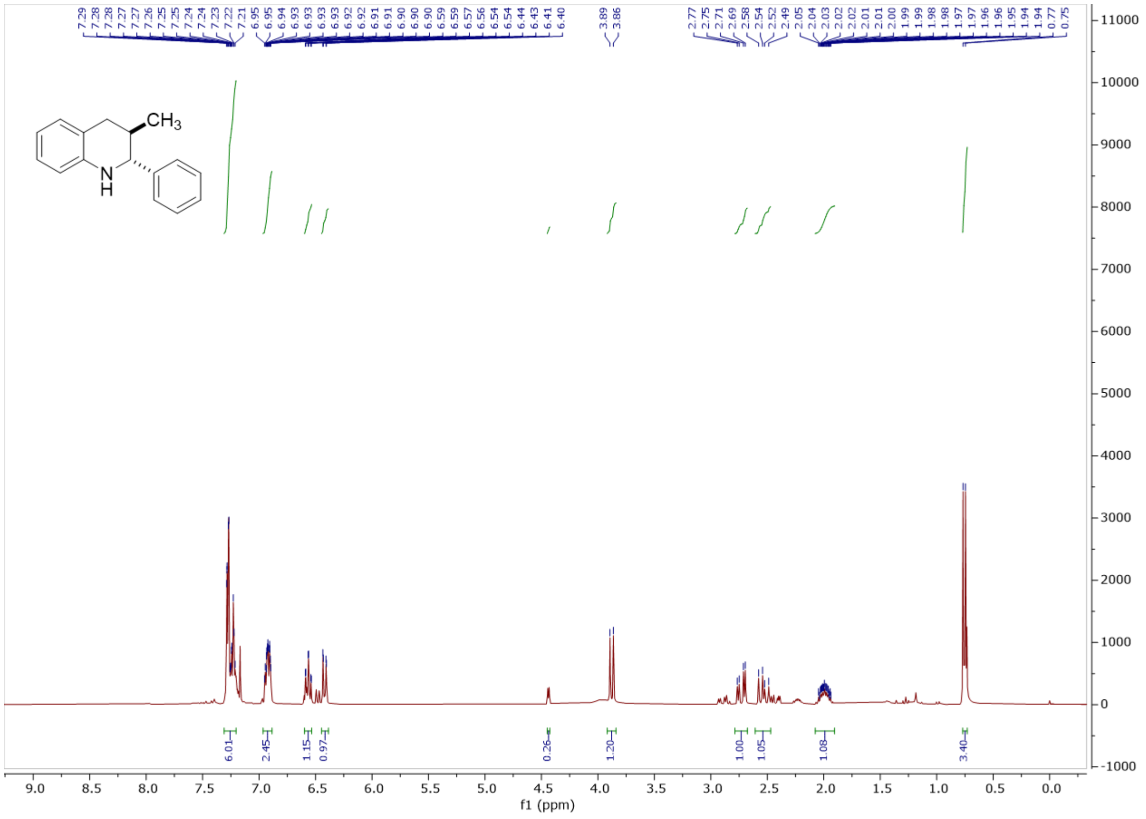

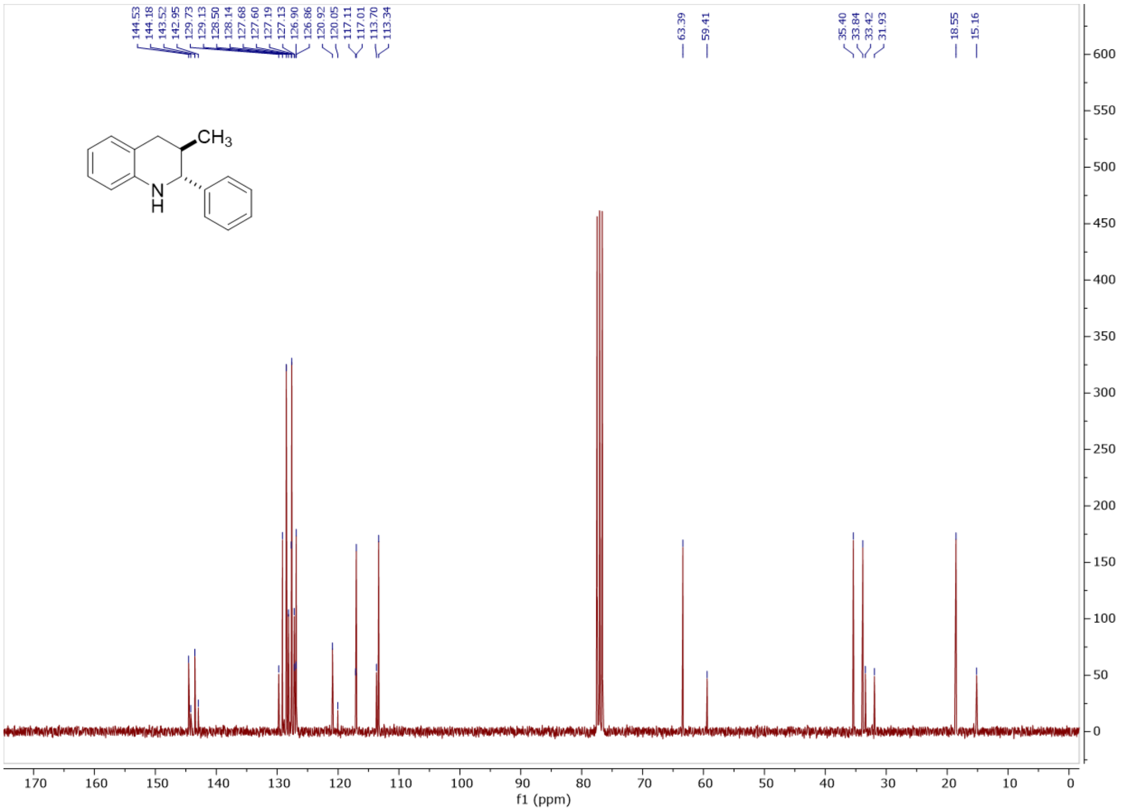

^{1}H NMR (300 MHz, $CDCl_3$) and ^{13}C NMR (76 MHz, $CDCl_3$) spectra for **2af**

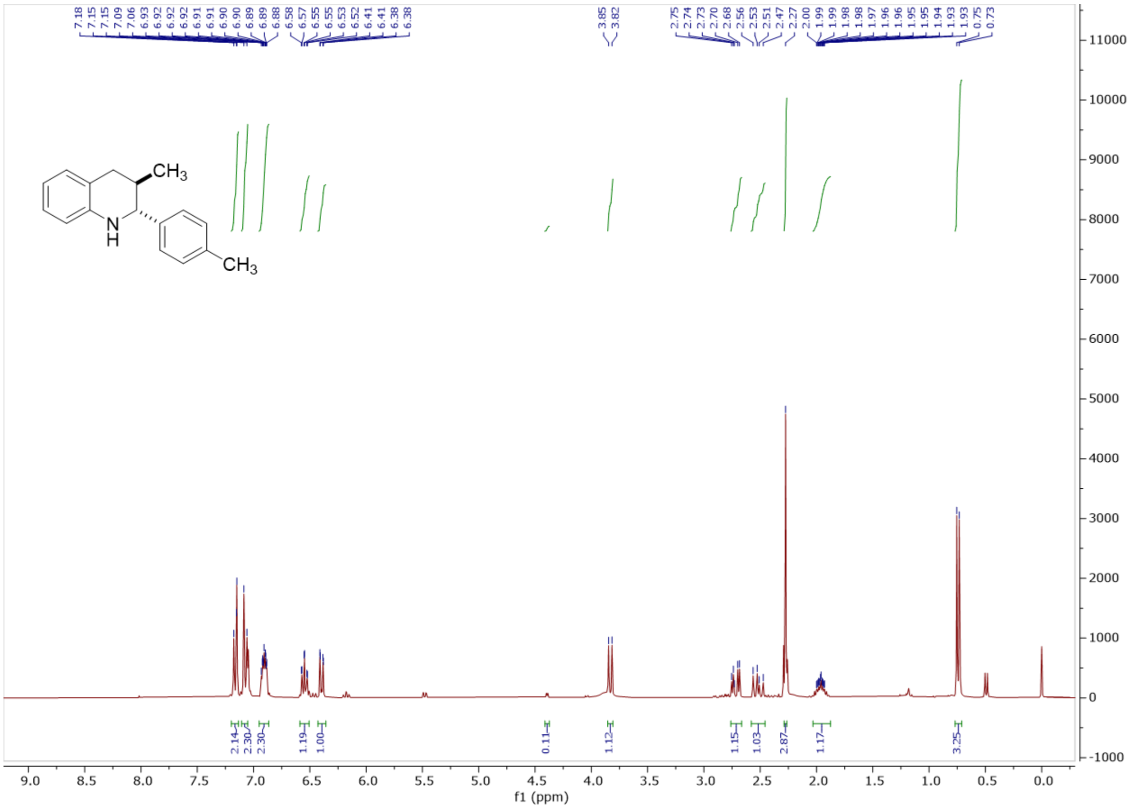

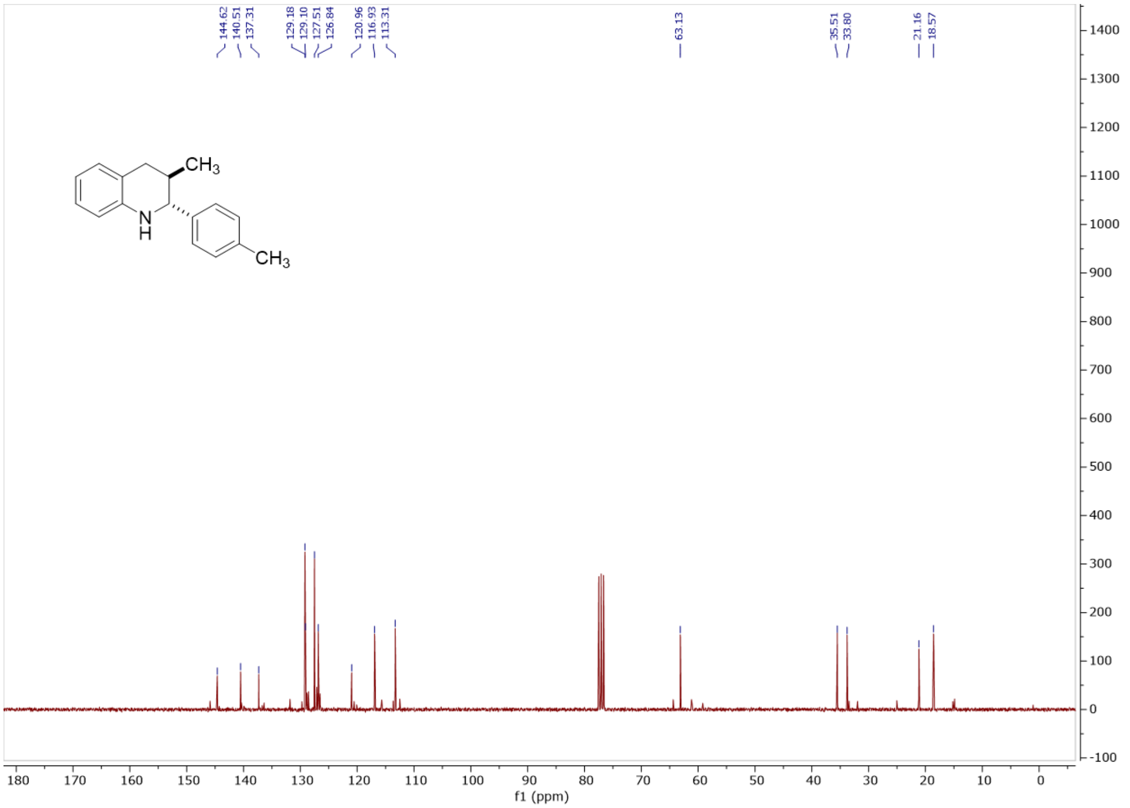

^{1}H NMR (300 MHz, $CDCl_3$) and ^{13}C NMR (76 MHz, $CDCl_3$) spectra for **2ag**

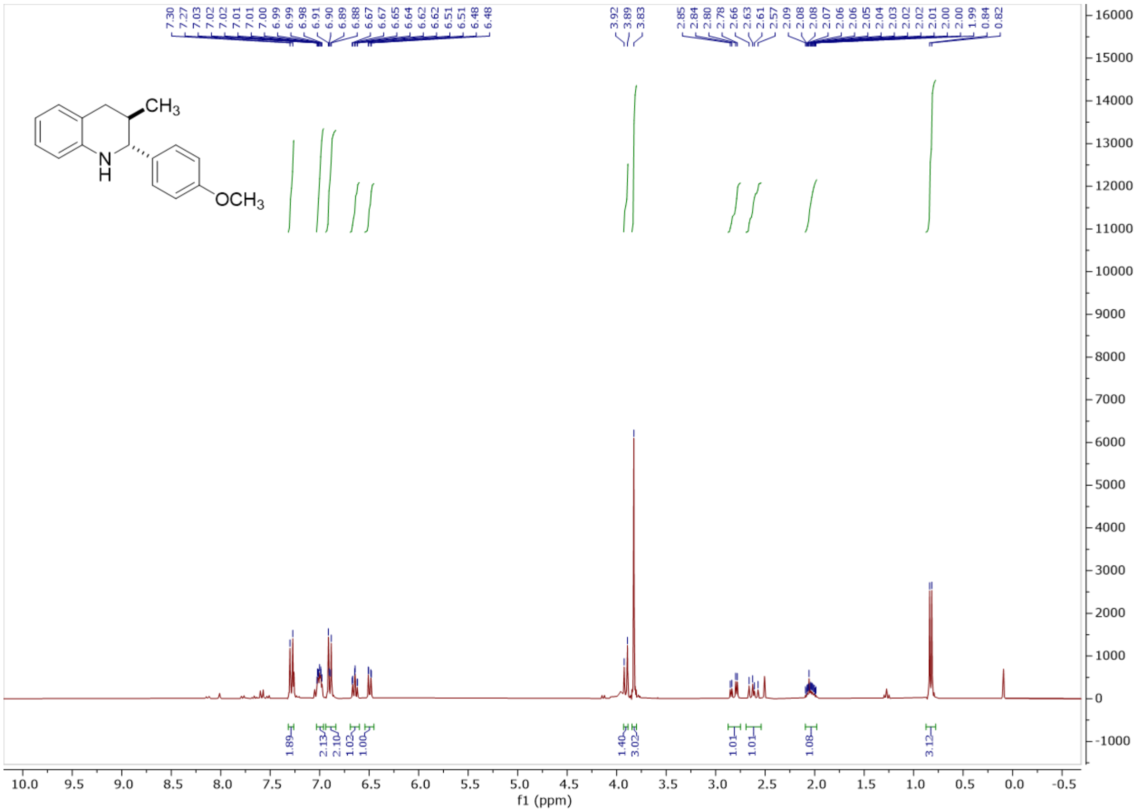

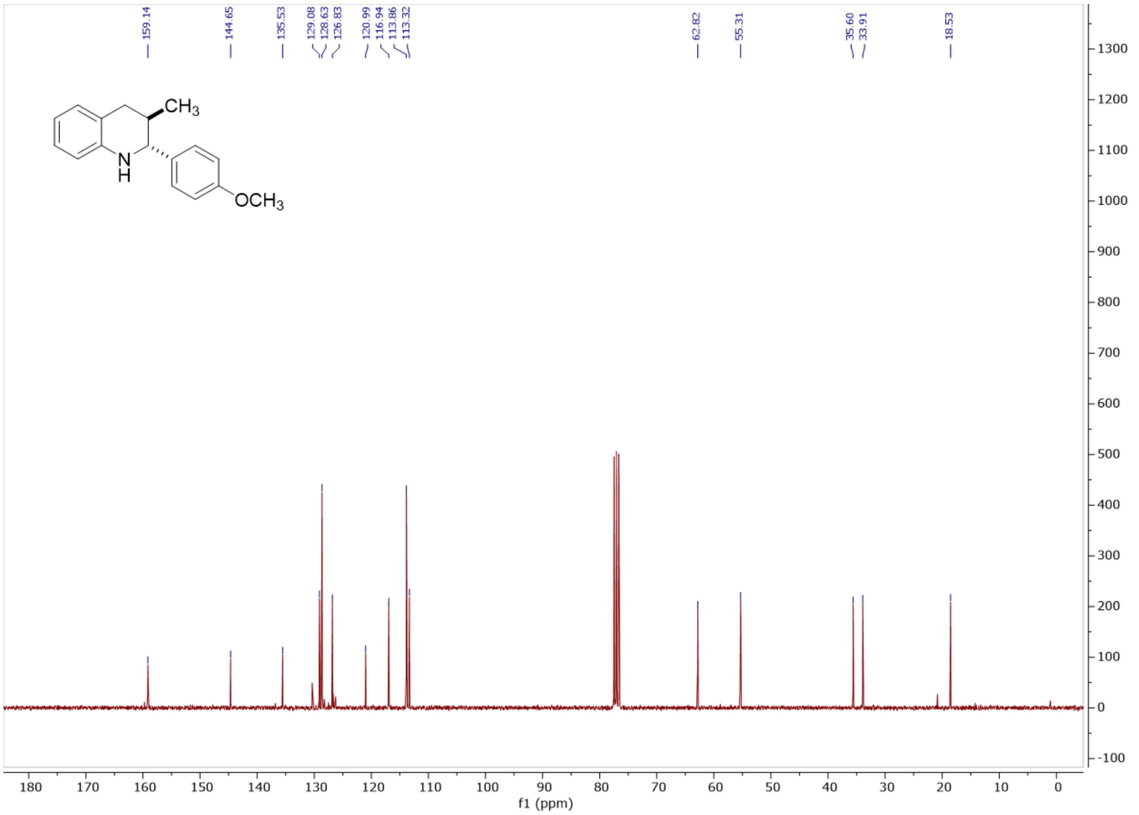

^{1}H NMR (300 MHz, $CDCl_3$) and ^{13}C NMR (101 MHz, $CDCl_3$) spectra for **2ah**

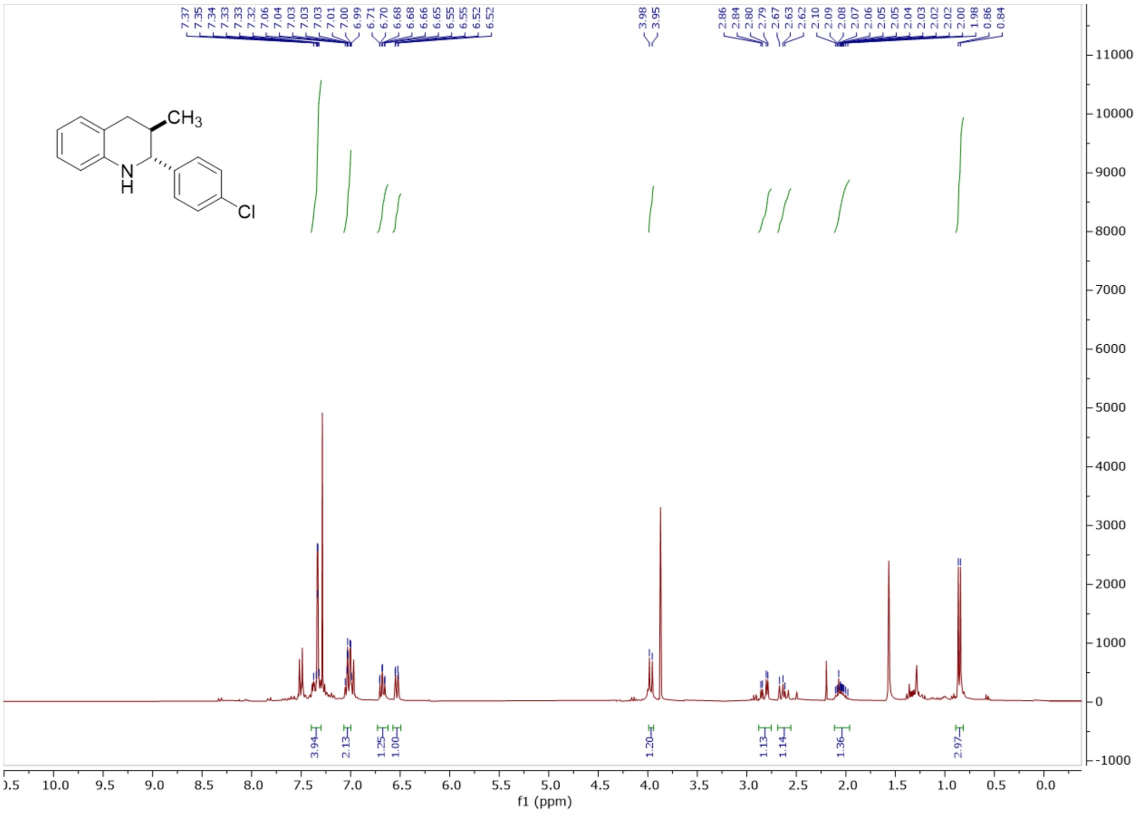

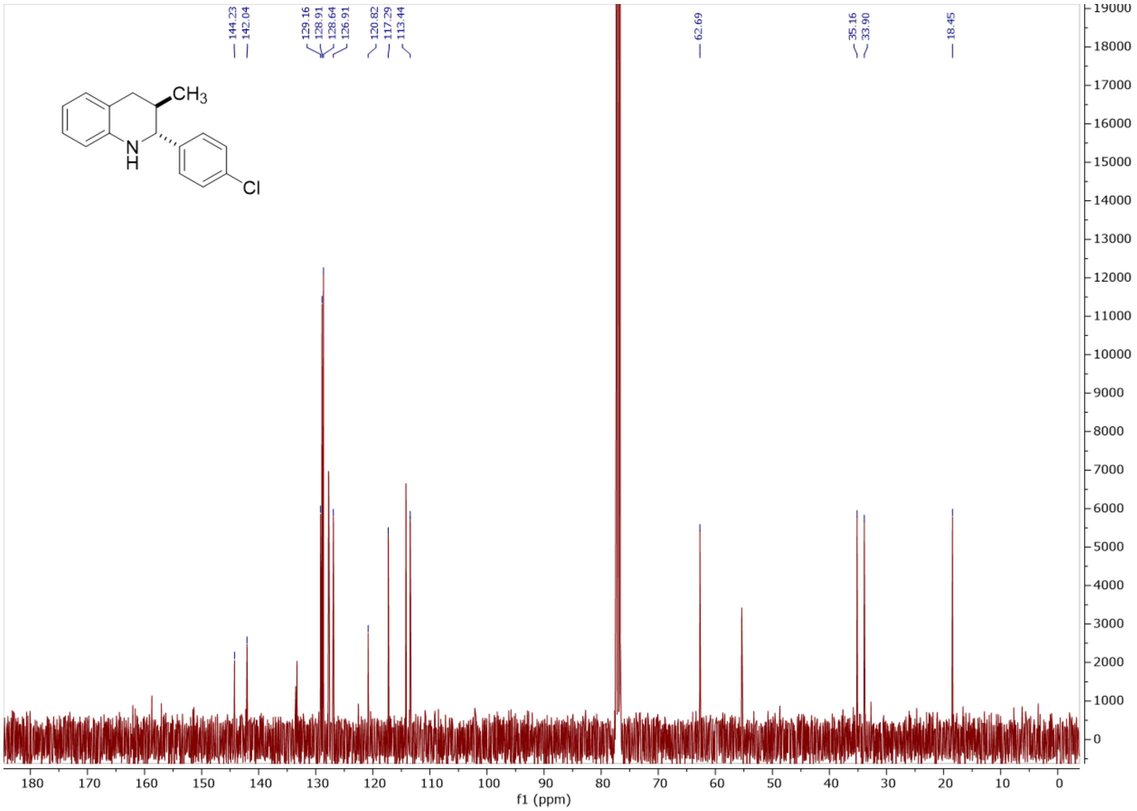

^{1}H NMR (300 MHz, $CDCl_3$) and ^{19}F NMR (282 MHz, $CDCl_3$) spectra for **2ai**

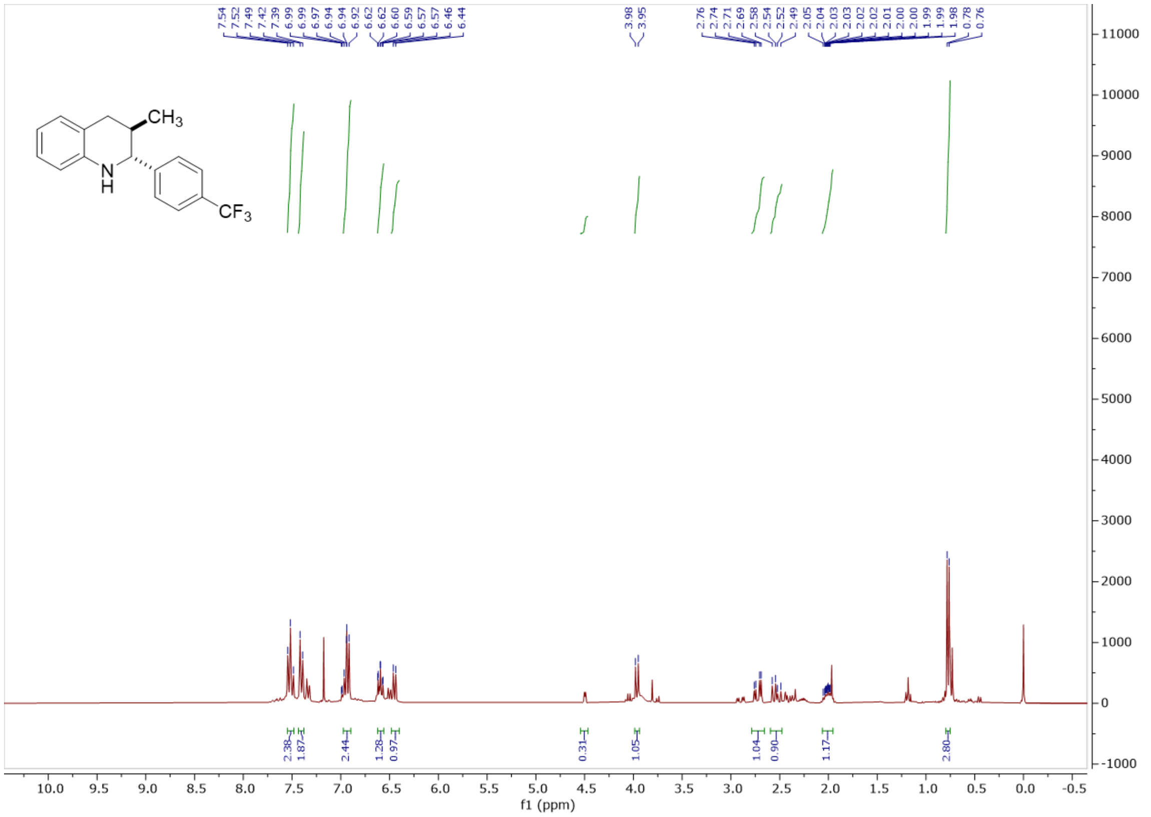

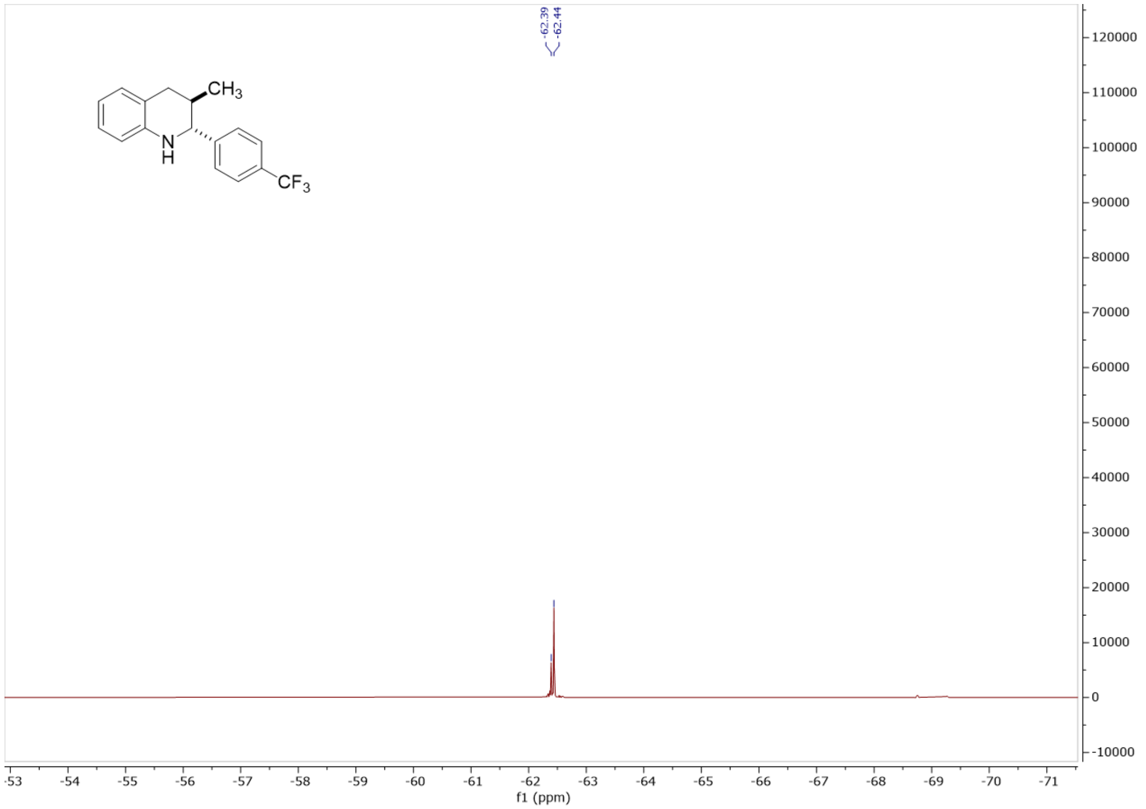

^{1}H NMR (400 MHz, $CDCl_3$) and ^{13}C NMR (101 MHz, $CDCl_3$) spectra for **2aj**

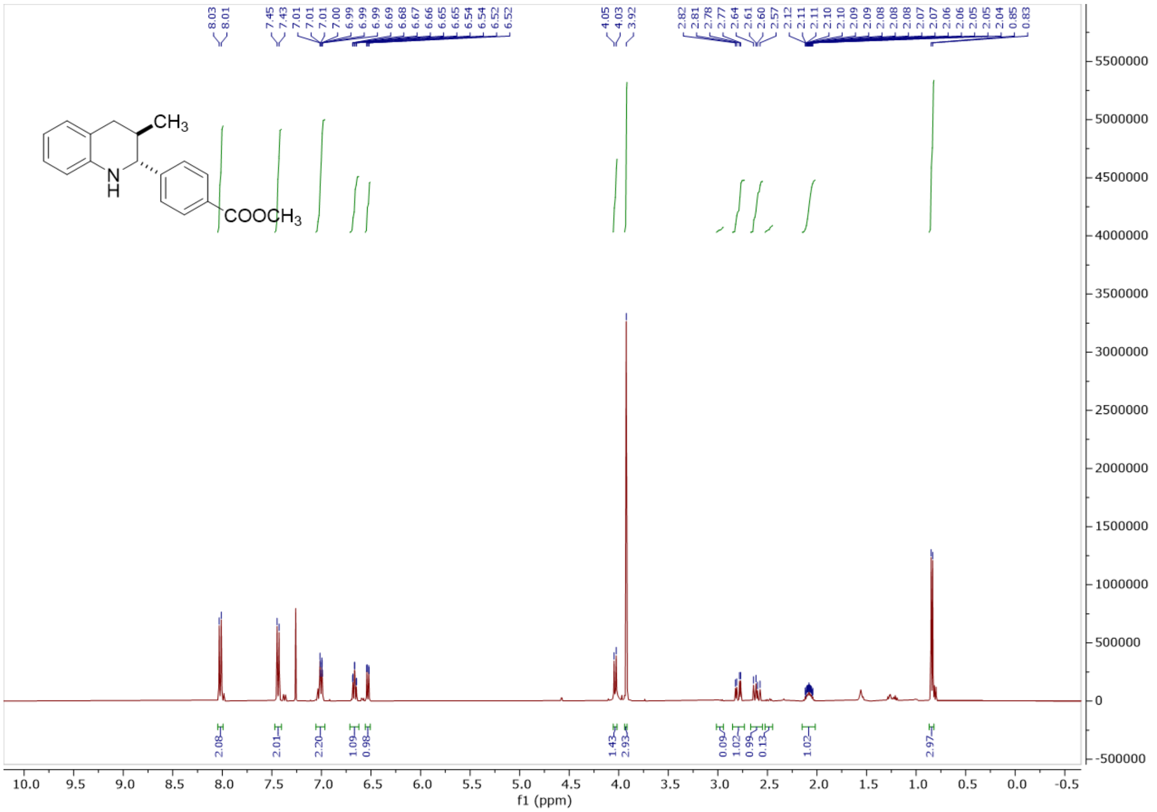

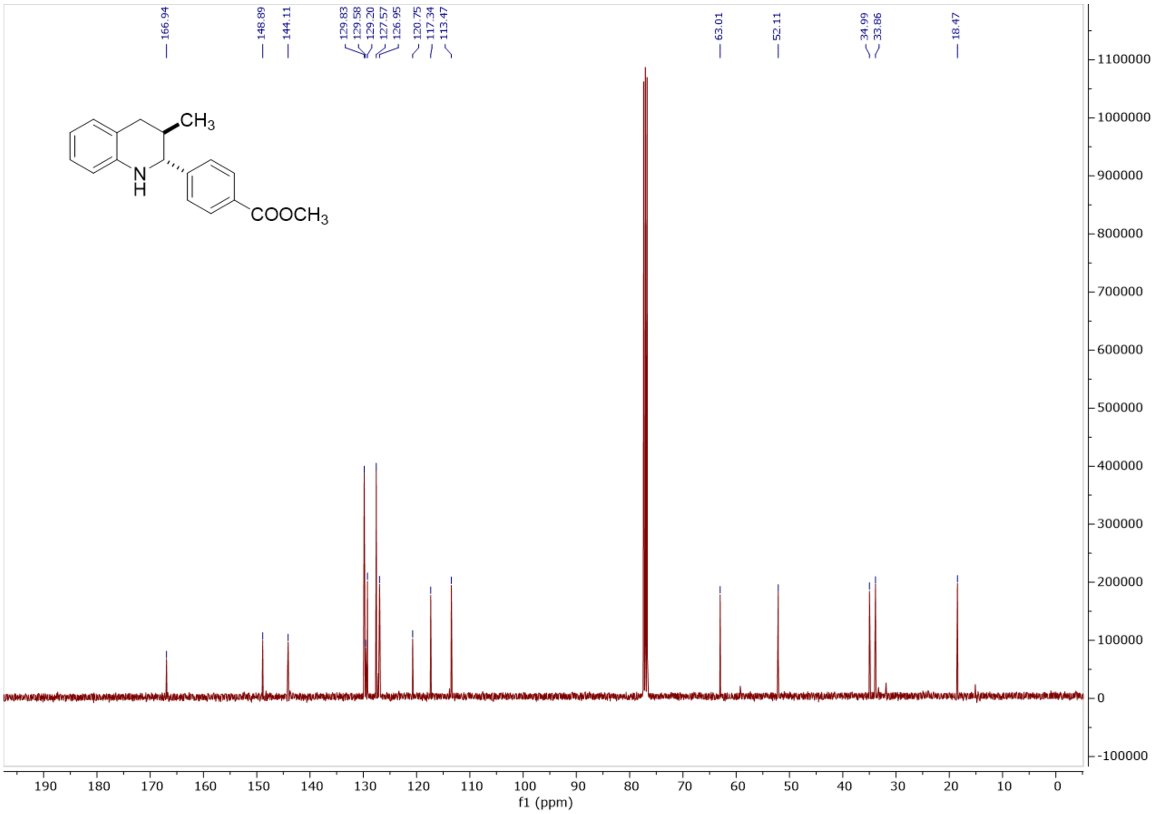

^{1}H NMR (300 MHz, $CDCl_3$) and ^{13}C NMR (76 MHz, $CDCl_3$) spectra for **2ak**

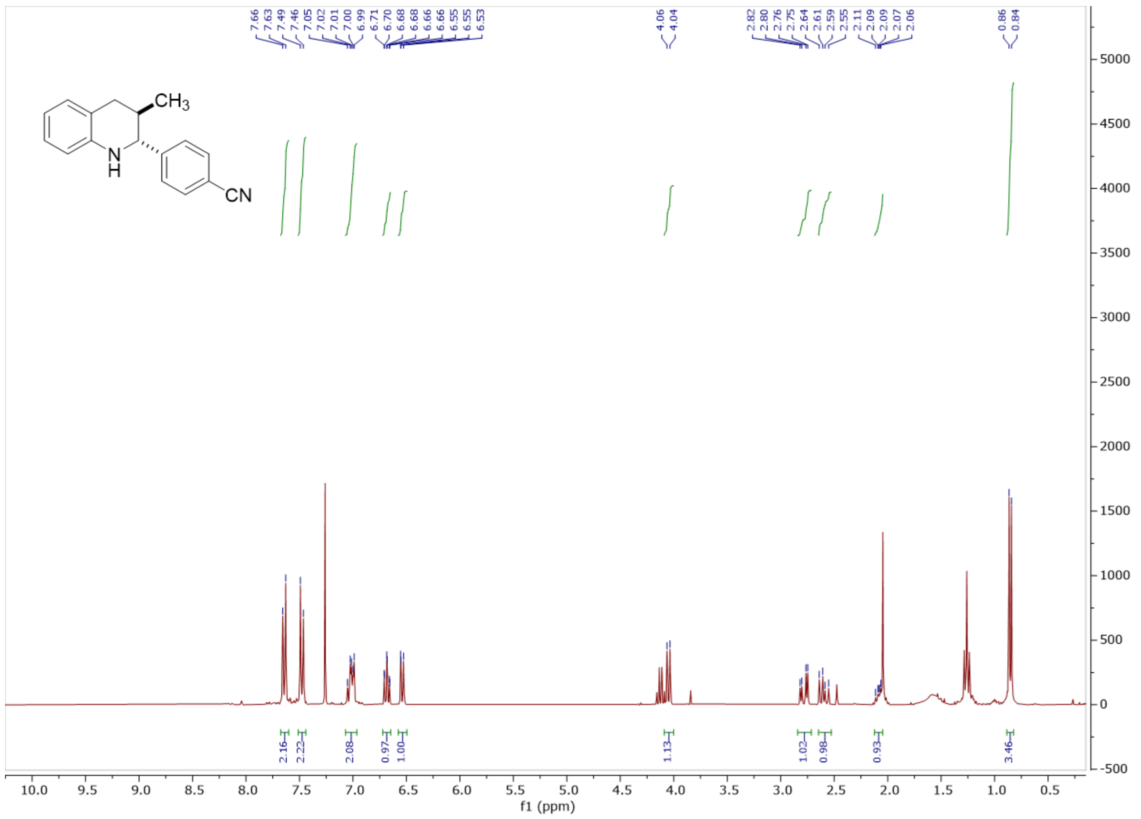

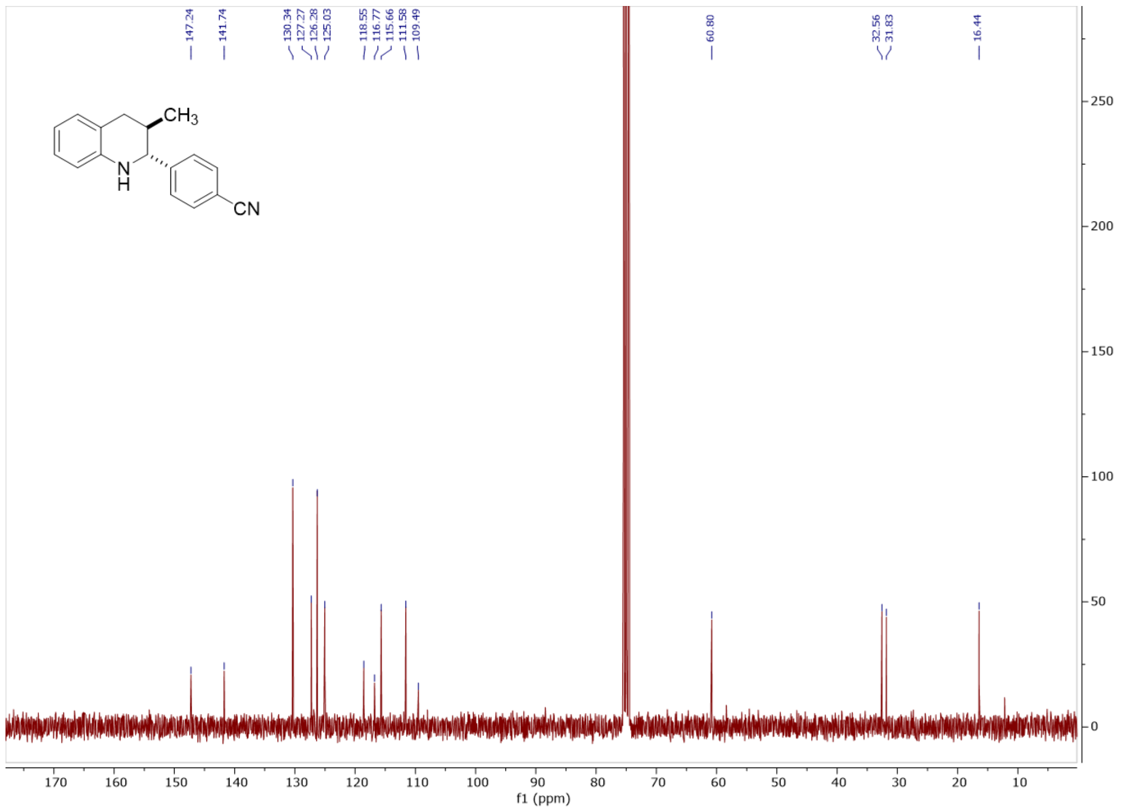

^{1}H NMR (300 MHz, $CDCl_3$) and ^{13}C NMR (76 MHz, $CDCl_3$) spectra for **2al**

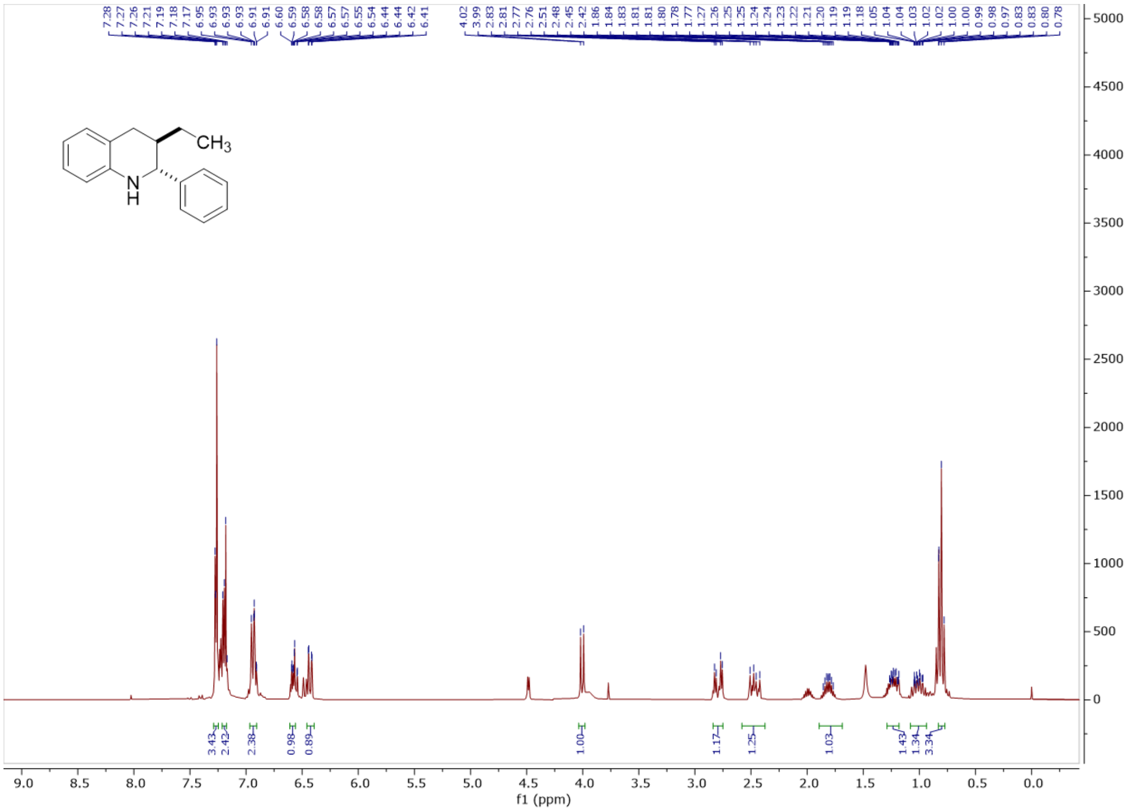

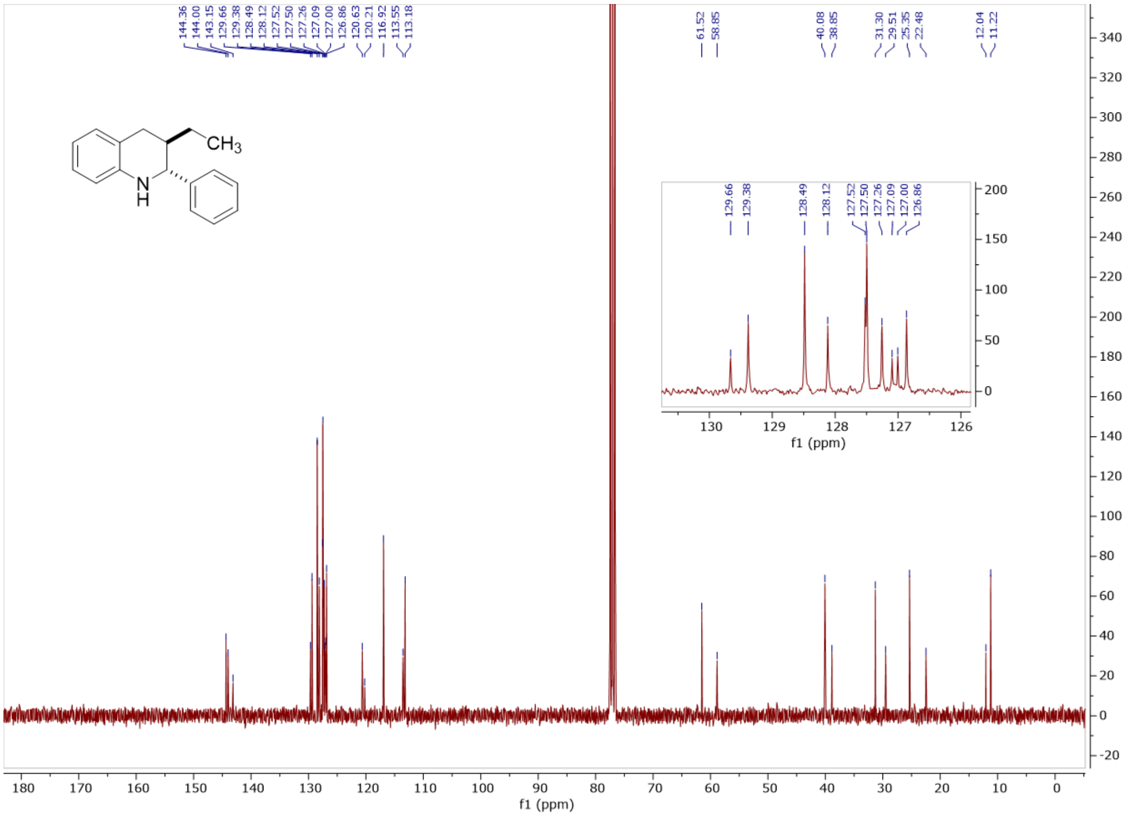

^{1}H NMR (300 MHz, $CDCl_3$) and ^{13}C NMR (76 MHz, $CDCl_3$) spectra for *trans*-**2am**

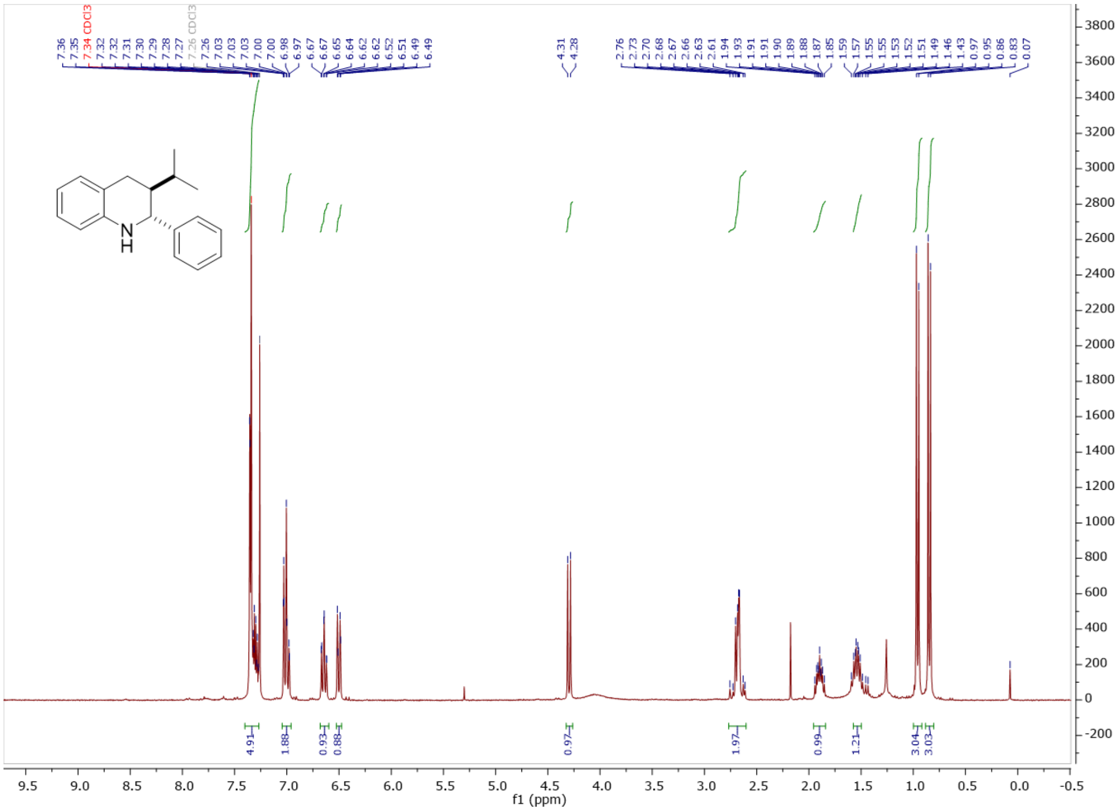

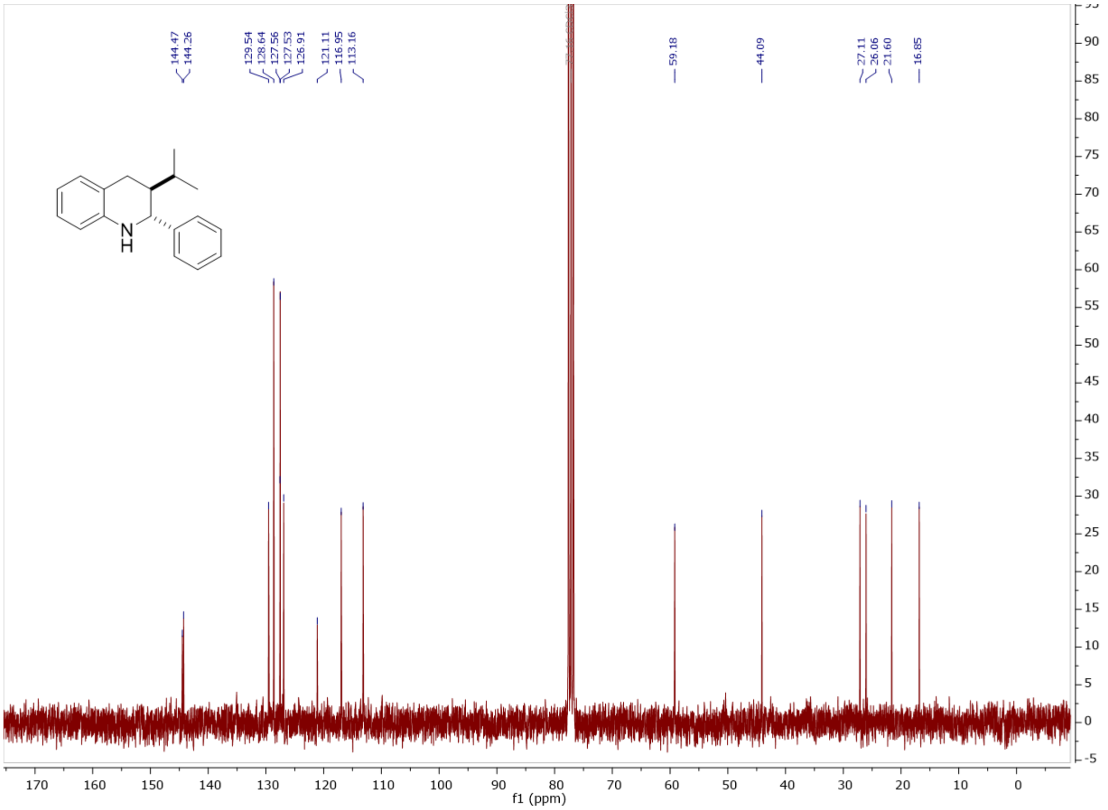

^{1}H NMR (300 MHz, $CDCl_3$) and ^{13}C NMR (76 MHz, $CDCl_3$) spectra for *cis*-**2am**

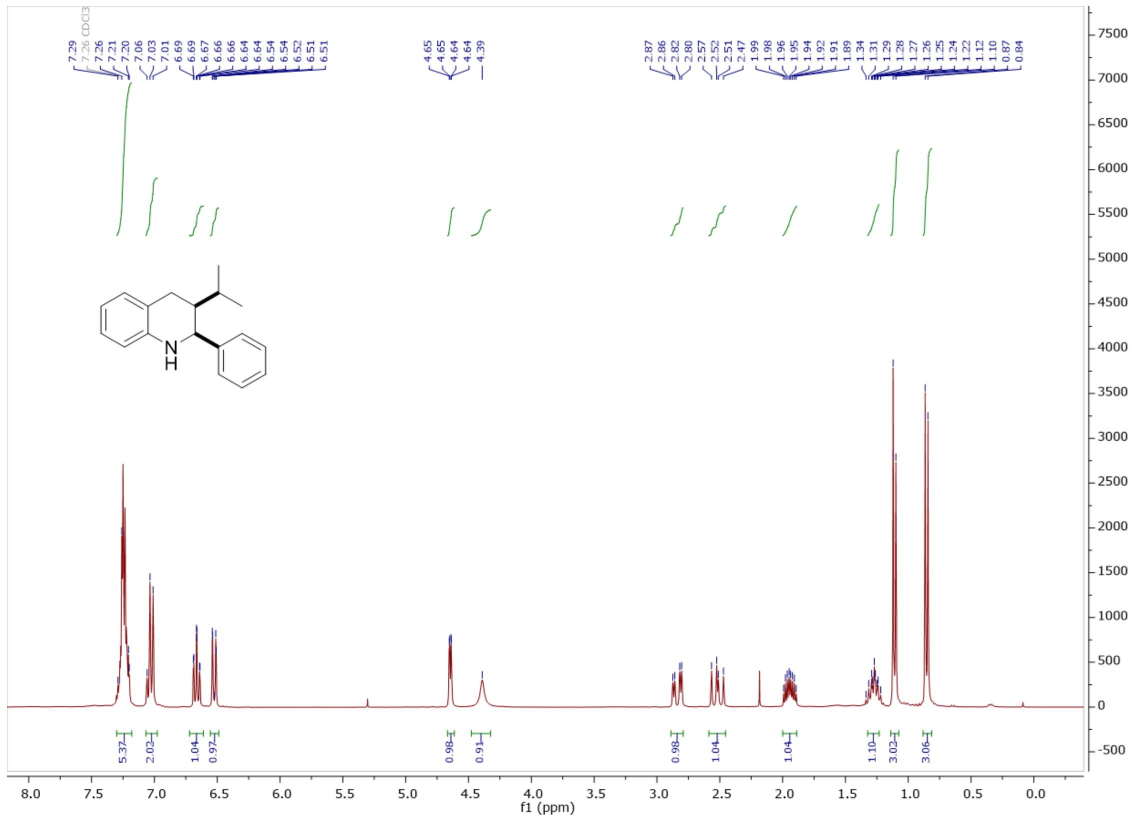

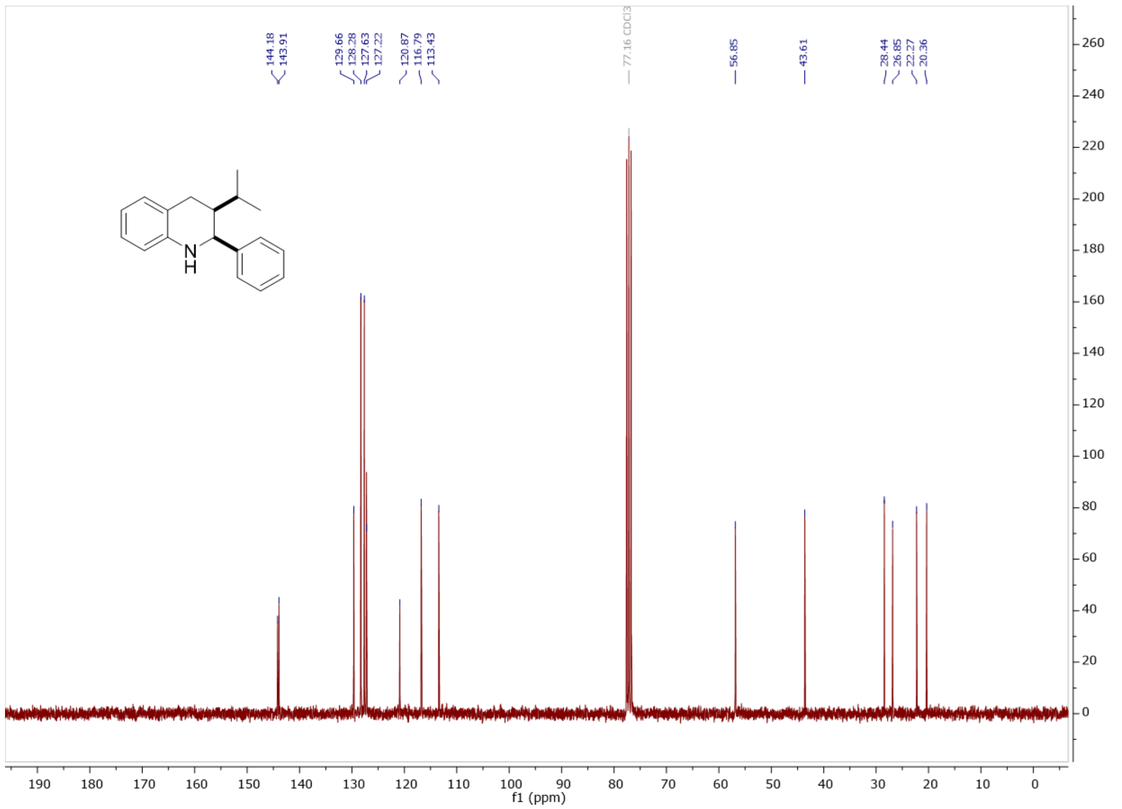

^1H NMR (400 MHz, $CDCl_3$) and ^{13}C NMR (101 MHz, $CDCl_3$) spectra for **2an**

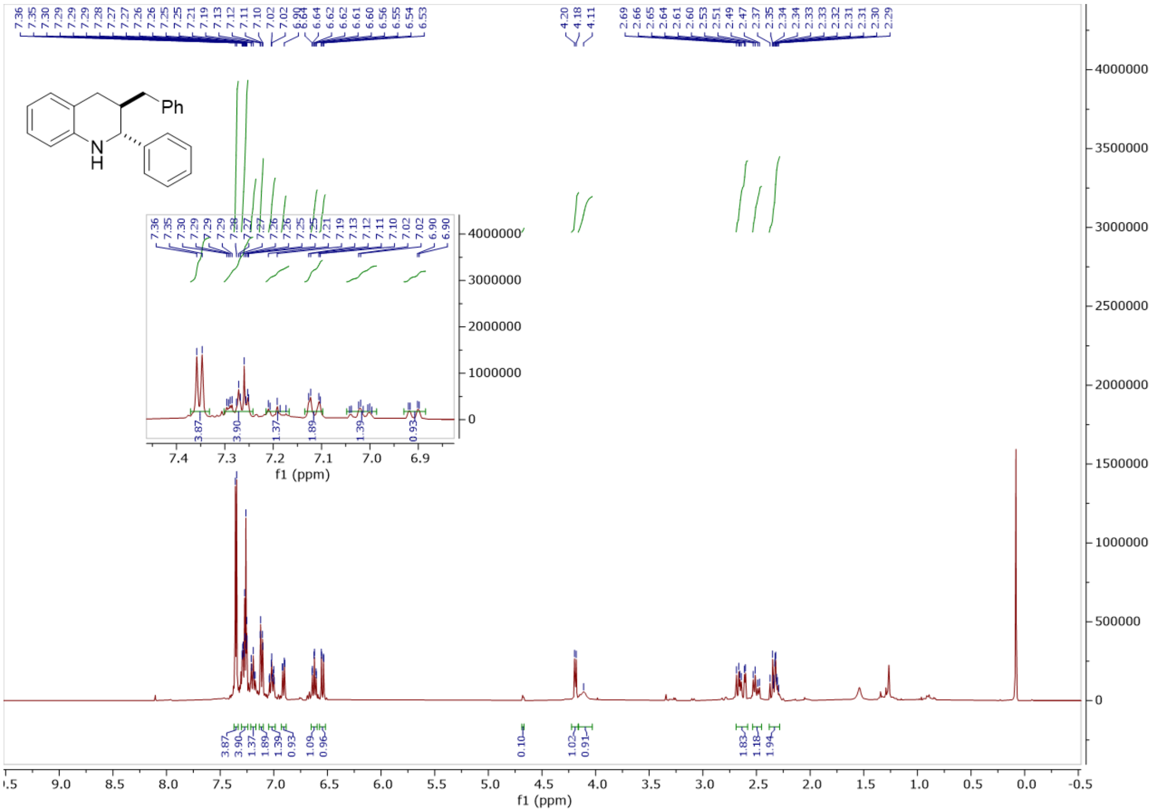

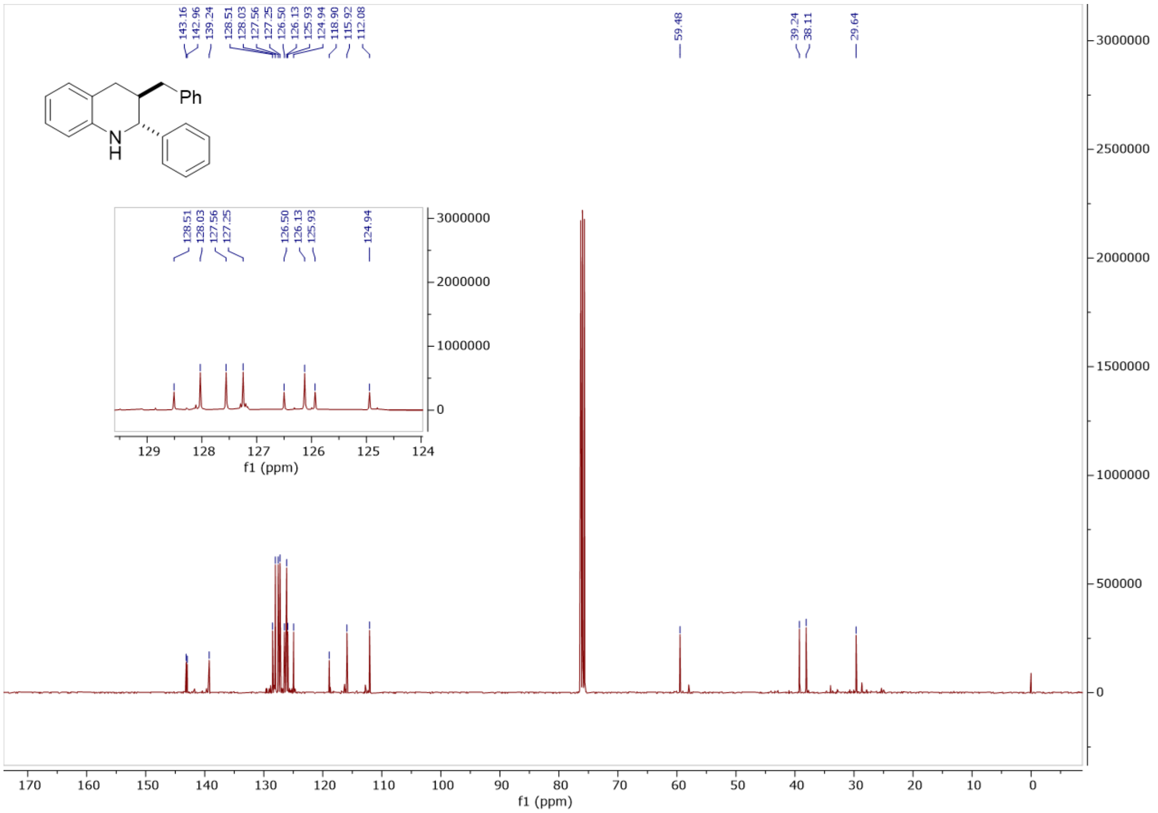

^{1}H NMR (300 MHz, $CDCl_3$) and ^{13}C NMR (76 MHz, $CDCl_3$) spectra for **2ao**

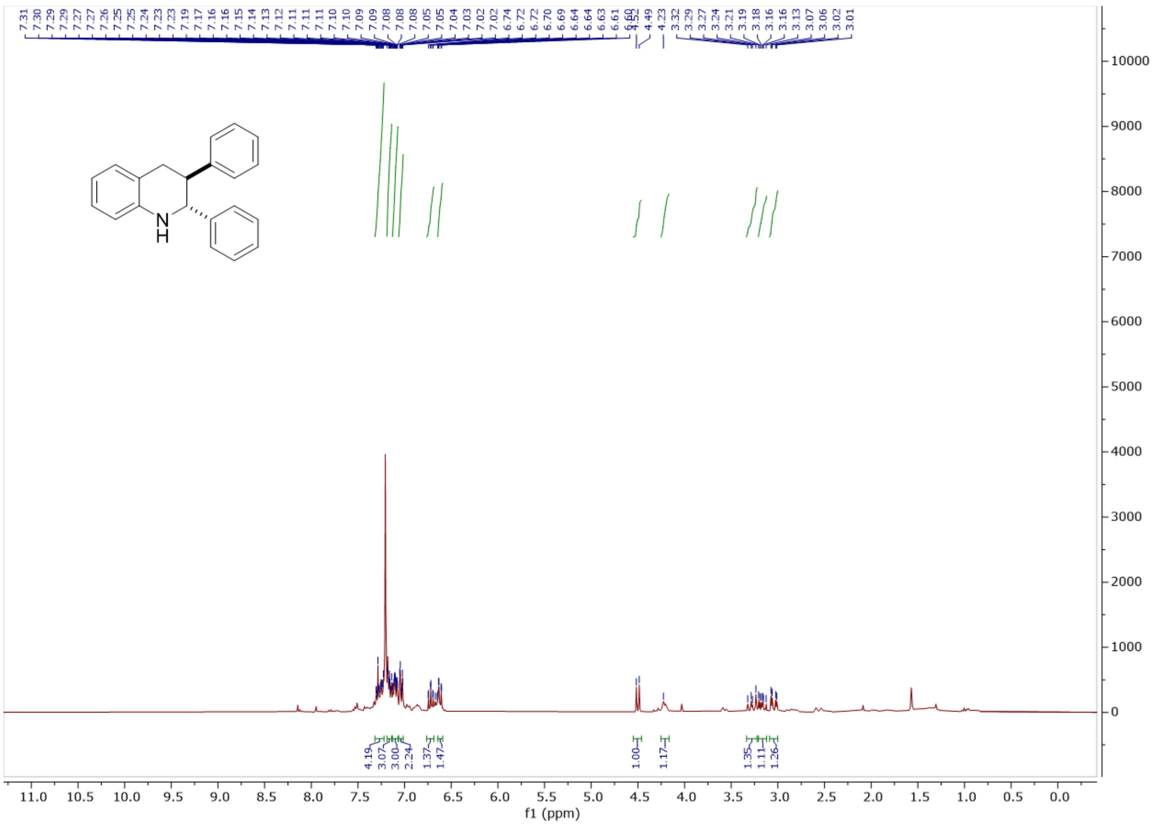

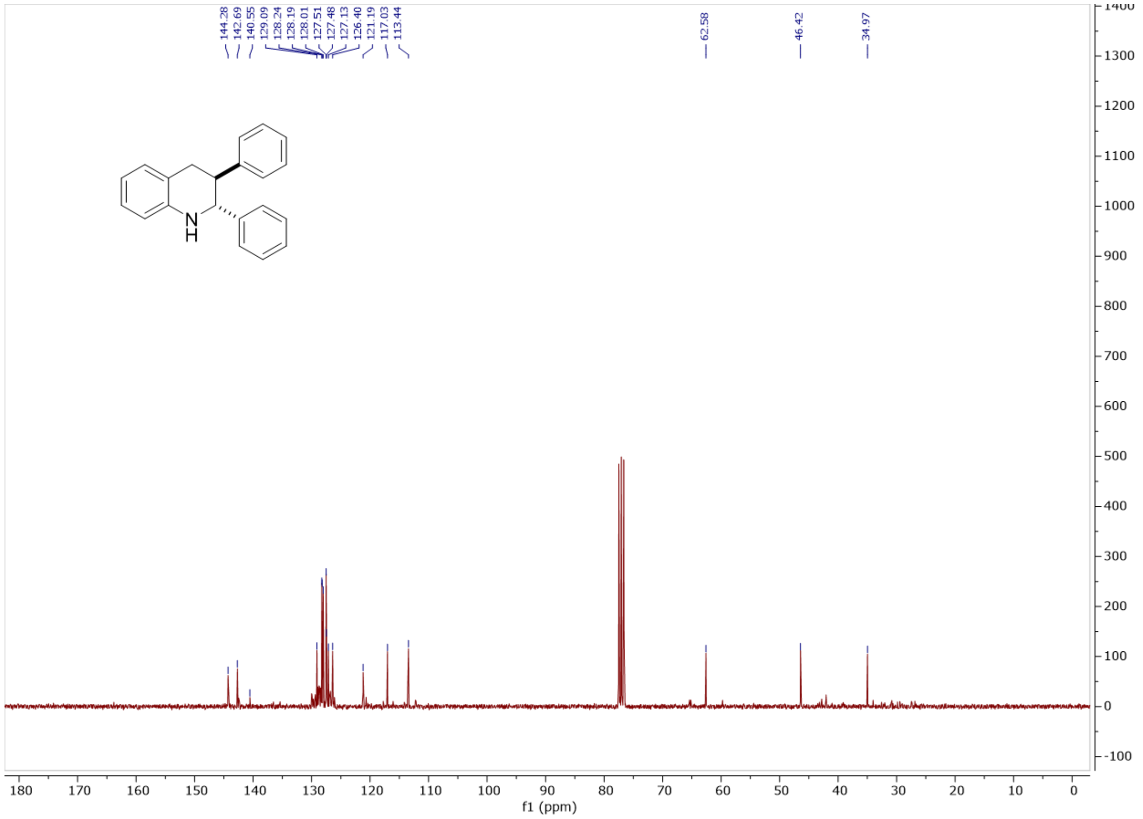

^{1}H NMR (300 MHz, $CDCl_3$) and ^{13}C NMR (76 MHz, $CDCl_3$) spectra for **2ap**

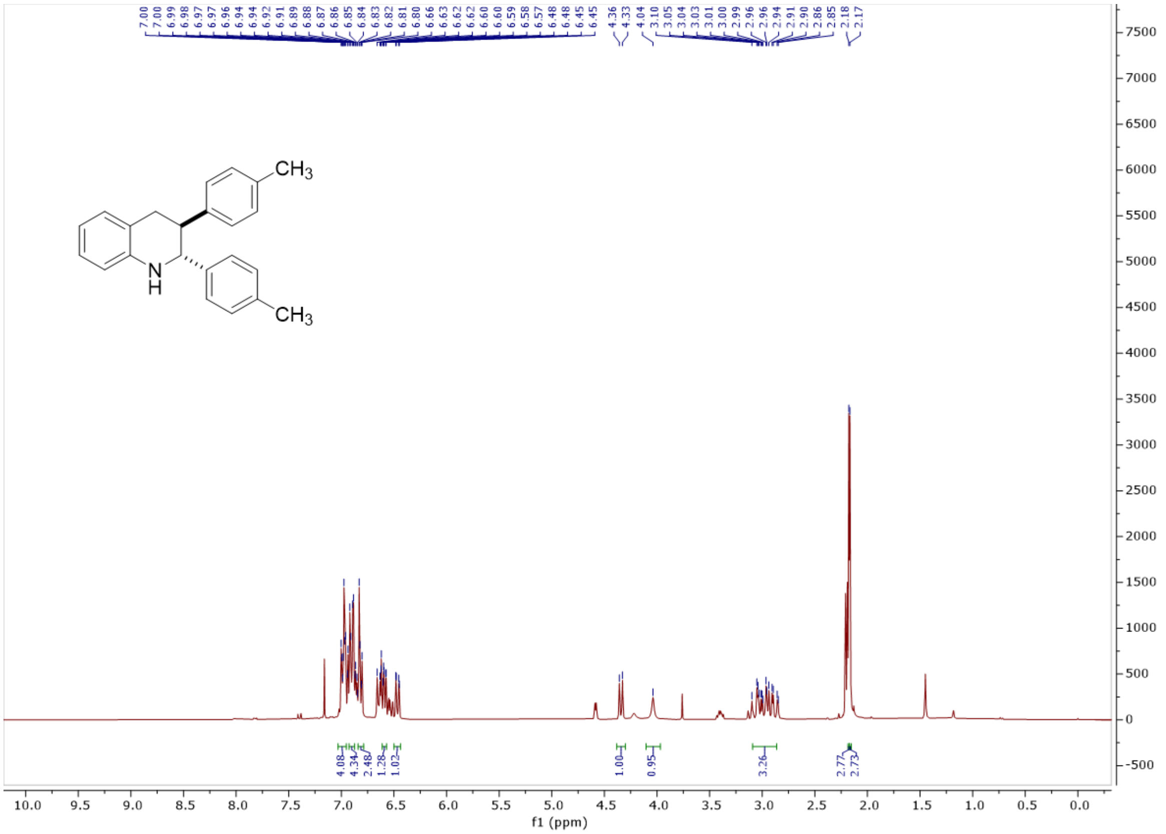

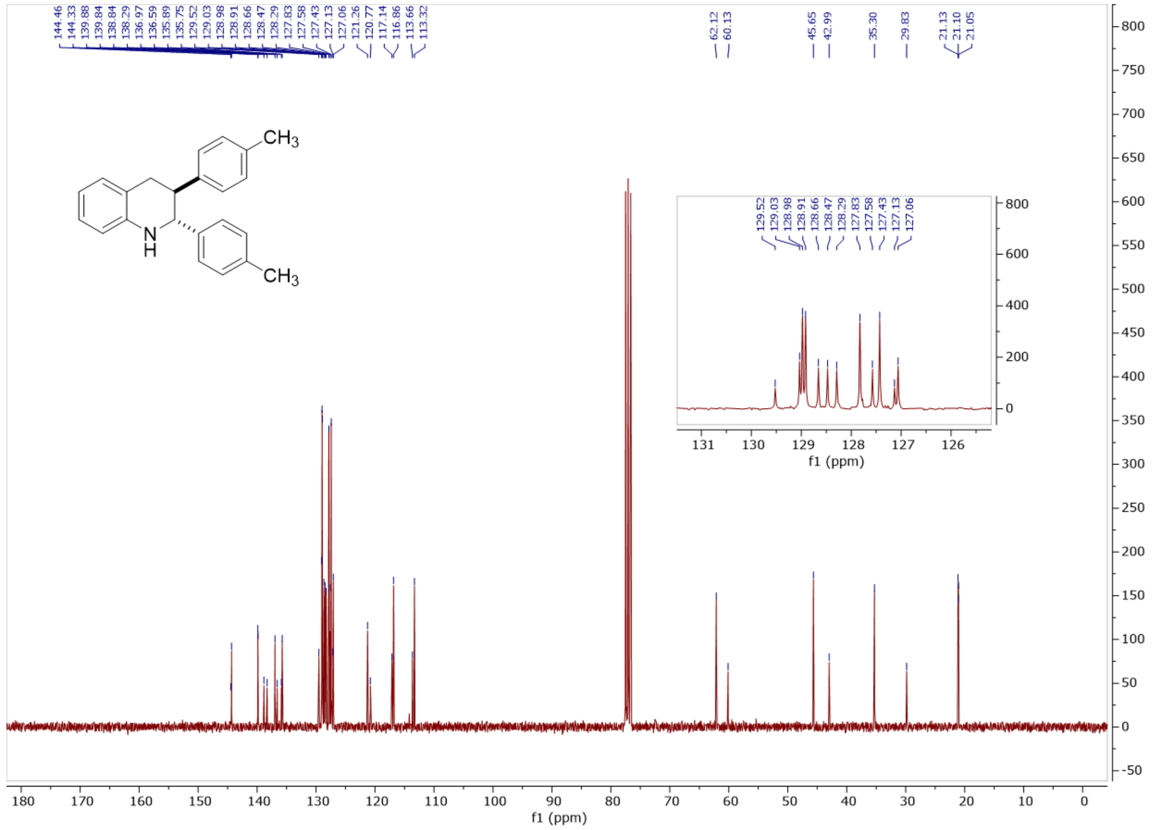

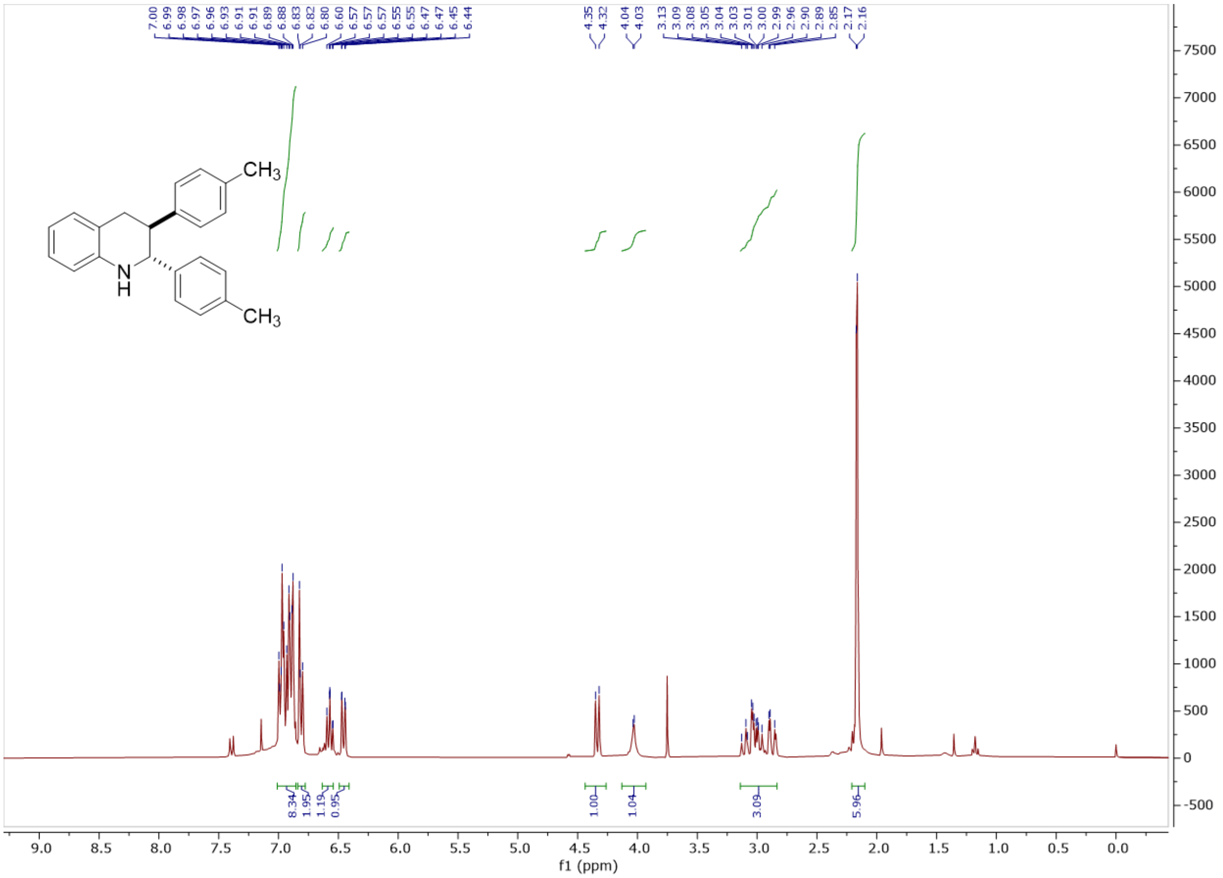

^{1}H NMR (599 MHz, $CDCl_3$), ^{19}F NMR (282 MHz, $CDCl_3$) and ^{13}C NMR (151 MHz, $CDCl_3$) spectra for **2aq**

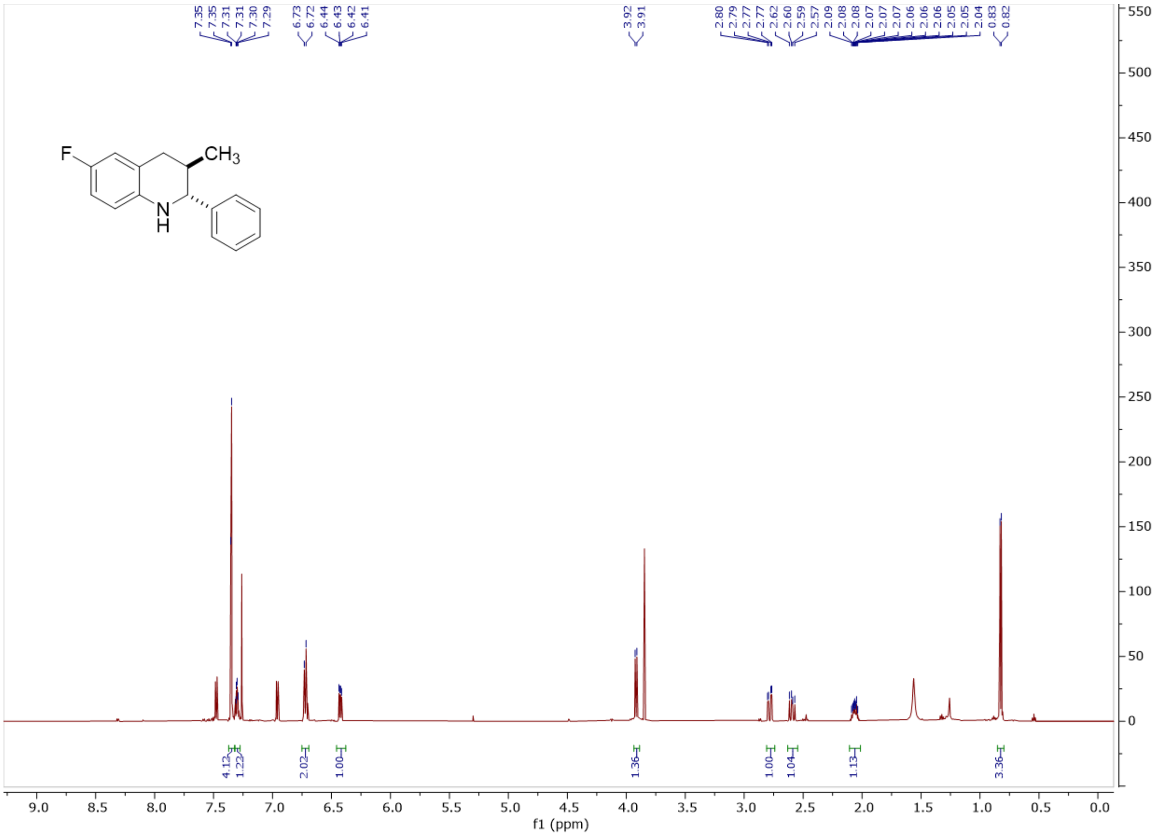

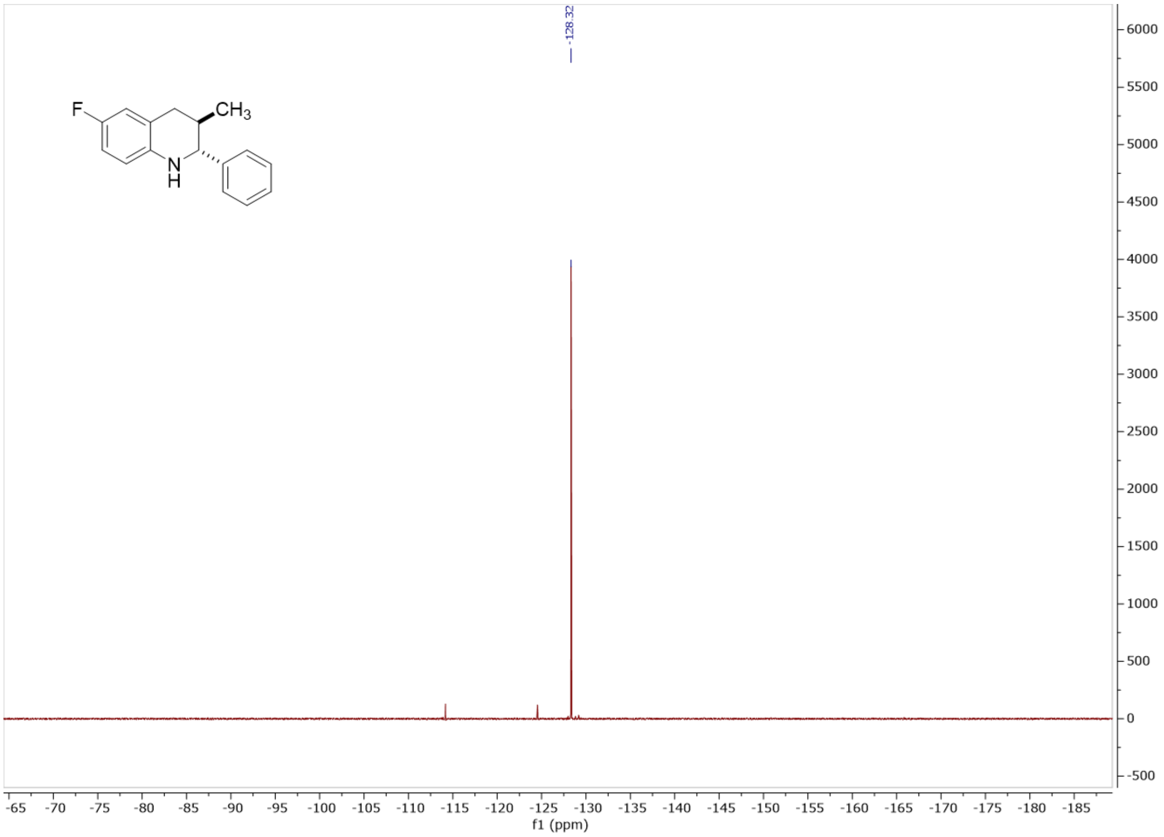

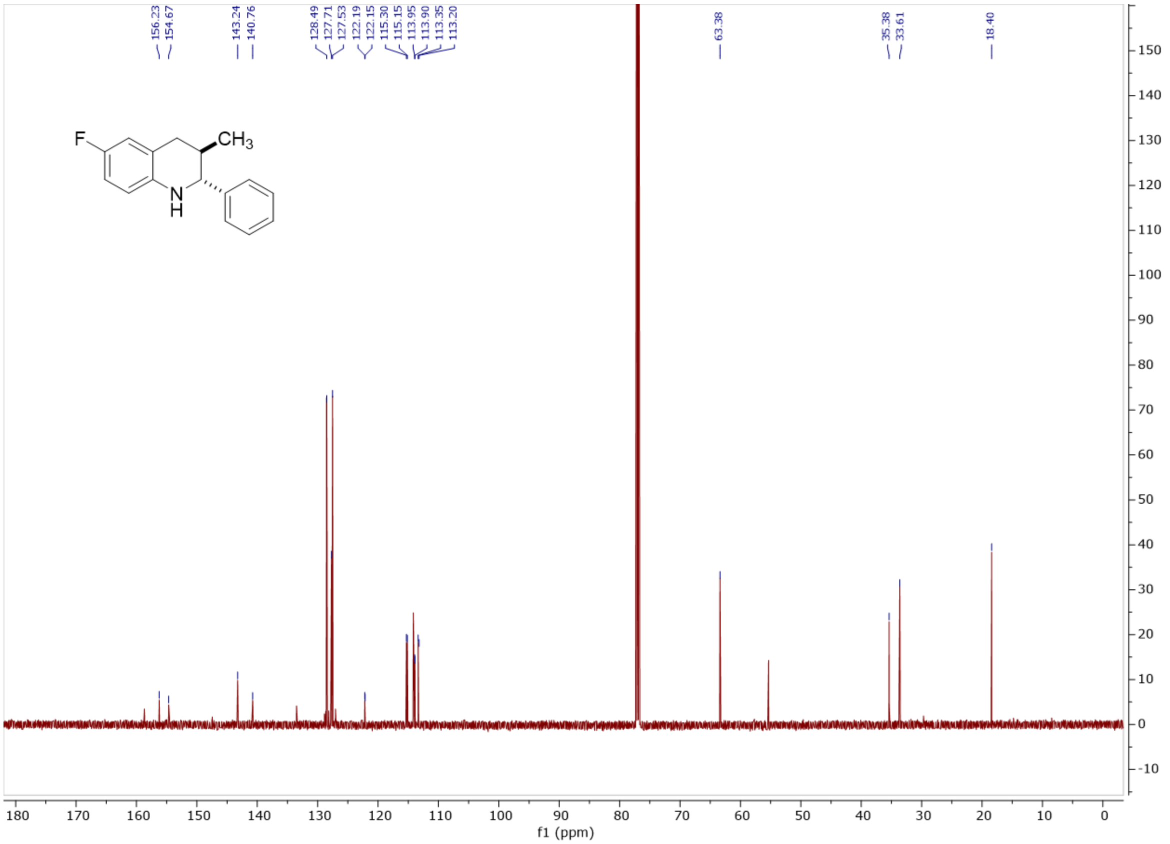
156.23
154.67
143.24
140.76
128.49
127.71
127.53
122.19
122.15
115.30
115.15
113.95
113.90
113.35
113.20
63.38
35.38
33.61
18.40
F
CH3
N
H
f1 (ppm)

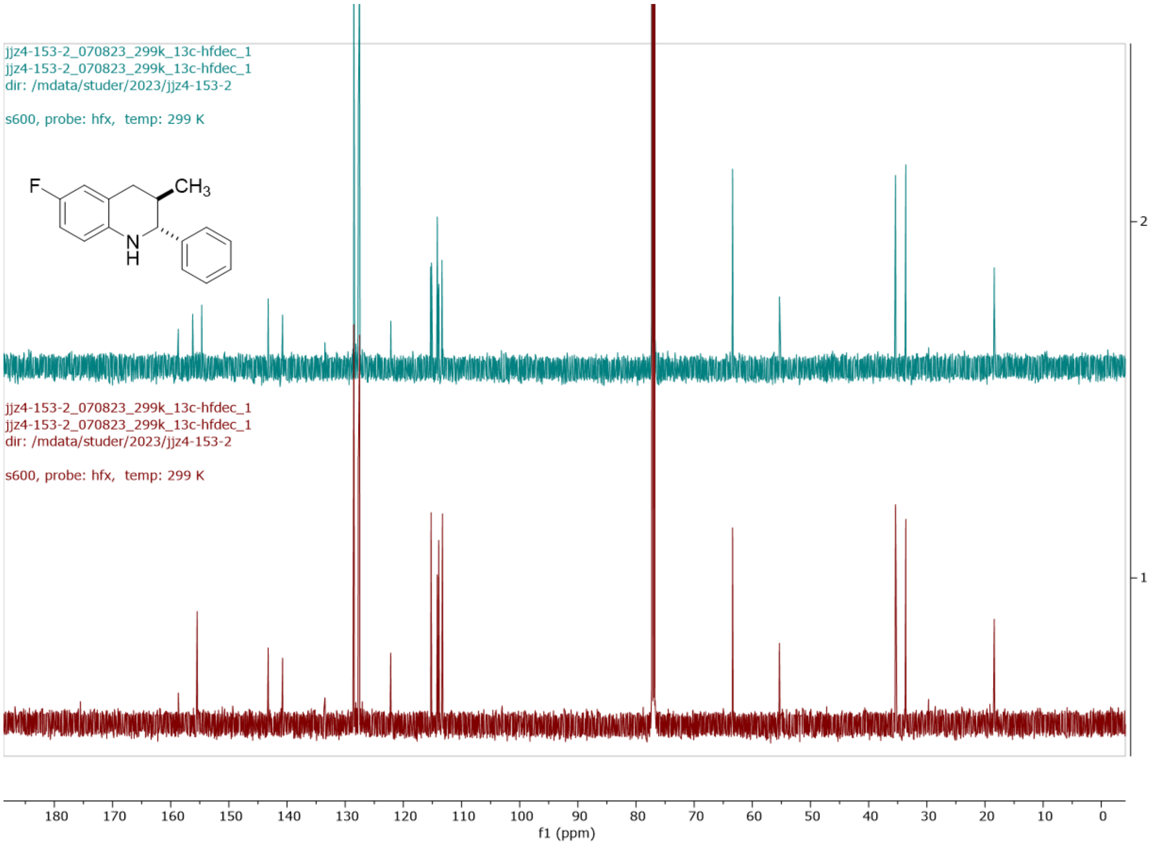
jjz4-153-2_070823_299k_13c-hfdec_1
jjz4-153-2_070823_299k_13c-hfdec_1
dir: /mdata/studer/2023/jjz4-153-2
s600, probe: hfx, temp: 299 K
F
CH3
N
H
jjz4-153-2_070823_299k_13c-hfdec_1
jjz4-153-2_070823_299k_13c-hfdec_1
dir: /mdata/studer/2023/jjz4-153-2
s600, probe: hfx, temp: 299 K
f1 (ppm)

^{1}H NMR (599 MHz, $CDCl_3$) and ^{13}C NMR (151 MHz, $CDCl_3$) spectra for **2ar**

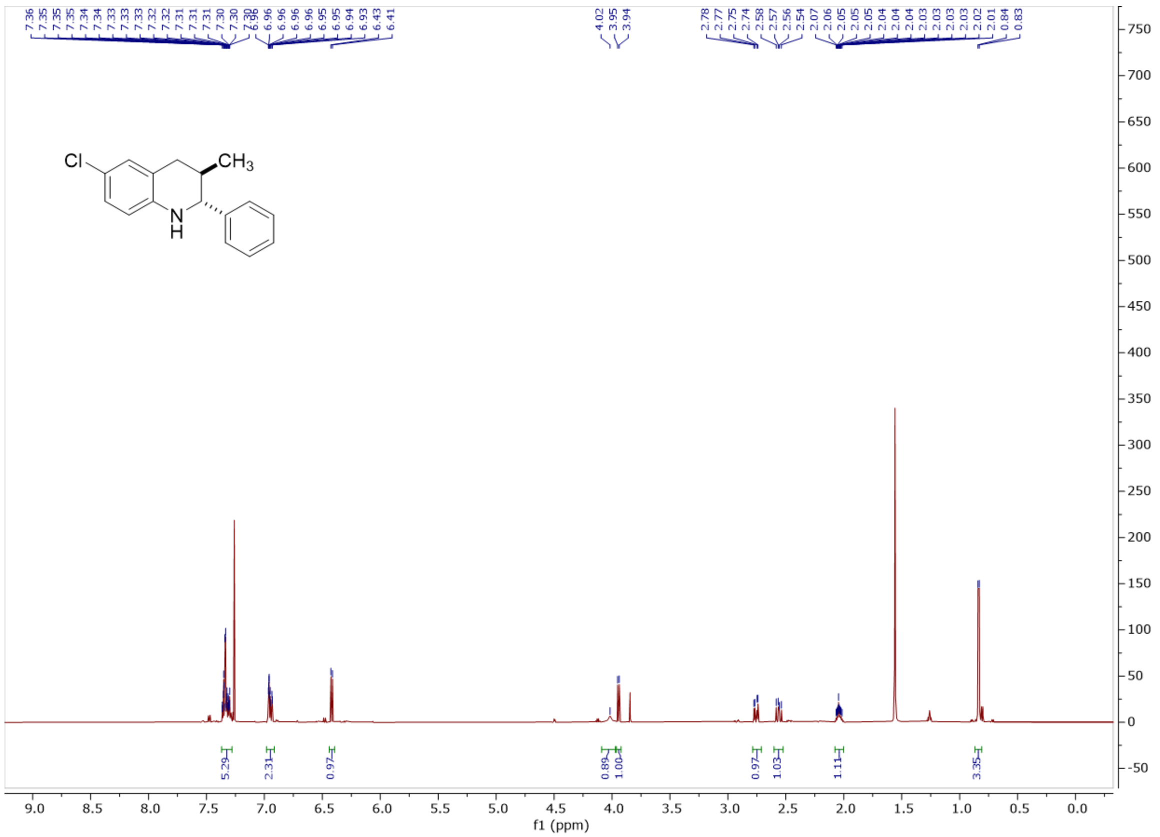

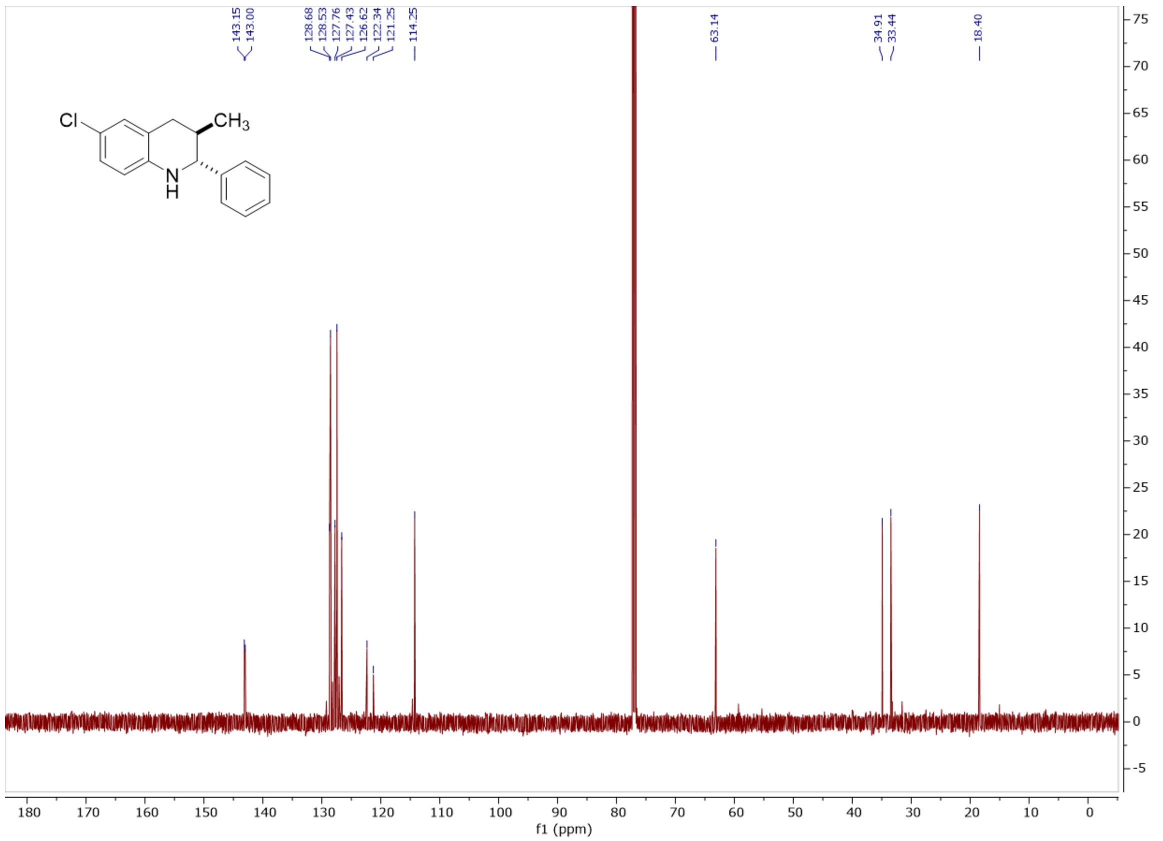

^{1}H NMR (300 MHz, $CDCl_3$) and ^{13}C NMR (76 MHz, $CDCl_3$) spectra for **2as**

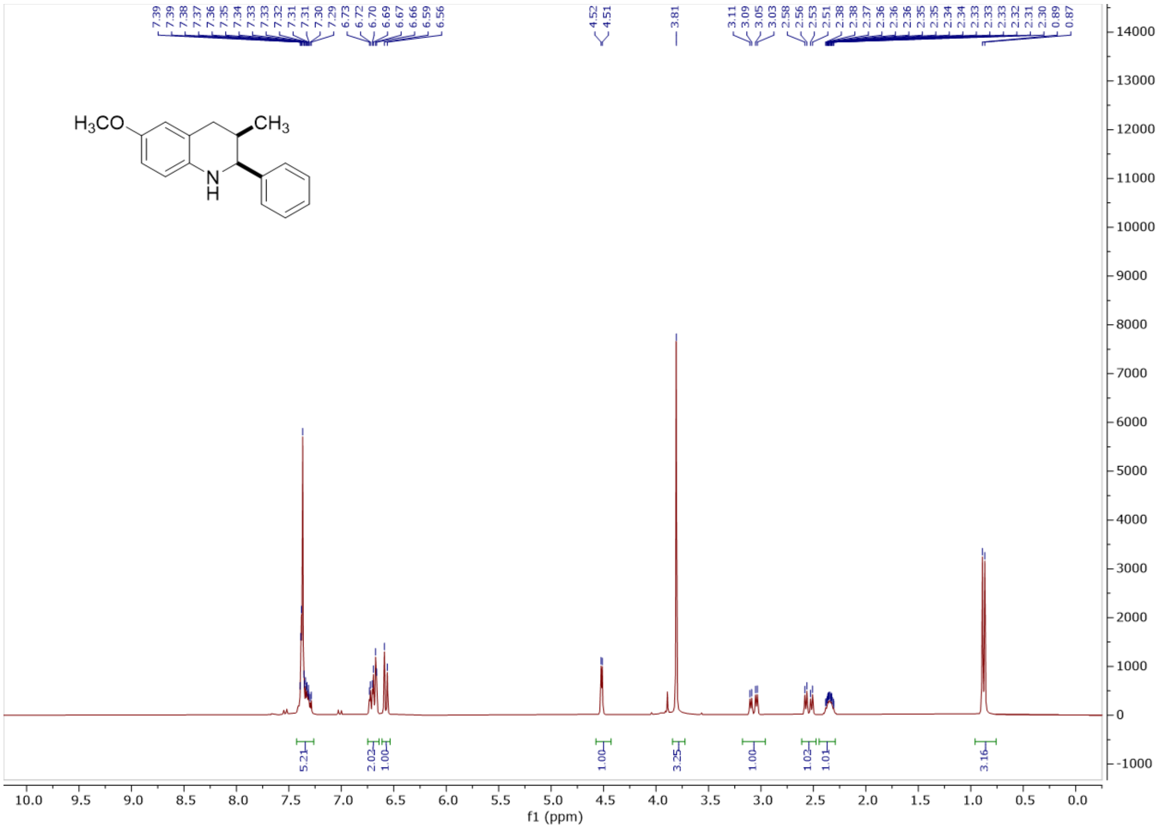

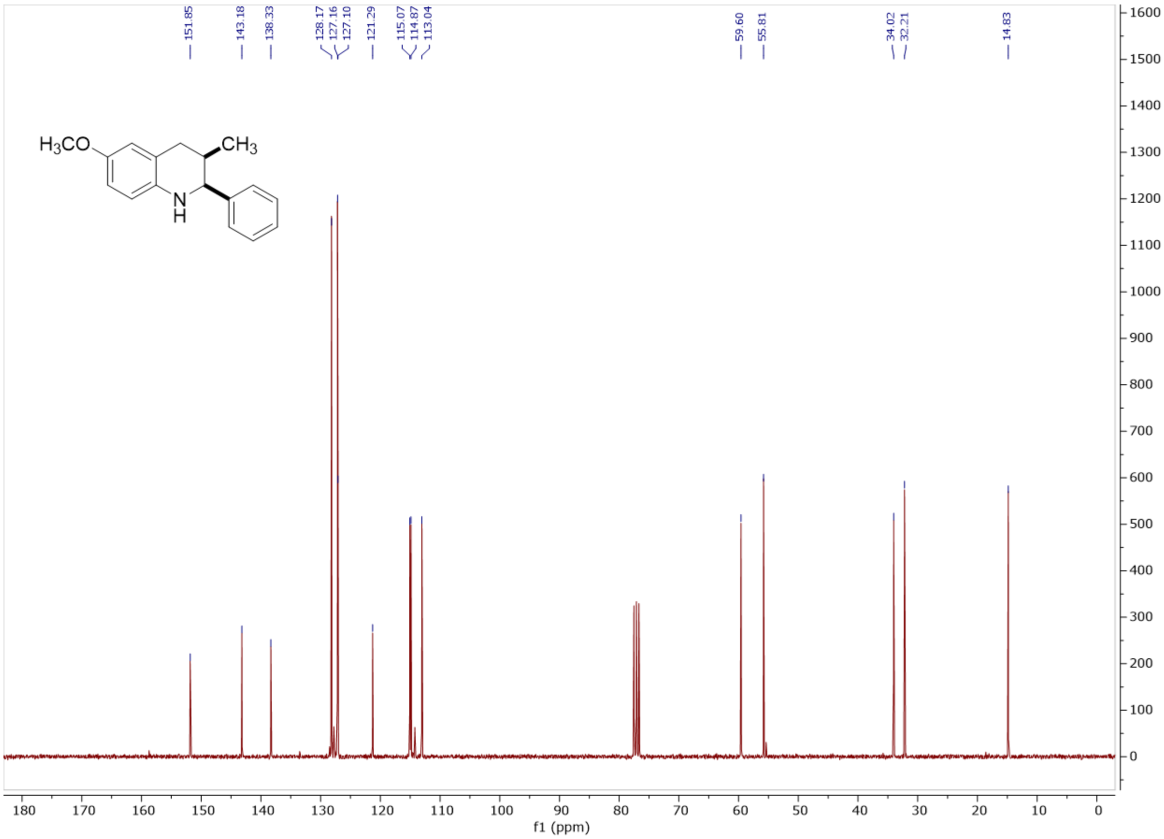

^{1}H NMR (300 MHz, $CDCl_3$) and ^{13}C NMR (76 MHz, $CDCl_3$) spectra for **2at**

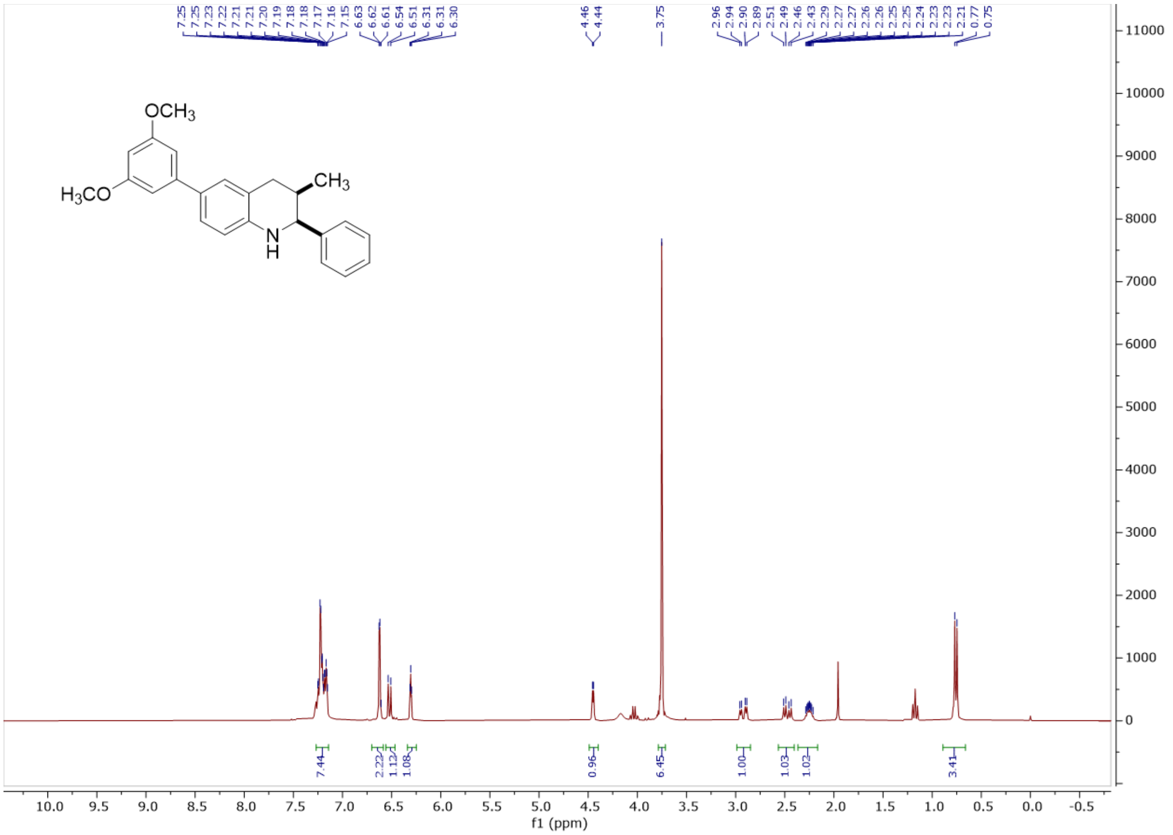

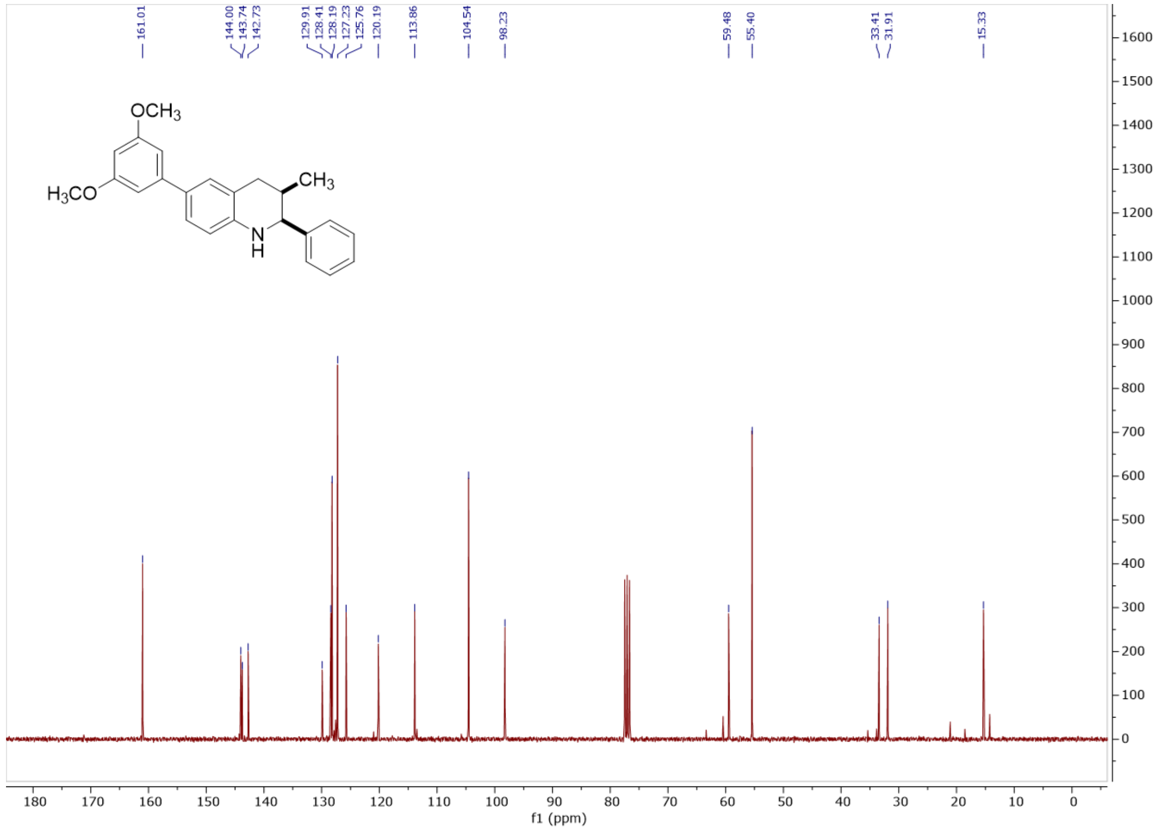

^{1}H NMR (400 MHz, $CDCl_3$) and ^{13}C NMR (101 MHz, $CDCl_3$) spectra for **2b'**

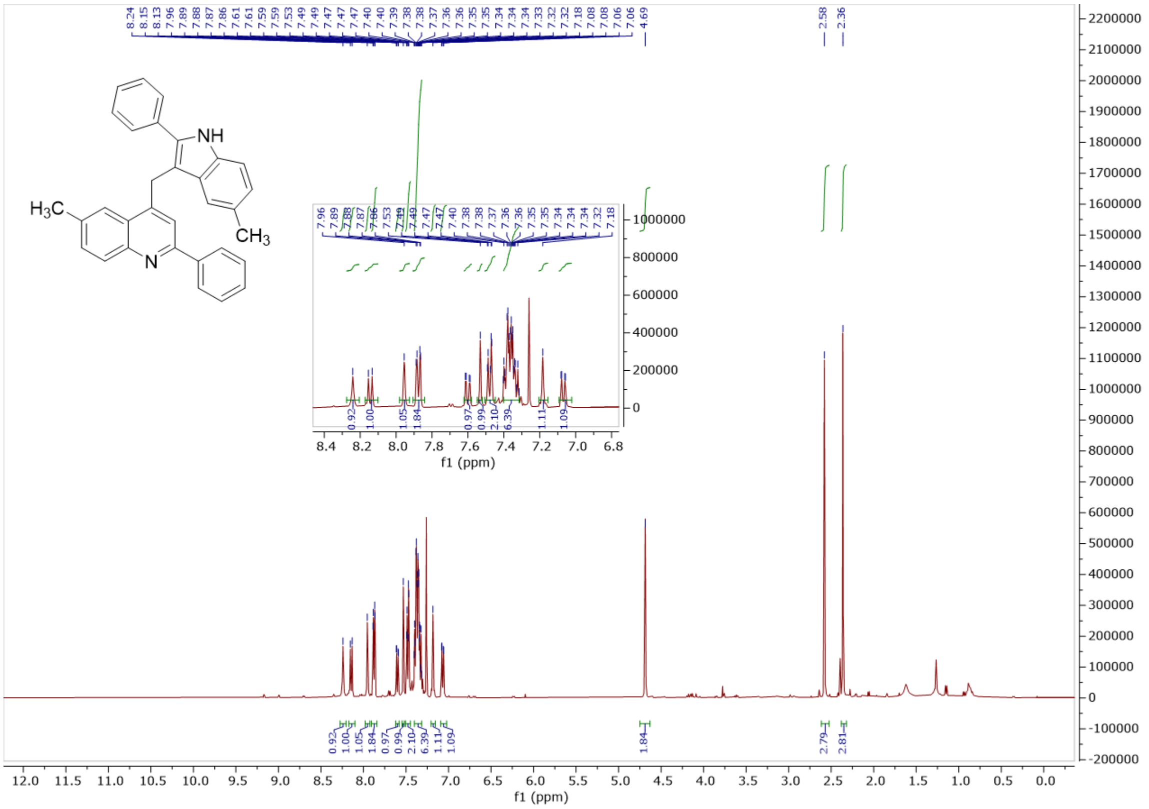

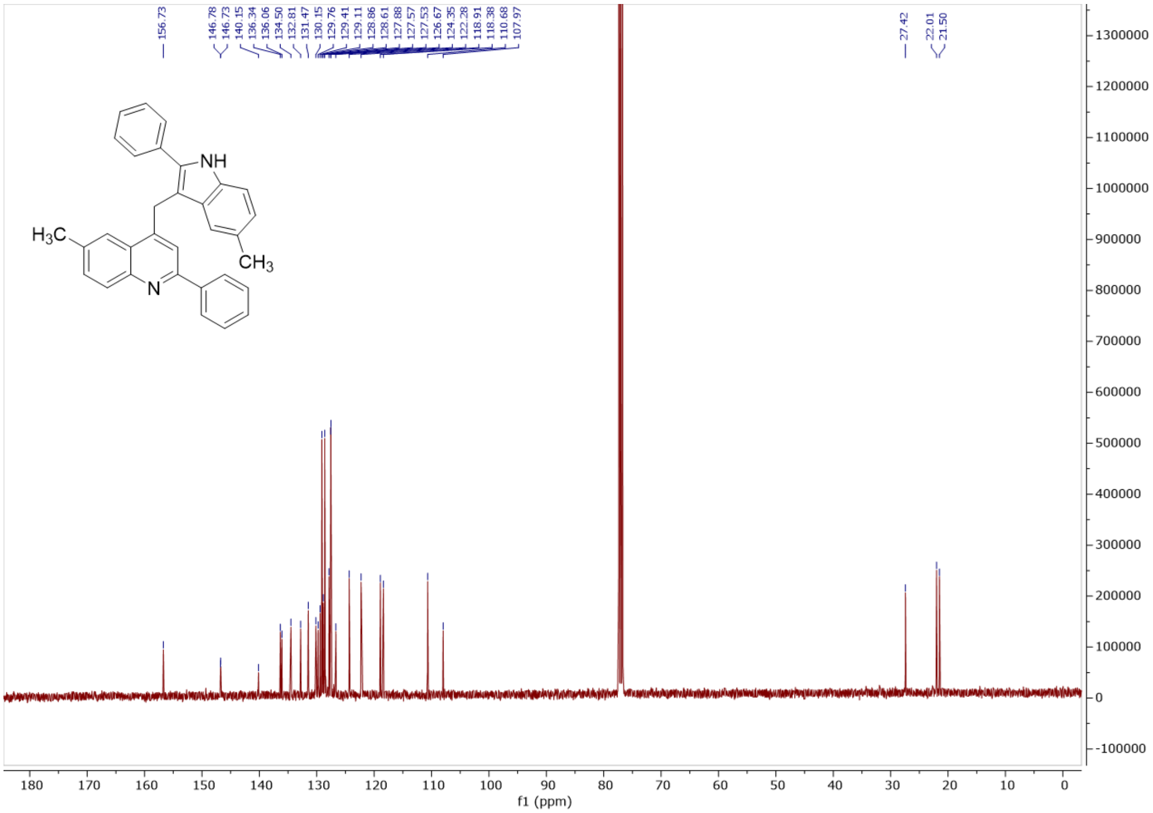

^{1}H NMR (599 MHz, $CDCl_3$) and ^{13}C NMR (151 MHz, $CDCl_3$) spectra for **2c'**

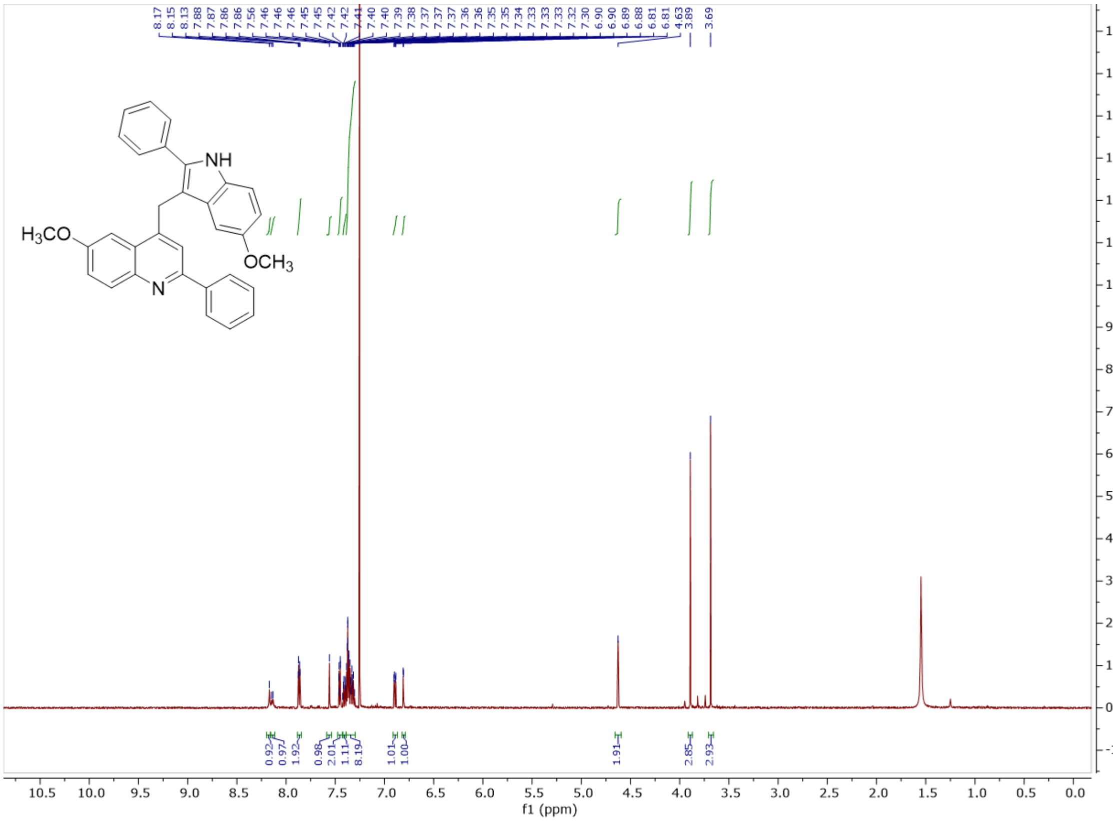

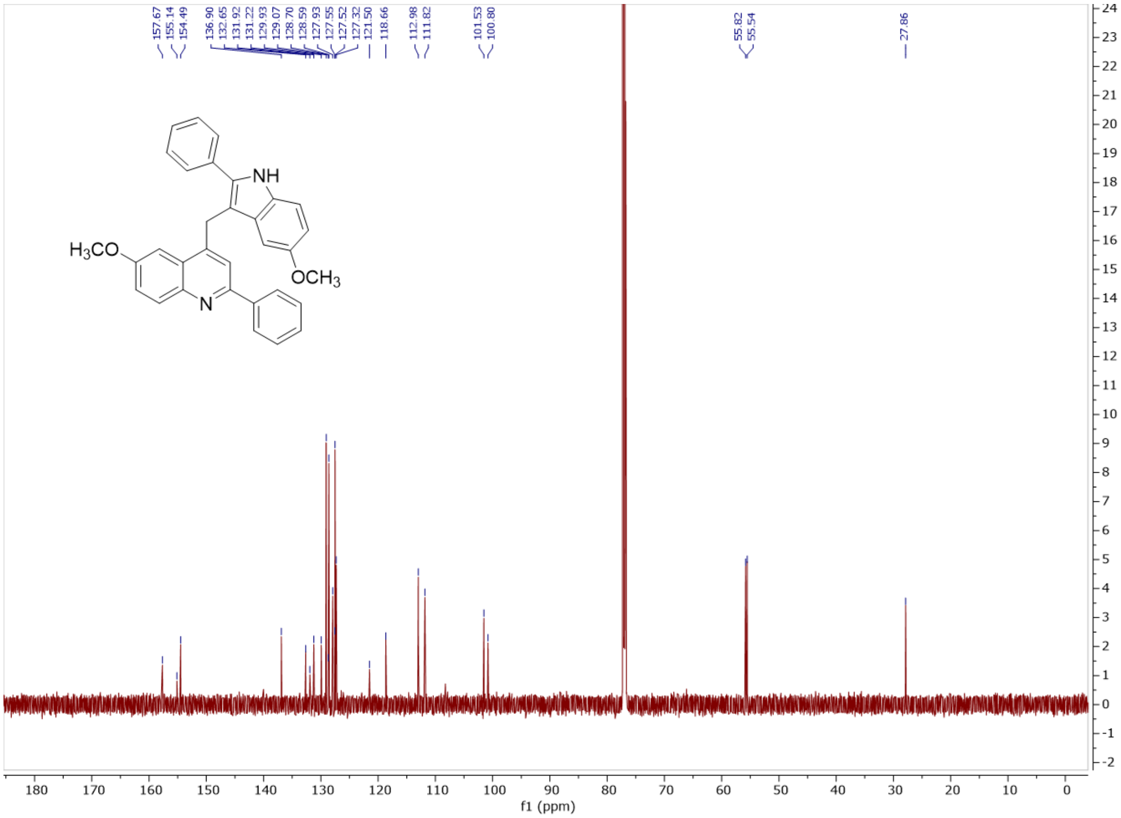

^{1}H NMR (599 MHz, $CDCl_3$) and ^{13}C NMR (151 MHz, $CDCl_3$) spectra for **2d'**

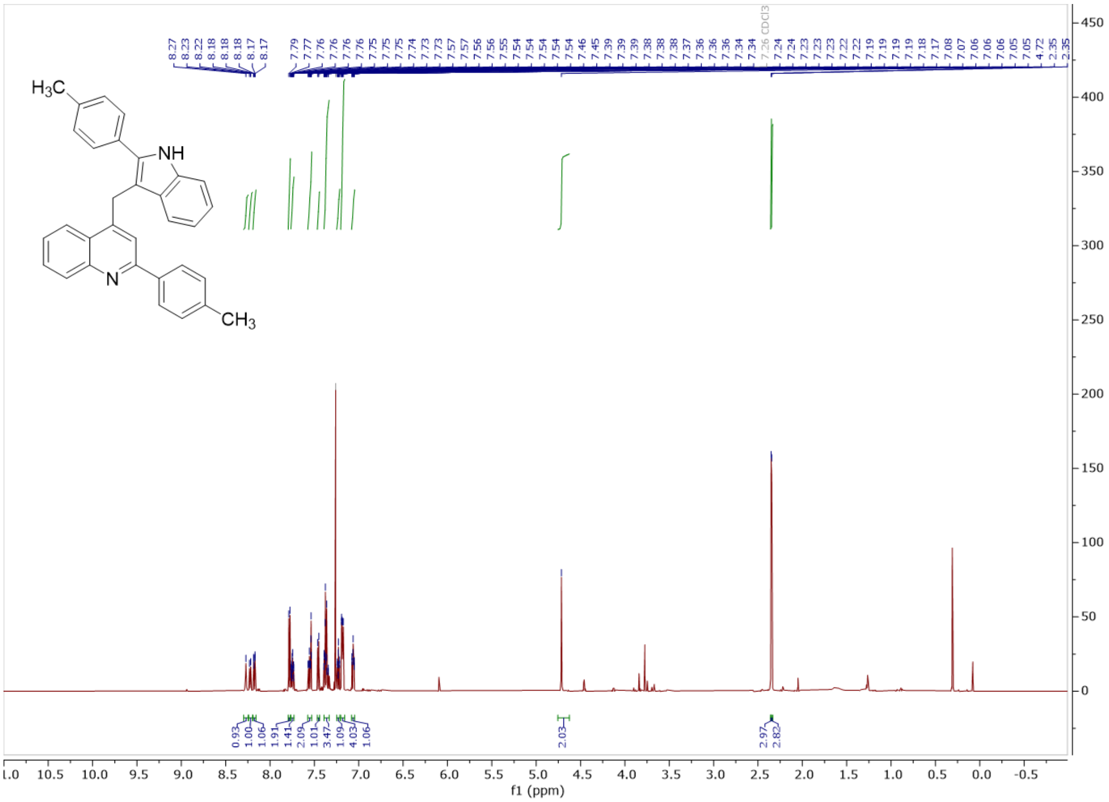

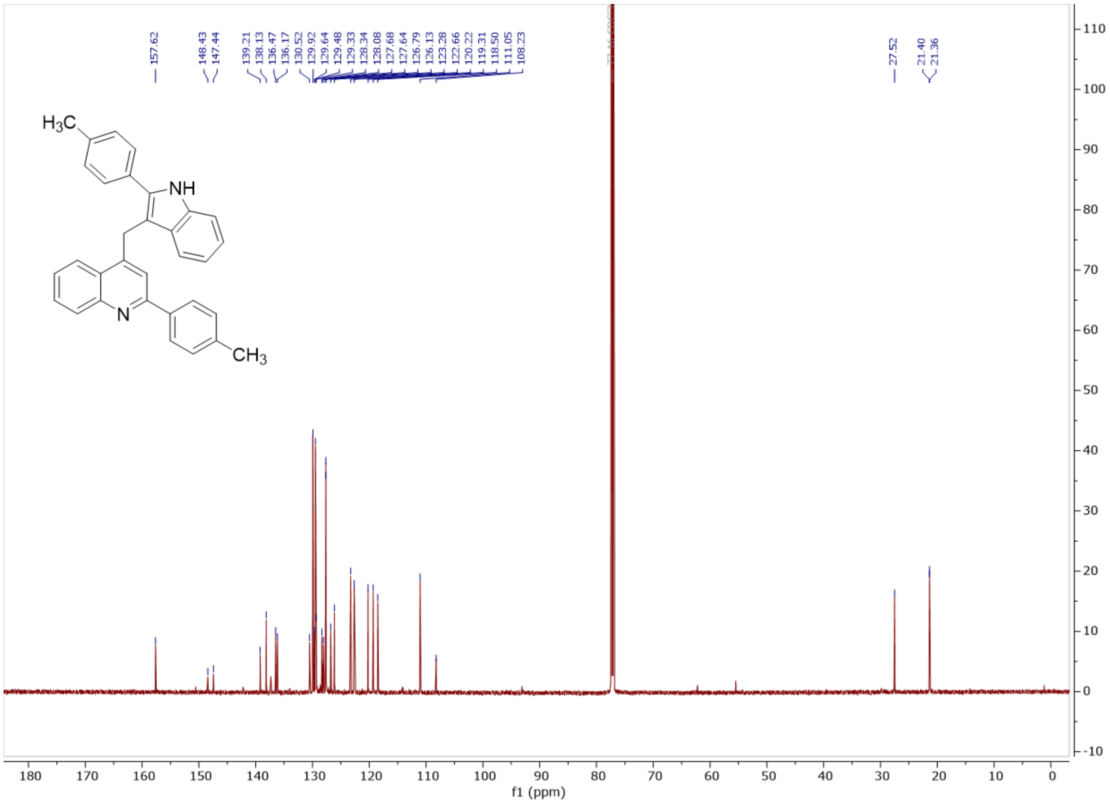

^{1}H NMR (599 MHz, $CDCl_3$) and ^{13}C NMR (151 MHz, $CDCl_3$) spectra for **2e'**

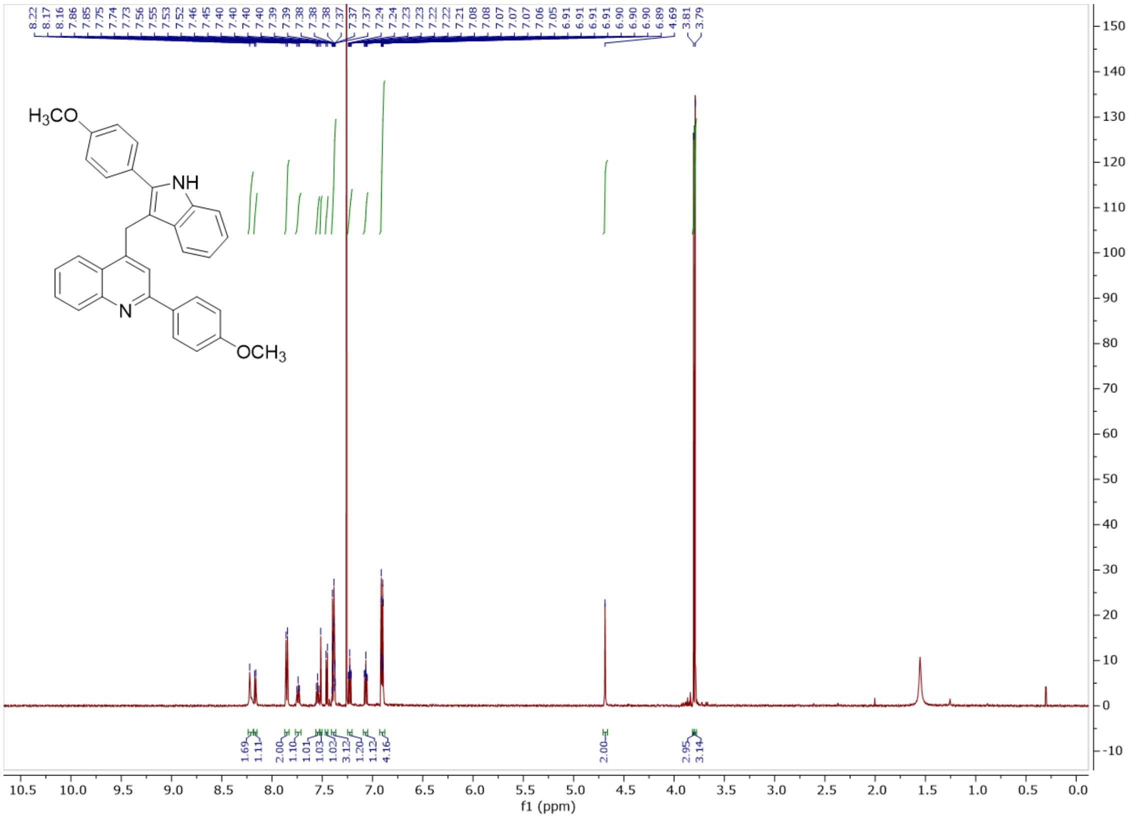

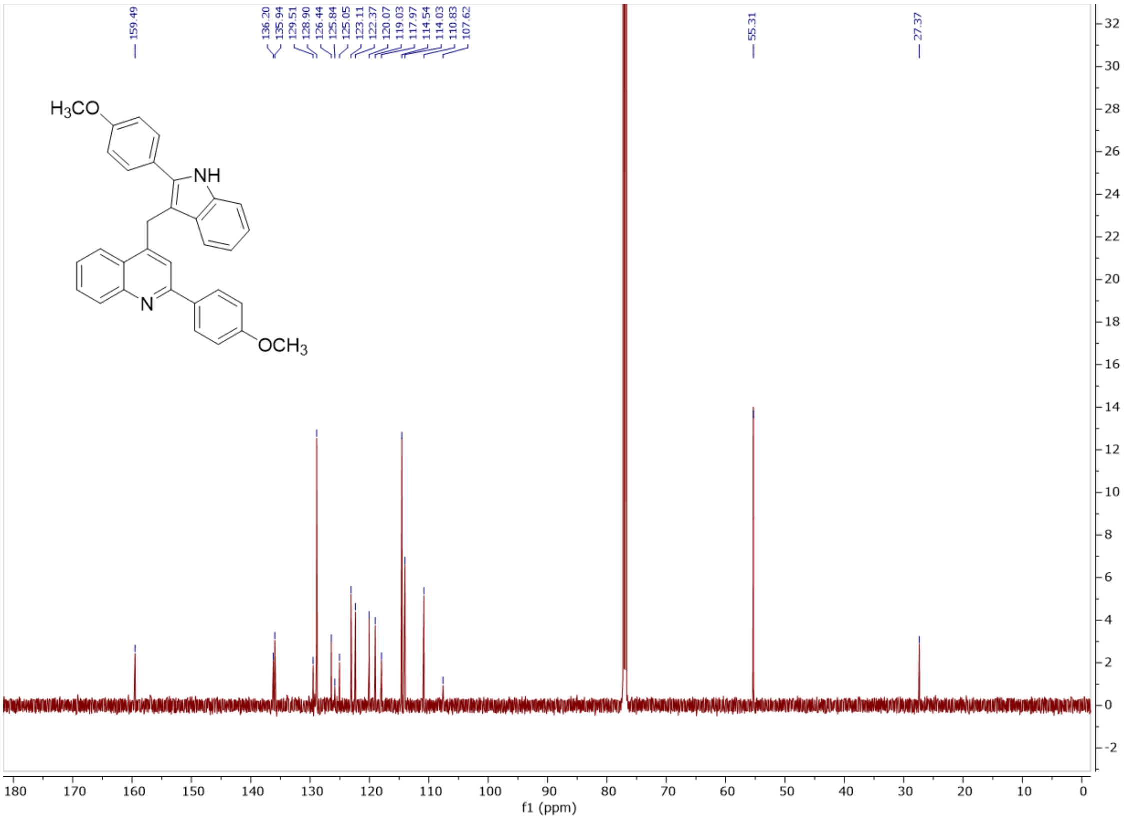

^{1}H NMR (300 MHz, $CDCl_3$) and ^{13}C NMR (76 MHz, $CDCl_3$) spectra for **2f'**

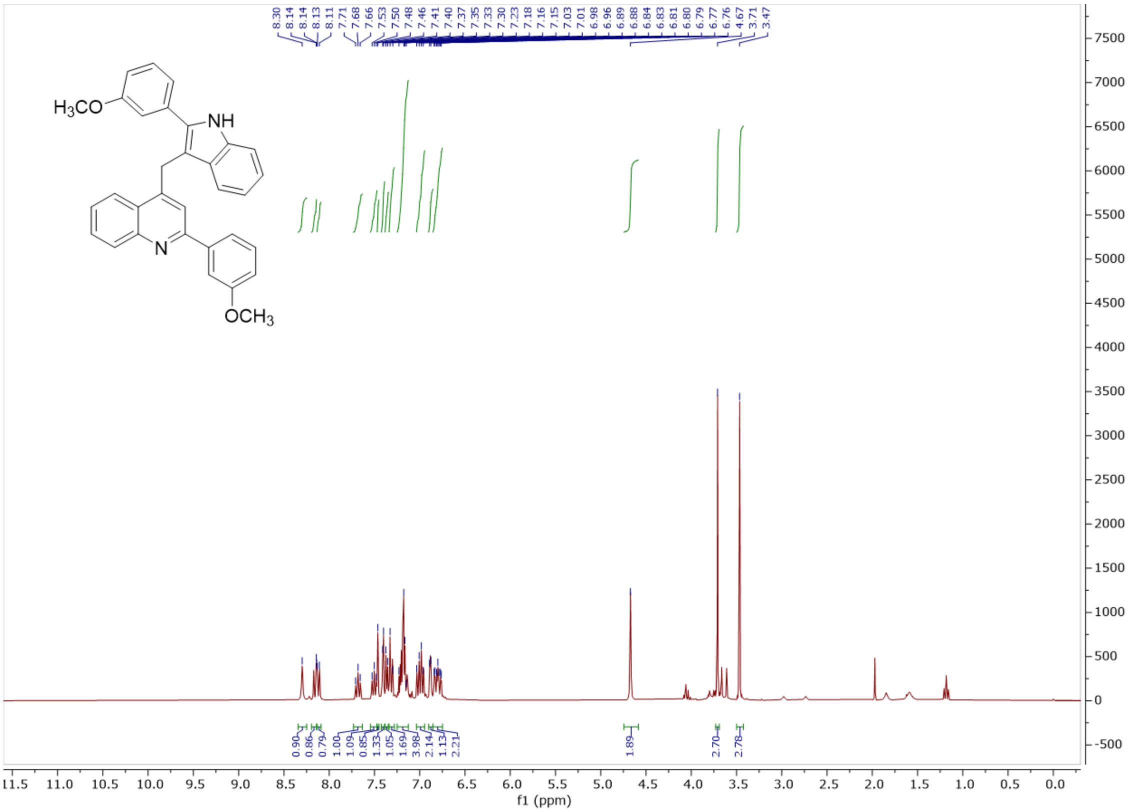

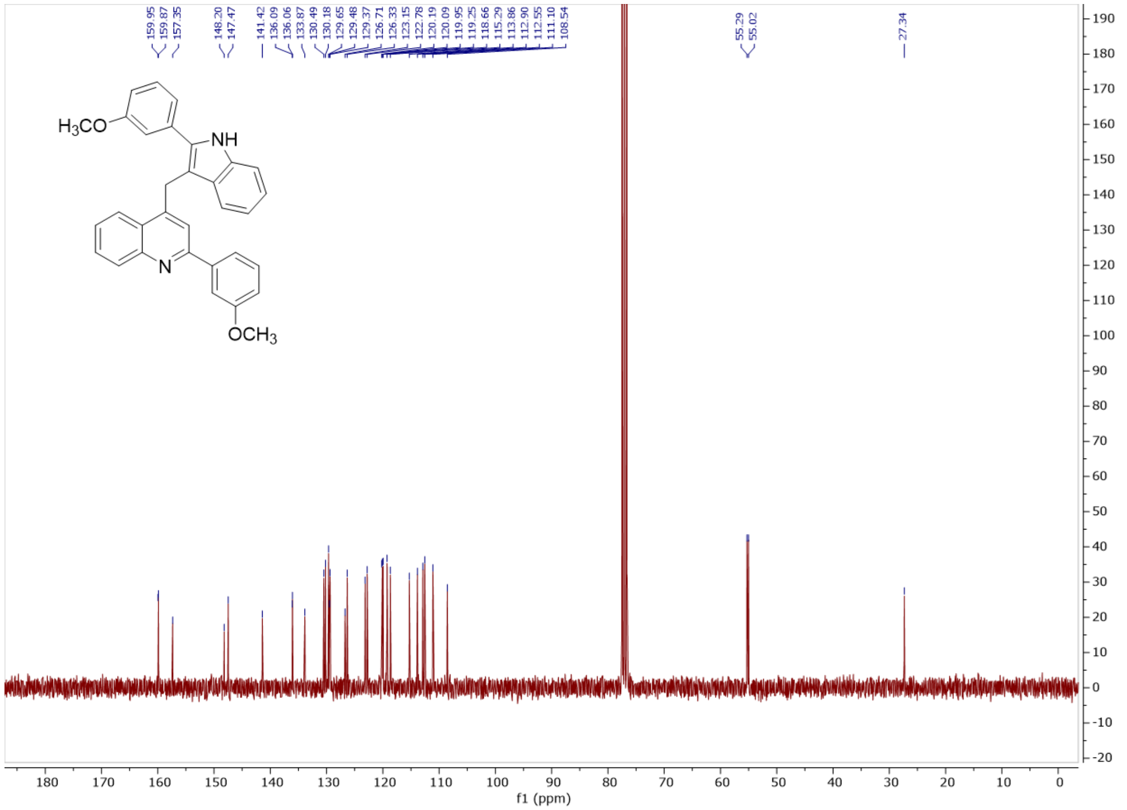

^{1}H NMR (599 MHz, $CDCl_3$), ^{13}C NMR (126 MHz, $CDCl_3$) and ^{19}F NMR (470 MHz, $CDCl_3$) spectra for **2g'**

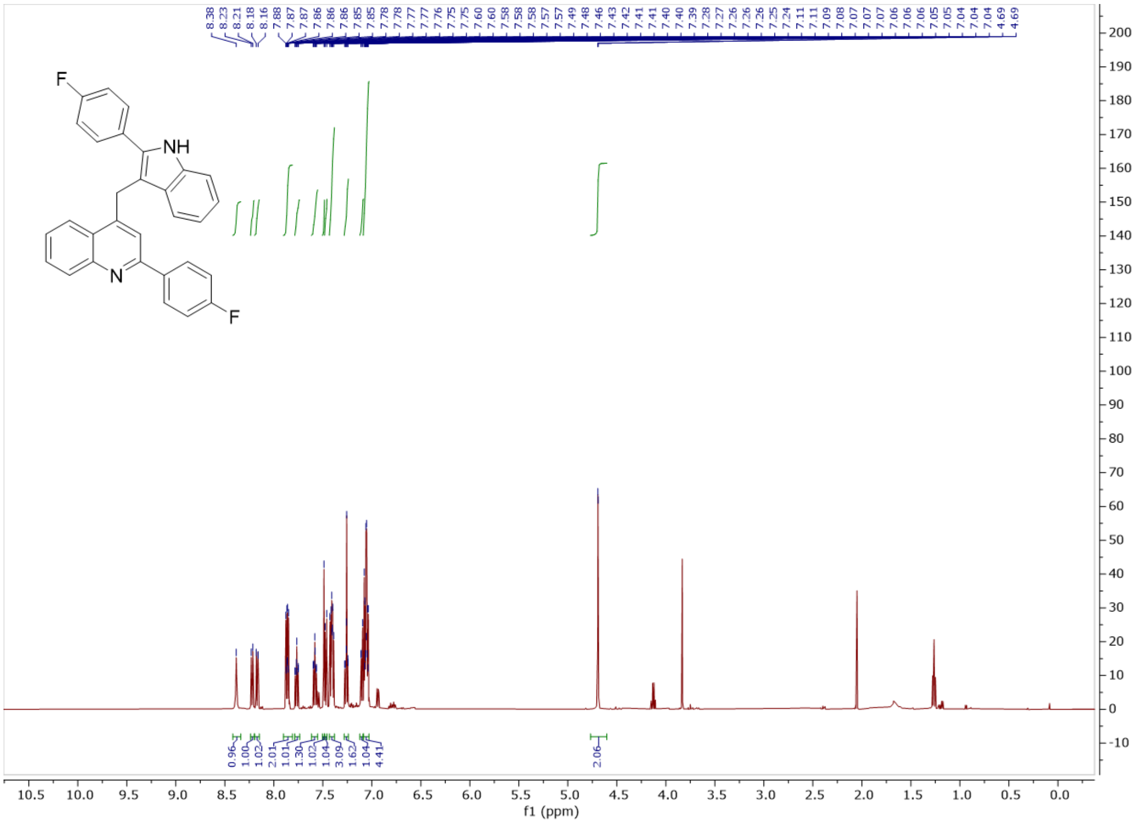

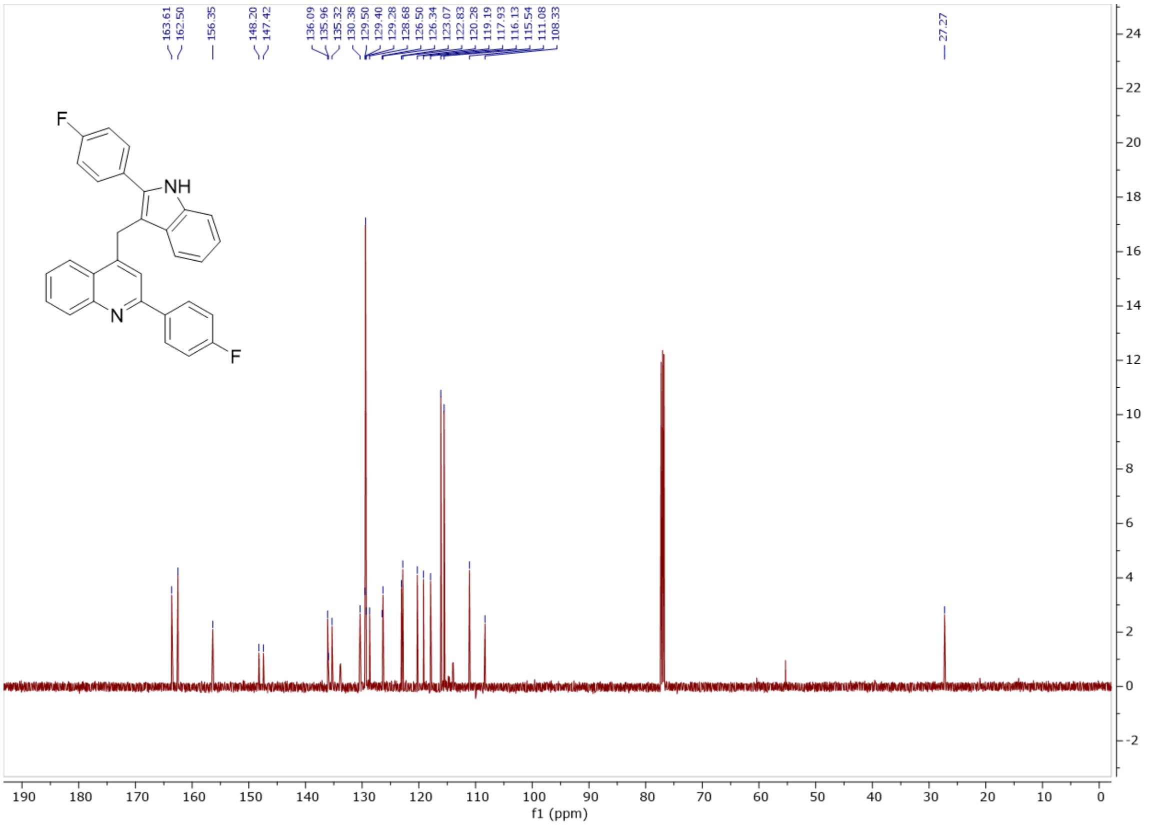

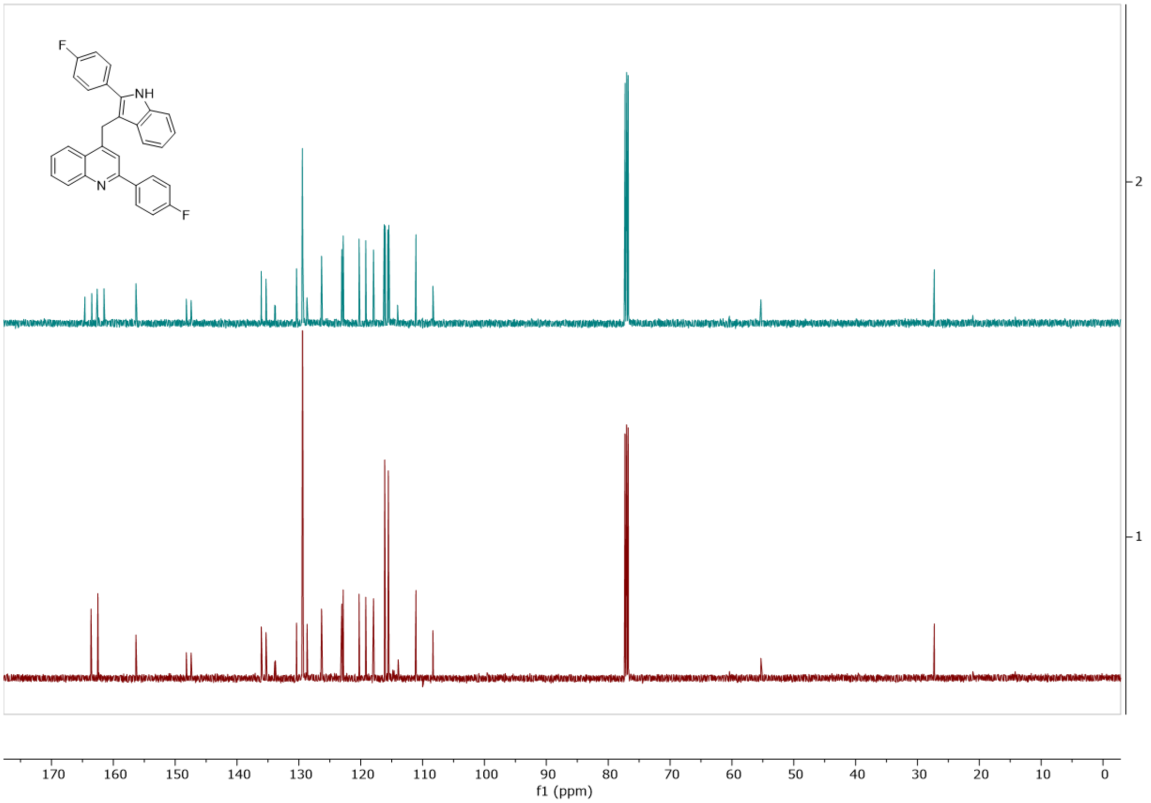
F
NH
N
F
2
1
170
160
150
140
130
120
110
100
90
80
70
60
50
40
30
20
10
0
f1 (ppm)

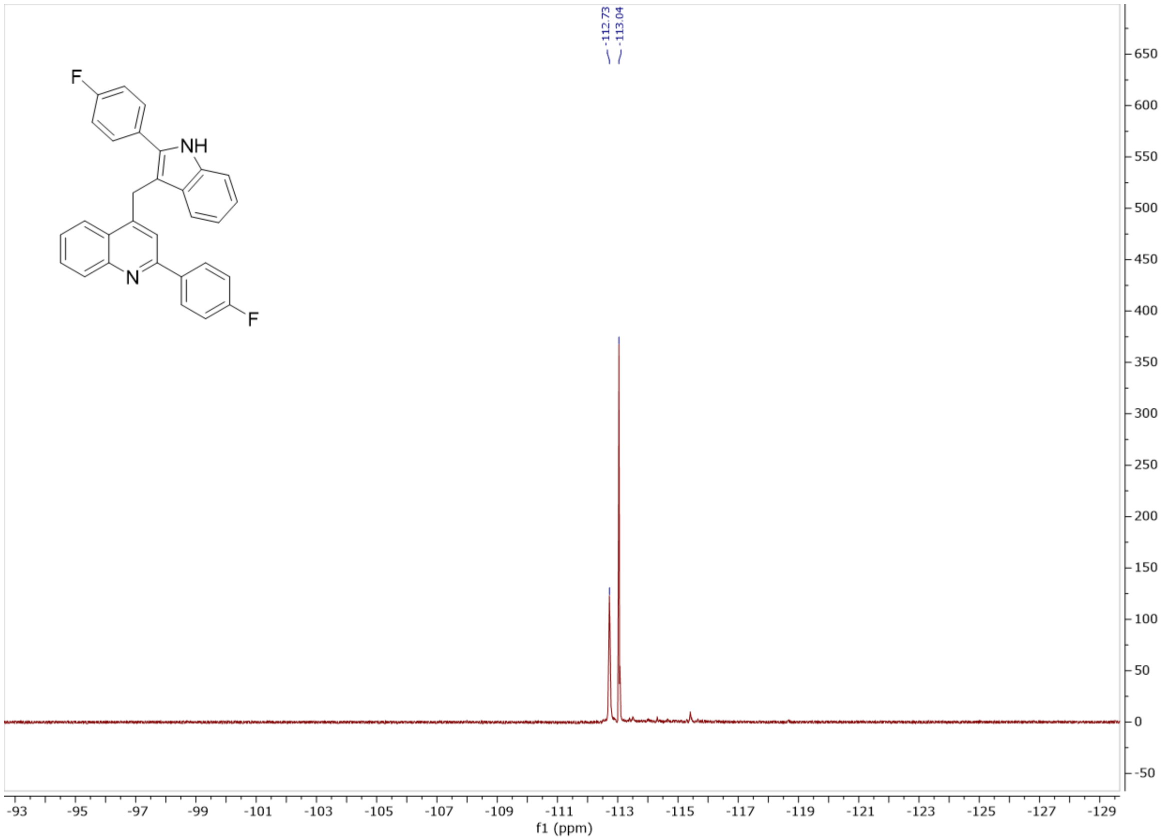
-112.73
-113.04
F
NH
N
F
650
600
550
500
450
400
350
300
250
200
150
100
50
0
-50
-93
-95
-97
-99
-101
-103
-105
-107
-109
-111
-113
-115
-117
-119
-121
-123
-125
-127
-129
f1 (ppm)

^{1}H NMR (400 MHz, $CDCl_3$) and ^{13}C NMR (101 MHz, $CDCl_3$) spectra for **2h'**

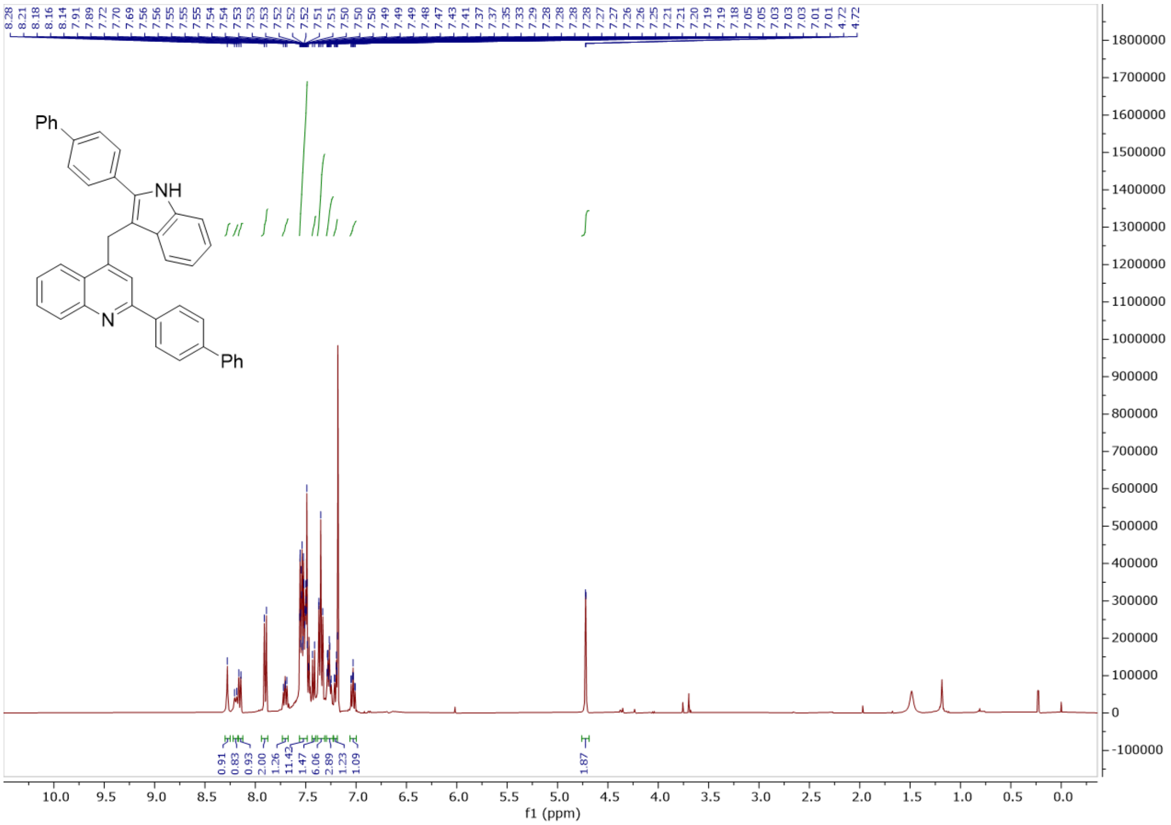

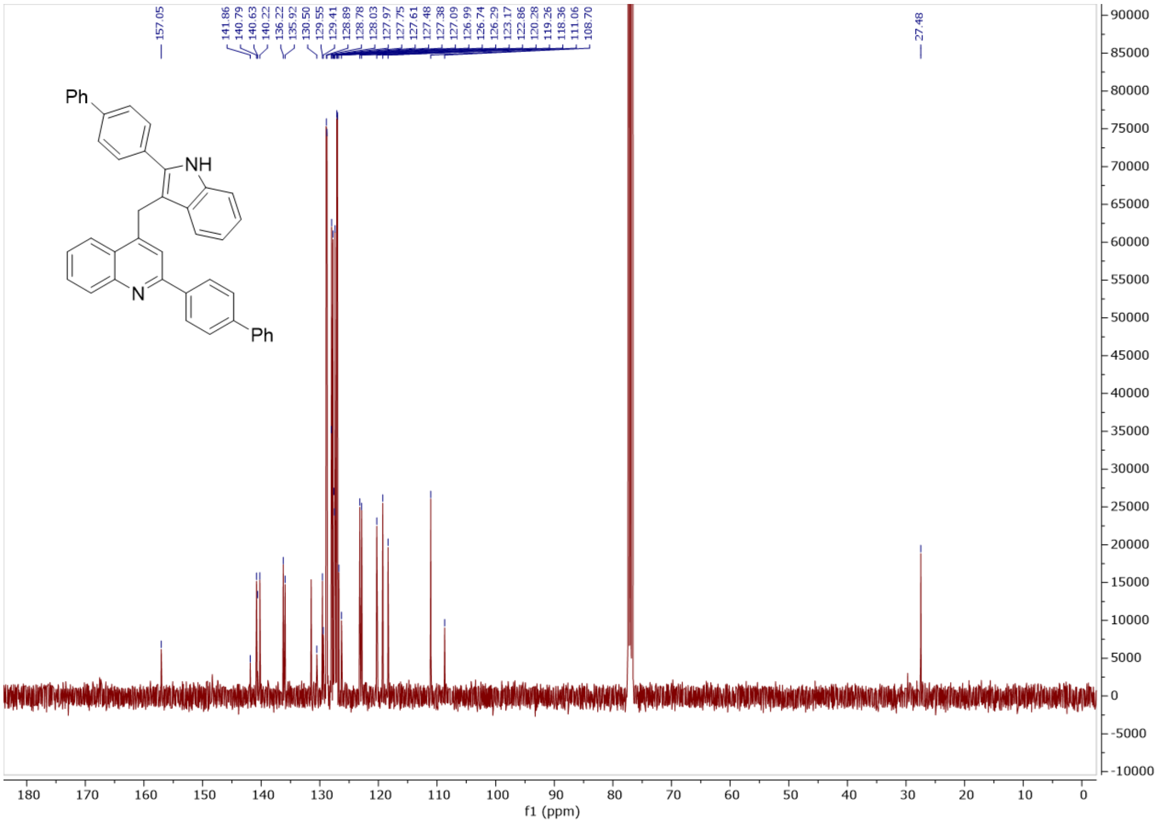

^{1}H NMR (300 MHz, $CDCl_3$) spectrum for *cis*-**2ag**

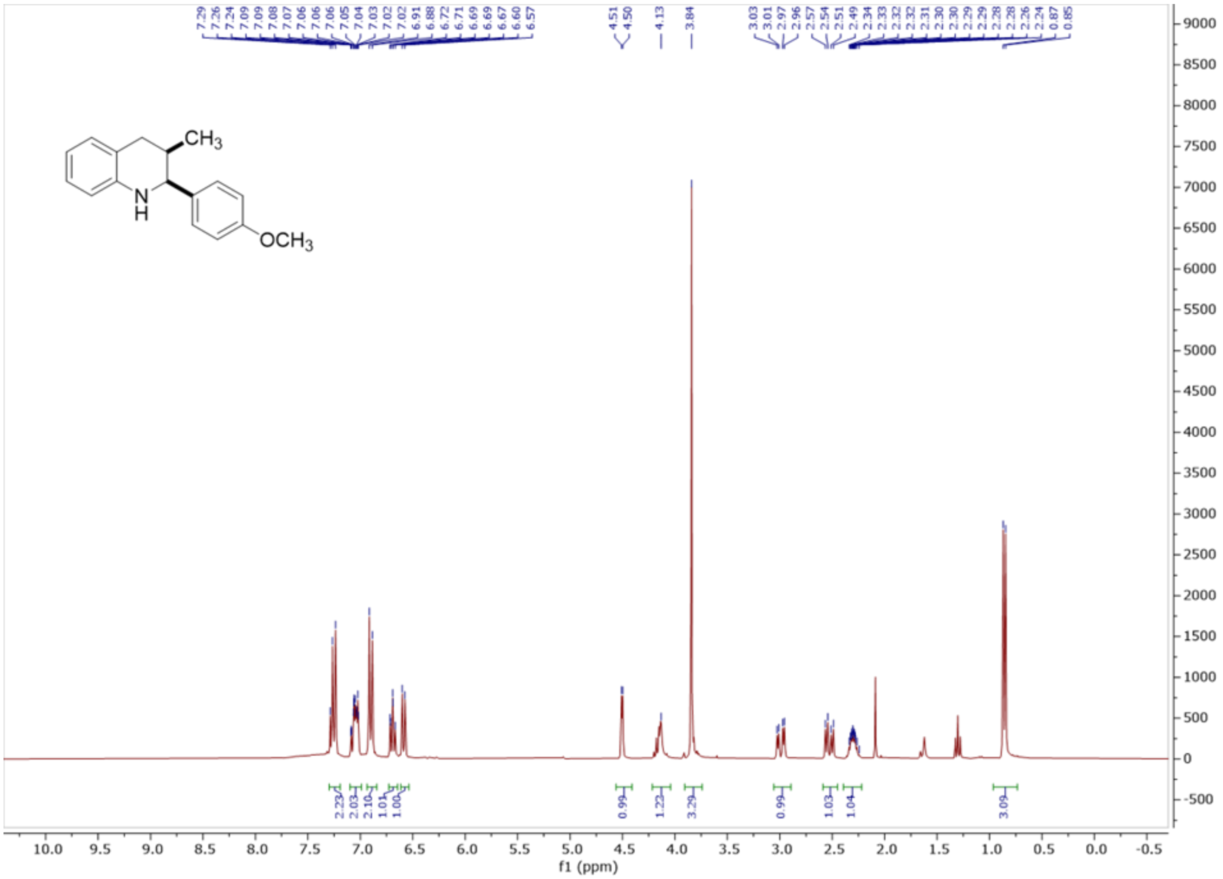

17. References

1. Xu, J.-x.; Pan, N.-l.; Chen, J.-x.; Zhao, J.-w., Visible-Light-Mediated Oxidative Cyclization of 2-Aminobenzyl Alcohols and Secondary Alcohols Enabled by an Organic Photocatalyst. *J. Org. Chem.* **2021,** *86* (15), 10747-10754.
2. Dang-Bao, T.; Pradel, C.; Favier, I.; Gómez, M., Making Copper(0) Nanoparticles in Glycerol: A Straightforward Synthesis for a Multipurpose Catalyst. *Adv. Synth. Catal.* **2017,** *359* (16), 2832-2846.
3. Ren, X.; Han, S.; Gao, X.; Li, J.; Zou, D.; Wu, Y.; Wu, Y., Direct arylation for the synthesis of 2-arylquinolines from N-methoxyquinoline-1-ium tetrafluoroborate salts and arylboronic acids. *Tetrahedron Lett.* **2018,** *59* (11), 1065-1068.
4. Kuriyama, M.; Matsuo, S.; Shinozawa, M.; Onomura, O., Ether-Imidazolium Carbenes for Suzuki–Miyaura Cross-Coupling of Heteroaryl Chlorides with Aryl/Heteroarylboron Reagents. *Org. Lett.* **2013,** *15* (11), 2716-2719.
5. Anderson, C. M.; Mastrocinque, C.; Greenberg, M. W.; McClellan, I. C.; Duman, L.; Oh, N.; Mastrocinque, F.; Pizzuto, M.; Tran, K.; Tanski, J. M., Synthesis, characterization, and photophysical properties of bismetalated platinum complexes with benzothiophene ligands. *J. Organomet. Chem.* **2019,** *882*, 10-17.
6. Santra, S.; Hota, P. K.; Bhattacharyya, R.; Bera, P.; Ghosh, P.; Mandal, S. K., Palladium Nanoparticles on Graphite Oxide: A Recyclable Catalyst for the Synthesis of Biaryl Cores. *Acs. Catal.* **2013,** *3* (12), 2776-2789.
7. Ji, J.; Jiang, L.; Wang, Z.; Bin, Z.; You, J.; Yang, Y., Copper-Catalyzed Oxidative C–H Annulation of Quinolines with Dichloroethane toward Benzoquinoliziniums Using an In Situ Activation Strategy. *Org. Lett.* **2022,** *24* (34), 6256-6260.
8. Li, C.; Li, J.; An, Y.; Peng, J.; Wu, W.; Jiang, H., Palladium-Catalyzed Allylic C–H Oxidative Annulation for Assembly of Functionalized 2-Substituted Quinoline Derivatives. *J. Org. Chem.* **2016,** *81* (24), 12189-12196.
9. Xu, J.; Sun, J.; Zhao, J.; Huang, B.; Li, X.; Sun, Y., Palladium-catalyzed synthesis of quinolines from allyl alcohols and anilines. *RSC Advances* **2017,** *7* (58), 36242-36245.
10. Zhuo, F.-F.; Xie, W.-W.; Yang, Y.-X.; Zhang, L.; Wang, P.; Yuan, R.; Da, C.-S., TMEDA-Assisted Effective Direct Ortho Arylation of Electron-Deficient N-Heteroarenes with Aromatic Grignard Reagents. *J. Org. Chem.* **2013,** *78* (7), 3243-3249.
11. Duvelleroy, D.; Perrio, C.; Parisel, O.; Lasne, M.-C., Rapid synthesis of quinoline-4-carboxylic acid derivatives from arylimines and 2-substituted acrylates or acrylamides under indium(iii) chloride and microwave activations. Scope and limitations of the reaction. *Org. Biomol. Chem.* **2005,** *3* (20), 3794-3804.
12. Saunthwal, R. K.; Patel, M.; Verma, A. K., Metal- and Protection-Free [4 + 2] Cycloadditions of Alkynes with Azadienes: Assembly of Functionalized Quinolines. *Org. Lett.* **2016,** *18* (9), 2200-2203.
13. Yang, G.; Yao, L.; Mao, G.; Deng, G.-J.; Xiao, F., Synthesis of Indenoquinolinones and 2-Substituted Quinolines via [4 + 2] Cycloaddition Reaction. *J. Org. Chem.* **2022,** *87* (21), 14523-14535.
14. Hu, X.-H.; Hu, X.-P., Highly Diastereo- and Enantioselective Ir-Catalyzed Hydrogenation of 2,3-Disubstituted Quinolines with Structurally Fine-Tuned Phosphine–Phosphoramidite Ligands. *Org. Lett.* **2019,** *21* (24), 10003-10006.

15. Das, J.; Singh, K.; Vellakkaran, M.; Banerjee, D., Nickel-Catalyzed Hydrogen-Borrowing Strategy for α-Alkylation of Ketones with Alcohols: A New Route to Branched gem-Bis(alkyl) Ketones. *Org. Lett.* **2018,** *20* (18), 5587-5591.
16. Banik, A.; Datta, P.; Mandal, S. K., C-Alkylation by Phenalenyl-Based Molecule via a Borrowing Hydrogen Pathway. *Org. Lett.* **2023,** *25* (8), 1305-1309.
17. Xu, Z.-M.; Hu, Z.; Huang, Y.; Bao, S.-J.; Niu, Z.; Lang, J.-P.; Al-Enizi, A. M.; Nafady, A.; Ma, S., Introducing Frustrated Lewis Pairs to Metal–Organic Framework for Selective Hydrogenation of N-Heterocycles. *J. Am. Chem. Soc.* **2023,** *145* (27), 14994-15000.
18. Ueda, M.; Kawai, S.; Hayashi, M.; Naito, T.; Miyata, O., Efficient Entry into 2-Substituted Tetrahydroquinoline Systems through Alkylative Ring Expansion: Stereoselective Formal Synthesis of (±)-Martinellic Acid. *J. Org. Chem.* **2010,** *75* (3), 914-921.
19. Maji, S.; Sarkar, P.; Das, A.; Pati, S. K.; Mandal, S. K., Benzimidazolylidene-Stabilized Borenium Ion for Catalytic Hydrogenation of N-Heterocycles. *Inorg. Chem.* **2022,** *61* (36), 14282-14287.
20. Wu, J.; Wang, C.; Tang, W.; Pettman, A.; Xiao, J., The Remarkable Effect of a Simple Ion: Iodide-Promoted Transfer Hydrogenation of Heteroaromatics. *Chem. Eur. J.* **2012,** *18* (31), 9525-9529.
21. Jiang, X.; Wang, C.; Wei, Y.; Xue, D.; Liu, Z.; Xiao, J., A General Method for N-Methylation of Amines and Nitro Compounds with Dimethylsulfoxide. *Chem. Eur. J.* **2014,** *20* (1), 58-63.
22. Wang, Y.; Dong, B.; Wang, Z.; Cong, X.; Bi, X., Silver-Catalyzed Reduction of Quinolines in Water. *Org. Lett.* **2019,** *21* (10), 3631-3634.
23. Tu, X.-F.; Gong, L.-Z., Highly Enantioselective Transfer Hydrogenation of Quinolines Catalyzed by Gold Phosphates: Achiral Ligand Tuning and Chiral-Anion Control of Stereoselectivity. *Angew. Chem. Int. Ed.* **2012,** *51* (45), 11346-11349.
24. Chen, M.; Sun, J., Catalytic Asymmetric N-Alkylation of Indoles and Carbazoles through 1,6-Conjugate Addition of Aza-para-quinone Methides. *Angew. Chem. Int. Ed.* **2017,** *56* (16), 4583-4587.
25. Li, X.; Tian, J.-J.; Liu, N.; Tu, X.-S.; Zeng, N.-N.; Wang, X.-C., Spiro-Bicyclic Bisborane Catalysts for Metal-Free Chemoselective and Enantioselective Hydrogenation of Quinolines. *Angew. Chem. Int. Ed.* **2019,** *58* (14), 4664-4668.
26. Zhang, J.; Mück-Lichtenfeld, C.; Studer, A., Photocatalytic phosphine-mediated water activation for radical hydrogenation. *Nature* **2023,** *619* (7970), 506-513.
27. Mitra, R.; Zhu, H.; Grimme, S.; Niemeyer, J., Functional Mechanically Interlocked Molecules: Asymmetric Organocatalysis with a Catenated Bifunctional Brønsted Acid. *Angew. Chem. Int. Ed.* **2017,** *56* (38), 11456-11459.
28. Liang, Y.; Luo, J.; Diskin-Posner, Y.; Milstein, D., Designing New Magnesium Pincer Complexes for Catalytic Hydrogenation of Imines and N-Heteroarenes: H2 and N–H Activation by Metal–Ligand Cooperation as Key Steps. *J. Am. Chem. Soc.* **2023,** *145* (16), 9164-9175.
29. Zhao, X.; Xiao, J.; Tang, W., Enantioselective Reduction of 3-Substituted Quinolines with a Cyclopentadiene-Based Chiral Brønsted Acid. *Synthesis* **2017,** *49* (14), 3157-3164.

30. Gao, Y.; Kubota, K.; Ito, H., Mechanochemical Approach for Air-Tolerant and Extremely Fast Lithium-Based Birch Reductions in Minutes. *Angew. Chem. Int. Ed.* **2023,** *62* (21), e202217723.
31. Chen, F.; Surkus, A.-E.; He, L.; Pohl, M.-M.; Radnik, J.; Topf, C.; Junge, K.; Beller, M., Selective Catalytic Hydrogenation of Heteroarenes with N-Graphene-Modified Cobalt Nanoparticles (Co3O4–Co/NGr@α-Al2O3). *J. Am. Chem. Soc.* **2015,** *137* (36), 11718-11724.
32. Papa, V.; Fessler, J.; Zaccaria, F.; Hervochon, J.; Dam, P.; Kubis, C.; Spannenberg, A.; Wei, Z.; Jiao, H.; Zuccaccia, C.; Macchioni, A.; Junge, K.; Beller, M., Efficient Hydrogenation of N-Heterocycles Catalyzed by NNP–Manganese(I) Pincer Complexes at Ambient Temperature. *Chem. Eur. J.* **2023,** *29* (2), e202202774.
33. Barrios-Rivera, J.; Xu, Y.; Wills, M., Asymmetric Transfer Hydrogenation of Unhindered and Non-Electron-Rich 1-Aryl Dihydroisoquinolines with High Enantioselectivity. *Org. Lett.* **2020,** *22* (16), 6283-6287.
34. Zhang, Z.; Du, H., Enantioselective Metal-Free Hydrogenations of Disubstituted Quinolines. *Org. Lett.* **2015,** *17* (24), 6266-6269.
35. Kaga, A.; Hayashi, H.; Hakamata, H.; Oi, M.; Uchiyama, M.; Takita, R.; Chiba, S., Nucleophilic Amination of Methoxy Arenes Promoted by a Sodium Hydride/Iodide Composite. *Angew. Chem. Int. Ed.* **2017,** *56* (39), 11807-11811.
36. Gandhamsetty, N.; Park, S.;Chang, S., Boron-Catalyzed Hydrogenative Reduction of Substituted Quinolines to Tetrahydroquinolines with Hydrosilanes. *Synlett* **2017**, *28* (18), 2396–2400.
37. Perdew, J. P., Ernzerhof, M., Burke, K. Rationale for mixing exact exchange with density functional approximations. *J. Chem. Phys.* **1996***, 105* (22), 9982-9985.
38. Adamo, C., Barone, V. Toward reliable density functional methods without adjustable parameters: The PBE0 model. *J. Chem. Phy.* **1999**, *110* (13), 6158-6170.
39. Grimme, S., Antony, J., Ehrlich, S., Krieg, H. A consistent and accurate ab initio parametrization of density functional dispersion correction (DFT-D) for the 94 elements H-Pu. *J. Chem. Phys.* **2010**, *132* (15), 154104.
40. Grimme, S., Ehrlich., S., Goerigk, L. Effect of the damping function in dispersion corrected density functional theory. *J. Comput. Chem.* **2011***, 32* (7), 1456–1465.
41. Weigend, F., Ahlrichs, R. Balanced basis sets of split valence. triple zeta valence and quadruple zeta valence quality for H to Rn: Design and assessment of accuracy. *Phys. Chem. Chem. Phys.* **2005**, *7* (18), 3297–3305.
42. Grimme, S. Supramolecular binding thermodynamics by dispersion-corrected density functional theory. *Chem. Eur. J.* **2012**, *18* (32), 9955-9964.
43. Goerigk, L., Grimme, S. Efficient and accurate double-hybrid-meta-GGA density functionals – Evaluation with the extended GMTKN30 Database for General Main Group Thermochemistry, Kinetics, and Noncovalent Interactions *J. Chem. Theory Comput.* **2011**, *7* (2), 291-309.
44. Mills, G., Jónsson, H., Schenter, G. Reversible work transition state theory: application to dissociative adsorption of hydrogen. *Surface Science* **1995**, *324* (2-3), 305-337.
45. Henkelman, G., Jónsson, H. Improved tangent estimate in the nudged elastic band method for finding minimum energy paths and saddle points. *J. Chem. Phys.* **2000***, 113* (22), 9978-9985.

46. Grimme, S., Brandenburg, J. G., Bannwarth, C., Hansen, A. Consistent structures and interactions by density functional theory with small atomic orbital basis sets. *J. Chem. Phys.* **2015**, *143* (5), 054107.
47. Klamt, A. Conductor-like screening model for real solvents: A new approach to the quantitative calculation of solvation phenomena. *J. Phys. Chem.* **1995**, *99* (7), 2224-2235.
48. Klamt, A., Jonas V., Bürger, T., Lohrenz, J. C. Refinement and parametrization of COSMO-RS. *J. Phys. Chem. A* **1998**, *102* (26), 5074-5085.
49. TURBOMOLE V7.8.1 (2024), a development of University of Karlsruhe and Forschungszentrum Karlsruhe GmbH, 1989-2007, TURBOMOLE GmbH, since 2007; available from http://www.turbomole.com.
50. Neese, F. The ORCA program system, *Wiley Interdisciplinary Reviews: Computational Molecular Science,* **2012**, *2* (1), 73-78.
